# The swan genome and transcriptome, it is not all black and white

**DOI:** 10.1186/s13059-022-02838-0

**Published:** 2023-01-23

**Authors:** Anjana C. Karawita, Yuanyuan Cheng, Keng Yih Chew, Arjun Challagulla, Robert Kraus, Ralf C. Mueller, Marcus Z. W. Tong, Katina D. Hulme, Helle Bielefeldt-Ohmann, Lauren E. Steele, Melanie Wu, Julian Sng, Ellesandra Noye, Timothy J. Bruxner, Gough G. Au, Suzanne Lowther, Julie Blommaert, Alexander Suh, Alexander J. McCauley, Parwinder Kaur, Olga Dudchenko, Erez Aiden, Olivier Fedrigo, Giulio Formenti, Jacquelyn Mountcastle, William Chow, Fergal J. Martin, Denye N. Ogeh, Françoise Thiaud-Nissen, Kerstin Howe, Alan Tracey, Jacqueline Smith, Richard I. Kuo, Marilyn B. Renfree, Takashi Kimura, Yoshihiro Sakoda, Mathew McDougall, Hamish G. Spencer, Michael Pyne, Conny Tolf, Jonas Waldenström, Erich D. Jarvis, Michelle L. Baker, David W. Burt, Kirsty R. Short

**Affiliations:** 1 School of Chemistry and Molecular Biosciences, The University of Queensland, St Lucia, QLD 4072 Australia; 2 Commonwealth Scientific and Industrial Research Organisation, Australian Centre for Disease Preparedness, 5 Portarlington Road, Geelong, VIC 3220 Australia; 3 School of Life and Environmental Sciences, The University of Sydney, Sydney, NSW 2006 Australia; 4 Department of Migration, Max Planck Institute of Animal Behavior, Radolfzell, 78315 Germany; 5 Department of Biology, University of Konstanz, Konstanz, 78457 Germany; 6 Institute for Molecular Bioscience, The University of Queensland, St Lucia, QLD 4072 Australia; 7 Department of Organismal Biology – Systematic Biology, Evolutionary Biology Centre, Uppsala University, Science for Life Laboratory, Uppsala, 752 36 Sweden; 8 The New Zealand Institute for Plant & Food Research Ltd, Nelson, 7010 New Zealand; 9 School of Biological Sciences, University of East Anglia, Norwich Research Park, Norwich, NR4 7TU UK; 10 School of Agriculture and Environment, The University of Western Australia, Perth, WA 6009 Australia; 11 The Centre for Genome Architecture, Department of Molecular and Human Genetics, Baylor College of Medicine, Houston, TX 77030 USA; 12 Centre for Theoretical Biological Physics and Department of Computer Science, Rice University, Houston, TX 77030 USA; 13 Broad Institute of MIT and Harvard, Cambridge, MA 02139 USA; 14 Shanghai Institute for Advanced Immunochemical Studies, ShanghaiTech, Pudong, 201210 China; 15 The Vertebrate Genome Laboratory, The Rockefeller University, NY, 10065 USA; 16 Tree of Life, Welcome Sanger Institute, Cambridge, CB10 1SA UK; 17 European Molecular Biology Laboratory, European Bioinformatics Institute, Wellcome Genome Campus, Hinxton, Cambridge, CB10 1SD UK; 18 National Centre for Biotechnology Information, National Library of Medicine, National Institutes of Health, Bethesda, MD USA; 19 The Roslin Institute and Royal (Dick) School of Veterinary Studies, University of Edinburgh, Easter Bush Campus, Midlothian, EH25 9RG UK; 20 School of Biosciences, The University of Melbourne, Melbourne, VIC 3052 Australia; 21 Faculty of Veterinary Medicine, Hokkaido University, Sapporo, Hokkaido 060-0818 Japan; 22 New Zealand Fish & Game – Eastern Region, Rotorua, 3046 New Zealand; 23 Department of Zoology, University of Otago, Dunedin, 9054 New Zealand; 24 Currumbin Wildlife Sanctuary, Currumbin, QLD 4223 Australia; 25 Centre for Ecology and Evolution in Microbial Model Systems (EEMiS), Linnaeus University, Kalmar, SE-391 82 Sweden

**Keywords:** Genomes, Virology, Black swan

## Abstract

**Background:**

The Australian black swan (*Cygnus atratus*) is an iconic species with contrasting plumage to that of the closely related northern hemisphere white swans. The relative geographic isolation of the black swan may have resulted in a limited immune repertoire and increased susceptibility to infectious diseases, notably infectious diseases from which Australia has been largely shielded. Unlike mallard ducks and the mute swan (*Cygnus olor*), the black swan is extremely sensitive to highly pathogenic avian influenza. Understanding this susceptibility has been impaired by the absence of any available swan genome and transcriptome information.

**Results:**

Here, we generate the first chromosome-length black and mute swan genomes annotated with transcriptome data, all using long-read based pipelines generated for vertebrate species. We use these genomes and transcriptomes to show that unlike other wild waterfowl, black swans lack an expanded immune gene repertoire, lack a key viral pattern-recognition receptor in endothelial cells and mount a poorly controlled inflammatory response to highly pathogenic avian influenza. We also implicate genetic differences in *SLC45A2* gene in the iconic plumage of the black swan.

**Conclusion:**

Together, these data suggest that the immune system of the black swan is such that should any avian viral infection become established in its native habitat, the black swan would be in a significant peril.

**Supplementary Information:**

The online version contains supplementary material available at 10.1186/s13059-022-02838-0.

## Background

The distinctive black plumage of the native Australian black swan (*Cygnus atratus*) is in stark contrast to the white swans that are native to Europe and North America. This unique feature has resulted in the black swan playing an important role in Australian heraldry and culture. The limited native geographic range (Australia) and relative isolation of the black swan has direct consequences for its immune repertoire and susceptibility to infectious disease common to other parts of the world. Specifically, geographic isolation can result in founder effects and reduced immune diversity as a result of limited pathogen challenge [1].

The native Australian black swan has a remarkably distinct response to infection by highly pathogenic avian influenza (HPAI) virus compared to the closely related white swans (e.g., the mute swan; *Cygnus olor*) and other waterfowl [2, 3]. Unlike mallard ducks and mute swans, the black swan is extremely sensitive to HPAI, succumbing to the disease within 2 to 3 days post-infection. This disease pathogenesis mirrors that of infected chickens, viewed as the most susceptible species to HPAI [3]. One of the striking features common to both black swans and chickens is that HPAI viruses preferentially infect endothelial cells, which may contribute to the disease severity in these two species [3]. These experimental studies are consistent with reports of natural infections, which suggest that captive black swans quickly succumb to HPAI while co-housed mute swans survive the infection [4].

Comparative genomics has played an important role in understanding species-dependent differences in HPAI pathogenesis [5] while also revealing the unique immune systems of many native Australian fauna [6]. However, comparative genomics is contingent upon the availability of high-quality species-specific genomes and transcriptomes.

Here, we generate the first black and mute swan reference genomes and transcriptomes, including the transcriptional response of primary black swan endothelial cells to HPAI. These data show that the black swan has numerous unique characteristics including (i) lack of an expanded immune gene repertoire, (ii) undetectable *Toll-like Receptor (TLR) 7* gene expression in infected endothelial cells, and (iii) a dysregulated pro-inflammatory response to viral infection that is likely to leave the species highly susceptible to viral infections such as HPAI. It is also likely that genetic differences in melanin production contribute to the distinctive black plumage of the black swan.

## Results

### Genome landscape

The chromosome-length reference genomes for black and mute swan were constructed according to a Pacbio continuous long-read-based (CLR) DNA Zoo pipeline and Vertebrate Genomes Pipeline 1.5 [7], respectively (Additional file 1: Figures S1 and S2). This included scaffolding contigs with Hi-C (Hi-C contact maps; Additional file 1: Figure S3) and Bionano maps. Curations of the assemblies identified scaffolds representing 34 autosomes plus the Z sex chromosome and were named according to the descending order of the physical size. Others were nominated as unplaced scaffolds due to their lack of resolution to be placed as chromosomes. We assigned final scaffolds to 35 chromosomes and 36 chromosomes for black swan and mute swans respectively (including the sex chromosome), based on the physical size. The W chromosome was not assigned a scaffold in black swan as the DNA was derived from a male. The expected diploid number of chromosomes in the mute swan is 80 [8]. The black swan genome was sequenced using 90x PacBio CLR coverage, generating a 1.12 Gb reference assembly. The mute swan genome was sequenced using 60x PacBio CLR coverage, generating a 1.13 Gb reference assembly. The details of both genomes, including a comparison to the latest VGP (Vertebrate Genome Project) chicken (*Gallus gallus*) and mallard duck (*Anas platyrhynchos*) genomes, are shown in Table 1. For each genome, we estimated the heterozygosity, QV, and completeness values using Merqury based on the highly accurate Illumina® short reads (Additional file 2: Table S1 and Additional file 3: Online Methods).

**Table 1:** Comparison of genomes between black and mute swans (generated herein) and published high-quality avian genomes generated by the vertebrate genome project (VGP).

Species	Assembly size (estimated size) (Gb)	G + C content (%) Macro-chromosome^a^	G + C content (%) Micro-chromosome^b^	Contig N50 (Mb)	Contig L50	Number of contigs	Scaffold N50 (Mb)	Scaffold L50	No. scaffolds	ISO-seq coverage^c^	Coding genes	Non-coding genes	Pseudogenes	Ref
Black swan	1.12	47	52	29	12	697	84.6	4	567	68.87% of the genome	15478	1792	104	This study
Mute swan	1.13	47	53	8.3	39	621	81.5	4	170	54.36% of the genome	14791	2720	126	This study
Chicken	1.09	40	48	18.8	18	676	90.9	4	213	NA	16878	7166	312	bGalGal1.mat.broiler.GRCg7b (GCF_016699485.2)
Duck	1.18	40.72	46.51	5.7	57	1660	76.3	5	755	NA	16618	9285	49	ZJU1.0 (GCF_015476345.1)

^a^ Macro-chromosomes > 10 Mbps

^b^Micro-chromosomes < 10 Mbps

^c^Of the largest 34 chromosomes

The total classified repeat content of the genome was 10.56% for the black swan, with 1.71% unclassified repeats, and 10.76% for the mute swan genome, with 1.55% unclassified. This is lower than the repeat content recorded in the chicken (15%) and the mallard duck (17%) [9, 10].

The black and mute swan genomes were annotated with RNASeq and IsoSeq transcriptome data, homology-based alignments with other species and with bioinformatically inferred gene predictions, according to the methods listed in Additional file 1: Figure S4.

The completeness of the black and mute swan genomes was assessed using the Core Eukaryotic Genes Mapping (CEGMA) and Benchmark Universal Single Copy Orthologues (BUSCO) analyses and compared to the chicken and mallard duck genome (Additional file 4: Table S2, Additional file 5: Table S3). Notably, the black swan genome had the highest complete BUSCOs (8093), followed by the chicken (8054) and the mute swan (8010) genomes. While the chicken genome had the highest complete core-eukaryotic gene content (224), this was only marginally higher than that of the mute (221) and black swan (219) genomes.

One-to-one alignment between the black and mute swan genomes showed 98.35% average nucleotide identity between the 34 autosomes of the black and mute swans (Additional file 1: Figure S5). The Z chromosome of the black and mute swan had several large (>1kb) structural variants (Additional file 1: Figure S6), but were otherwise largely consistent. These structural differences in the Z chromosome may be associated with the speciation of the black and mute swan from their last common ancestor (approximately 6.1 million years ago) [11].

Inversions have been associated with the differential susceptibility of chickens and ducks to avian influenza [12]. We found no substantial inversions between the black and mute swan. However, given that both ducks and swans are *Anseriformes*, we compared structural genomic differences between the susceptible black swan and mute swan and relatively resistant duck, using the ostrich as an outgroup. We investigated genes in the inverted genomic regions present in the duck but absent in the swans on chromosome 1 (Additional file 1: Figure S7). Strikingly, we found that 53 inverted genes (out of 1758 total genes) in chromosome 1 of the duck genome were mapped to immune system processes (Additional file 6: Table S4). However, given the absence of substantial structural variants between the black and mute swan, it is likely that any immunological consequences of these structural variants would be present in multiple swan species.

The black and mute swan genomes were then annotated according to the methods listed in Additional file 1: Figure S4. Sixteen thousand two hundred four (16,204) and 15,789 gene models were obtained through Evidence Modeler as the final gene models in the black swan and the mute swan, respectively. Protein alignment against the UniProt/Swiss-Prot database was used to infer 15,478 gene model names for the black swan and 14,791 gene model names for the mute swan.

Importantly, it is impossible to infer if these differences represent true biological differences in the number of annotated genes or simply reflect the higher quality of the black swan genome.

### Mutations in *SLC45A2* may account for differences in swan plumage

**Fig. 1:**
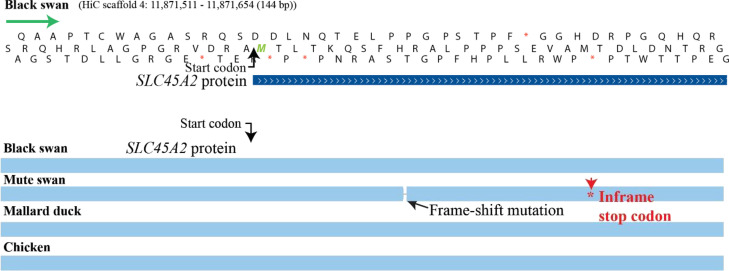
Position of the frame-shift mutation in the mute swan *SLC45A2* compared to the black swan genome. The figure was created with whole-genome HAL alignment produced from CACTUS. The alignment was visualized with the University of California, Santa Cruz (UCSC) Genome Assembly in a Box (GBiB). The predicted mute swan gene *SLC45A2* was aligned using the BLAT alignment algorithm.

One of the most remarkable features of the black swan is its distinct plumage colour. To determine if the highly annotated genomes presented herein could offer insight into the iconic plumage, we examined genes (Additional file 7: Table S5) known to be associated in avian plumage color [13, 14]. We observed that *SLC45A2* in the mute swan had a nucleotide deletion in the open reading frame instigating a frame-shift mutation and an in-frame early stop codon (Fig. 1). Multiple non-homologous nucleotides were detected in the chicken and the duck *SLC45A2* relative to that of the black swan (Fig. 1). *SLC45A2* encodes a membrane-associated transport protein which regulates the tyrosinase activity and the melanin content of melanosomes [15]. The knockdown of this gene causes reduced tyrosinase activity and low melanin content in human melanoma cell lines [16]. These results suggest that this deletion in *SLC45A2* is a candidate genetic change that could be responsible for the white plumage in white swans in the genus *Cygnus*.

### Immune gene families are expanded in the mallard duck and mute swan genomes

**Fig. 2:**
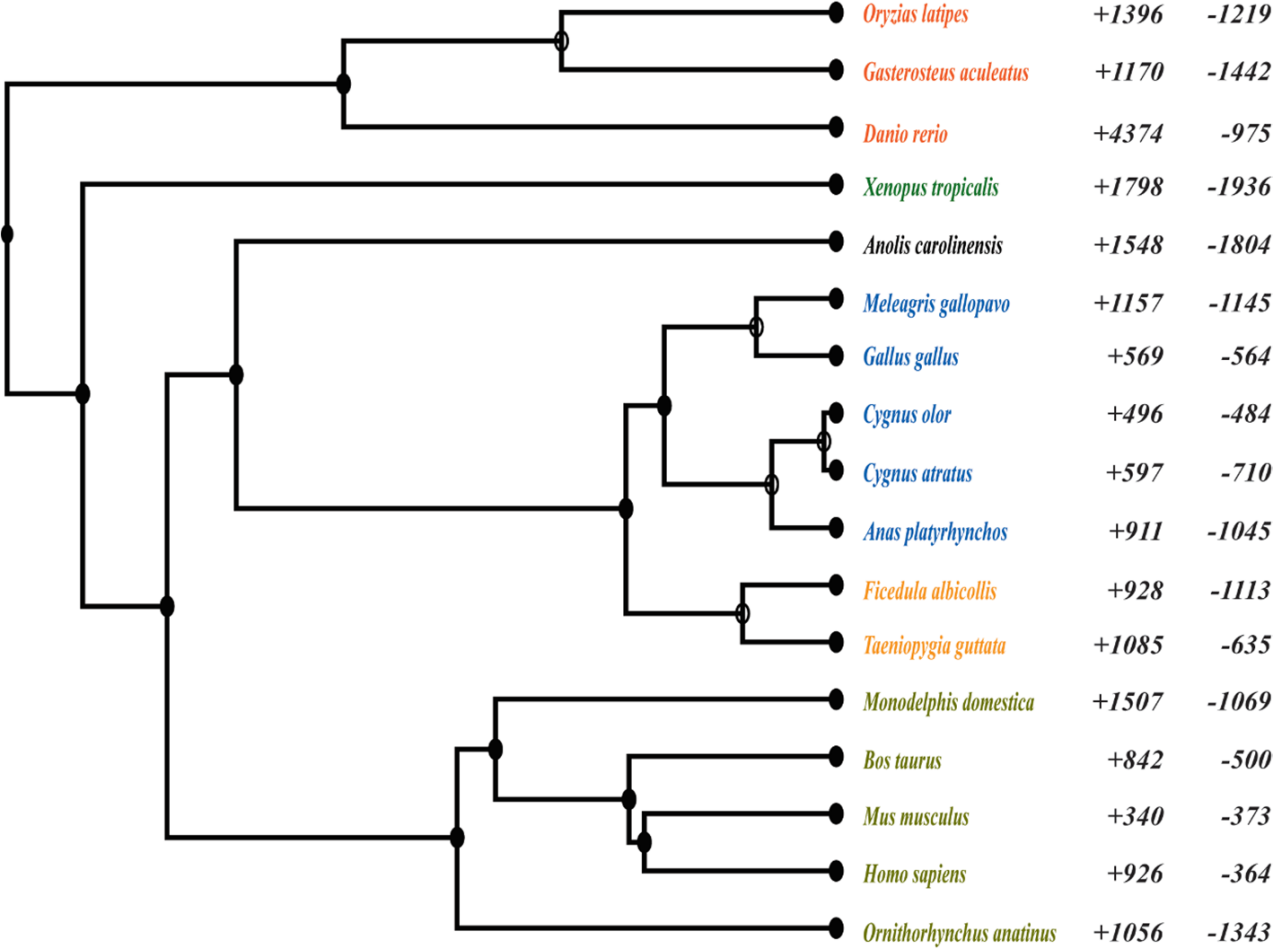
Gene losses and gains across vertebrate species tree. Data are shown for 17 vertebrate species, three teleost (red), an amphibian (green), six reptilians (including birds) (black, blue, and orange), and five mammals (yellow). The numbers of gene family gains (+) and losses (−) are given to the right of the taxa (*p-value* < 0.05) compared to the taxon’s last common ancestor. The rate of gene birth and death for clades derived from the most recent common ancestor (MRCA) for *Gallianseriformes* is 0.0016 (per gene per million years)

To understand whether the black swan (which has been isolated in Australia) has an altered immune gene repertoire compared to its close relatives, evolutionary gene gain and loss were determined (Fig. 2) (*p-value* < 0.05). The black swan genome was estimated to be contractive, indicating that the total gene gain was less than the gene loss from the last common ancestor. The biological function of expanded genes in the black swan, chicken, mute swan, and duck was then investigated.

**Fig. 3:**
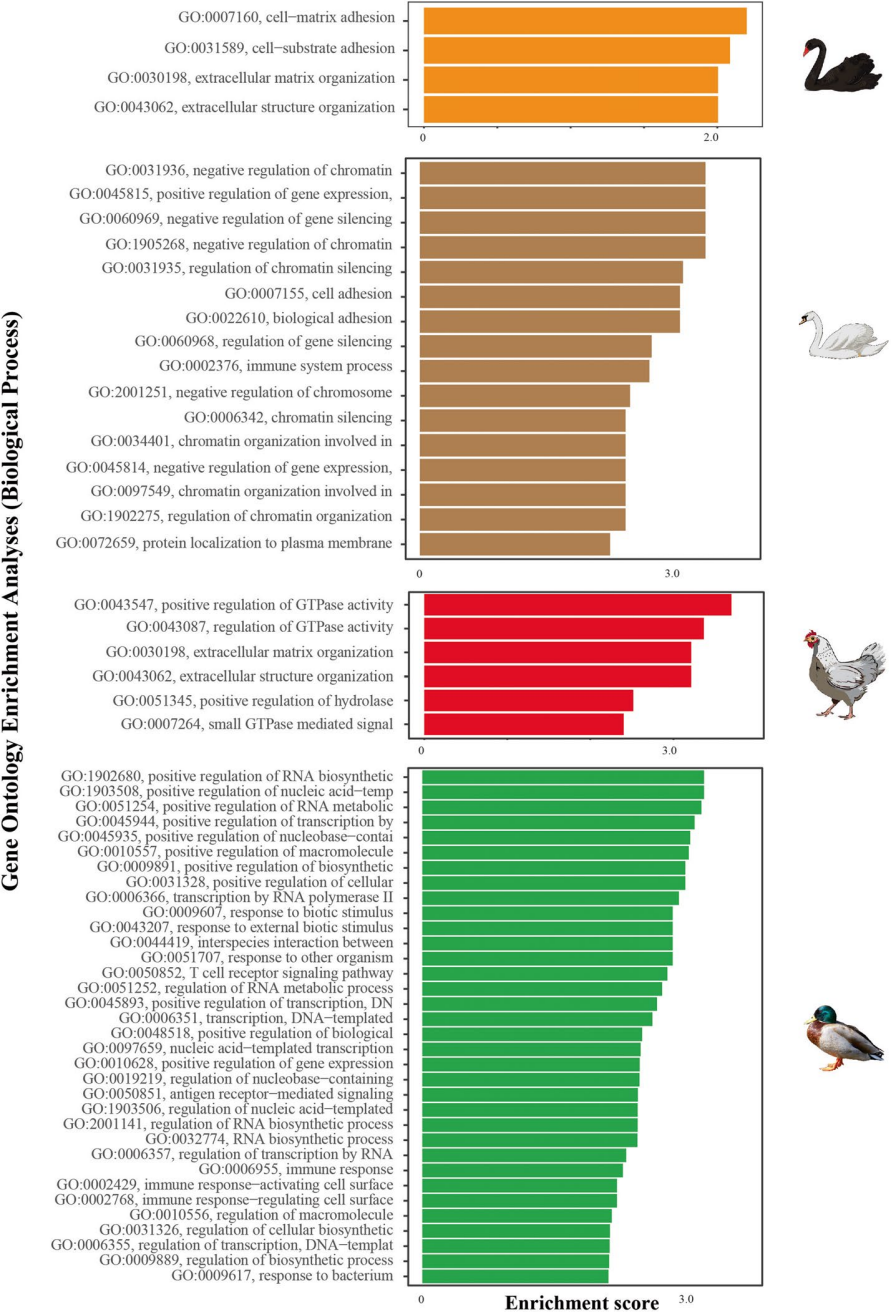
GO enrichment analysis for over-represented GO terms associated with significantly expanded gene families in black swan, mute swan, chicken, and mallard duck

Strikingly, gene families related to immune system processes (e.g., GO0002376) were only expanded in the mallard duck and the mute swan genomes (Fig. 3). In contrast, in chickens, expanded gene families were associated with regulation of GTPase activity, extracellular matrix and structure organization, while the over-represented functional terms for expanded black swan gene families included cell-matrix adhesion, cell-substrate adhesion, extracellular matrix organization and extracellular structure organization. To specifically compare the immune gene repertoire of black and mute swans, we used human and mouse immune genes to identify immune gene families in *Cygnus* species. Thirty-nine immune-related gene families of the black swan were contractive compared to the mute swan (Additional file 8: Table S6). The PANTHER pathways related to these genes included apoptosis signaling, cadherin signaling, general transcription by RNA polymerase, gonadotropin-releasing hormone receptor, inflammation mediated by chemokine and cytokine signaling, interleukin signaling, TGF-beta signaling, and Wnt signaling pathways.

### Black and mute swan classical Major Histocompatability Complex loci are homologous

Major Histocompatability Complex (MHC) diversity is altered in some avian species [17], which may affect susceptibility to disease [18]. We therefore compared MHC loci between black and mute swans. Two MHC class I and MHC class II loci were identified in the black and mute swan (Additional file 1: Figure S8). These were located on chromosome 33 in the swan genomes. A similar number of MHC complex associated genes were identified in each species. None of these genes appeared to be pseudogenes. Compared to mammals, both mute and the black swans have a compact, relatively simple MHC B locus (Additional file 1: Figure S9), with two class IIb (*BLB*) genes followed by a pair of class I (*BF*) genes that flank the *TAP* genes. The *TAPBP* gene in both birds, unlike chickens, does not flank the two-class-IIb genes [19]. Overall, the MHC region of both the black and mute swan share a similar genome landscape and represent a "minimal essential MHC" similar to chicken and mallard duck [20]. It is therefore unlikely that differences in the MHC complex contribute to species-specific differences in the response to HPAI virus infection.

### Toll-like receptor 7 expression cannot be detected in black swan endothelial cells

**Fig. 4:**
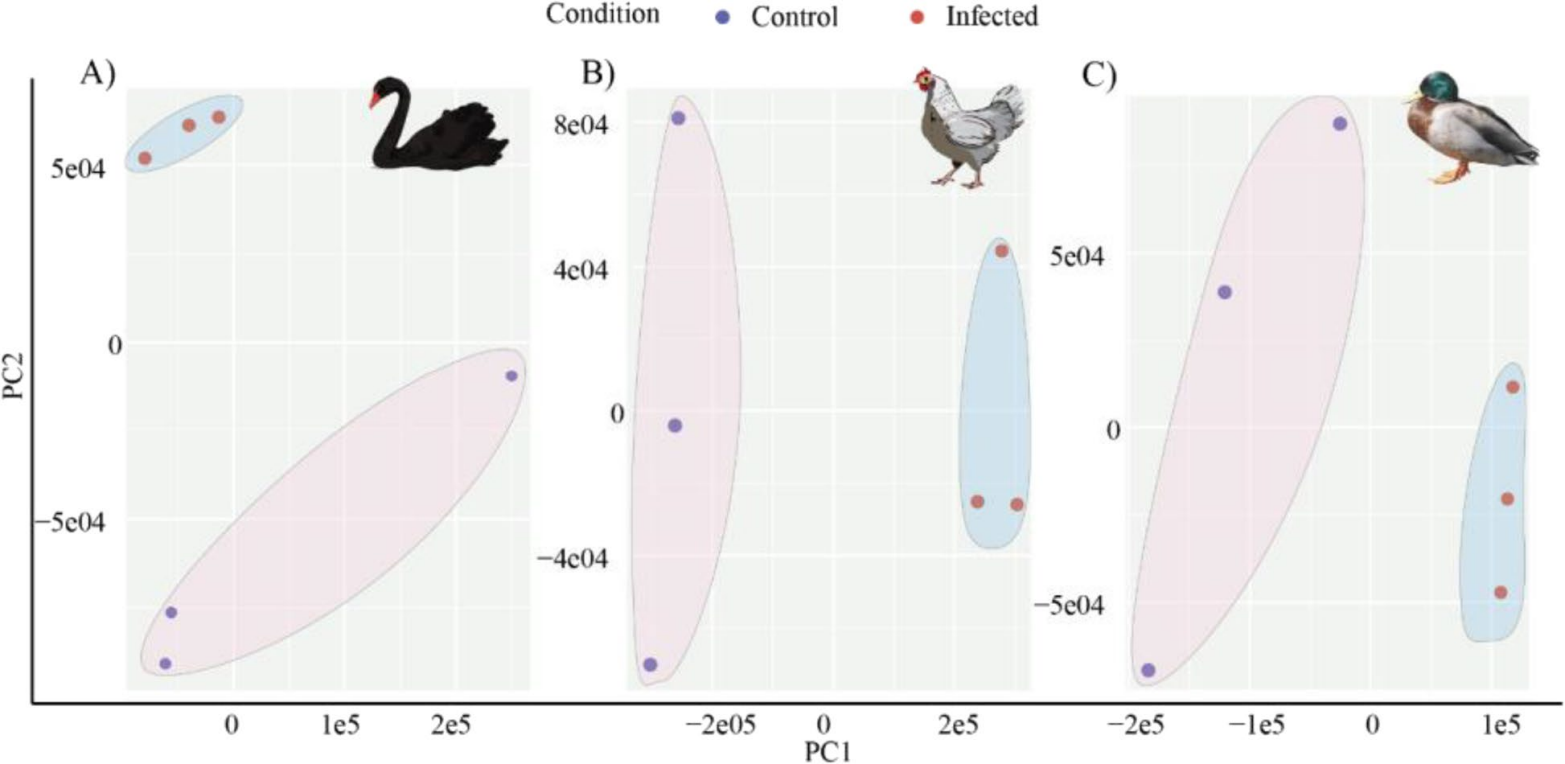
Principal component analysis (PCA) of infected and uninfected **A** black swan, **B** chicken, and **C** duck endothelial cells. The control and the infected groups showed intergroup clustering, indicating differences in whole transcriptome profiles between the control and the infected group in each species

Toll-like receptor 7 (TLR7) signaling has been implicated in influenza A virus recognition in mammals and birds where it functions as a pathogen recognition receptor that recognizes single-stranded viral-RNA [21]. *TLR7* has been duplicated independently in several avian species [22] and differences in *TLR7* tropism and function have been associated with the increased resistance of ducks to HPAI [23]. There was no notable structural difference in the *TLR7* gene between the black and mute swan genomes. However, strikingly, *TLR7* expression signals were detected in ISO-Seq analysis of mute swans but not in the ISO-seq analysis of black swan (Additional file 1: Figure S10). To independently confirm these data, we investigated the expression of *TLR7* using qRT-PCR in black swan tissues collected post-mortem. *TLR7* mRNA could not be detected in any of the collected black swan tissues (Additional file 9: Table S7). As *TLR7* expression can be induced by interferon, we reasoned that gene expression in the black swan may only be detected in the presence of virus infection. Accordingly, we sought to establish an in vitro model of HPAI infection in black swans. In black swans experimentally infected with HPAI virus, endothelial cells are the primary target of infection [3]. We observed similar infection of endothelial cells in swans naturally infected with HPAI (Additional file 1: Figure S11). We therefore cultured primary black swan endothelial cells according to our previously described protocol for avian species [24] and endothelial cell identity was confirmed by tube formation, uptake of acetylated low density lipoprotein, von Willebrand factor expression and the absence of *CD45* expression (Additional file 1: Figure S12). Chicken, duck, and black swan endothelial cells were infected with A/Chicken/Vietnam/008/2004/H5N1 (VN04) and 6 h later gene expression was examined by RNASeq. PCA plots showed that mock and virus-infected samples clustered separately for all three species (Fig. 4). Viral-RNA was detected in the infected endothelial cells of all three species (data not shown). Importantly, while *TLR7* transcription was upregulated (although not statistically significant) in infected duck and chicken endothelial cells, *TLR7* transcription could not be detected in infected or naïve black swan endothelial cells (Table 2). Moreover, while *myeloid differentiation primary response 88 (MyD88)*, the downstream adaptor of *TLR7* was upregulated in infected duck and chicken endothelial cells, it was downregulated in infected black swan endothelial cells (Table 2). These data are consistent with the absence of *TLR7* expression in black swan endothelial cells, despite an apparently intact *TLR7* gene in the genome. No other notable expression differences in ISOSeq/RNASeq transcripts were recorded in immune genes noted in the influenza A KEGG pathway (https://www.kegg.jp/kegg-bin/show_pathway?ko05164).

**Table 2:** Quantified *TLR7* and *MyD88* expression following VN04 infection of avian endothelial cells

Species	Gene	Fold-change	Adjusted ***p*** value	Ensemble gene ID
Duck	*TLR7*	3.13	>0.1 (0.57)	ENSAPLG00000026818
Duck	*MyD88*	1.37	<0.1 (9.87e-8)	ENSAPLG00000026818
Chicken	*TLR7*	2.37	>0.1 (0.23)	ENSGALG00000016590
Chicken	*MyD88*	1.31	<0.1 (1.9e-7)	ENSGALG00000005947
Black swan	*TLR7*	NE	NA	^a^ENSACYG00000003732
Black swan	*MyD88*	-1.22	>0.1 (0.15857)	^a^ENSACYG00000016507

^a^Corresponding gene ID Ensembl annotation for black swan

*NA* not applicable, *NE* not expressed/detected

### Black swan endothelial cells display a dysregulated pro-inflammatory response to HPAI virus infection

**Fig. 5:**
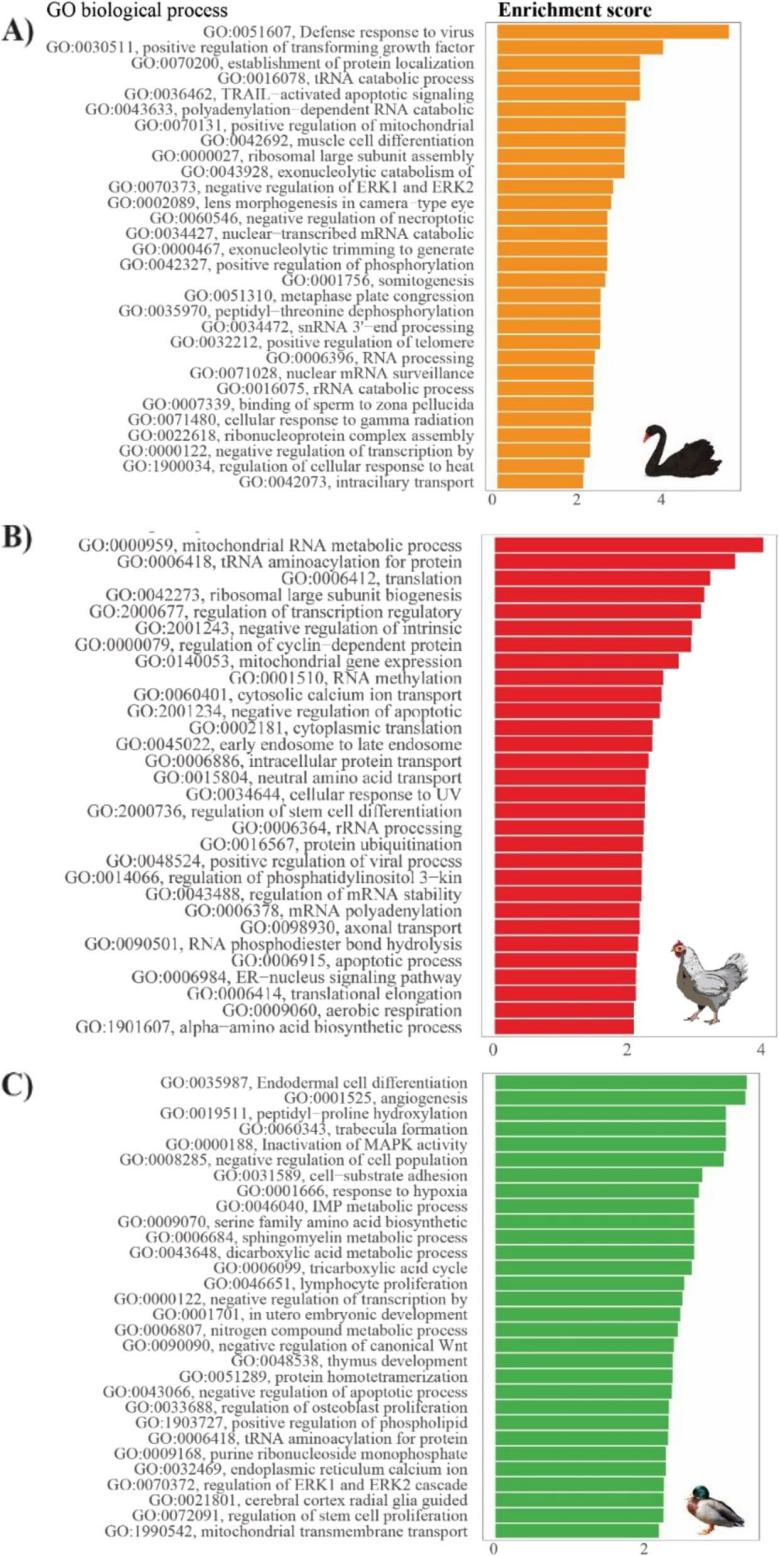
Top 30 GO biological process terms that were significantly enriched in infected **A** black swan, **B** chicken, and **C** duck endothelial cells. The bars represent the enrichment score for the corresponding GO biological term with a *p*-value <0.05

The transcriptomic data generated herein offer a unique insight into the transcriptomic response of black swans to HPAI virus infection. To explore this matter further, we performed Gene Ontology (GO) enrichment analysis using significantly differentially expressed genes in infected chicken, duck, and black swan endothelial cells (Additional file 10: Table S8, Additional file 11: Table S9, Additional file 12: Table S10). The most significantly upregulated gene in black swans was *IFIT5, IL8* in chickens, and *BCOR* in ducks. *IL6* was significantly upregulated in the black swan (log_2_fold change = 1.89) and chicken (log_2_fold change = 1.21), indicating a strong pro-inflammatory response while not differentially expressed in ducks. Black swan, chicken, and duck endothelial cells differentially expressed other cytokines/chemokines and their receptors in response to HPAI VN04 infection (Additional file 13: Table S11). Typically, black swan and chicken endothelial cells upregulated more cytokines and cytokine receptors than duck endothelial cells in response to HPAI VN04 infection. Indeed, the highest number of cytokines and cytokine receptors were upregulated by infected chicken endothelial cells (Additional file 13: Table S11). In infected black swan endothelial cells, 113 GO terms were significantly enriched (Additional file 14: Table S12; Fig. 5A). Many of these GO terms were associated with innate immunity, the cytokine signaling response and chemokine signaling. Several innate immunity pathways were increased in response to viral infection (*z*-score > 0) while GO terms such as negative regulation of Mitogen-Activated Protein Kinase (MAPK) activity and negative regulation of c-JUN N-terminal Kinase (JNK) cascade were decreased (*z*-score < 0). Similarly, 123 enriched GO terms in infected chicken endothelial cells included positive regulation of viral response and regulation of leukocyte chemotaxis (Additional file 15: Table S13; Fig. 5B). Terms such as leukocyte mediated cytotoxicity were increased after infection (*z*-score > 0) while negative regulation of apoptotic signaling and the positive regulation of innate immune responses were decreased. GO biological process terms enriched in infected duck endothelial cells were not primarily associated with the innate immune response (Additional file 16: Table S14; Fig. 5C). Rather, most genes were linked to cellular biological signaling and activity. This finding is consistent with our previous study of HPAI viruses in duck endothelial cells [25]. Interestingly, in direct contrast to black swans, the inactivation of MAPK activity was significantly increased in ducks (*z*-score < 0). Due to the wide-ranging role of the MAPK cascade, including pro-inflammatory responses, we further investigated the expression profiles of the genes and identified ten genes involved in the “inactivation of MAPK pathway,” five of which were significantly downregulated genes (i.e., *DUSP1*, *DUSP4*, *DUSP7*, *DUSP10*, and *RGS3*) in black swans (Additional file 1: Figure S13). Dual-specificity phosphatases (DUSPs) are negative regulators of MAPKs and their associated pro-inflammatory effects [26]. Accordingly, we specifically examined the differential expression of DUSPs across the three avian species. In the black swan, all DUSPs were either not differentially expressed or downregulated in response to infection. In contrast, in the duck, all DUSPS (except for *DUSP15*) were upregulated. Similar, in the chicken, *DUSP1*, *DUSP5*, *DUSP7*, *DUSP10*, *DUSP15*, and *DUSP16* were significantly downregulated in response to infection (Additional file 17: Table S15). In contrast, in the duck, all *DUSPs* (except for *DUSP15*) were upregulated. This transcriptional profile is consistent with poor regulation of a pro-inflammatory response to HPAI virus in black swans.

## Discussion

The black and mute swan reference genomes provided herein represent the first publicly available swan genomes. The analyses of these genomes, together with the first black swan transcriptome in response to HPAI virus infection, has provided a unique insight into the plumage and immune system of the black swan.

The genomic insights provided by the present study were only possible due to the growing availability and accessibility of third generation sequencing. Specifically, older technologies that generate short-read sequences can result in incorrect assembly, annotation errors, and a large amount of manual effort to correct individual genes [7]. In contrast, and consistent with the broader goals of the Vertebrate Genomes Project [7], the use of longer read sequences herein allowed us to generate black swan and mute swan genomes that were scaffolded to near chromosomal length and that were of comparable quality to the well-annotated chicken reference genome.

Genomic analysis of genes known to be associated with plumage color in other birds [27] identified a potential frame-shift mutation in the first exon of *SLC45A2* in the mute swan, which may have led to pseudogenization of this gene. *SLC45A2* encodes a transporter protein involved in melanin synthesis and is considered one of the most important proteins affecting human pigmentation [28]. Mutations in the *SLC45A2* gene have been reported in albinism in humans [29]. Furthermore, mutations in the gene have been associated with plumage color variation in Japanese quails [13], indicating the importance of the *SLC45A2* in avian plumage. Interestingly, should a mutation of *SLC45A2* have resulted in the differential plumage of the black and mute swan, it would suggest that the last common ancestor of these birds was, in fact, black. This is direct contrast to the metaphor of “black swan events” that are so defined because of their unprecedented and unexpected nature. Instead, it would appear that at one point in history black plumage for the swan was the norm rather than the exception.

Compared to the last common ancestor, mute swan and mallard duck gene families involved in immune system processes were expansive. In contrast, no expansion in immune gene families was noted in the chicken or the black swan. This differential immune gene expansion, and its implications for susceptibility to HPAIV, is likely compounded by the observed impaired expression of *TLR7* in the endothelial cells of black swans. Interestingly, other genes that have been observed to be differentially expressed between chickens and ducks, and implicated in susceptibility to HPAIV, were not differentially expressed between infected black swan and duck endothelial cells (e.g., *RIG-I* and *IFITM3*) [30–33]. It is interesting to speculate as to whether mute swan endothelial cells would express *TLR7*. However, the presence or absence of *TLR7* in the endothelial cells of mute swans is perhaps irrelevant to the pathogenesis of HPAIV, as the virus is not heavily endothelial tropic in this species [3]. In the black swan, the observed differences in *TLR7* expression in endothelial cells speak to the value of combining genomics with both primary cell culture and transcriptomics, as has recently been suggested as the new standard for comparative genomics by Stephan and colleagues [34]. Either as a result of, or in addition to, these observed immune differences, black swan endothelial cells also mounted a markedly pro-inflammatory response to HPAIV infection. We have previously reported a similar pro-inflammatory response in infected chicken endothelial cells (compared to those of ducks) and speculated that this inflammatory response leads to immunopathology in chickens in vivo[25]. Whether disease severity in black swans is driven by immunopathology remains to be determined, although it is consistent with the observed pathology in infected birds [2, 3]. In sum, it is likely that this combination of species-specific differences in the immune response contribute to the marked susceptibility of both the black swan and chicken to HPAIVs.

## Conclusions

The observed species-dependent differences in the immune responses of swans raise the intriguing question as to why the black swan continues to thrive in its native Australia as well as in New Zealand (where it was introduced in nineteenth century). This may be due to the fact that HPAIV is not endemic in Australia and New Zealand. Indeed, captive populations of black swans located in parts of the world frequently exposed to HPAIV are highly susceptible to severe disease [4]. The data presented in this study would therefore suggest that should HPAI become more prevalent in the Oceania region, the ongoing survival of the black swan would be at significant risk. Moreover, many of the immune limitations described herein are not specific to avian influenza viruses. For example, TLR7 is essential in the immune recognition of a wide number of viral pathogens including avian coronaviruses [35]. These data suggest that should any avian endothelial specific viral infection become established in the native habitat of the black swan, the survival of this iconic species would be in significant peril.

## Methods

A full description of the methods can be found in Additional file 3: Online Methods. No statistical methods were used to predetermine sample size.

### Black swan genome

#### Sequencing

For genomic DNA and RNA extraction, liver, spleen, kidney, skeletal muscles, lung, heart, stomach, intestines, and gonads were obtained from an adult male black swan that had to be euthanized at Currumbin Wildlife Sanctuary hospital (https://currumbinsanctuary.com.au/wildlife-hospital) due to injury. Genomic DNA from the stomach tissue was extracted using a commercially available kit (MagAttract HMW DNA kit – Qiagen®). A DNA library was prepared using Single Molecular Real-Time (SMRT) bell template Prep Kit 1.0 (Pacific Biosciences) with unsheared gDNA with a size cut-off of 20kb using a Blue Pippin instrument (Sage Science ©) according to the manufacturer’s protocol. SMRT sequencing was performed using PacBio Sequel instrument at the Institute of Molecular Biosciences, the University of Queensland using 1-6 pica Molar (pM) loading concentration, version 3.0 sequencing chemistry, diffusion loading, and 10-h movies over 10 SMRT cells (1M V.3).

#### Genome assembly

Raw SMRT sequencing reads were used for de novo black swan genome assembly using open-source FLACON (v2018. 31-08-03.0)/FALCON-UNZIP (6.0.0.47841) diploid aware genome assembly algorithms to produce primary and alternative haplotigs as the primary assembly. For FALCON assembly, the estimated genome size (haplotype) used for the black swan was 1.4181 × 10^9^ base pairs. Additional parameters used for FALCON assembly are listed in Additional file 18: Table S16. Raw FASTA sequences were extracted from the subread BAM files produced by SMRT sequencing using PacBio® BAM2fasta tool. Raw FASTA sequences from 10 SMRT cells were given as the input for the FALCON assembler, and the FALCON assembly parameters were provided using a configuration file. Once the FALCON run was completed, FALCON-UNZIP was executed in the same directory as the initial FALCON run. For the FALCON-UNZIP run, the subread BAM data was also used. Once the FALCON UNZIP run was complete, two genome polishing steps were undertaken through PacBio® SMRT Link version 7.0.0 resequencing pipeline using PacBio CLR reads with default settings (-x 30) in addition to FALCON_UNZIP integrated arrow polishing to produce the primary PacBio assembly. This PacBio assembly was screened for the presence of mitochondrial/non-chromosomal DNA suing NCBI Blast+ (v2.9.0+) against the black swan mitochondrial genome [36]. The mitochondrial DNA sequences were removed from the primary PacBio assembly. PURGE_DUPS (v1.2.5) was next employed to remove false haplotypic duplications from the concatenated primary PacBio assembly and alternate haplotigs. The PacBio assembly completeness was assessed via BUSCO V.5.1.2 against avian orthoDB10. The presence of highly conserved core vertebrate genes was evaluated using the CEGMA pipeline, available online in gVolante (https://gvolante.riken.jp/).

The FALCON draft was scaffolded to chromosome-length by the DNA Zoo Consortium following the methodology described here: www.dnazoo.org/methods. Briefly, the Hi-C data was processed using Juicer [37] and used as input into the 3D-DNA pipeline [38] to produce a candidate chromosome-length genome assembly. We performed additional finishing on the scaffolds using Juicebox Assembly Tools [37, 38]. The contact matrices generated by aligning the Hi-C data to the genome assembly before and after the Hi-C scaffolding are available for browsing at multiple resolutions on https://www.dnazoo.org/assemblies/Cygnus_atratus visualized using Juicebox.js, a cloud-based visualization system for Hi-C data [39]. Finally, the Hi-C assembly was subjected to gap closing using the TGS-GapCloser (v1.0.1) algorithm [40] with default settings. PacBio continuous long reads (CLRs) were used as input for the software tool for gap closing.

The *Anatidae* family’s established terminology for the chromosome-length scaffolds was based on the homology between black swan scaffolds/chromosomes and the closely related mute swan. MUMMER (v3) was used to establish a 1:1 homology between the black swan and the mute swan before assigning the macro and microchromosomes terminology to black swan chromosomes.

### Mute swan genome

#### Sequencing

The mute swan genome was sequenced by the vertebrate genome project (VGP) according to the “vertebrate genome project assembly phase I” pipeline. The genomic DNA was extracted from the stomach tissue of a male adult mute swan. The sequencing technology and data used to build the mute swan haploid chromosome length hybrid assembly included PacBio Sequel I CLR, Illumina Novaseq®, Arima® Hi-C linkage data and BioNano optical maps.

#### Assembly

FALCON (v2018. 31-08-03.0)/FALCON-UNZIP (6.0.0.47841) was used to generate the phased contigs. To remove the false duplications in the phased haplotigs, Purge_Haplotigs (v1.0.3+) was used. After that, two rounds of scaffolding were performed using 10x Genomics link data with scaff10x (v4.1.0) (https://github.com/wtsi-hpag/Scaff10X) for the first step of scaffolding with 10xGenomics® link data to build the S1 scaffold set. Next, the second step of scaffolding was performed on S1 using BioNano data.

BioNano solve (v3.2.1_04_122018) software was used to produce the S2 scaffold. The last scaffolding was performed to scaffold the S2 set using Arima® Hi-C linkage data with SALSA (v2.2) [41, 42]. The Hi-C contact matrices were generated and visualized as described in the vgp assembly pipeline (https://github.com/VGP/vgp-assembly). Primary and alternate haplotigs were concatenated, and bases were polished with Arrow (SMRTanalysis v6.0.0.47841) using PacBio CLR reads. Two more rounds of polishing were performed with linked reads by aligning with Longranger (v2.2.2) and variant calling with FreeBayes (v1.3.1) followed by consensus calling with bcftools consensus. Finally, manual curation of the assembly was performed using gEVAL (v2019-09-26).

The chromosome assignment was performed using evidence from Hi-C. A scaffold was considered a chromosome with Hi-C evidence when there is a clear unbroken diagonal in the Hi-C maps in the Juicebox. Every Hi-C box from the largest to the smallest for evidence-validated scaffolds was considered as a chromosome. Subsequently, established terminology for the chromosomes was given for the *Anatidae* family.

#### Genome annotation

The final assembly’s structural and functional annotation of genes was conducted using de novo homology-based and evidence-based methods (for details, see Additional file 3: Online Methods). Firstly, the primary PacBio assembly was annotated using NCBI eukaryotic and Ensembl gene annotation pipelines. A set of transcriptomic data (of both short-reads and long reads) derived from the liver, spleen, peripheral blood mononuclear cells and primary bone marrow-derived endothelial cells was used for black swan genome annotation, and the ISOseq data for mute swan annotation was derived from the peripheral blood.

#### Structural variant assessment of the genes associated with plumage color in swans

Structural variants in genes associated with plumage color were examined using the University of California, Santa Cruz (UCSC) genome browser with multiple whole-genome alignments. We first aligned four Gallianseriformes’ whole genomes (black swan, mute swan, chicken and mallard duck) with CACTUS to produce a HAL alignment file [43]. The HAL file alignment was then visualized in the Genome assembly In a Box (GBiB) using the HAL tools package (V2.1). We then used predicted protein and mRNA sequences plumage color genes (from all four species) as queries to perform protein coding region alignment with the BLAT built in the GBiB and compare with each species in the HAL alignment. We validated putative open reading frames (ORF) for each gene from each species by visual inspection. Validity was assessed based on the presence or absence of putative donor-recipient splice sites, start codon, and in-frame stop codons. We also calculated the percentage identify and coverage for each gene between mute and black swans using Blast pairwise alignment.

#### Gene family evolution

The longest peptide sequence for a given protein-coding gene for 17 species was retrieved from Ensembl (release 104) and NCBI Annotation release 103, including zebrafish (*Danio rerio*) (GCA_000002035.4), Japanese rice fish (*Oryzias latipes*) (GCF_002234675.1), three-pinned stickleback fish (*Gasterosteus aculeatus*) (BROAD S1), tropical clawed frog (*Xenopus tropicalis*) (GCA_000004195.3), platypus (*Ornithorhynchus anatinus*) (GCA_004115215.2), gray short-tailed opossum (*Monodelphis domestic*) (GCA_000002295.1), mouse (*Mus musculus*) (GCA_000001635.9), human (*Homo sapiens*) (GCA_000001405.28), cattle (*Bos taurus*) (GCA_002263795.2), anole lizard (*Anolis carolinensis*) (GCA_000090745.2), mallard duck (*Anas platyrhynchos*) (GCA_002743455.1), turkey (*Meleagris gallopavo*) (GCA_000146605.4), chicken (*Gallus gallus*) (GCA_000002315.5), zebra finch (*Taeniopygia guttata*) (GCA_003957565.2), and collared flycatcher (*Ficedula albicollis*) (GCA_000247815.2). The longest amino acid sequences from coding genes in the final reference annotations were used for the black and mute swan. The phylogenetic tree for the above 17 species was constructed using the Orthofinder pipeline with – M msa -S blast -I 1.3 settings. The final rooted species tree was inferred using STAG from the Orthofinder, which was then made ultra-metric with the root-age of 435 million years (obtained from TimeTree.org database). Peptide sequences in 17 species were scored using PANTHER (v15.0) database and clustered into PANTHER families and subfamilies and then identified by a family-specific PANTHER identifier. The maximum likelihood of gene family evolution for a given taxon was estimated by running CAFÉ(v5) with the PANTHER assigned protein families of the 17 species and the Orthofinder-based ultrametric species tree (significant at *p-value* < 0.05).

#### Functional profiling of expanded and contracted gene families in swans

Each PANTHER sub-family identification with a significant gene family evolution was assigned to the gene ontology category (GOslim identification number) to examine the biological role of significantly evolving (either by expansion or contraction) (Vertibri *p-value* < 0.05) gene families through the r-package PANTHER.db. The significant gene families that appeared to have expanded or contracted were separated. Subsequently, GO enrichment analyses were conducted using topGO [44] to test for over-represented functional terms associated with expanded and contracted gene families (a given gene family is considered expanded if the number of genes are increased compared to last common ancestor). The significant gene sub-families annotated with GOslim identifications were used as the foreground, and the GOslim annotated gene subfamilies of the last common ancestor with at least one gene present in the family were used as the background. Fisher’s exact test was used for statistical testing, and the terms with corrected *p-value* < 0.01 were chosen as overrepresented GO terms.

#### Comparison of the immune gene repertoire

Selected immune databases were used to obtain the list of known immune genes from Ensembl release and were compared to that of the black swan and mute swan genome (see Additional file 3: Online Methods for details)

#### Major histocompatibility complex (MHC) class annotation in black and mute swan genomes

The major histocompatibility complex (MHC) loci (MHC class I and MHC class IIb) for both mute and the black swan genomes were identified as previously described [45]. Briefly, the avian order-level consensus sequences for MHC I and MHC II loci were built using several different bird species covering at least three exons (exon 2, exon 3, and exon 4). The blast algorithm aligned the order-level consensus sequences to the black swan and mute swan genomes. MHC loci were estimated as the number of blast hits that contained all three exons within 2kb of each other. Each exon was examined for in-frame stop codons, and it was eliminated as a locus if present. Genes present in regions 100kb upstream and downstream of each locus were manually annotated and the gene location plotted through genes to identify the additional genes of each predicted locus. Each predicted MHC locus was manually inspected for premature stop codons, and if present, was eliminated. Identified MHC molecules and MHC B loci associated genes were visualized for black and the mute swan for comparison using Circlize (R-package).

#### *TLR7* expression in black swan tissues

Total RNA was extracted from tissues of an adult male black swan (stomach, kidney, and liver) using an RNA plus mini kit (Qiagen, Hilden, Germany) according to the manufacturer’s instructions. Genomic DNA was extracted from the stomach muscles using the MagAttract HMW DNA kit (Qiagen®, Hilden, Germany) for the positive control, following the manufacturer’s recommendation. The black swan-specific *TLR7 (FW: TTGCACTTCCACACTCCAAG; RVTCAGTCCAATTGCACCTCTG; Probe:CTCCGAAACAATCGCATTCAACGG)* and *18S (FW: CCTGCGGCTTAATTTGACTC; RV: AGACAAATCGCTCCACCAAC; Probe:TTGAGAGCTCTTTCTCGATTCCGTGG)* primers and probes were designed using the primer3plus web tool

#### Immunohistochemistry

Immunohistochemistry for influenza A virus nucleoprotein (NP) was performed according to Additional file 3: Online Methods.

#### Isolation of primary black swan, chicken, and duck endothelial cells

We used previously isolated and characterized chicken and duck primary aortic endothelial cells as the controls for black swan primary endothelial cell characterization [24, 25]. Briefly, 17-day-old chicken and 21-day-old Pekin duck embryonated eggs were purchased from Darling Downs Hatchery (Queensland, Australia). Primary aortic endothelial cells were cultured from the aortic arches of chicken and duck embryos as described previously using EGM-2MV medium (Lonza, Basel, Switzerland) with 10% FBS (Gibco, Waltham, MA, USA) [305, 307]. All avian cells were cultured at 40 °C 5% CO2 unless otherwise stated.

The black swans used for cell isolation were sourced from Currumbin Wildlife Animal Hospital (−28.14, 153.48), Queensland, Australia, and Tauranga Harbor, Bay of Plenty, New Zealand (−37.57, 175.97). The animals were either euthanized due to a terminal illness or as part of a government-approved cull. Tibiotarsi and femurs were obtained from all birds. The bones were then partially opened in the middle to expose the bone marrow and then submerged in pre-chilled (4°C) Dulbecco’s modified Eagle medium (DMEM) in T175 flasks. Flasks were then transferred to a personal containment level 2 (PC2) laboratory for further processing within 4 to 3 h post-mortem.

Bone marrow was subsequently removed and resuspended in pre-chilled DMEM. A 15-ml suspension of DMEM containing bone marrow was then layered after filtering through a 40-μm sterile filter over 15ml of Lymphoprep® in a 50-ml SepMate® tube. The layer of cells at the DMEM-Lymphoprep interface was separated according to the SepMate manufacturer’s instructions. The separated cell layer was then resuspended in 1ml EGM-2MV media and enumerated. The cell concentration was adjusted to approximately 1 × 10^6^ cells per ml before transferring to a Petri dish with 10ml EGM-2MV media. The Petri dish was incubated at 40°C with 5% CO2. The media was replaced every other day until cells became confluent. Bone marrow-derived primary cells (EPC) of passage 4 were cultured to confluency and incubated with 1.75μg/ml Alexa Fluor™ 488 Acetylated low-density lipoprotein (ac-LDL) (Invitrogen, USA) for 4 h. The cells were detached by washing twice with PBS and incubating with 0.05% Trypsin-EDTA (Thermo Fisher®, USA). The cells were mixed in 2% (v/v) FBS in PBS and subsequently sorted with the Moflo Astrios ® High-Speed cell sorter (Beckman Coulter®, CA, USA) under sterile conditions. Ac-LDL-stained cells were sorted based on prior knowledge of high and low ac-LDL intake [303] using BD FACSDiva™ (version 8.0.1 – BD©). The sorted cells were counted and transferred to gelatine-coated plates and grown in EGM-2MV (Endothelial Basal Media with EGM – 2MV bullet kit (Lonza©). Cells at post-sorted passages were known as black swan primary endothelial cells (bsEPCs). The purity of bsEPCs was confirmed as described by Additional file 3: Online Methods.

#### Modeling HPAI in avian endothelial cells

The experiment was designed to include two groups viz. challenge and control, from each of the species, i.e., black swan, chicken and duck. Each group consisted of three biological replicates of endothelial cells.

#### Viral infection

A/Chicken/Vietnam/0008/2004(H5N1) (VN04) virus was amplified in MDCK cells. All experiments using HPAIVs were performed under physical containment level 3 (PC3) settings at the Australian Centre for Disease Preparedness (Geelong, Australia). Primary endothelial cells were grown to at least 60% confluency and were inoculated with 5 × 10^5^ PFU/mL of VN04 for 60 min at 40°C under physical containment 3 (PC3) settings. The inoculum was removed, and the cells were washed once with PBS, and EGM-2MV media without FBS was added. The cells were incubated for an additional 5 h.

#### RNA extraction

According to the manufacturer’s instructions, cellular RNA was extracted from cultured cells using the RNeasy Plus Kit (Qiagen, Hilden, Germany). Subsequently, 3M of sodium acetate (NaOAc, pH 5.5) and 100% ethanol were added for RNA precipitation.

#### RNA sequencing

Paired-end, 150bp long, RNAseq was performed by Macrogen (Macrogen, Seoul, South Korea) approximately 40 million reads per sample. Library preparation was performed using the SMARTer® ultra-low kit (Takara Bio® USA, Inc.). All the libraries were barcoded and subsequently sequenced using the Novaseq® 6000 (Illumina, San Diego, CA, USA) with Novaseq® 6000 S4 Reagent Kit (Illumina, San Diego, CA, USA) as per the manufacturer’s instructions using within a single lane in a single cell to avoid potential technical variations. RNAseq reads were separated into original samples based on the corresponding bar code.

#### Differential gene expression analysis

RNAseq-based differential gene expression analysis was performed after assessing the quality of the raw reads using the FastQC tool before and after adaptor and quality trimming with TrimGalore version 0.6.2 (RRID: SCR_011847). RNAseq reads were then quantified at the transcript level using Salmon v1.5.2. First, the indexes were created for the black swan reference transcriptome and the Ensembl version 104 chicken (GRCg6a) and duck (CAU_Duck1.0) transcriptomes with following parameters (salmon quant -i (params.ind) -l (params.libtype) -g (params.genemap) -r input_r1 -f input_r2 --validateMappings -o (output.dir)). The transcript abundance data of each species were imported through the R package “tximport” by transforming data from transcripts to gene-level quantification. The expression data from each species (black swan, chicken, and duck) were independently analyzed for differential gene expression. Once RNAseq read libraries were normalized for sequencing depth and RNA composition, differential gene expression analysis was performed using DESeq2 version 1.30.1 [46]. A Bonferroni adjusted *p-value* (*adj.p.val*) cut-off of 0.05 and log2fold enrichment of 0.58 (fold-change 1.5) were used as the significance threshold (differential gene expression: mock vs. infected).

#### Gene Ontology enrichment analysis

Gene Ontology (GO) terms were assigned to all quantified genes in chicken and duck using the BioConductor package BioMart [47], and the GO terms were assigned to all expressed and quantified genes using InterProScan5 [48]. For black swan genes, we used InterProScan 5 [48] for GO annotation. Using the GO annotation, we then performed GO enrichment analysis with topGO [44]. For the statistical enrichment, Fisher’s exact test was used with the “weight01” algorithm. GO enrichment analyses were performed for all three GO categories, including “biological process,” “cellular component,” and “molecular function.” Results of the GO enrichment were visualized with “ggplot2” and “GOplot” [49] R packages. We calculated a score (named as “*z*-score” in the GOplot package) to evaluate the trend (increasing or decreasing biological process) using GOplot in-built mathematical formula (Formula 1) for enriched terms of the GO biological process category in each species.

### Availability of data and materials

The codes and data used for the bioinformatic analysis are detailed in Tables 3 and 4, respectively.

**Table 3:** The script usage and availability

Usage	DOI	Ref
Genome assembly Black swan	10.5281/zenodo.7272708	[50]
Genome assembly mute swan (forked repository from original)	10.5281/zenodo.7272713	[7]
MHC class annotation	10.5281/zenodo.7272715	[51]
Genome annotation	10.5281/zenodo.7272717	[52]
RNAseq data analysis	10.5281/zenodo.7272720	[53]
Gene family evolution	10.5281/zenodo.7272722	[54]
Comparison of immune gene repertoire	10.5281/zenodo.7272722	[54]

**Table 4:** Data availability

Title/description	Species	Ref
Swan assembly—primary	Black swan	[55]
Swan assembly—Final	Black swan	[56]
Swan assembly—Final	Mute swan	[57]
Swan ISOseq	Black swan	[58]
Swan ISOseq	Mute swan	[59]
PacBio raw reads	Black swan	[60]
PacBio raw reads	Mute swan	[61]
RNA-seq data—Black swan endothelial cells	Black swan	[62]
RNA-seq data—Black swan endothelial cells	Black swan	[63]
RNA seq data—Black swan endothelial cells	Black swan	[63]
Scaffolding	Black swan	[64]
Short-read DNA sequences—Black swan	Black swan	[65]
Scaffolding	Mute swan	[61]

## Supplementary Information

Additional file 1: Supplementary Figure S1. Schematic overview of the process used to construct the black swan genome. Supplementary Figure S2. Schematic overview of the assembly pipeline version 1.5 of the vertebrate genome project used to construct the mute swan genome. Supplementary Figure S3. Hi-C contact maps for the black swan (a) and mute swan (b). Supplementary Figure S4. Schematic overview of the gene annotation pipeline. Supplementary Figure S5. The largest 34 chromosomes aligned between the black swan (left) and the mute swan (right). Supplementary Figure S6. Collinearity analysis of the Z chromosome between the mute swan (left) and black swan (right). Supplementary Figure S7. Black swan and duck synteny plot between the first chromosome. Supplementary Figure S8. MHC Class I (A) and MHC Class II (B) aligned regions of order-level consensus exons of the mute and black swan. Supplementary Figure S9. Relative locations of MHC complex and associated genes in black and mute swan chromosome 33. Supplementary Figure S10. TLR7 expression can be detected in ISO Seq analysis of the mute swan (A) but not the black swan (B). Supplementary Figure S11. IAV NP antigen distribution in tissues from a black swan naturally infected with A/Black swan/Akita/2/2016 (H5N6) in 2016 in Akita prefecture, Japan. Supplementary Figure S12. The successful culture of primary black swan endothelial cells confirmed by qRT-PCR, immunofluorescence and tube formation. Supplementary Figure S13. Differential regulation of DEGs linked to the “inactivation of MAPK” pathway in black swan endothelial cells in response to VN04 infection along with randomly selected GO terms.

Additional file 2: Supplementary Table S1. Heterozygosity, QV and completeness values for swan genomes.

Additional file 3: Online Methods.

Additional file 4: Supplementary Table S2. BUSCO analysis of the final genomes.

Additional file 5: Supplementary Table S3. CEGMA analysis of the final genomes.

Additional file 6: Supplementary Table S4. GO biological process annotation of inverted genes in the Mallard duck (relative to the black swan).

Additional file 7: Supplementary Table S5. List of genes known to affect plumage colour in birds.

Additional file 8: Supplementary Table S6. Immune gene families are contractive in black swans compared to mute swans. The number of genes in each immune gene sub-family identified is given in each column for the corresponding species.

Additional file 9: Supplementary Table S7. TLR7 expression could not be detected in black swan spleen, liver and kidney by qRT-PCR. *ND: Not detected.

Additional file 10: Supplementary Table S8. Differentially expressed genes in infected black swan endothelial cells.

Additional file 11: Supplementary Table S9. Differentially expressed genes in infected chicken endothelial cells.

Additional file 12: Supplementary Table S10. Differentially expressed genes in infected duck endothelial cells.

Additional file 13: Supplementary Table S11. Differentially expressed cytokine or cytokine related genes in infected avian endothelial cells.

Additional file 14: Supplementary Table S12. 113 GO terms were significantly enriched in infected black swan endothelial cells.

Additional file 15: Supplementary Table S13. 123 GO terms were significantly enriched in infected chicken endothelial cells.

Additional file 16: Supplementary Table S14. 150 GO terms were significantly enriched in infected duck endothelial cells.

Additional file 17: Supplementary Table S15. Dual-specificity phosphatases in black swan, chicken and duck endothelial cells infected with VN04*.

Additional file 18: Supplementary Table S16. Parameters used for the FALCON run. Length cut-off was detected automatically for seed-read length.

Additional file 19: Review history.
