# Supplementary material for: The swan genome and transcriptome, it is not all black and white
Source: Genome Biol. 2023 Jan 23;24:13. doi: 10.1186/s13059-022-02838-0 (PMC9867998; doi:10.1186/s13059-022-02838-0)
Supplement: Supplementary file 1 — Additional file 1: Supplementary Figure S1. Schematic overview of the process used to construct the black swan genome. Supplementary Figure S2. Schematic overview of the assembly pipeline version 1.5 of the vertebrate genome project used to construct the mute swan genome. Supplementary Figure S3. Hi-C contact maps for the black swan (a) and mute swan (b). Supplementary Figure S4. Schematic overview of the gene annotation pipeline. Supplementary Figure S5. The largest 34 chromosomes aligned between the black swan (left) and the mute swan (right). Supplementary Figure S6. Collinearity analysis of the Z chromosome between the mute swan (left) and black swan (right). Supplementary Figure S7. Black swan and duck synteny plot between the first chromosome. Supplementary Figure S8. MHC Class I (A) and MHC Class II (B) aligned regions of order-level consensus exons of the mute and black swan. Supplementary Figure S9. Relative locations of MHC complex and associated genes in black and mute swan chromosome 33. Supplementary Figure S10. TLR7 expression can be detected in ISO Seq analysis of the mute swan (A) but not the black swan (B). Supplementary Figure S11. IAV NP antigen distribution in tissues from a black swan naturally infected with A/Black swan/Akita/2/2016 (H5N6) in 2016 in Akita prefecture, Japan. Supplementary Figure S12. The successful culture of primary black swan endothelial cells confirmed by qRT-PCR, immunofluorescence and tube formation. Supplementary Figure S13. Differential regulation of DEGs linked to the “inactivation of MAPK” pathway in black swan endothelial cells in response to VN04 infection along with randomly selected GO terms. [file 13059_2022_2838_MOESM1_ESM.docx]

**
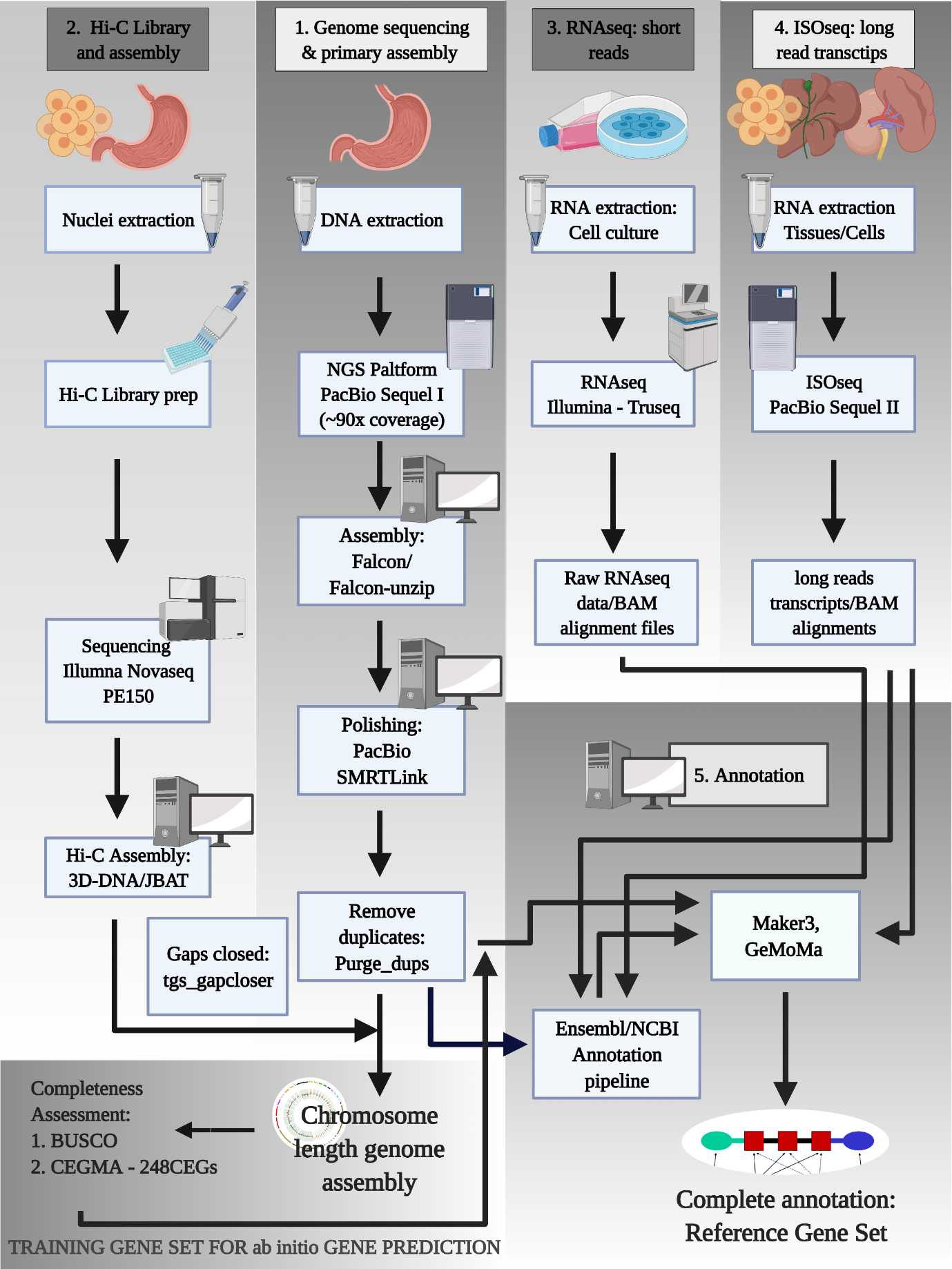
**

**Supplementary Figure S1:** Schematic overview of the process used to construct the black swan genome.


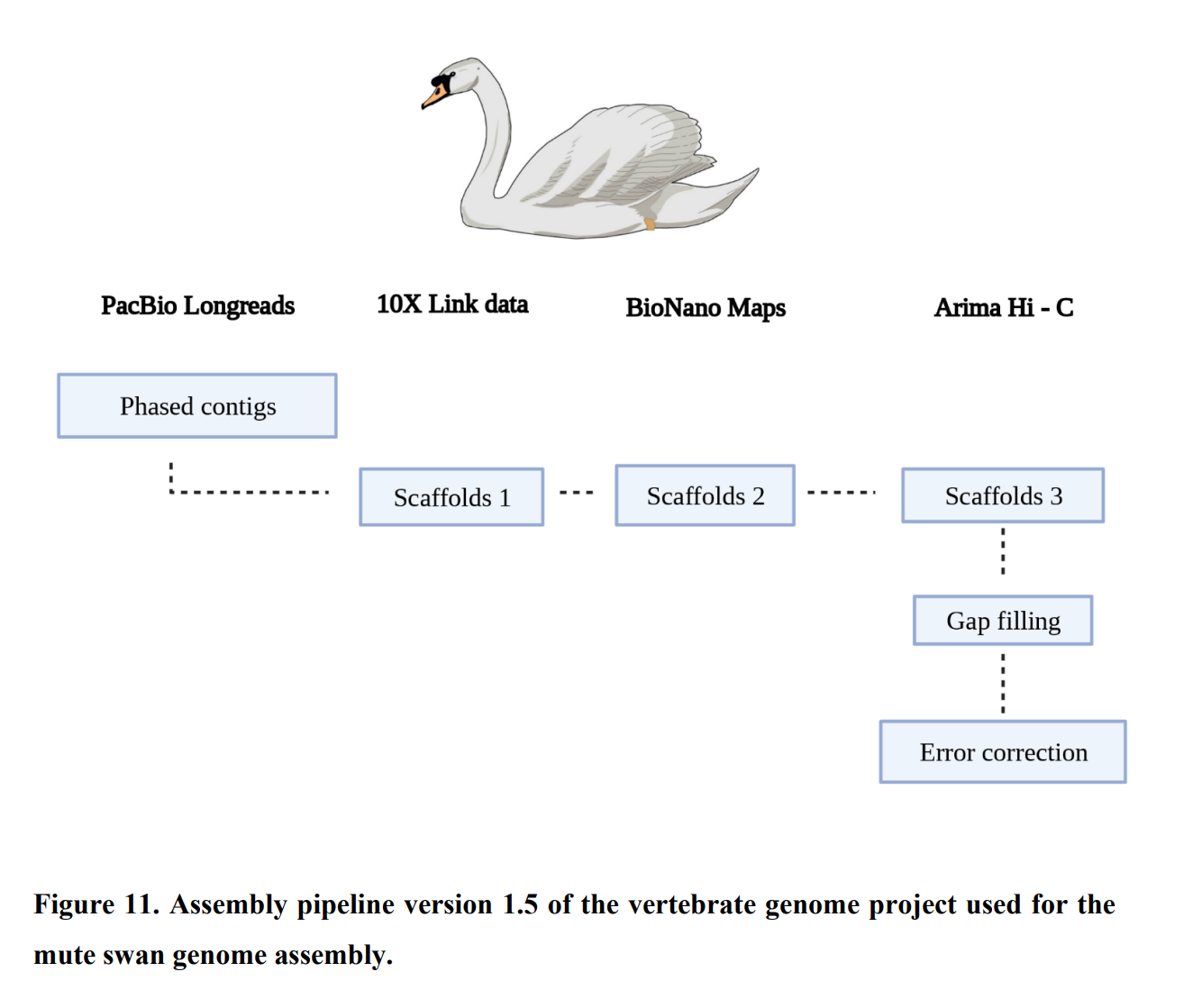


**Supplementary Figure S2:** Schematic overview of the assembly pipeline version 1.5 of the vertebrate genome project used to construct the mute swan genome.

a)


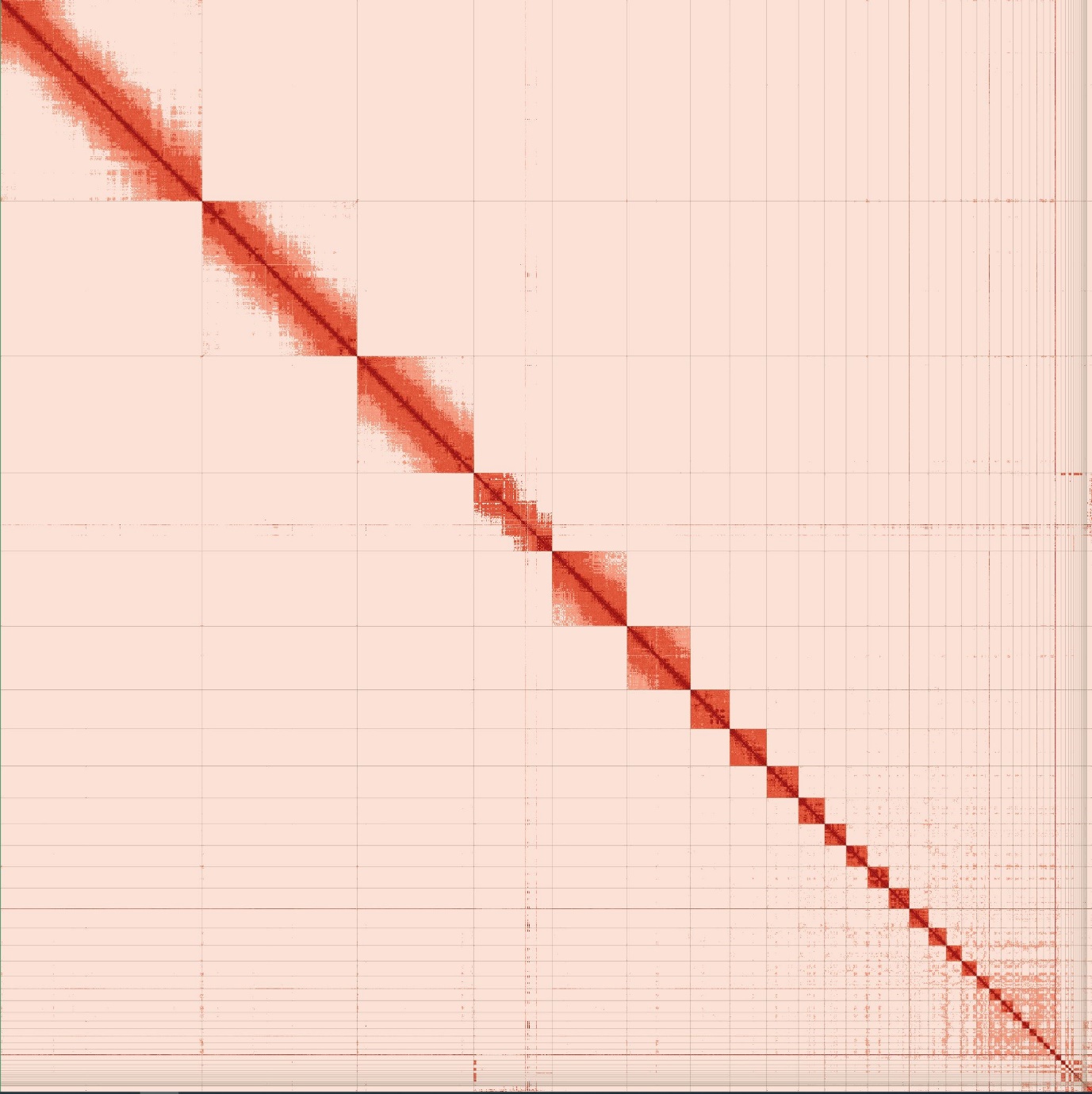


b

**Supplementary Figure S3: Hi-C contact maps for the black swan (a) and mute swan (b)**


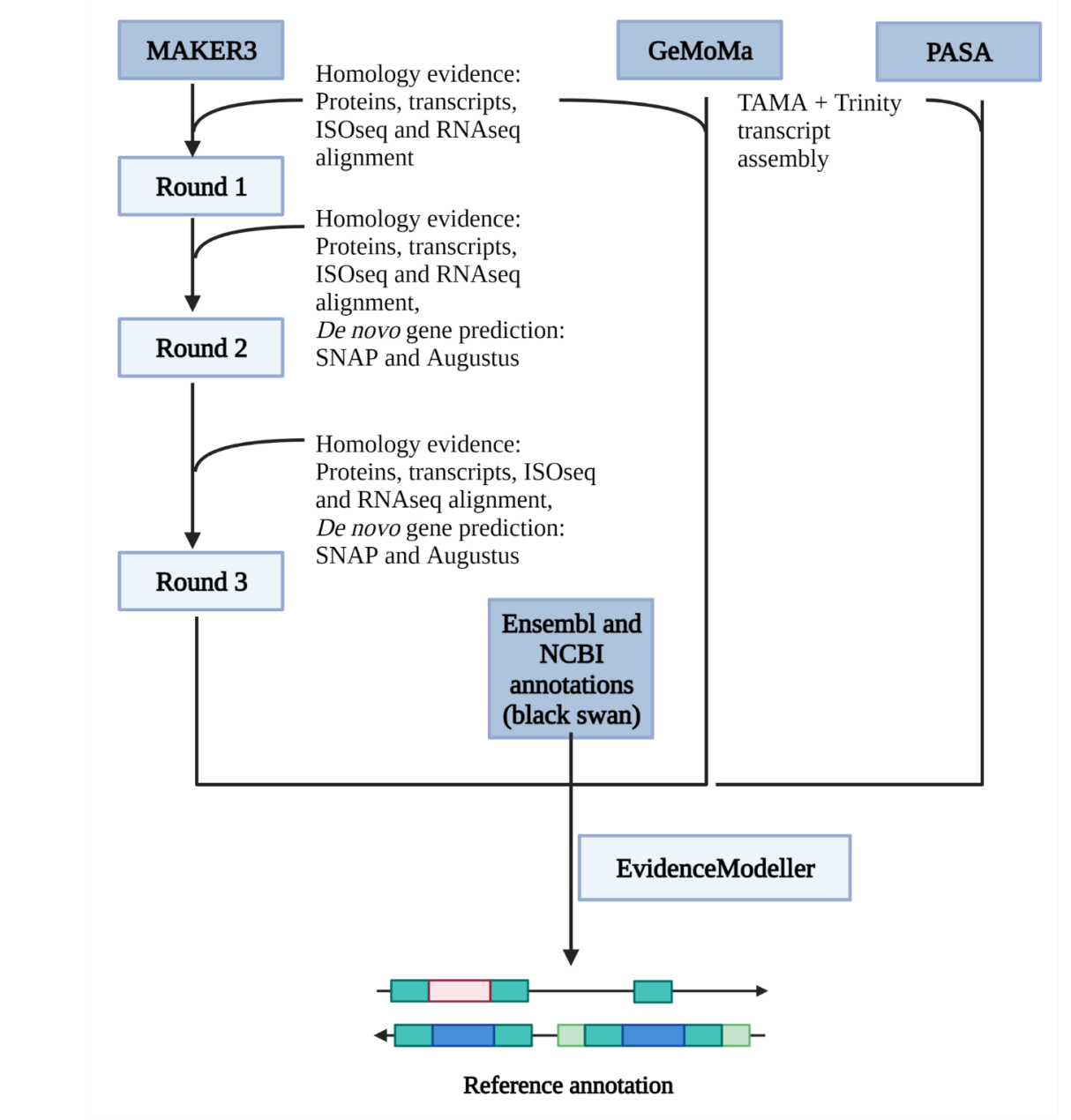


**Supplementary Figure S4: Schematic overview of the gene annotation pipeline.**


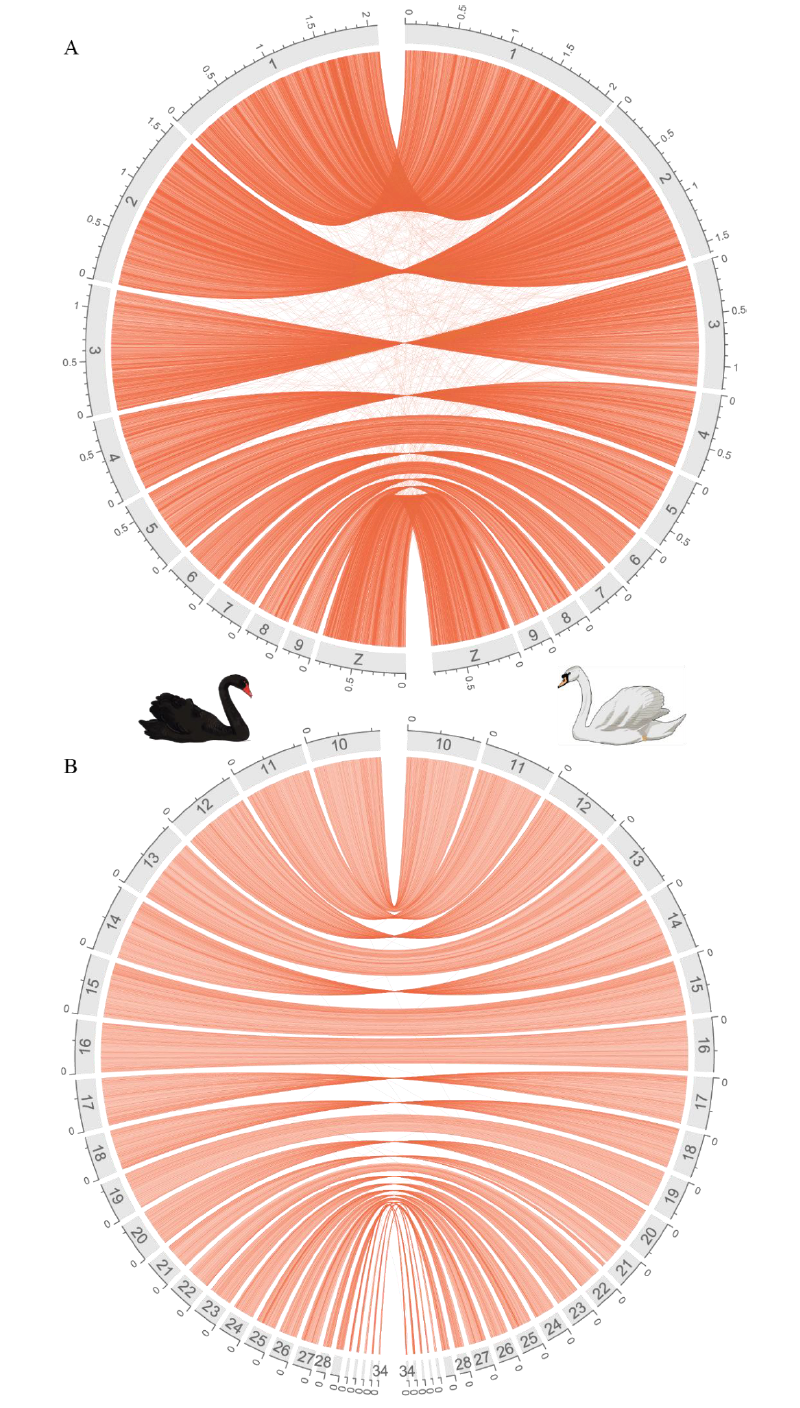


**Supplementary Figure S5: The largest 34 chromosomes aligned between the black swan (left) and the mute swan (right). A)** Macrochromosomes **B)** Microchromosomes. There was a 1-to-1 alignment identity of 98.35% between the two species. Figures were created using MUMMER and Circlize.


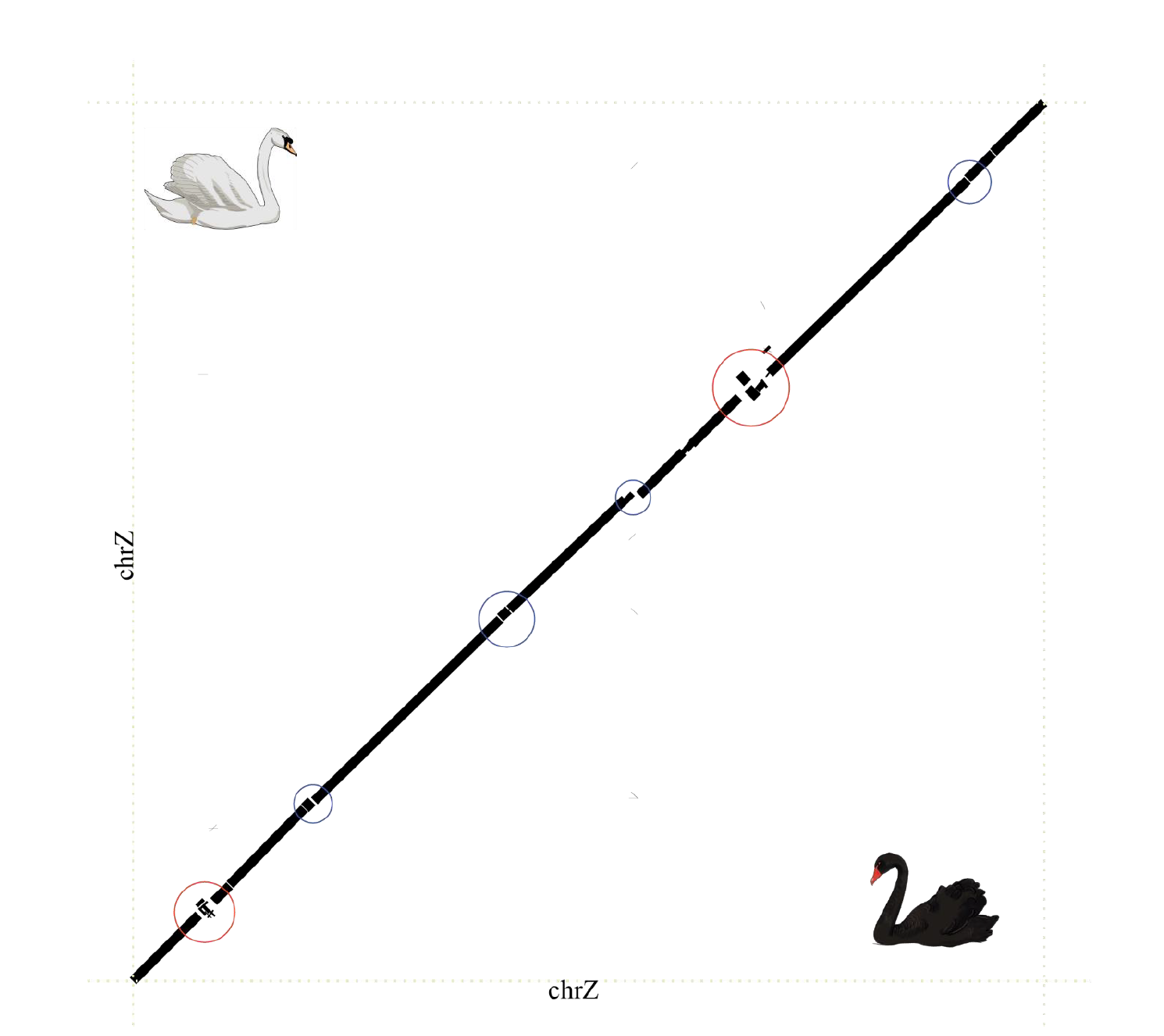


**Supplementary Figure S6: Collinearity analysis of the Z chromosome between the mute swan (left) and black swan (right).** Genomic inversions are circled in red and indels are circled in blue.

**Supplementary Figure S7: Black swan and duck synteny plot between the first chromosome**


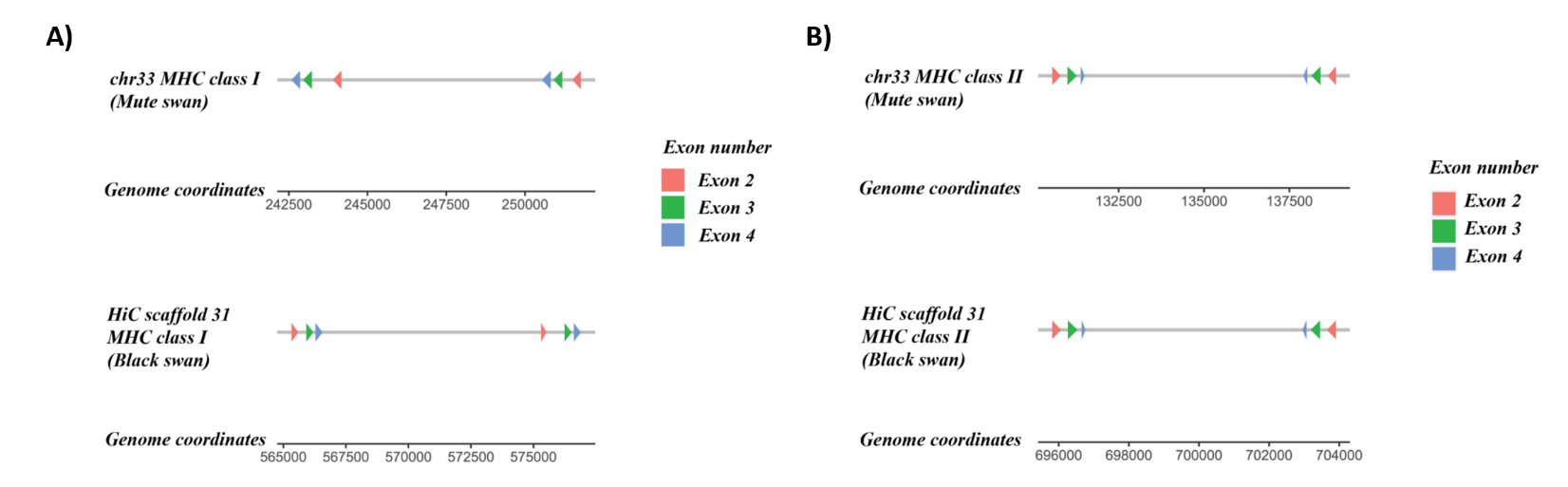


**Supplementary Figure S8: MHC Class I (A) and MHC Class II (B) aligned regions of order-level consensus exons of the mute and black swan.**


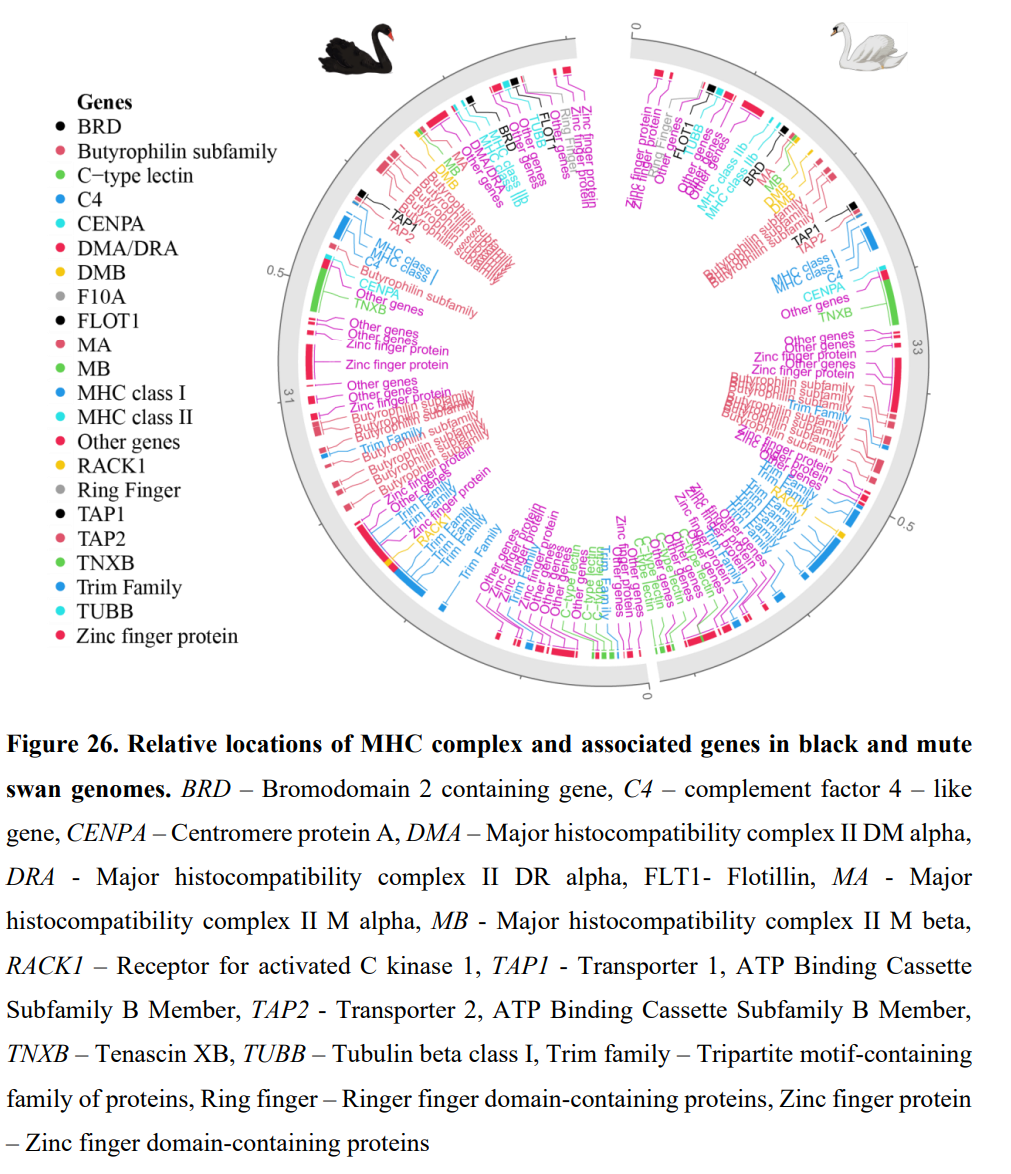


**Supplementary Figure S9: Relative locations of MHC complex and associated genes in black and mute swan chromosome 33.** *BRD* – Bromodomain 2 containing gene, *C4* – complement factor 4 – like gene, *CENPA* – Centromere protein A, *DMA* – Major histocompatibility complex II DM alpha, *DRA* - Major histocompatibility complex II DR alpha, FLT1- Flotillin, *MA* - Major histocompatibility complex II M alpha, *MB* - Major histocompatibility complex II M beta, *RACK1* – Receptor for activated C kinase 1, *TAP1* - Transporter 1, ATP Binding Cassette Subfamily B Member, *TAP2* - Transporter 2, ATP Binding Cassette Subfamily B Member, *TNXB* – Tenascin XB, *TUBB* – Tubulin beta class I, Trim family – Tripartite motif-containing family of proteins, Ring finger – Ringer finger domain-containing proteins, Zinc finger protein – Zinc finger domain-containing proteins

**
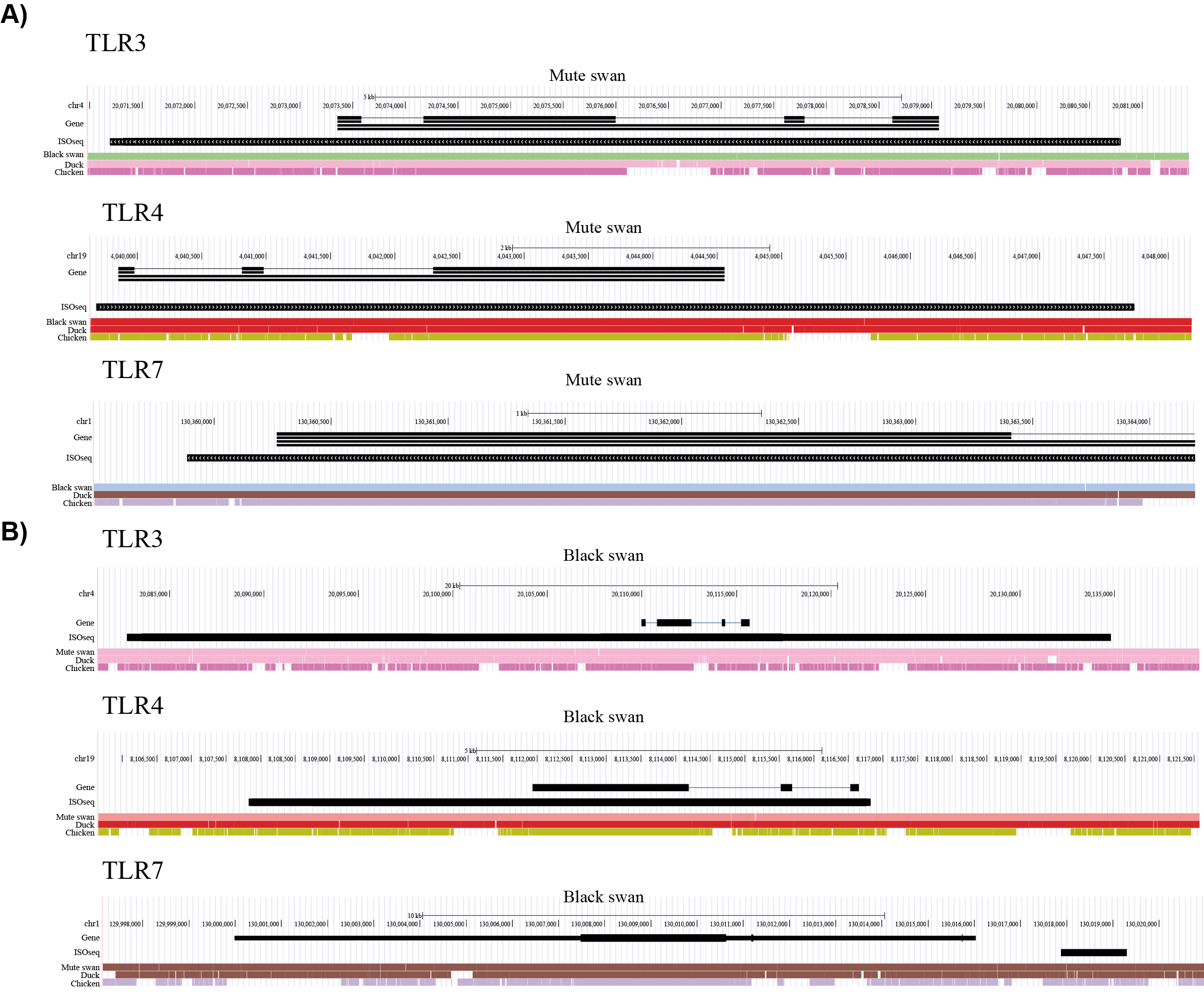
**

**Supplementary Figure S10: TLR7 expression can be detected in ISO Seq analysis of the mute swan (A) but not the black swan (B).** Chr: chromosome; Gene: the annotated gene model; ISOseq: ISOseq read alignment.

**
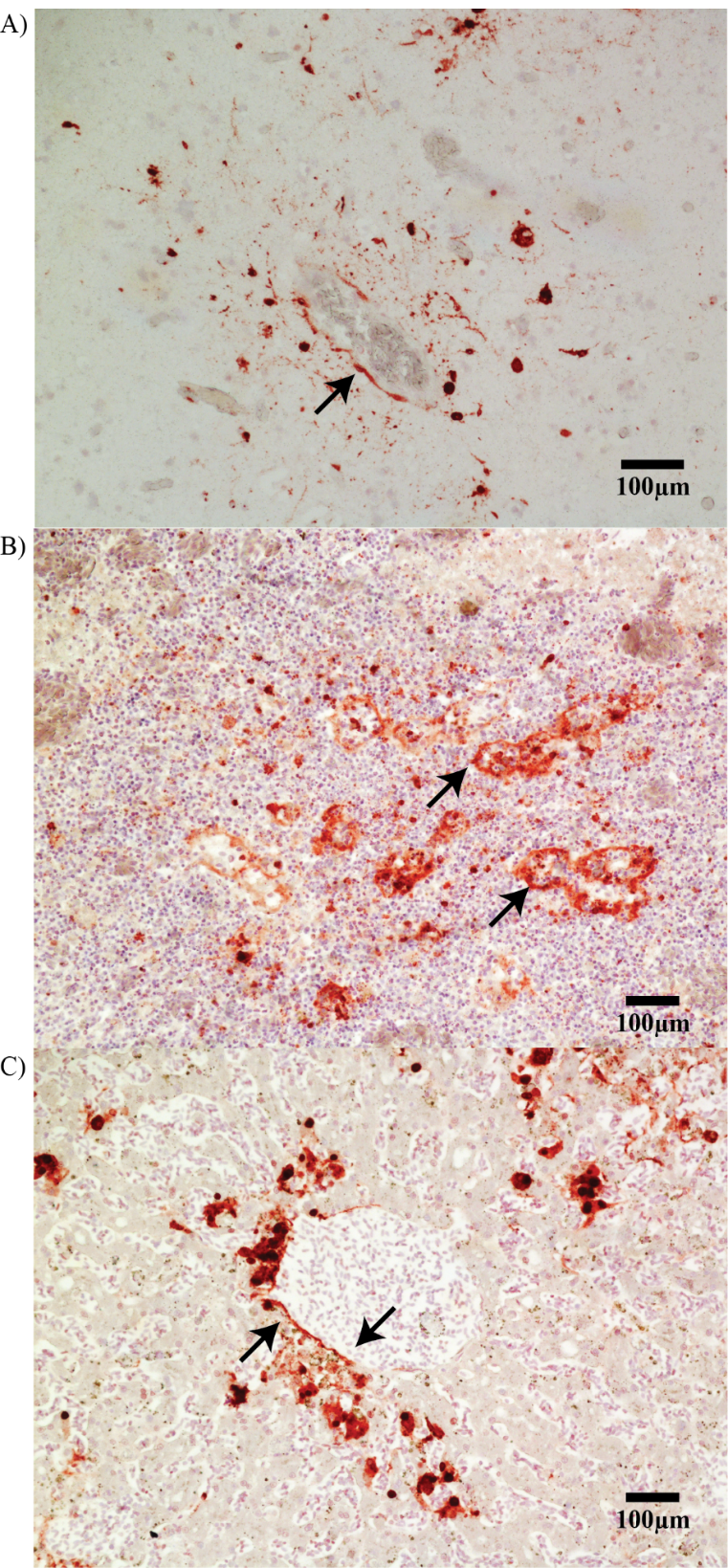
**

**Supplementary Figure S11: IAV NP antigen distribution in tissues from a black swan naturally infected with A/Black swan/Akita/2/2016 (H5N6) in 2016 in Akita prefecture, Japan.** Immunohistochemical detection of H5N6 nuclear protein in paraffin-embedded sections of black swan **A)** brain, **B)** spleen and **C)** liver (bar = 100µm). Arrows point to the IAV-positive endothelial cell lining. The presence of NP viral antigen is indicated by red/brown stain. No NP viral antigen was detected in the control stained serial sections.


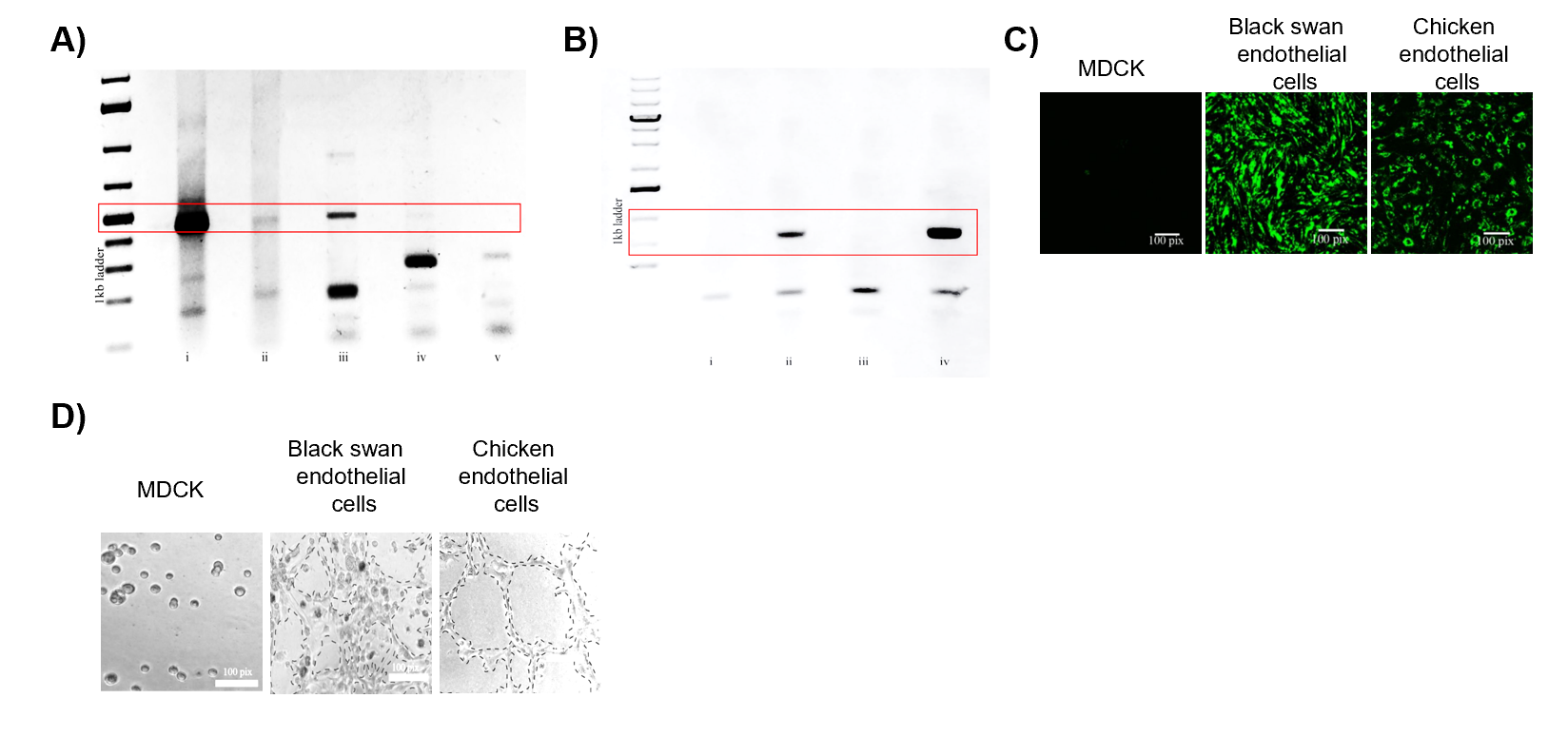


**Supplementary Figure S12: The successful culture of primary black swan endothelial cells confirmed by qRT-PCR, immunofluorescence and tube formation. A)** von Willebrand factor (vWF) expression in endothelial cells. i) duck aortic endothelial cells ii) duck bone marrow cells in culture for one day iii) black swan endothelial cells iv) black swan bone marrow cells in culture for one day v) black swan peripheral blood mononuclear cells. PCR amplicon size = ~450bp. **B)** CD45 expression in endothelial cells i) duck aortic endothelial cells ii) duck bone marrow cells in culture for one day iii) black swan endothelial cells iv) black swan bone marrow cells in culture for one day iv) black swan peripheral blood mononuclear cells. PCR amplicon size = ~310bp. **C)** Immunofluorescence of cells following a four hour incubation with FITC-labelled acetylated low-density lipoprotein. **D)** Cellular tube formation on solidified Cultrex® Basement Membrane Extract Type 2.


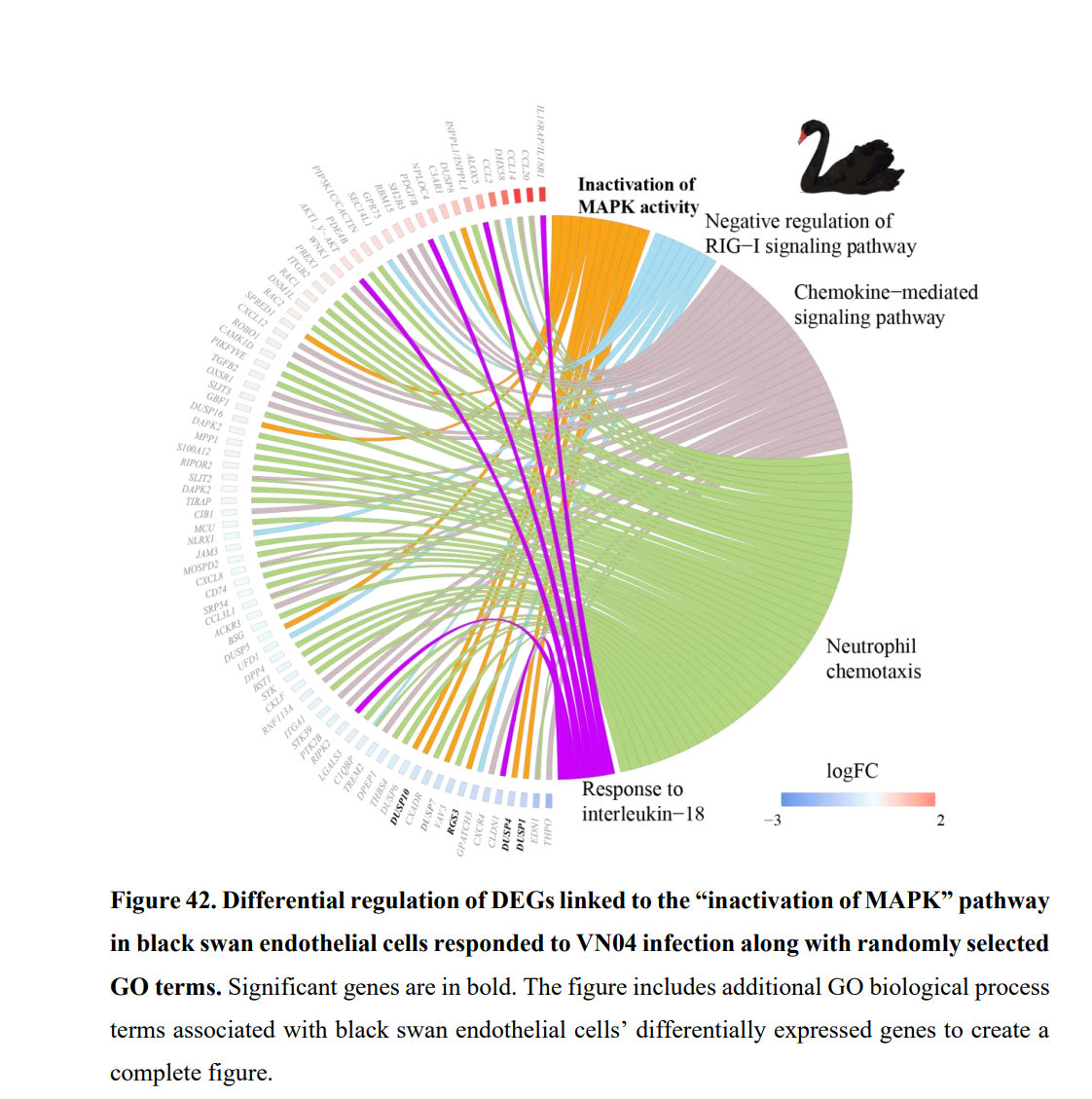


**Supplementary Figure S13: Differential regulation of DEGs linked to the “inactivation of MAPK’’ pathway in black swan endothelial cells in response to VN04 infection along with randomly selected GO terms.** Significant genes are in bold. The figure includes additional GO biological process terms associated with black swan endothelial cells’ DEGs to create a complete picture
