# Supplementary material for: The swan genome and transcriptome, it is not all black and white
Source: Genome Biol. 2023 Jan 23;24:13. doi: 10.1186/s13059-022-02838-0 (PMC9867998; doi:10.1186/s13059-022-02838-0)
Supplement: Supplementary file 2 — Additional file 2: Supplementary Table S1. Heterozygosity, QV and completeness values for swan genomes. [file 13059_2022_2838_MOESM2_ESM.docx]

**Supplementary Table S1:** Heterozygosity, QV and completeness values for swan genomes

| **Species** | **Black swan** | **Mute swan** |
| --- | --- | --- |
| **Heterozygosity** | 0.5% | 0.4% |
| **QV** | 40.9373 | 38.5247 |
| **Completeness value** | 95.0324% | 94.4407% |
