# Supplementary material for: The swan genome and transcriptome, it is not all black and white
Source: Genome Biol. 2023 Jan 23;24:13. doi: 10.1186/s13059-022-02838-0 (PMC9867998; doi:10.1186/s13059-022-02838-0)
Supplement: Supplementary file 4 — Additional file 4: Supplementary Table S2. BUSCO analysis of the final genomes. [file 13059_2022_2838_MOESM4_ESM.docx]

**Supplementary Table S2:** BUSCO analysis of the final genomes

| **BUSCOs categories** | **Black swan** | **Mute swan** | **Chicken (bGalGal1)** |
| --- | --- | --- | --- |
| **Complete** | 8093 | 8010 | 8054 |
| **Complete single copy** | 8061 | 7967 | 8021 |
| **Complete duplicated** | 32 | 43 | 33 |
| **Fragmented** | 56 | 66 | 67 |
| **Missing** | 189 | 262 | 216 |
