# Supplementary material for: The swan genome and transcriptome, it is not all black and white
Source: Genome Biol. 2023 Jan 23;24:13. doi: 10.1186/s13059-022-02838-0 (PMC9867998; doi:10.1186/s13059-022-02838-0)
Supplement: Supplementary file 5 — Additional file 5: Supplementary Table S3. CEGMA analysis of the final genomes. [file 13059_2022_2838_MOESM5_ESM.docx]

**Supplementary Table S3:** CEGMA analysis of the final genomes

| **CEGMA (core eukaryotic genes)** | **Black swan** | **Mute swan** | **Chicken (bGalGal1)** |
| --- | --- | --- | --- |
| **Complete** | 219 | 221 | 224 |
| **Complete and partial** | 226 | 228 | 229 |
| **Missing** | 22 | 20 | 19 |
| **Average ortho per gene** | 1.27 | 1.24 | 1.21 |
| **% of detected core genes that have more than one ortholog** | 20.55 | 19.46 | 16.52 |
