# Supplementary material for: The swan genome and transcriptome, it is not all black and white
Source: Genome Biol. 2023 Jan 23;24:13. doi: 10.1186/s13059-022-02838-0 (PMC9867998; doi:10.1186/s13059-022-02838-0)
Supplement: Supplementary file 6 — Additional file 6: Supplementary Table S4. GO biological process annotation of inverted genes in the Mallard duck (relative to the black swan). [file 13059_2022_2838_MOESM6_ESM.docx]

**Supplementary Table S4: GO biological process annotation of inverted genes in the Mallard duck (relative to the black swan)**

| **GO biological process** | **Gene count** |
| --- | --- |
| Cellular process (GO:0009987) | 319 |
| Reproductive process (GO:0022414) | 1 |
| Localisation (GO0051179) | 58 |
| Interspecies interaction between organisms (GO:0044419) | 4 |
| Reproduction (GO:0000003) | 1 |
| Biological regulation (GO:0065007) | 235 |
| Response to stimulus (GO:0050896) | 75 |
| Behaviour (GO:0007610) | 6 |
| Signalling (GO:0023052) | 69 |
| Developmental process (GO: 0032502) | 35 |
| Multicellular organismal process (GO:0032501) | 89 |
| Locomotion (GO:0040011) | 7 |
| Biological adhesion (GO0022610) | 6 |
| Metabolic process (GO:0008152) | 235 |
| Immune system process (GO:0002376) | 53 |
