# Supplementary material for: The swan genome and transcriptome, it is not all black and white
Source: Genome Biol. 2023 Jan 23;24:13. doi: 10.1186/s13059-022-02838-0 (PMC9867998; doi:10.1186/s13059-022-02838-0)
Supplement: Supplementary file 7 — Additional file 7: Supplementary Table S5. List of genes known to affect plumage colour in birds. [file 13059_2022_2838_MOESM7_ESM.docx]

**Supplementary Table S5: List of genes known to affect plumage colour in birds**

|  |  | Blast Results (Pairwise alignment) | |
| --- | --- | --- | --- |
|  |  | Coverage (against Mute swan gene) | Identity to Mute swan genome |
| melanocortin-1 receptor (MC1R) | (Guo, Li et al. 2010)  (Takeuchi, Suzuki et al. 1996)  (Gunnarsson, Hellström et al. 2007) | 100% | 98.99% |
| Agouti-signaling protein (ASIP) | (Estalles, Turbek et al. 2022) | 96.3% | 100% |
| Tyrosinase (TYR) | (Estalles, Turbek et al. 2022) | 100% | 99.43% |
| OCA2 Melanosomal Transmembrane Protein (OCA2) | (Estalles, Turbek et al. 2022) | 100% | 99.1% |
| HECT And RLD Domain Containing E3 Ubiquitin Protein Ligase 2 (HERC2) | (Estalles, Turbek et al. 2022) | 100% | 99.8% |
| Melan – A (MLANA) | (Estalles, Turbek et al. 2022) | 100% | 96.5% |
| solute carrier family 45, member 2, (SLC45A2) | (Estalles, Turbek et al. 2022) | 100% | 98.9% |
| Endothelin 3 (EDN3) | (Li, Sun et al. 2020) | 100% | 100% |
| PRELI Domain Containing 3B (SLMO2) | (Li, Sun et al. 2020) | 100% | 100% |
| ATP Synthase F1 Subunit Epsilon (ATP5E) | (Li, Sun et al. 2020) | 100% | 100% |
