# Supplementary material for: The swan genome and transcriptome, it is not all black and white
Source: Genome Biol. 2023 Jan 23;24:13. doi: 10.1186/s13059-022-02838-0 (PMC9867998; doi:10.1186/s13059-022-02838-0)
Supplement: Supplementary file 8 — Additional file 8: Supplementary Table S6. Immune gene families are contractive in black swans compared to mute swans. The number of genes in each immune gene sub-family identified is given in each column for the corresponding species. [file 13059_2022_2838_MOESM8_ESM.docx]

**Supplementary Table S6: Immune gene families are contractive in black swans compared to mute swans.** The number of genes in each immune gene sub-family identified is given in each column for the corresponding species

| **PANTHER protein sub-family** | **Family name** | ***Anas platyrhynchos*** | ***Gallus gallus*** | ***Cygnus atratus*** | ***Cygnus olor*** |
| --- | --- | --- | --- | --- | --- |
| PTHR10621:SF29 | UV Excision repair protein RAD23 Homolog A | 1 | 1 | 0 | 1 |
| PTHR11073:SF16 | Calreticulin | 1 | 1 | 0 | 1 |
| PTHR11829:SF68 | Forkhead Box Protein C1 | 1 | 0 | 0 | 1 |
| PTHR11955:SF56 | Retinol binding protein 1 | 1 | 1 | 0 | 1 |
| PTHR12306:SF10 | Cell death activator Cide-B | 1 | 0 | 0 | 1 |
| PTHR12610:SF23 | Single-stranded DNA binding Protein 2 | 1 | 1 | 0 | 2 |
| PTHR13843:SF11 | Mircotubule associated protein 1s | 1 | 1 | 0 | 1 |
| PTHR15492:SF1 | Cyclin-D1-Binding Protein 1 | 1 | 1 | 1 | 2 |
| PTHR20875:SF2 | EF-Hand calcium-binding domain-containing protein 6 | 1 | 2 | 0 | 4 |
| PTHR21540:SF3 | E3 Ubiquitin-protein ligase ZSWIM2 | 1 | 0 | 0 | 2 |
| PTHR22923:SF69 | Complement C1Q-like Protein 2 | 2 | 1 | 0 | 1 |
| PTHR22940:SF4 | Protein timeless homolog | 1 | 1 | 0 | 1 |
| PTHR23238:SF5 | RNA-Binding Protein FUS | 0 | 1 | 0 | 1 |
| PTHR23347:SF5 | USHER SYNDROME TYPE-1C PROTEIN-BINDING PROTEIN 1 | 1 | 2 | 0 | 1 |
| PTHR24026:SF36 | [CADHERIN EGF LAG SEVEN-PASS G-TYPE RECEPTOR 1](http://www.pantherdb.org/panther/family.do?clsAccession=PTHR24026:SF36) | 1 | 0 | 0 | 1 |
| PTHR24346:SF56 | [SERINE/THREONINE-PROTEIN KINASE MARK2](http://www.pantherdb.org/panther/family.do?clsAccession=PTHR24346:SF56) | 2 | 1 | 0 | 1 |
| PTHR31770:SF2 | [CHEMOKINE-LIKE PROTEIN TAFA-1](http://www.pantherdb.org/panther/family.do?clsAccession=PTHR31770:SF2) | 1 | 1 | 0 | 1 |
| PTHR44329:SF6 | [RECEPTOR-INTERACTING SERINE/THREONINE-PROTEIN KINASE 1](http://www.pantherdb.org/panther/family.do?clsAccession=PTHR44329:SF6) | 1 | 1 | 0 | 1 |
| PTHR45636:SF26 | [PAIRED BOX PROTEIN PAX-7](http://www.pantherdb.org/panther/family.do?clsAccession=PTHR45636:SF26) | 1 | 1 | 0 | 1 |
| PTHR45664:SF11 | [HOMEOBOX PROTEIN HOX-B3](http://www.pantherdb.org/panther/family.do?clsAccession=PTHR45664:SF11) | 1 | 1 | 0 | 1 |
| PTHR45807:SF6 | [NON-RECEPTOR TYROSINE-PROTEIN KINASE TYK2](http://www.pantherdb.org/panther/family.do?clsAccession=PTHR45807:SF6) | 1 | 1 | 0 | 1 |
| PTHR45894:SF1 | [RNA-BINDING PROTEIN 8A](http://www.pantherdb.org/panther/family.do?clsAccession=PTHR45894:SF1) | 1 | 1 | 0 | 1 |
| PTHR46318:SF4 | [NUCLEOLAR TRANSCRIPTION FACTOR 1](http://www.pantherdb.org/panther/family.do?clsAccession=PTHR46318:SF4) | 1 | 1 | 0 | 1 |
| PTHR10155:SF6 | [SRC-LIKE-ADAPTER 2](http://www.pantherdb.org/panther/family.do?clsAccession=PTHR10155:SF6) | 2 | 1 | 1 | 2 |
| PTHR10460:SF2 | [ABL INTERACTOR 1](http://www.pantherdb.org/panther/family.do?clsAccession=PTHR10460:SF2) | 2 | 1 | 1 | 2 |
| PTHR11412:SF86 | [COMPLEMENT C4-A-RELATED](http://www.pantherdb.org/panther/family.do?clsAccession=PTHR11412:SF86) | 2 | 2 | 1 | 2 |
| PTHR11753:SF45 | [AP-1 COMPLEX SUBUNIT SIGMA-2](http://www.pantherdb.org/panther/family.do?clsAccession=PTHR11753:SF45) | 2 | 1 | 1 | 2 |
| PTHR13703:SF42 | [MOTHERS AGAINST DECAPENTAPLEGIC HOMOLOG 2](http://www.pantherdb.org/panther/family.do?clsAccession=PTHR13703:SF42) | 3 | 4 | 1 | 4 |
| PTHR14879:SF2 | [E3 UBIQUITIN-PROTEIN LIGASE RIFIFYLIN](http://www.pantherdb.org/panther/family.do?clsAccession=PTHR14879:SF2) | 2 | 1 | 1 | 2 |
| PTHR16830:SF13 | [FYN-BINDING PROTEIN 1](http://www.pantherdb.org/panther/family.do?clsAccession=PTHR16830:SF13) | 3 | 2 | 1 | 3 |
| PTHR22741:SF11 | [SICKLE TAIL PROTEIN HOMOLOG](http://www.pantherdb.org/panther/family.do?clsAccession=PTHR22741:SF11) | 1 | 1 | 1 | 2 |
| PTHR32546:SF11 | [G-PROTEIN COUPLED RECEPTOR 158-RELATED](http://www.pantherdb.org/panther/family.do?clsAccession=PTHR32546:SF11) | 2 | 1 | 1 | 2 |
| PTHR43607:SF1 | [V-TYPE PROTON ATPASE CATALYTIC SUBUNIT A](http://www.pantherdb.org/panther/family.do?clsAccession=PTHR43607:SF1) | 2 | 2 | 1 | 2 |
| PTHR48021:SF59 | [SOLUTE CARRIER FAMILY 2, FACILITATED GLUCOSE TRANSPORTER MEMBER 6](http://www.pantherdb.org/panther/family.do?clsAccession=PTHR48021:SF59) | 2 | 1 | 1 | 2 |
| PTHR22826:SF104 | [TRIPLE FUNCTIONAL DOMAIN PROTEIN](http://www.pantherdb.org/panther/family.do?clsAccession=PTHR22826:SF104) | 4 | 4 | 2 | 3 |
| PTHR23036:SF29 | [INTERLEUKIN-11 RECEPTOR SUBUNIT ALPHA](http://www.pantherdb.org/panther/family.do?clsAccession=PTHR23036:SF29) | 1 | 1 | 1 | 2 |
| PTHR11267:SF104 | [T-BOX TRANSCRIPTION FACTOR TBX1](http://www.pantherdb.org/panther/family.do?clsAccession=PTHR11267:SF104) | 4 | 2 | 3 | 4 |
| PTHR24046:SF4 | [SIGNAL PEPTIDE, CUB AND EGF-LIKE DOMAIN-CONTAINING PROTEIN 1](http://www.pantherdb.org/panther/family.do?clsAccession=PTHR24046:SF4) | 4 | 3 | 3 | 4 |
| PTHR11709:SF226 | [CERULOPLASMIN](http://www.pantherdb.org/panther/family.do?clsAccession=PTHR11709:SF226) | 8 | 8 | 4 | 5 |
