# Supplementary material for: The swan genome and transcriptome, it is not all black and white
Source: Genome Biol. 2023 Jan 23;24:13. doi: 10.1186/s13059-022-02838-0 (PMC9867998; doi:10.1186/s13059-022-02838-0)
Supplement: Supplementary file 9 — Additional file 9: Supplementary Table S7. TLR7 expression could not be detected in black swan spleen, liver and kidney by qRT-PCR. *ND: Not detected. [file 13059_2022_2838_MOESM9_ESM.docx]

**Supplementary Table S7: TLR7 expression could not be detected in black swan spleen, liver and kidney by qRT-PCR. *ND: Not detected**

| **Sample** | **18S Ct** | **TLR7 Ct** |
| --- | --- | --- |
| Black swan 1 Kidney | 16.90791 | ND^*^ |
| Black swan 1 Spleen | 29.62261 | ND |
| Black swan 1 Liver | 14.65389 | ND |
| Black swan 2 Kidney | 15.75131 | ND |
| Black swan 2 Liver | 20.61559 | ND |
| Black swan genomic DNA | 25.61549 | 29.36461 |
| Black swan RNA | ND | ND |
