# Supplementary material for: The swan genome and transcriptome, it is not all black and white
Source: Genome Biol. 2023 Jan 23;24:13. doi: 10.1186/s13059-022-02838-0 (PMC9867998; doi:10.1186/s13059-022-02838-0)
Supplement: Supplementary file 10 — Additional file 10: Supplementary Table S8. Differentially expressed genes in infected black swan endothelial cells. [file 13059_2022_2838_MOESM10_ESM.docx]

**Supplementary Table S8: Differentially expressed genes in infected black swan endothelial cells**

| Gene Name | log2FoldChange | p-value | p-adj |
| --- | --- | --- | --- |
| LOC118246182 | 4.580175048 | 2.79E-91 | 3.68E-87 |
| ENSACYG00000016114 | 6.195069474 | 7.14E-76 | 4.71E-72 |
| RSAD2 | 3.695484293 | 2.36E-74 | 1.04E-70 |
| LOC118254221 | 3.58506455 | 3.62E-60 | 1.19E-56 |
| USP18 | 3.502175442 | 2.68E-59 | 7.07E-56 |
| HELZ2 | 3.645057686 | 2.48E-46 | 5.46E-43 |
| RGS2 | -2.383023552 | 3.86E-44 | 7.28E-41 |
| ENSACYG00000016981 | 3.41066558 | 8.38E-42 | 1.38E-38 |
| LOC118247687 | 3.652176405 | 1.30E-34 | 1.91E-31 |
| SPRY1 | -1.782290425 | 8.67E-25 | 1.14E-21 |
| DHX58 | 2.148332185 | 2.75E-24 | 3.30E-21 |
| CCL20 | 3.574765402 | 3.36E-23 | 3.43E-20 |
| TNFSF8 | 4.466255163 | 3.38E-23 | 3.43E-20 |
| LOC118247453 | 2.445280893 | 2.39E-22 | 2.25E-19 |
| CMPK2 | 1.816769385 | 6.59E-21 | 5.61E-18 |
| CCN2 | -1.484283988 | 6.80E-21 | 5.61E-18 |
| EIF2AK2 | 1.642487532 | 1.29E-20 | 1.00E-17 |
| LOC118253574 | 5.72343541 | 7.52E-20 | 5.51E-17 |
| LOC118257698 | 7.095131916 | 2.51E-19 | 1.74E-16 |
| EDN1 | -1.588052126 | 8.88E-19 | 5.86E-16 |
| JUN | -1.379333866 | 4.92E-18 | 3.09E-15 |
| LOC118243513 | 3.561929001 | 1.83E-17 | 1.10E-14 |
| ENSACYG00000007562 | 4.962435236 | 1.14E-16 | 6.55E-14 |
| ENSACYG00000015363 | 2.617776861 | 1.63E-16 | 8.98E-14 |
| GRN | -1.185595554 | 8.03E-16 | 4.24E-13 |
| DDIT4 | -1.795782961 | 1.27E-15 | 6.44E-13 |
| ENSACYG00000007800 | -2.2221888 | 2.31E-15 | 1.13E-12 |
| HSPA2 | -1.072147928 | 5.95E-15 | 2.80E-12 |
| RRAD | -1.294474384 | 7.50E-15 | 3.33E-12 |
| ENSACYG00000014576 | 6.252514906 | 7.57E-15 | 3.33E-12 |
| SRSF5 | -1.582237015 | 9.76E-15 | 4.16E-12 |
| FSIP1 | 2.278172621 | 1.47E-14 | 6.04E-12 |
| ST6GALNAC1 | 2.164273093 | 7.22E-14 | 2.89E-11 |
| CITED4 | -1.249362893 | 1.01E-13 | 3.90E-11 |
| ENSACYG00000007716 | 4.290252805 | 1.03E-13 | 3.90E-11 |
| TNFAIP2 | 1.21345486 | 1.25E-13 | 4.58E-11 |
| ENSACYG00000003191 | 3.256022692 | 1.33E-13 | 4.73E-11 |
| SHQ1 | -1.457936056 | 1.63E-13 | 5.66E-11 |
| HSP90AA1 | -1.015067647 | 8.86E-13 | 2.99E-10 |
| ENC1 | -1.290660525 | 9.05E-13 | 2.99E-10 |
| CREB5 | 1.14513956 | 2.59E-12 | 8.33E-10 |
| FAAH2 | -1.184134502 | 2.97E-12 | 9.34E-10 |
| MAP1LC3C | -1.432055932 | 4.32E-12 | 1.33E-09 |
| DUSP4 | -1.335636982 | 4.88E-12 | 1.47E-09 |
| LOC118244434 | 1.080351876 | 5.43E-12 | 1.59E-09 |
| ATP2B1 | -1.04326864 | 7.84E-12 | 2.25E-09 |
| LOC118259586 | 3.69922664 | 8.63E-12 | 2.42E-09 |
| ING1 | -1.438405111 | 9.52E-12 | 2.62E-09 |
| IL12RB2 | 2.59592289 | 1.14E-11 | 3.08E-09 |
| LOC118250194 | 1.302368927 | 1.24E-11 | 3.28E-09 |
| FASN | -1.011409185 | 1.61E-11 | 4.17E-09 |
| TCIM | -1.989711736 | 1.75E-11 | 4.43E-09 |
| KLF2 | -1.249259299 | 2.61E-11 | 6.51E-09 |
| LOC118260896 | -1.316698577 | 3.31E-11 | 8.09E-09 |
| DDX60 | 1.686597891 | 3.49E-11 | 8.37E-09 |
| BTG1 | -1.077831805 | 4.32E-11 | 1.02E-08 |
| DNAH17 | 3.092624528 | 4.80E-11 | 1.11E-08 |
| NEU3 | -1.946778268 | 5.09E-11 | 1.16E-08 |
| COL9A3 | 3.08487153 | 5.62E-11 | 1.26E-08 |
| ENSACYG00000008104 | -1.43977034 | 5.73E-11 | 1.26E-08 |
| NR0B1 | -1.663653787 | 7.49E-11 | 1.62E-08 |
| MARS2 | -1.290585705 | 8.41E-11 | 1.79E-08 |
| SLC16A10 | 2.387959825 | 8.79E-11 | 1.84E-08 |
| CUNH3orf52 | -1.408583777 | 1.42E-10 | 2.93E-08 |
| VCAN | -0.936519302 | 1.45E-10 | 2.95E-08 |
| PTX3 | -0.853272376 | 1.59E-10 | 3.17E-08 |
| ID2 | -1.454905827 | 1.72E-10 | 3.39E-08 |
| PLEKHA6 | 1.701525256 | 1.77E-10 | 3.44E-08 |
| RIOX1 | -1.090113776 | 1.95E-10 | 3.74E-08 |
| METTL18 | -1.119667826 | 2.16E-10 | 4.07E-08 |
| SPRY2 | -1.313198245 | 2.71E-10 | 5.04E-08 |
| RBM20 | 3.491956132 | 3.56E-10 | 6.52E-08 |
| LINGO1 | 0.919570232 | 5.40E-10 | 9.77E-08 |
| ENSACYG00000017295 | 2.632321833 | 6.48E-10 | 1.16E-07 |
| MITD1 | 1.025077977 | 7.98E-10 | 1.41E-07 |
| FANCF | -1.171218516 | 8.79E-10 | 1.53E-07 |
| SLC35E4 | -0.954045479 | 9.59E-10 | 1.64E-07 |
| CCKAR | 4.071263531 | 1.15E-09 | 1.95E-07 |
| FBXO48 | -1.282634229 | 1.21E-09 | 2.00E-07 |
| GTF2B | -0.980863724 | 1.21E-09 | 2.00E-07 |
| ENSACYG00000005816 | 4.908638144 | 1.33E-09 | 2.17E-07 |
| LOC118258706 | 1.551283193 | 1.37E-09 | 2.20E-07 |
| IRF7 | 1.745901208 | 1.45E-09 | 2.22E-07 |
| SLC2A9 | 7.797230023 | 1.44E-09 | 2.22E-07 |
| IL6 | 1.844550235 | 1.41E-09 | 2.22E-07 |
| ENSACYG00000008294 | 4.275266889 | 1.42E-09 | 2.22E-07 |
| LOC118254002 | 1.688382627 | 1.54E-09 | 2.33E-07 |
| TBXAS1 | 2.818796526 | 1.66E-09 | 2.48E-07 |
| LOC118254070 | -0.852998203 | 1.78E-09 | 2.64E-07 |
| ZFAND2B | -1.230402071 | 2.26E-09 | 3.32E-07 |
| TRUB1 | -1.119258098 | 2.66E-09 | 3.81E-07 |
| NELFA | -0.924509813 | 2.65E-09 | 3.81E-07 |
| DUS3L | -1.24552966 | 2.81E-09 | 3.99E-07 |
| SASS6 | -0.98428245 | 3.30E-09 | 4.63E-07 |
| ENSACYG00000001324 | -1.237713592 | 3.40E-09 | 4.73E-07 |
| R3HDM1 | 0.880811897 | 4.10E-09 | 5.64E-07 |
| RRBP1 | -0.864458522 | 4.15E-09 | 5.65E-07 |
| ENSACYG00000012255 | 3.554320128 | 4.62E-09 | 6.22E-07 |
| CCN1 | -1.086904006 | 5.34E-09 | 7.05E-07 |
| SLC38A2 | 0.975729567 | 5.29E-09 | 7.05E-07 |
| DUSP1 | -1.083200374 | 6.57E-09 | 8.59E-07 |
| LOC118248062 | 3.03903724 | 8.56E-09 | 1.11E-06 |
| MYH9 | -1.055902423 | 8.98E-09 | 1.15E-06 |
| G0S2 | -1.109953144 | 9.35E-09 | 1.19E-06 |
| VCAM1 | 1.790847269 | 1.05E-08 | 1.32E-06 |
| SLC26A4 | 6.376011231 | 1.12E-08 | 1.40E-06 |
| LOC118251676 | 3.447902462 | 1.13E-08 | 1.40E-06 |
| TPM1 | 2.402101785 | 1.15E-08 | 1.41E-06 |
| TPR | -0.950697258 | 1.21E-08 | 1.46E-06 |
| HAUS8 | -0.864383898 | 1.21E-08 | 1.46E-06 |
| DNPEP | -1.066736825 | 1.26E-08 | 1.49E-06 |
| MED18 | -1.197063088 | 1.26E-08 | 1.49E-06 |
| HEXB | -0.813078015 | 1.38E-08 | 1.60E-06 |
| LOC118251878 | 1.456187298 | 1.38E-08 | 1.60E-06 |
| ENSACYG00000008278 | 2.938877762 | 1.54E-08 | 1.77E-06 |
| NEMP2 | 1.061732056 | 1.56E-08 | 1.77E-06 |
| CUNH2orf49 | -0.907704957 | 1.68E-08 | 1.90E-06 |
| SYNE1 | -0.980365761 | 1.80E-08 | 2.01E-06 |
| ESS2 | -1.111893384 | 1.96E-08 | 2.18E-06 |
| MED13 | 1.090369967 | 2.02E-08 | 2.22E-06 |
| RASL11B | -0.899780172 | 2.08E-08 | 2.25E-06 |
| ENSACYG00000012368 | 3.125181819 | 2.07E-08 | 2.25E-06 |
| MCL1 | -0.943125585 | 2.13E-08 | 2.29E-06 |
| SNAPC3 | -1.03563998 | 2.20E-08 | 2.34E-06 |
| MYC | -0.892925534 | 2.39E-08 | 2.52E-06 |
| ARL4A | -0.961099269 | 2.57E-08 | 2.70E-06 |
| NECTIN3 | -0.792077121 | 2.61E-08 | 2.72E-06 |
| SLC5A1 | 3.993031358 | 2.67E-08 | 2.75E-06 |
| MMAA | -0.913567628 | 3.06E-08 | 3.13E-06 |
| HIPK2 | 0.888853789 | 3.12E-08 | 3.17E-06 |
| CCNL2 | -1.061472207 | 3.90E-08 | 3.93E-06 |
| LOC118244242 | -0.755677579 | 4.25E-08 | 4.25E-06 |
| CLDN1 | -0.825540386 | 4.78E-08 | 4.74E-06 |
| ZBTB25 | -0.974410821 | 5.39E-08 | 5.31E-06 |
| WEE1 | -1.164838979 | 5.43E-08 | 5.31E-06 |
| PPP2R2B | 0.838024139 | 5.72E-08 | 5.56E-06 |
| TENT5A | -0.875170139 | 5.89E-08 | 5.67E-06 |
| SMTN | -0.836298296 | 6.28E-08 | 6.01E-06 |
| ARL10 | -1.156946133 | 6.50E-08 | 6.18E-06 |
| ENSACYG00000009359 | 2.011367472 | 6.86E-08 | 6.45E-06 |
| TXLNB | 3.414940487 | 6.89E-08 | 6.45E-06 |
| ENSACYG00000000041 | 2.6435625 | 7.27E-08 | 6.76E-06 |
| EXOSC3 | -1.235916813 | 7.77E-08 | 7.18E-06 |
| TRA2B | -0.908413209 | 8.74E-08 | 8.01E-06 |
| ATF4 | -0.924615219 | 8.86E-08 | 8.07E-06 |
| IL1RL1 | 1.614160155 | 9.01E-08 | 8.15E-06 |
| ENSACYG00000013288 | -1.391271802 | 9.51E-08 | 8.54E-06 |
| LIPT1 | -0.984761858 | 1.05E-07 | 9.31E-06 |
| LOC118258158 | 1.070987532 | 1.05E-07 | 9.31E-06 |
| LOC118249052 | -0.805846326 | 1.07E-07 | 9.38E-06 |
| BMPR1B | 4.494371119 | 1.11E-07 | 9.70E-06 |
| LOC118248085 | -1.110420319 | 1.13E-07 | 9.83E-06 |
| CCM2 | 0.755094907 | 1.17E-07 | 9.93E-06 |
| DND1 | -3.273289986 | 1.17E-07 | 9.93E-06 |
| RND2 | -1.089125135 | 1.15E-07 | 9.93E-06 |
| PAX1 | 1.134763224 | 1.17E-07 | 9.93E-06 |
| CCNB2 | -0.927169767 | 1.18E-07 | 9.95E-06 |
| PARS2 | -0.915264627 | 1.23E-07 | 1.03E-05 |
| ANAPC7 | -0.921617429 | 1.28E-07 | 1.06E-05 |
| EXOSC9 | -0.932195397 | 1.33E-07 | 1.09E-05 |
| LOC118253381 | -1.654932298 | 1.33E-07 | 1.09E-05 |
| WSB1 | -0.765716622 | 1.36E-07 | 1.11E-05 |
| WDR89 | -0.887090585 | 1.40E-07 | 1.13E-05 |
| ASPHD2 | 1.059771392 | 1.43E-07 | 1.15E-05 |
| ENSACYG00000008802 | 7.083971698 | 1.52E-07 | 1.22E-05 |
| NLRC5 | 1.769350073 | 1.59E-07 | 1.26E-05 |
| POC1A | -0.997006512 | 1.60E-07 | 1.26E-05 |
| DYNC1H1 | -0.854031071 | 1.62E-07 | 1.27E-05 |
| POP1 | -0.960294536 | 1.66E-07 | 1.29E-05 |
| FLNB | -0.854381119 | 1.69E-07 | 1.31E-05 |
| PLEC | -1.06066991 | 1.87E-07 | 1.44E-05 |
| ENSACYG00000002531 | 4.452858297 | 1.90E-07 | 1.46E-05 |
| LOC118245596 | 7.092582392 | 1.98E-07 | 1.51E-05 |
| PABPC1 | 0.74800618 | 2.09E-07 | 1.58E-05 |
| PUS3 | -0.839919272 | 2.13E-07 | 1.61E-05 |
| TLN1 | -1.227764504 | 2.25E-07 | 1.69E-05 |
| FERMT1 | -0.793829415 | 2.39E-07 | 1.78E-05 |
| ENSACYG00000017004 | 7.011333312 | 2.48E-07 | 1.84E-05 |
| GCH1 | 1.002391677 | 2.52E-07 | 1.86E-05 |
| TGDS | -0.871534306 | 2.78E-07 | 2.04E-05 |
| NOC3L | -0.809976362 | 2.85E-07 | 2.08E-05 |
| NCOA7 | 0.904600837 | 2.95E-07 | 2.14E-05 |
| LOC118247374 | -1.049362264 | 3.33E-07 | 2.40E-05 |
| CDC20 | -0.800666466 | 3.42E-07 | 2.46E-05 |
| LOC118261382 | 1.247185742 | 3.46E-07 | 2.47E-05 |
| CXCR4 | -0.856779646 | 3.52E-07 | 2.50E-05 |
| OGT | -0.817270643 | 3.76E-07 | 2.63E-05 |
| ENSACYG00000009286 | 2.508441777 | 3.76E-07 | 2.63E-05 |
| ENSACYG00000012852 | 1.837357737 | 3.73E-07 | 2.63E-05 |
| ARMC6 | -0.733491202 | 3.87E-07 | 2.69E-05 |
| EPSTI1 | 1.762138054 | 3.93E-07 | 2.71E-05 |
| ENSACYG00000000249 | -1.006917773 | 3.99E-07 | 2.74E-05 |
| ALG11 | -0.845949625 | 4.16E-07 | 2.84E-05 |
| TRAF7 | 0.822751166 | 4.17E-07 | 2.84E-05 |
| PHOSPHO1 | 1.129145206 | 4.35E-07 | 2.93E-05 |
| MYCN | -1.066468717 | 4.37E-07 | 2.93E-05 |
| RNF217 | 0.911279599 | 4.36E-07 | 2.93E-05 |
| SLC9A4 | 6.92936322 | 4.41E-07 | 2.93E-05 |
| KLHL42 | -0.797332517 | 4.42E-07 | 2.93E-05 |
| PIGL | -0.796261666 | 4.54E-07 | 2.99E-05 |
| RPP25L | -0.771593225 | 4.66E-07 | 3.06E-05 |
| SRSF7 | -0.82592492 | 4.73E-07 | 3.09E-05 |
| TMEM200B | 1.073437235 | 4.96E-07 | 3.23E-05 |
| ENSACYG00000011789 | 5.116057588 | 5.00E-07 | 3.24E-05 |
| ATP2A2 | -0.727762152 | 5.11E-07 | 3.29E-05 |
| ZBTB20 | 0.912955108 | 5.26E-07 | 3.37E-05 |
| GOT2 | -0.671124793 | 5.36E-07 | 3.42E-05 |
| POLR1F | -1.069375365 | 5.70E-07 | 3.62E-05 |
| LOC118244925 | -0.952798602 | 5.96E-07 | 3.77E-05 |
| WIF1 | -0.815473635 | 6.42E-07 | 4.03E-05 |
| SETD6 | -1.047046099 | 6.60E-07 | 4.13E-05 |
| DYNAP | 4.073014401 | 6.77E-07 | 4.22E-05 |
| INPP5B | -1.162274219 | 7.00E-07 | 4.34E-05 |
| CX3CL1 | 1.939813044 | 7.17E-07 | 4.41E-05 |
| FBXO42 | 0.880471227 | 7.18E-07 | 4.41E-05 |
| CLK1 | -0.89144568 | 7.68E-07 | 4.69E-05 |
| PIM3 | -0.703341269 | 7.79E-07 | 4.74E-05 |
| SURF2 | -1.035464848 | 8.00E-07 | 4.85E-05 |
| MAP3K8 | 1.077754107 | 8.19E-07 | 4.94E-05 |
| LONRF3 | 0.731613904 | 8.77E-07 | 5.26E-05 |
| LOC118253911 | -0.845608339 | 9.37E-07 | 5.60E-05 |
| ATM | -0.82493099 | 9.52E-07 | 5.66E-05 |
| JMJD4 | 0.718561255 | 9.68E-07 | 5.73E-05 |
| LOC118248082 | -0.904416384 | 9.85E-07 | 5.81E-05 |
| LOC118254130 | 1.391697946 | 1.01E-06 | 5.90E-05 |
| PKP4 | 0.847441798 | 1.03E-06 | 6.01E-05 |
| BTG2 | -0.999699575 | 1.07E-06 | 6.25E-05 |
| MTG2 | -0.96681488 | 1.10E-06 | 6.36E-05 |
| HSF1 | 0.884756713 | 1.13E-06 | 6.52E-05 |
| SCNN1B | 0.684176826 | 1.16E-06 | 6.63E-05 |
| LOC118244481 | 2.115945447 | 1.26E-06 | 7.21E-05 |
| ENSACYG00000000109 | 1.916095771 | 1.31E-06 | 7.46E-05 |
| MYBL1 | 0.828317566 | 1.33E-06 | 7.52E-05 |
| DGKH | 0.800113915 | 1.44E-06 | 8.12E-05 |
| CWC25 | -1.21363937 | 1.45E-06 | 8.12E-05 |
| TRIM45 | -0.9787944 | 1.46E-06 | 8.15E-05 |
| ENSACYG00000004093 | 3.397086649 | 1.53E-06 | 8.53E-05 |
| LOC118253948 | 0.816152101 | 1.58E-06 | 8.74E-05 |
| LMBRD1 | -0.73086765 | 1.60E-06 | 8.81E-05 |
| DOK1 | -1.033069502 | 1.60E-06 | 8.81E-05 |
| E2F6 | -1.005766608 | 1.62E-06 | 8.86E-05 |
| CMYA5 | 3.365914847 | 1.63E-06 | 8.91E-05 |
| ENSACYG00000007266 | 2.602613758 | 1.67E-06 | 9.10E-05 |
| HERPUD1 | -0.747666229 | 1.69E-06 | 9.17E-05 |
| ADM | -0.97699209 | 1.82E-06 | 9.82E-05 |
| SLC16A12 | 3.522122914 | 1.96E-06 | 0.000104634 |
| LOC118261525 | 6.680507627 | 1.96E-06 | 0.000104634 |
| RUBCNL | -0.902166764 | 1.99E-06 | 0.000105424 |
| OGFR | 0.762863693 | 1.99E-06 | 0.000105424 |
| UTP23 | -0.887940824 | 2.23E-06 | 0.000117894 |
| KLHDC1 | -0.882203126 | 2.33E-06 | 0.000122752 |
| LOC118247601 | 6.627722545 | 2.36E-06 | 0.000123823 |
| LRRC47 | -0.937035309 | 2.37E-06 | 0.000123911 |
| DDX49 | -0.786737853 | 2.51E-06 | 0.00013021 |
| ATP5F1A | -0.754237952 | 2.63E-06 | 0.000135935 |
| LOC118251879 | 1.392034441 | 2.67E-06 | 0.000137177 |
| SDR16C5 | -0.746705121 | 2.67E-06 | 0.000137177 |
| ANKRD37 | -0.726028123 | 2.80E-06 | 0.000143104 |
| ZNF143 | -0.837150234 | 2.99E-06 | 0.000152178 |
| ZNFX1 | -0.65411607 | 3.15E-06 | 0.000159749 |
| NES | -1.205426175 | 3.18E-06 | 0.000160732 |
| ANAPC10 | -1.155238545 | 3.29E-06 | 0.000165558 |
| ATXN7 | 0.927624662 | 3.36E-06 | 0.000168883 |
| LOC118259027 | -0.928018269 | 3.40E-06 | 0.000169661 |
| FAM53A | -0.715286518 | 3.41E-06 | 0.000169661 |
| SLC48A1 | 0.675994487 | 3.73E-06 | 0.000185015 |
| CNOT10 | -0.792394127 | 3.87E-06 | 0.00019068 |
| MED27 | -0.736891508 | 3.87E-06 | 0.00019068 |
| LOC118247555 | -0.715443409 | 3.94E-06 | 0.000193502 |
| METTL21A | -0.919231573 | 4.02E-06 | 0.000196681 |
| ATG10 | 0.842633025 | 4.05E-06 | 0.000197262 |
| TKT | -0.657973246 | 4.10E-06 | 0.000198891 |
| AKAP10 | 0.80482146 | 4.14E-06 | 0.000200173 |
| ENSACYG00000011963 | -1.049804387 | 4.15E-06 | 0.000200173 |
| SLC34A2 | 1.736195424 | 4.22E-06 | 0.000202386 |
| MBTPS1 | 0.829753652 | 4.26E-06 | 0.000203531 |
| POMGNT2 | 0.673430773 | 4.28E-06 | 0.000203752 |
| LOC118252548 | -1.021354336 | 4.35E-06 | 0.000206554 |
| LOC118251921 | -1.054889039 | 4.39E-06 | 0.000207709 |
| MYH10 | -0.658155848 | 4.41E-06 | 0.000207709 |
| MTA3 | -1.01770599 | 4.46E-06 | 0.000209524 |
| PRKCD | -0.694449713 | 4.58E-06 | 0.000214408 |
| ACTR5 | -1.074077064 | 4.64E-06 | 0.000216459 |
| LOC118260289 | -0.789108077 | 4.82E-06 | 0.000223988 |
| BAMBI | -0.810476031 | 4.84E-06 | 0.000224169 |
| ERICH1 | -0.922975817 | 4.90E-06 | 0.000225971 |
| CD83 | 2.439266654 | 5.31E-06 | 0.000244139 |
| NAT10 | -0.815660689 | 5.36E-06 | 0.000245533 |
| ZBTB8B | -1.057009176 | 5.37E-06 | 0.000245533 |
| CCNL1 | -0.689897221 | 5.45E-06 | 0.000248319 |
| ENSACYG00000007129 | 3.333535918 | 6.21E-06 | 0.00028176 |
| TOP3B | -0.902007316 | 6.28E-06 | 0.000283987 |
| TPRG1L | 0.639348807 | 6.33E-06 | 0.000285114 |
| ASTE1 | -1.156726176 | 6.46E-06 | 0.000289874 |
| MOB3C | -0.91374161 | 6.63E-06 | 0.000296681 |
| AIP | -0.774463877 | 6.79E-06 | 0.000301847 |
| EDAR | 6.591255428 | 6.82E-06 | 0.000301847 |
| PPP1R3C | -0.703154457 | 6.84E-06 | 0.000301847 |
| LOC118258728 | -0.89152534 | 6.83E-06 | 0.000301847 |
| GXYLT1 | 1.239018573 | 6.95E-06 | 0.000305891 |
| MGAT3 | 0.843327761 | 7.01E-06 | 0.000307642 |
| DRAM1 | 0.862555334 | 7.05E-06 | 0.000308076 |
| CCDC141 | 3.785691695 | 7.41E-06 | 0.000323019 |
| NR4A3 | 0.921645654 | 7.48E-06 | 0.000324712 |
| UNC50 | -0.935618623 | 7.51E-06 | 0.000324905 |
| KLHL10 | 3.237019792 | 7.66E-06 | 0.000330662 |
| STK17B | -0.843655383 | 7.76E-06 | 0.000333667 |
| FBLN5 | 2.349278213 | 7.84E-06 | 0.000334977 |
| CUNH5orf51 | -2.051158434 | 7.82E-06 | 0.000334977 |
| PRPF38A | -0.750951481 | 7.96E-06 | 0.000339055 |
| BICD1 | -6.24631199 | 8.15E-06 | 0.000345801 |
| CUBN | 0.826515429 | 8.24E-06 | 0.000348843 |
| CKAP2L | -0.694732428 | 8.36E-06 | 0.000352615 |
| PIP5K1A | 0.821612502 | 8.50E-06 | 0.000356965 |
| ST6GALNAC6 | 1.050660762 | 8.52E-06 | 0.000356965 |
| ENSACYG00000001459 | 4.285340565 | 8.59E-06 | 0.00035905 |
| CACNG3 | 0.662739191 | 8.66E-06 | 0.000360688 |
| MTF1 | 0.626285751 | 8.73E-06 | 0.000362612 |
| DACT1 | -1.012491459 | 9.04E-06 | 0.000373983 |
| BBS12 | -1.026174973 | 9.09E-06 | 0.000375086 |
| ALKBH4 | -1.018771987 | 9.18E-06 | 0.000377392 |
| ABHD17B | -1.026943677 | 9.24E-06 | 0.000378772 |
| ILVBL | -0.921848386 | 9.29E-06 | 0.000379593 |
| LOC118244680 | -1.884122954 | 9.37E-06 | 0.000381822 |
| RC3H2 | 0.657731936 | 9.58E-06 | 0.000389163 |
| UTP6 | -0.662390294 | 9.62E-06 | 0.000389734 |
| TADA1 | -0.761717147 | 9.73E-06 | 0.000392811 |
| ENSACYG00000001091 | 0.610235468 | 9.95E-06 | 0.000400393 |
| LEO1 | -0.807654507 | 1.04E-05 | 0.000416162 |
| TSPAN5 | 0.798805498 | 1.04E-05 | 0.000416162 |
| ABHD17A | 0.641783554 | 1.05E-05 | 0.000417592 |
| UBAP1 | 0.690805783 | 1.08E-05 | 0.000427262 |
| TUBB4B | -0.749829853 | 1.08E-05 | 0.000427262 |
| LOC118260317 | -1.103959218 | 1.08E-05 | 0.000427262 |
| IFI30 | -0.847841895 | 1.09E-05 | 0.00043066 |
| HTR1B | 0.730787254 | 1.10E-05 | 0.00043066 |
| SIMC1 | -1.44159215 | 1.10E-05 | 0.00043066 |
| LOC118246392 | -0.647431193 | 1.11E-05 | 0.000433194 |
| HHIPL2 | 3.421815264 | 1.18E-05 | 0.000457802 |
| EIF4G2 | 0.759010403 | 1.21E-05 | 0.000468617 |
| PSTK | -0.826830832 | 1.23E-05 | 0.000477175 |
| UBC | -0.714307168 | 1.25E-05 | 0.000482114 |
| PDIA3 | -0.599629461 | 1.28E-05 | 0.000492251 |
| RGS3 | -0.880537112 | 1.28E-05 | 0.000492251 |
| CCT6A | -0.627408422 | 1.29E-05 | 0.000495474 |
| SPOUT1 | -0.778919904 | 1.34E-05 | 0.000511149 |
| HAUS2 | -1.099998664 | 1.36E-05 | 0.00051748 |
| SAXO1 | 0.742552158 | 1.46E-05 | 0.000555445 |
| ENSACYG00000001051 | 0.705497004 | 1.48E-05 | 0.000559395 |
| CASTOR1 | -0.578284777 | 1.48E-05 | 0.000559395 |
| LOC118243550 | 3.929151293 | 1.52E-05 | 0.000573201 |
| HMGCL | -0.642805757 | 1.54E-05 | 0.000578727 |
| ENSACYG00000001520 | 4.199139957 | 1.58E-05 | 0.000590053 |
| FNBP1L | -0.834132974 | 1.59E-05 | 0.000590053 |
| LOC118256259 | 0.635797664 | 1.58E-05 | 0.000590053 |
| LATS2 | 0.896620032 | 1.60E-05 | 0.000594264 |
| LOC118260046 | 0.629400592 | 1.66E-05 | 0.000612531 |
| EDC3 | -0.656028261 | 1.66E-05 | 0.000612531 |
| RHOQ | 0.578948213 | 1.68E-05 | 0.000618754 |
| WDR74 | -0.747416796 | 1.69E-05 | 0.000618785 |
| LOC118260593 | -1.017878939 | 1.73E-05 | 0.000633905 |
| ARL15 | 0.697447958 | 1.79E-05 | 0.000650581 |
| CITED2 | -0.957856486 | 1.79E-05 | 0.000650581 |
| LOC118243364 | -0.77983434 | 1.84E-05 | 0.000666724 |
| SQLE | -0.661594709 | 1.85E-05 | 0.000667579 |
| TLCD3A | 0.642613397 | 1.86E-05 | 0.000669734 |
| LOC118260291 | -1.21225064 | 1.90E-05 | 0.000684638 |
| LPAR2 | -0.79596046 | 1.91E-05 | 0.000685283 |
| MFAP5 | 0.886984896 | 1.92E-05 | 0.000687841 |
| TULP1 | 5.2464326 | 2.01E-05 | 0.000718927 |
| TRIM8 | 0.684981012 | 2.06E-05 | 0.000733171 |
| TCFL5 | 2.813858608 | 2.20E-05 | 0.000779563 |
| SMARCC2 | -1.4525534 | 2.22E-05 | 0.000784569 |
| EXOSC8 | -0.814092061 | 2.23E-05 | 0.000787331 |
| ENSACYG00000003770 | 6.24002163 | 2.24E-05 | 0.000789133 |
| ADORA2B | 1.133670946 | 2.29E-05 | 0.000804563 |
| CTSL | -0.615420546 | 2.35E-05 | 0.000819868 |
| ENSACYG00000015548 | 1.701178907 | 2.34E-05 | 0.000819868 |
| RBM41 | -0.922992465 | 2.40E-05 | 0.000834538 |
| SLC25A28 | 0.707182076 | 2.52E-05 | 0.000875643 |
| SH3BGRL2 | 0.745432379 | 2.55E-05 | 0.000880936 |
| AFAP1 | -0.572400493 | 2.55E-05 | 0.000880936 |
| GPAT4 | 0.649446633 | 2.62E-05 | 0.000904006 |
| ZBTB8OS | -0.73950974 | 2.64E-05 | 0.000905979 |
| CUNH4orf48 | 0.612001313 | 2.66E-05 | 0.000911589 |
| VAV3 | -0.834358379 | 2.69E-05 | 0.000921154 |
| LOC118251182 | -0.727897611 | 2.77E-05 | 0.000946184 |
| LOC118261260 | 0.647425261 | 2.82E-05 | 0.000960472 |
| CENPF | -0.903158775 | 2.92E-05 | 0.000990019 |
| CDC42SE2 | 0.559417497 | 2.97E-05 | 0.001002857 |
| SLC11A2 | 0.69225311 | 2.97E-05 | 0.001002857 |
| MRPS22 | -0.83780253 | 3.05E-05 | 0.001026729 |
| PABPC1L | -1.237293517 | 3.07E-05 | 0.001030584 |
| P4HB | -0.589818047 | 3.10E-05 | 0.001037307 |
| CASP8 | -0.69676408 | 3.12E-05 | 0.00104084 |
| MRPL32 | -0.787783443 | 3.12E-05 | 0.00104084 |
| ENSACYG00000016778 | 2.904743919 | 3.15E-05 | 0.001046269 |
| B4GALT2 | 0.703621922 | 3.17E-05 | 0.0010526 |
| CSNK1G2 | 0.585801781 | 3.21E-05 | 0.001061479 |
| KTN1 | -0.688062913 | 3.26E-05 | 0.001074953 |
| NLE1 | -0.664648288 | 3.29E-05 | 0.001084304 |
| ENSACYG00000004655 | 2.719901556 | 3.33E-05 | 0.00109147 |
| SUOX | -0.730016073 | 3.33E-05 | 0.00109147 |
| TRIM54 | -0.666045495 | 3.36E-05 | 0.001098928 |
| ENSACYG00000002826 | 1.444761487 | 3.47E-05 | 0.001127163 |
| AKTIP | -0.728179635 | 3.46E-05 | 0.001127163 |
| MED4 | -0.792130765 | 3.52E-05 | 0.001140435 |
| SPAG16 | -1.087482204 | 3.56E-05 | 0.0011479 |
| TBX19 | 2.188538267 | 3.55E-05 | 0.0011479 |
| LOC118243824 | 4.402458635 | 3.61E-05 | 0.001160869 |
| EXOSC4 | -0.778629228 | 3.61E-05 | 0.001160869 |
| GMCL1 | 0.717835806 | 3.71E-05 | 0.001187889 |
| SGK1 | -0.669979141 | 3.75E-05 | 0.001199365 |
| LOC118251862 | 1.189677661 | 3.80E-05 | 0.001210845 |
| FASTKD3 | -0.940703785 | 3.87E-05 | 0.001232531 |
| MRM1 | -0.730054078 | 3.96E-05 | 0.001256546 |
| ENSACYG00000010865 | 3.820112046 | 4.04E-05 | 0.00127821 |
| FBXO33 | 0.816273962 | 4.07E-05 | 0.001282187 |
| GYS2 | 6.119032688 | 4.08E-05 | 0.001282187 |
| GATAD2A | 0.64805902 | 4.07E-05 | 0.001282187 |
| TAF7L | -0.696994163 | 4.10E-05 | 0.001284964 |
| PRLHR | 3.242154591 | 4.25E-05 | 0.001328788 |
| PTPN14 | 0.69732254 | 4.26E-05 | 0.001328788 |
| YEATS4 | -0.723714833 | 4.28E-05 | 0.001332909 |
| RBM48 | -0.767928755 | 4.37E-05 | 0.001357074 |
| SIAH1 | -0.67829425 | 4.42E-05 | 0.001371309 |
| PDHA1 | -0.639898033 | 4.49E-05 | 0.001387624 |
| SLC25A29 | -0.671816274 | 4.54E-05 | 0.001401929 |
| TMCC2 | 1.096562443 | 4.62E-05 | 0.001422839 |
| LOC118261676 | -0.663635667 | 4.74E-05 | 0.001455136 |
| SERTAD2 | 0.82500163 | 4.79E-05 | 0.001465573 |
| CTSK | -0.548603151 | 4.80E-05 | 0.001465573 |
| SRPX2 | -0.593499597 | 4.85E-05 | 0.001479183 |
| PRNP | 0.601082333 | 4.95E-05 | 0.001505415 |
| ACAT1 | -0.601844436 | 5.00E-05 | 0.001518127 |
| YBX3 | 0.619708905 | 5.07E-05 | 0.001534643 |
| CH25H | 0.890155256 | 5.09E-05 | 0.001538397 |
| LOC118251550 | 0.568970601 | 5.19E-05 | 0.001565289 |
| ME1 | -1.086252686 | 5.22E-05 | 0.001567598 |
| DNASE1L3 | 3.729830923 | 5.21E-05 | 0.001567598 |
| HMOX2 | -0.567698996 | 5.26E-05 | 0.001568268 |
| TNIP2 | -0.968879158 | 5.25E-05 | 0.001568268 |
| ENSACYG00000016081 | 1.364479016 | 5.25E-05 | 0.001568268 |
| HIPK1 | 0.649530202 | 5.35E-05 | 0.001589317 |
| C1QTNF12 | 0.602091543 | 5.46E-05 | 0.001620112 |
| TNFSF13B | 1.953200693 | 5.55E-05 | 0.001643805 |
| TGIF1 | -1.219993779 | 5.58E-05 | 0.001647921 |
| ENSACYG00000009966 | 0.802272977 | 5.69E-05 | 0.001673225 |
| MTHFR | 0.581370297 | 5.70E-05 | 0.001673225 |
| CXCR5 | 2.944047405 | 5.70E-05 | 0.001673225 |
| SLC5A7 | 0.812560708 | 5.73E-05 | 0.001678367 |
| LTO1 | -0.713370347 | 5.79E-05 | 0.001691729 |
| NAE1 | -0.639777317 | 5.84E-05 | 0.001700248 |
| ENSACYG00000004268 | 5.592818313 | 5.85E-05 | 0.001700248 |
| TIAL1 | -0.578547186 | 5.89E-05 | 0.001708504 |
| ENSACYG00000006242 | 0.87694933 | 5.96E-05 | 0.001725019 |
| DMP1 | 3.878746965 | 5.99E-05 | 0.001725345 |
| ENSACYG00000001849 | 0.67548638 | 5.98E-05 | 0.001725345 |
| CNOT7 | 0.533277038 | 6.05E-05 | 0.001741492 |
| XRCC5 | -0.779701162 | 6.11E-05 | 0.001745614 |
| MEOX2 | -0.667092465 | 6.09E-05 | 0.001745614 |
| PLEKHF1 | -0.833526908 | 6.10E-05 | 0.001745614 |
| HPSE | -0.655735253 | 6.20E-05 | 0.001763552 |
| ZWILCH | -0.633641914 | 6.19E-05 | 0.001763552 |
| ANXA6 | -0.830187903 | 6.27E-05 | 0.001781058 |
| CUNH1orf109 | -0.791454761 | 6.43E-05 | 0.001818747 |
| NBN | -0.627401018 | 6.42E-05 | 0.001818747 |
| RFC4 | -0.777393905 | 6.49E-05 | 0.00182963 |
| GLMN | -0.671503926 | 6.54E-05 | 0.001840245 |
| SPTBN1 | -0.666939988 | 6.61E-05 | 0.001855494 |
| HDX | -0.75549689 | 6.66E-05 | 0.001865703 |
| TAX1BP3 | 0.597955322 | 6.73E-05 | 0.001882062 |
| PRTG | 2.054020524 | 6.79E-05 | 0.00189384 |
| URB1 | -0.879792809 | 6.83E-05 | 0.0019015 |
| LOC118261526 | 5.611832357 | 6.97E-05 | 0.00193449 |
| RBM7 | -0.721068336 | 6.96E-05 | 0.00193449 |
| BMPR2 | 0.593182784 | 7.02E-05 | 0.001942458 |
| AFF2 | 0.772664967 | 7.03E-05 | 0.001943 |
| CSTF1 | -0.671833591 | 7.38E-05 | 0.002034669 |
| EIF2B1 | -0.610728526 | 7.43E-05 | 0.002043274 |
| CDC37L1 | -0.70954265 | 7.49E-05 | 0.002050969 |
| LOC118259344 | -0.738636485 | 7.49E-05 | 0.002050969 |
| SCG3 | -0.739415535 | 7.74E-05 | 0.002114141 |
| ITGB1BP1 | -0.706772174 | 7.75E-05 | 0.002114141 |
| DUSP10 | -0.624322488 | 7.82E-05 | 0.002127907 |
| IGFBP7 | -0.620687373 | 7.94E-05 | 0.002152602 |
| MBNL2 | 0.613201587 | 7.93E-05 | 0.002152602 |
| THNSL2 | -0.799469252 | 8.10E-05 | 0.002187187 |
| FGF9 | 0.713587955 | 8.11E-05 | 0.002187187 |
| ZCCHC2 | 0.735851479 | 8.12E-05 | 0.002187187 |
| LOC118257380 | -0.737061011 | 8.22E-05 | 0.002210262 |
| DYSF | -0.682084862 | 8.40E-05 | 0.002253429 |
| CFL2 | 0.591784502 | 8.42E-05 | 0.002253993 |
| LOC118245549 | -1.902091725 | 8.46E-05 | 0.002261611 |
| KAT6B | -0.83634461 | 8.52E-05 | 0.00227203 |
| EAPP | -0.66397705 | 9.16E-05 | 0.00243941 |
| SC5D | 0.563872208 | 9.34E-05 | 0.00247619 |
| LOC118257919 | 5.984608151 | 9.33E-05 | 0.00247619 |
| GMNN | -0.749577467 | 9.37E-05 | 0.002478834 |
| NIM1K | 2.36467093 | 9.56E-05 | 0.002523568 |
| EXOC1 | -0.697504066 | 9.69E-05 | 0.002552201 |
| CREB3L1 | 0.966071903 | 9.74E-05 | 0.0025622 |
| ABCG2 | 3.340175697 | 9.79E-05 | 0.002563897 |
| MGLL | 0.844067336 | 9.77E-05 | 0.002563897 |
| NATD1 | 0.8860576 | 0.000100296 | 0.002621983 |
| TPP1 | -0.548053161 | 0.000101491 | 0.002647986 |
| ENSACYG00000013245 | 1.179075791 | 0.000102011 | 0.002656305 |
| MICU1 | -0.538650788 | 0.000102669 | 0.002668188 |
| UST | 0.571820074 | 0.0001035 | 0.0026845 |
| ENSACYG00000017148 | 5.518920504 | 0.000104369 | 0.002701726 |
| NEB | 1.703342533 | 0.000105386 | 0.002722725 |
| FN1 | -0.595774231 | 0.000106564 | 0.002743118 |
| EPRS1 | -0.575987506 | 0.000106591 | 0.002743118 |
| CDK7 | -0.643056931 | 0.000108668 | 0.002785691 |
| LOC118252592 | -0.724772032 | 0.000108584 | 0.002785691 |
| THAP12 | -0.972231953 | 0.000109727 | 0.002807391 |
| ENSACYG00000000083 | 2.507231261 | 0.000111024 | 0.002824153 |
| ST6GALNAC2 | -0.763206321 | 0.000110907 | 0.002824153 |
| LOC118260034 | -0.613440361 | 0.000110705 | 0.002824153 |
| DMAP1 | -0.789695465 | 0.000111467 | 0.002829966 |
| PMS1 | -0.675454978 | 0.000111886 | 0.002833929 |
| MTFR2 | -0.775296021 | 0.000112052 | 0.002833929 |
| RAD17 | -0.730587217 | 0.000112441 | 0.002838331 |
| SELE | 5.597246114 | 0.000113009 | 0.00284647 |
| APPBP2 | -0.653314028 | 0.000113626 | 0.00284647 |
| NHP2 | -0.739106678 | 0.000113566 | 0.00284647 |
| LOC118248865 | 5.908234277 | 0.000113595 | 0.00284647 |
| LOC118255061 | 0.751254632 | 0.000114581 | 0.002864947 |
| LOC118255340 | 0.879670797 | 0.000115972 | 0.00289425 |
| LOC118257056 | -0.985508954 | 0.000116243 | 0.002895539 |
| COBL | -1.240212151 | 0.000117528 | 0.002922031 |
| PRRG3 | 1.101662913 | 0.000117924 | 0.002926385 |
| RNF19B | 1.238824155 | 0.000118242 | 0.002928754 |
| TTI2 | -0.684693483 | 0.000118629 | 0.002932837 |
| MRPL46 | -0.656996806 | 0.000119715 | 0.002948646 |
| CCDC88A | -0.774857391 | 0.000119672 | 0.002948646 |
| ENSACYG00000007688 | -0.906931227 | 0.000120488 | 0.00296135 |
| RNF144A | 0.686880761 | 0.000120679 | 0.00296135 |
| TNK2 | -0.95366246 | 0.00012202 | 0.002977886 |
| CCNI | 0.589774562 | 0.000121795 | 0.002977886 |
| SERPINE2 | -0.576148852 | 0.00012203 | 0.002977886 |
| DNAJC16 | 0.676890118 | 0.000122394 | 0.002981253 |
| EVL | 0.57695267 | 0.000123545 | 0.003003761 |
| LOC118245354 | 0.70765823 | 0.000124548 | 0.003022583 |
| SMC2 | -0.639482295 | 0.000124956 | 0.003026908 |
| ENSACYG00000016847 | -0.88102821 | 0.000127091 | 0.003072993 |
| TSC22D3 | -0.791497373 | 0.000128772 | 0.00310794 |
| HIVEP2 | 0.862449358 | 0.000130455 | 0.003142812 |
| RAI2 | 0.94259211 | 0.000131731 | 0.003162502 |
| DNAJC5G | -0.769697821 | 0.000131751 | 0.003162502 |
| TUFT1 | -0.520938296 | 0.00013316 | 0.003190523 |
| PRPF4B | -0.664211731 | 0.000134341 | 0.00321298 |
| RBM17 | -0.592022159 | 0.00013806 | 0.003290016 |
| CSPG4 | -0.830825573 | 0.000137969 | 0.003290016 |
| AP5Z1 | -0.81313534 | 0.000141518 | 0.003364964 |
| HINFP | -0.868855841 | 0.000141715 | 0.003364964 |
| EFNA1 | -1.172593947 | 0.000144662 | 0.003428764 |
| CTSH | -0.516459065 | 0.000145272 | 0.00343707 |
| CUNH2orf50 | 1.766997217 | 0.000147998 | 0.003495292 |
| COL6A3 | -0.597345467 | 0.000150553 | 0.003549291 |
| SAMSN1 | 5.885751251 | 0.000151833 | 0.003573086 |
| RPUSD1 | 0.675760717 | 0.00015385 | 0.003614113 |
| OLFM1 | 0.821055133 | 0.000155856 | 0.003654724 |
| ZFYVE19 | -1.039237684 | 0.00015739 | 0.003684144 |
| CPE | -0.882418583 | 0.000160837 | 0.003758186 |
| ENSACYG00000006869 | 0.636510763 | 0.000161743 | 0.003770653 |
| VPS33A | -0.587689777 | 0.000162166 | 0.003770653 |
| DCAF5 | 0.636605671 | 0.000162228 | 0.003770653 |
| SRGAP2 | 0.63532288 | 0.000162647 | 0.003773749 |
| MAPKAP1 | 0.584317188 | 0.000163005 | 0.003775434 |
| LOC118247959 | -0.645008026 | 0.000163797 | 0.003787128 |
| HSPD1 | -0.643147168 | 0.000164704 | 0.003788197 |
| VASH2 | 0.870254188 | 0.000164162 | 0.003788197 |
| SLC30A4 | 0.796619981 | 0.000164458 | 0.003788197 |
| CTNS | -0.629780709 | 0.000167721 | 0.003844179 |
| LOC118245892 | -0.90387024 | 0.000167437 | 0.003844179 |
| RBBP6 | 0.580041551 | 0.0001683 | 0.003850784 |
| VLDLR | -0.733206222 | 0.000170112 | 0.003885499 |
| NUP93 | -0.597411287 | 0.000172104 | 0.00392422 |
| ENSACYG00000008373 | 1.541268004 | 0.000173018 | 0.003938239 |
| NSUN5 | -0.563780703 | 0.000174857 | 0.003973251 |
| NHEJ1 | -2.071973714 | 0.000175514 | 0.003981344 |
| LMO7 | -0.613524128 | 0.000176682 | 0.004000953 |
| ST3GAL1 | 0.551945686 | 0.000177129 | 0.004004216 |
| IRF8 | 1.097135429 | 0.00018108 | 0.004086516 |
| LOC118255699 | -0.957917994 | 0.000182537 | 0.004112384 |
| PIAS2 | 0.553188229 | 0.000185508 | 0.004172187 |
| GPR162 | 0.627988785 | 0.000192714 | 0.004319546 |
| ADHFE1 | 0.54380821 | 0.000192623 | 0.004319546 |
| DCSTAMP | -0.789818473 | 0.000193834 | 0.004329933 |
| R3HDM2 | 0.695059567 | 0.000193599 | 0.004329933 |
| MKKS | 0.655008318 | 0.000195651 | 0.004363156 |
| SELENOO | -0.699183709 | 0.000197611 | 0.004392019 |
| FAM219A | 0.622580401 | 0.000197474 | 0.004392019 |
| CBX8 | -0.72563813 | 0.000198602 | 0.00440663 |
| TPX2 | -0.549395031 | 0.000199026 | 0.004408616 |
| EIF3A | -0.599512739 | 0.000200348 | 0.004415682 |
| RCHY1 | -0.542336754 | 0.000200146 | 0.004415682 |
| LOC118259472 | -0.658960942 | 0.000199733 | 0.004415682 |
| ENSACYG00000003165 | 3.879709475 | 0.000203477 | 0.004477168 |
| LOC118244561 | -0.622431248 | 0.000205107 | 0.00450553 |
| YRDC | -0.645616105 | 0.000205525 | 0.00450721 |
| CCNI2 | 0.528302822 | 0.000206676 | 0.004524946 |
| CD40 | -0.836317779 | 0.000210521 | 0.004580056 |
| KATNA1 | -0.695454035 | 0.000210581 | 0.004580056 |
| MYO5B | 2.540875821 | 0.000210439 | 0.004580056 |
| CEP44 | -0.678148928 | 0.000210297 | 0.004580056 |
| LOC118253960 | 0.743146197 | 0.000213032 | 0.00462573 |
| CCDC71L | -0.882369778 | 0.00021484 | 0.004657341 |
| CNOT4 | 0.688111014 | 0.000220238 | 0.004758719 |
| FER1L6 | 5.820352293 | 0.000220178 | 0.004758719 |
| SGMS2 | 0.504829249 | 0.0002223 | 0.004795426 |
| LRRC8B | 0.772661574 | 0.000223712 | 0.004818027 |
| ARHGAP10 | -0.619996 | 0.000224295 | 0.004822712 |
| TARDBP | -0.573285548 | 0.000228692 | 0.004909245 |
| ENSACYG00000005007 | 0.702254525 | 0.00023031 | 0.004935969 |
| DKK2 | 0.670476995 | 0.000232623 | 0.004977453 |
| TP53RK | -0.549521516 | 0.000237529 | 0.005074209 |
| WDR75 | -0.65746501 | 0.000240253 | 0.005116842 |
| HGH1 | -0.638105424 | 0.0002403 | 0.005116842 |
| DNAJC17 | -0.719049863 | 0.000244157 | 0.005182255 |
| VCPKMT | -0.928032686 | 0.000244139 | 0.005182255 |
| UVRAG | 0.664236354 | 0.000245018 | 0.005192181 |
| HEXD | -0.886142631 | 0.00024669 | 0.005219236 |
| LOC118260553 | -0.731454454 | 0.000250034 | 0.005281511 |
| ZDHHC3 | 0.613908155 | 0.000254508 | 0.005367428 |
| POLD2 | -0.857049806 | 0.000256891 | 0.005409056 |
| PAQR3 | -0.570088623 | 0.000258485 | 0.005433951 |
| SPRING1 | 0.61311135 | 0.000259647 | 0.005447518 |
| CUNH12orf4 | -0.605798487 | 0.000259956 | 0.005447518 |
| CCT8 | -0.527754931 | 0.000261607 | 0.005456143 |
| POMT2 | 0.809922252 | 0.000260913 | 0.005456143 |
| LOC118245314 | -0.663070951 | 0.000261267 | 0.005456143 |
| FGFR2 | 0.719237755 | 0.000262556 | 0.005467303 |
| ENSACYG00000000051 | 5.772230898 | 0.000265281 | 0.005515339 |
| MMP2 | -0.501179174 | 0.000265892 | 0.005519339 |
| ENSACYG00000000843 | 5.778008077 | 0.000268735 | 0.005569614 |
| ETFBKMT | -0.898468648 | 0.000269825 | 0.005583436 |
| ADGRL3 | 1.161291356 | 0.000271476 | 0.005608799 |
| CMTM7 | 0.620172761 | 0.000271953 | 0.005609877 |
| CHM | -0.623657836 | 0.000273145 | 0.005612798 |
| TRAPPC12 | -0.559824352 | 0.00027337 | 0.005612798 |
| RGP1 | -0.646164538 | 0.000273016 | 0.005612798 |
| ENSACYG00000005978 | 1.054726806 | 0.000275269 | 0.005643021 |
| LOC118257862 | -0.950630422 | 0.000277579 | 0.005681552 |
| PLK2 | -0.656400813 | 0.000281318 | 0.00574916 |
| LOC118253380 | -0.559095753 | 0.000282307 | 0.005760468 |
| DIABLO | -0.5933467 | 0.000284952 | 0.005805455 |
| MED16 | 0.834878775 | 0.000290106 | 0.005901351 |
| DDX28 | -0.730702393 | 0.000292523 | 0.00594137 |
| RPP38 | -0.686381277 | 0.000293524 | 0.005952538 |
| ENSACYG00000005870 | 2.992632605 | 0.000294949 | 0.005972257 |
| MED7 | -0.640270971 | 0.000301022 | 0.006085898 |
| TMEM179B | -0.744091919 | 0.000301573 | 0.006087715 |
| LRRC8C | -0.580223408 | 0.000302848 | 0.006094814 |
| MEX3C | 0.603058416 | 0.000302431 | 0.006094814 |
| MGAT4B | 0.68868942 | 0.000303526 | 0.006099161 |
| SAXO2 | 1.266025727 | 0.000304781 | 0.006115078 |
| AGAP3 | 0.80653259 | 0.000306558 | 0.0061414 |
| MRPL19 | -0.616188982 | 0.0003079 | 0.006156576 |
| BRD8 | -0.811009661 | 0.000309181 | 0.006156576 |
| FAM110B | -0.747571492 | 0.000308854 | 0.006156576 |
| SLC22A23 | 0.73506827 | 0.000308256 | 0.006156576 |
| TLN2 | -0.992446217 | 0.000310634 | 0.006176196 |
| TTC26 | -0.643507394 | 0.000312289 | 0.00619975 |
| USP10 | 0.533451228 | 0.000313102 | 0.006206559 |
| DUS2 | -0.721012095 | 0.000314386 | 0.00622268 |
| PTP4A1 | 0.569313466 | 0.000314863 | 0.006222791 |
| EPB41L2 | -0.501603675 | 0.000318483 | 0.00627554 |
| LOC118246781 | 5.776775137 | 0.000318334 | 0.00627554 |
| TAF8 | -0.963834821 | 0.00031923 | 0.006280877 |
| ENSACYG00000006528 | 0.90289057 | 0.00032113 | 0.006308864 |
| ZFAND2A | -0.629020958 | 0.000323589 | 0.006347725 |
| IL22RA2 | 4.839302396 | 0.000324174 | 0.006349772 |
| ENSACYG00000000669 | 0.842371322 | 0.000327814 | 0.006377222 |
| YTHDF1 | 0.614150659 | 0.000327093 | 0.006377222 |
| TACC2 | 0.676751115 | 0.000327991 | 0.006377222 |
| ENSACYG00000007530 | 1.693949073 | 0.000327484 | 0.006377222 |
| PACSIN2 | -0.569326851 | 0.000327919 | 0.006377222 |
| CCNY | 0.65945913 | 0.000330686 | 0.006410735 |
| ENSACYG00000004254 | 4.656301664 | 0.000330382 | 0.006410735 |
| SORBS2 | 0.856595673 | 0.00033225 | 0.006431617 |
| CD276 | -0.509810014 | 0.000335399 | 0.006483063 |
| ENSACYG00000012635 | 3.835716589 | 0.00033899 | 0.0065429 |
| SIL1 | -0.593661368 | 0.000342366 | 0.006598419 |
| KPNA2 | -0.55194254 | 0.000348354 | 0.00670403 |
| MADCAM1 | 5.679546605 | 0.0003491 | 0.006708621 |
| CUNH16orf70 | -0.621908596 | 0.000350395 | 0.006723705 |
| ZNF704 | 0.539386973 | 0.000356061 | 0.006812631 |
| LOC118244782 | 1.350402883 | 0.000355623 | 0.006812631 |
| NRBP2 | 0.756186724 | 0.000359344 | 0.006865493 |
| NFATC3 | 0.76736878 | 0.000360116 | 0.006870308 |
| PRDM1 | 0.839179524 | 0.000363688 | 0.006928434 |
| AKAP17A | -0.601832071 | 0.000367312 | 0.006987394 |
| FNDC11 | 5.235161218 | 0.000368992 | 0.007009252 |
| PDXP | -0.586272644 | 0.000370585 | 0.007029393 |
| BTBD7 | 0.544208675 | 0.000372859 | 0.007062383 |
| SYT16 | 0.906559726 | 0.000373955 | 0.007072995 |
| MID1IP1 | -0.784694049 | 0.000377307 | 0.007116016 |
| GPX3 | 0.577599118 | 0.000376899 | 0.007116016 |
| LOC118258430 | 4.065431761 | 0.000383978 | 0.007231495 |
| RGCC | -0.632435626 | 0.000386401 | 0.007266767 |
| LOC118254681 | -0.768268523 | 0.000390437 | 0.007325653 |
| FGF18 | 2.381351024 | 0.000390642 | 0.007325653 |
| SEPTIN11 | -0.608342261 | 0.000394916 | 0.007384809 |
| MASP2 | 5.199900169 | 0.000394915 | 0.007384809 |
| PCDH17 | 0.954321114 | 0.00039724 | 0.007417764 |
| LOC118260312 | 5.203158461 | 0.000399756 | 0.007443698 |
| DDX6 | -0.553632071 | 0.000399715 | 0.007443698 |
| PARP9 | 0.525102535 | 0.000401563 | 0.007466813 |
| ST7L | -0.524725161 | 0.000403359 | 0.00748966 |
| VCL | -0.592090524 | 0.000405717 | 0.00752286 |
| CTSS | -0.55820435 | 0.000408737 | 0.007568226 |
| LOC118247783 | 5.654160177 | 0.000422103 | 0.007802605 |
| TTL | 0.628632417 | 0.000422624 | 0.007802605 |
| KCTD2 | 0.775944718 | 0.000423168 | 0.007802605 |
| BCL2 | 0.667750455 | 0.000425706 | 0.007838461 |
| FAM177A1 | 0.612177447 | 0.000427783 | 0.007854792 |
| ZNF639 | -0.64570522 | 0.000427355 | 0.007854792 |
| ARHGAP35 | 0.845325755 | 0.000430416 | 0.007892163 |
| ZNF830 | -0.656790483 | 0.000431416 | 0.007899514 |
| LOC118245664 | 2.209559364 | 0.000434631 | 0.007947371 |
| SMARCA1 | -0.576155501 | 0.000436525 | 0.007970961 |
| TFPI2 | -1.429678714 | 0.000443886 | 0.008079899 |
| ENSACYG00000016016 | 1.130203763 | 0.000444327 | 0.008079899 |
| PREB | -0.673567571 | 0.000443869 | 0.008079899 |
| B3GALNT2 | 0.589028198 | 0.000445542 | 0.008090853 |
| GABRA4 | 1.048107023 | 0.000449262 | 0.00814719 |
| SELPLG | 5.173983799 | 0.000450512 | 0.008158658 |
| NFATC1 | 0.931054709 | 0.000453302 | 0.008164375 |
| ENSACYG00000008594 | -0.75606771 | 0.000452871 | 0.008164375 |
| ENSACYG00000013730 | -0.76390651 | 0.000452018 | 0.008164375 |
| SLC41A2 | 0.602648263 | 0.000453107 | 0.008164375 |
| SYNE2 | -0.788656795 | 0.000454179 | 0.008169031 |
| FGF2 | 0.680438599 | 0.000455427 | 0.008180341 |
| DAGLB | -0.727731815 | 0.000457691 | 0.008201662 |
| FKBP11 | -0.740290486 | 0.000457857 | 0.008201662 |
| ENSACYG00000005318 | -0.59477981 | 0.000459125 | 0.008202126 |
| ENSACYG00000016331 | 1.027512875 | 0.000458986 | 0.008202126 |
| LOC118253067 | -0.713108063 | 0.000461041 | 0.00822522 |
| KCNE1 | 0.92129349 | 0.000462688 | 0.008243464 |
| BNIP1 | -0.675096294 | 0.000466025 | 0.00829172 |
| TMEM204 | 0.777194178 | 0.000472314 | 0.008392318 |
| TUBE1 | -0.648609245 | 0.000479402 | 0.008502184 |
| LOC118258757 | -0.503979527 | 0.000479785 | 0.008502184 |
| LOC118256212 | -0.937457433 | 0.000482584 | 0.008540309 |
| N6AMT1 | -0.678110018 | 0.000486029 | 0.008566833 |
| NFATC2 | 0.566545357 | 0.000485464 | 0.008566833 |
| ZNF652 | 0.616182332 | 0.000485927 | 0.008566833 |
| FOXL1 | 2.406656603 | 0.00048699 | 0.008572318 |
| CCT2 | -0.492202942 | 0.000489351 | 0.008602414 |
| ABHD5 | 0.661745077 | 0.000491541 | 0.008628426 |
| MAP3K3 | 0.957858308 | 0.000492138 | 0.008628426 |
| ENSACYG00000009513 | 2.369370458 | 0.00049369 | 0.008644154 |
| PTHLH | -0.600689125 | 0.000498894 | 0.008723709 |
| CENPE | -0.617083453 | 0.000500922 | 0.008747584 |
| ZDHHC14 | 0.657521861 | 0.000509812 | 0.008891074 |
| LIF | 0.60990564 | 0.000512633 | 0.008928464 |
| AIF1L | 0.550259681 | 0.000514143 | 0.00894297 |
| ENSACYG00000010664 | 1.258774035 | 0.000515093 | 0.008947708 |
| ALAS1 | -0.476250252 | 0.000518537 | 0.008995696 |
| MBNL1 | 0.659250331 | 0.000520443 | 0.009016918 |
| CUNH1orf52 | -0.846559181 | 0.000522597 | 0.009042366 |
| ENSACYG00000005373 | 3.581259164 | 0.000529613 | 0.009151767 |
| SLC35A1 | -0.670964802 | 0.000530971 | 0.009163239 |
| ENSACYG00000009389 | 1.546091427 | 0.000534411 | 0.009210575 |
| ZDHHC7 | 0.712308611 | 0.000537849 | 0.00925241 |
| NUP85 | -0.491680743 | 0.00053824 | 0.00925241 |
| LOC118256438 | 1.172131077 | 0.000539666 | 0.00926485 |
| LOC118245864 | 0.661480596 | 0.000540629 | 0.009269329 |
| LOC118251023 | -0.716803775 | 0.000542758 | 0.00929376 |
| ENSACYG00000002908 | 0.746756415 | 0.000551312 | 0.009428009 |
| ACAT2 | -0.506408977 | 0.000557209 | 0.00951652 |
| ZNF335 | 0.92183911 | 0.000558638 | 0.009528605 |
| XRN2 | -0.477634031 | 0.000562911 | 0.009589095 |
| CD109 | -0.767313591 | 0.000566177 | 0.009632313 |
| SCLT1 | -0.61699844 | 0.000569993 | 0.009647491 |
| TMEM267 | 0.492118789 | 0.000569855 | 0.009647491 |
| EFNB1 | 0.492338961 | 0.000569923 | 0.009647491 |
| CEP57 | -0.756222002 | 0.000567845 | 0.009647491 |
| CHRAC1 | -0.609089037 | 0.000573148 | 0.009688477 |
| LRFN5 | 0.695952215 | 0.00057451 | 0.009699072 |
| ATXN1L | 0.763694849 | 0.000575362 | 0.009701067 |
| ZNF831 | 2.150257985 | 0.000577836 | 0.009717949 |
| GFPT2 | -0.494712326 | 0.000577442 | 0.009717949 |
| TMEM18 | -0.695480515 | 0.000579466 | 0.009732964 |
| TAB3 | 0.684976409 | 0.000580458 | 0.009737234 |
| GPBP1L1 | 0.623641168 | 0.000587781 | 0.009847573 |
| RBM5 | -0.473853804 | 0.000592898 | 0.00992071 |
| TMLHE | 0.724444954 | 0.000596125 | 0.009940215 |
| N4BP2L1 | 0.603220692 | 0.000596672 | 0.009940215 |
| KDM4C | -0.57490819 | 0.000596244 | 0.009940215 |
| LOC118257087 | -0.755487387 | 0.000597075 | 0.009940215 |
| BRF2 | -1.021685557 | 0.000604226 | 0.010046586 |
| KIF11 | -0.750814842 | 0.000605979 | 0.010063056 |
| LPAR3 | 5.083463792 | 0.000607502 | 0.01006589 |
| ENSACYG00000010040 | 0.603164788 | 0.000607674 | 0.01006589 |
| LOC118243356 | 0.484433935 | 0.00061225 | 0.01012897 |
| ATF3 | 0.49851386 | 0.000614149 | 0.010147683 |
| LCLAT1 | 0.675573694 | 0.000617112 | 0.010183895 |
| PUM3 | -0.510464519 | 0.000620772 | 0.010231492 |
| ANKRD33B | 5.122353913 | 0.000621882 | 0.010237015 |
| ARL5A | 0.612396682 | 0.000624692 | 0.010267111 |
| ENSACYG00000010064 | -3.820381332 | 0.000625266 | 0.010267111 |
| UCHL5 | -0.596297333 | 0.000627667 | 0.010293733 |
| AOPEP | -0.579910339 | 0.000647921 | 0.010612728 |
| PDP2 | -0.564386037 | 0.000655416 | 0.010691636 |
| CKAP5 | -0.766863296 | 0.000653654 | 0.010691636 |
| DUS1L | -0.654241069 | 0.000654987 | 0.010691636 |
| ENSACYG00000015618 | 0.524870566 | 0.000655978 | 0.010691636 |
| GHITM | -0.502245167 | 0.000658614 | 0.010696381 |
| CPSF4 | 0.468420408 | 0.0006587 | 0.010696381 |
| PLTP | -0.597414933 | 0.000657351 | 0.010696381 |
| ENSACYG00000002600 | 0.709938822 | 0.000659913 | 0.010702914 |
| ARPC4 | -0.619120412 | 0.000662819 | 0.010736856 |
| SLC35F3 | 0.747785479 | 0.000667604 | 0.010798368 |
| ZNF319 | 0.676735298 | 0.000668252 | 0.010798368 |
| ENSACYG00000008946 | 3.916204769 | 0.000670417 | 0.010820099 |
| STXBP6 | 0.640294903 | 0.000676826 | 0.010905322 |
| PAPSS1 | -0.491892726 | 0.000678175 | 0.010905322 |
| PPM1B | 0.545630533 | 0.000677875 | 0.010905322 |
| RAP2C | 0.594723583 | 0.0006837 | 0.010980795 |
| LOC118244824 | -0.627743491 | 0.000688858 | 0.011050188 |
| ENSACYG00000005176 | 1.284448644 | 0.000692444 | 0.011088593 |
| SPIN1 | 0.553304712 | 0.000692932 | 0.011088593 |
| XRCC3 | -0.843233887 | 0.000694569 | 0.011101334 |
| GOLGB1 | -0.499446032 | 0.000696388 | 0.011116953 |
| LOC118251874 | -1.123149196 | 0.000701251 | 0.011167566 |
| NSUN6 | -0.816291548 | 0.000701137 | 0.011167566 |
| TSEN15 | -0.755088385 | 0.000708996 | 0.011213287 |
| LOC118253620 | -0.57926497 | 0.000709854 | 0.011213287 |
| IMPACT | -0.650103695 | 0.000709841 | 0.011213287 |
| CKS2 | -0.710049265 | 0.000710067 | 0.011213287 |
| FNDC7 | 3.336027606 | 0.00070799 | 0.011213287 |
| PUF60 | -0.484323335 | 0.000708644 | 0.011213287 |
| SLC25A25 | 0.736566825 | 0.000707133 | 0.011213287 |
| BBS2 | -0.49351549 | 0.000711661 | 0.011225035 |
| ENSACYG00000000170 | 2.22482295 | 0.000713079 | 0.011233967 |
| MOGS | -0.837110025 | 0.00071411 | 0.0112368 |
| TMEM38A | 0.481726002 | 0.000716442 | 0.011260088 |
| ENSACYG00000006103 | 0.598582186 | 0.000721454 | 0.011325365 |
| AGO2 | 0.719022839 | 0.000724501 | 0.011359689 |
| LTN1 | -0.643833835 | 0.00072903 | 0.011390116 |
| DCBLD2 | 0.573639242 | 0.000728878 | 0.011390116 |
| NAB1 | 0.524826775 | 0.000727623 | 0.011390116 |
| LRMDA | 0.518551671 | 0.000732073 | 0.011410661 |
| STAMBP | -0.509107763 | 0.000731393 | 0.011410661 |
| POLR1A | -0.646504484 | 0.000733382 | 0.011417582 |
| ENSACYG00000012047 | 1.077486031 | 0.000736817 | 0.011457554 |
| LOC118248965 | -0.880618936 | 0.000741826 | 0.011521868 |
| ENSACYG00000009906 | 0.520792927 | 0.000744647 | 0.011538526 |
| ATG13 | 0.813391215 | 0.000744505 | 0.011538526 |
| ADAMTS12 | 0.632278538 | 0.000748902 | 0.011590862 |
| P2RX5 | 1.035284162 | 0.000756098 | 0.011674866 |
| LOC118254393 | -0.519453812 | 0.000755469 | 0.011674866 |
| WDR45B | 0.518845345 | 0.000759085 | 0.011680046 |
| TMPRSS2 | 5.508750093 | 0.000759088 | 0.011680046 |
| SRGN | -0.671606505 | 0.000758148 | 0.011680046 |
| IFT57 | -0.652876569 | 0.000760512 | 0.011688342 |
| RNF186 | 0.733213474 | 0.000774374 | 0.011887546 |
| ARL4C | -0.561192747 | 0.000781558 | 0.011983892 |
| PCGF6 | -0.827508534 | 0.000786433 | 0.012044652 |
| ENSACYG00000001336 | 2.12251559 | 0.000788312 | 0.012059441 |
| PTCHD1 | 0.794074802 | 0.000790331 | 0.012076334 |
| HADHB | -0.491719316 | 0.000792997 | 0.012103065 |
| SLC52A3 | -0.676138346 | 0.000798048 | 0.012166082 |
| SPTB | -0.756331152 | 0.000801006 | 0.012193926 |
| MYBL2 | 0.577852129 | 0.000801722 | 0.012193926 |
| DUSP6 | -0.501921963 | 0.000802857 | 0.012196517 |
| NAA25 | -0.479250428 | 0.00080374 | 0.012196517 |
| SPSB3 | -0.583815187 | 0.000805488 | 0.012209008 |
| ENSACYG00000007059 | 0.97796631 | 0.000809025 | 0.012234538 |
| LOC118256771 | 4.467989192 | 0.000808737 | 0.012234538 |
| SLC40A1 | 0.667891463 | 0.000809987 | 0.012235061 |
| ENTPD5 | 0.758033975 | 0.000816986 | 0.012312606 |
| PHYHD1 | 1.245231725 | 0.00081686 | 0.012312606 |
| ABCB6 | -0.836699141 | 0.000820622 | 0.012325199 |
| ALS2CL | 0.741303145 | 0.000820136 | 0.012325199 |
| ENSACYG00000010518 | 2.151393964 | 0.000820398 | 0.012325199 |
| CTSZ | -0.556152111 | 0.000824968 | 0.012370616 |
| ANKRD49 | -0.5990997 | 0.00082552 | 0.012370616 |
| CLK4 | -0.60319734 | 0.000830626 | 0.012433025 |
| MRM3 | -0.632910747 | 0.000834839 | 0.012481931 |
| PRMT7 | -0.676757388 | 0.000837561 | 0.012508459 |
| RASA2 | -0.844794286 | 0.000845194 | 0.012608186 |
| CTHRC1 | -0.56258917 | 0.000846318 | 0.012610705 |
| HIPK3 | 0.622136184 | 0.000848031 | 0.012621984 |
| LOC118255547 | 4.451382364 | 0.000851259 | 0.012640107 |
| CUNH3orf14 | 0.515832996 | 0.000852121 | 0.012640107 |
| RPS6 | -0.542642917 | 0.000852064 | 0.012640107 |
| TTYH2 | 0.652348178 | 0.000856491 | 0.012689662 |
| GEMIN5 | -0.607732742 | 0.000857384 | 0.012689662 |
| UBE4A | 0.788083583 | 0.000859732 | 0.012710171 |
| TARBP1 | -0.782679386 | 0.000860831 | 0.012712186 |
| PRIM1 | -0.600396755 | 0.000880102 | 0.012982249 |
| ID4 | -0.769011704 | 0.000881702 | 0.012991322 |
| RPF1 | -0.673092795 | 0.000888935 | 0.013068733 |
| VGLL3 | 0.486049441 | 0.000888232 | 0.013068733 |
| SRPX | -0.617177989 | 0.000891572 | 0.013092751 |
| FBXW5 | 0.811155102 | 0.000892552 | 0.013092751 |
| VPS37D | -0.734062875 | 0.000901776 | 0.013213366 |
| PLD6 | 5.023146149 | 0.000904609 | 0.013240179 |
| STS | 0.601245405 | 0.000905644 | 0.01324066 |
| ENSACYG00000012501 | 2.384479048 | 0.000908498 | 0.013253026 |
| CFAP92 | 4.961397409 | 0.000907969 | 0.013253026 |
| DDX47 | -0.483881348 | 0.000916375 | 0.013353181 |
| RASD1 | 1.948052723 | 0.000918954 | 0.013376 |
| MRPL2 | -0.612655403 | 0.000928745 | 0.013503626 |
| GTF3C5 | -0.610497362 | 0.000931437 | 0.013527866 |
| RAD52 | -0.687371352 | 0.00093412 | 0.013551929 |
| THG1L | -0.55745579 | 0.000943142 | 0.01366779 |
| RMDN3 | 0.740438092 | 0.000952426 | 0.013787198 |
| MEIS1 | 0.886151481 | 0.000953677 | 0.013790195 |
| GLIS1 | 0.84148522 | 0.0009678 | 0.013948576 |
| EZR | -0.571089518 | 0.000966792 | 0.013948576 |
| LOC118248843 | -0.553825317 | 0.000966896 | 0.013948576 |
| CUNH21orf91 | -0.567267887 | 0.000971582 | 0.013987818 |
| LOC118253250 | -0.554594211 | 0.000980264 | 0.014097439 |
| B4GALT3 | 0.687028522 | 0.000982165 | 0.01410941 |
| PLB1 | 1.700295067 | 0.000985701 | 0.014125087 |
| MTCH2 | -0.695902852 | 0.000989552 | 0.014125087 |
| ENSACYG00000002341 | -2.660909032 | 0.000989676 | 0.014125087 |
| LOC118247123 | 2.454066719 | 0.000988075 | 0.014125087 |
| FOXM1 | 0.877858151 | 0.000987569 | 0.014125087 |
| PAK1IP1 | -0.663945931 | 0.00098546 | 0.014125087 |
| CUNH2orf69 | -0.570880881 | 0.000992546 | 0.01415075 |
| NFKB2 | -0.554914429 | 0.000994897 | 0.014153697 |
| GRM7 | 0.587903155 | 0.000994613 | 0.014153697 |
| MMD | 0.535552032 | 0.000997381 | 0.01417376 |
| NDUFB9 | -0.654916935 | 0.001005952 | 0.014272872 |
| SLC29A3 | -0.80351248 | 0.001006517 | 0.014272872 |
| SCN8A | 0.606153909 | 0.001009483 | 0.014299564 |
| S1PR3 | 0.687825081 | 0.00101172 | 0.014315889 |
| GSG1 | 0.539321342 | 0.001018221 | 0.014392461 |
| MIA3 | -0.572064888 | 0.001020898 | 0.014414855 |
| UNC13B | 0.896697088 | 0.001027343 | 0.014467775 |
| ZNF521 | 0.480906295 | 0.001027933 | 0.014467775 |
| FNDC3A | 0.500617529 | 0.001025775 | 0.014467775 |
| PGBD5 | 0.815694999 | 0.001036108 | 0.014567303 |
| TMEM200A | 0.732880755 | 0.001042365 | 0.01463969 |
| ENSACYG00000004415 | 2.62384456 | 0.001051003 | 0.014745317 |
| GLIS3 | 0.501959562 | 0.001052356 | 0.014748618 |
| LOC118253833 | 2.233775145 | 0.001064557 | 0.014903804 |
| EXOSC7 | -0.582232858 | 0.001069734 | 0.014960415 |
| CUNH5orf24 | 0.452626488 | 0.001079928 | 0.015087 |
| GGT5 | 3.110555842 | 0.00108108 | 0.015087129 |
| RARS1 | -0.475879676 | 0.001093747 | 0.015247776 |
| BACE1 | 0.856754217 | 0.001098536 | 0.015298394 |
| CBFB | 0.467885618 | 0.00110112 | 0.015302094 |
| ENSACYG00000015590 | 3.122250684 | 0.001100793 | 0.015302094 |
| BLCAP | 0.996428377 | 0.001105144 | 0.015341864 |
| CACUL1 | 0.534027879 | 0.001112189 | 0.015423439 |
| FAM214A | 0.46831828 | 0.001115614 | 0.015454713 |
| STAT1 | 0.566851192 | 0.001123442 | 0.015546839 |
| NCLN | -0.492088193 | 0.00113155 | 0.015642641 |
| ENSACYG00000000629 | 0.797782377 | 0.001140933 | 0.01575585 |
| SMYD4 | -0.562426747 | 0.00114442 | 0.015787502 |
| GPR27 | 0.503371255 | 0.001151893 | 0.015874 |
| SYNM | -0.66207539 | 0.001155276 | 0.015885181 |
| PEAK1 | 0.463774607 | 0.001156314 | 0.015885181 |
| ICMT | 0.500086245 | 0.001155035 | 0.015885181 |
| DPP7 | -0.465917326 | 0.001164227 | 0.015962542 |
| INSIG1 | -0.483199923 | 0.001164364 | 0.015962542 |
| SCAMP4 | 0.752333839 | 0.001170104 | 0.016024599 |
| ECHS1 | -0.585410838 | 0.001172962 | 0.016047088 |
| DARS2 | -0.718044574 | 0.001177509 | 0.016092626 |
| EVI5L | 0.60943843 | 0.001195294 | 0.016318793 |
| ENSACYG00000006346 | 0.483865566 | 0.001205863 | 0.016446084 |
| HHAT | 0.518628569 | 0.001207214 | 0.016447509 |
| ROCK2 | -0.450881396 | 0.001217515 | 0.016570755 |
| ISY1 | -0.534320435 | 0.001220933 | 0.016600158 |
| EPAS1 | 0.763123594 | 0.001223589 | 0.016619163 |
| PTGS2 | -0.822925924 | 0.001228685 | 0.016671229 |
| HCRTR2 | 5.384345791 | 0.001236989 | 0.016766665 |
| ATR | -0.902515948 | 0.001246011 | 0.016871629 |
| MRPS10 | -0.64402296 | 0.001250914 | 0.016920661 |
| YARS2 | -0.569979496 | 0.001254549 | 0.016952456 |
| ENSACYG00000016817 | 3.880079818 | 0.001257098 | 0.016969542 |
| LOC118251000 | -0.697271747 | 0.001260654 | 0.017000155 |
| SUSD3 | 0.499143298 | 0.001267128 | 0.017070029 |
| ENSACYG00000004126 | 3.771591077 | 0.001273608 | 0.01712238 |
| DR1 | 0.479642834 | 0.001272993 | 0.01712238 |
| TKFC | -0.792622573 | 0.001278123 | 0.017165601 |
| PFN2 | 0.471210111 | 0.001283867 | 0.017225221 |
| ENSACYG00000000445 | 1.608980042 | 0.001290034 | 0.01729038 |
| CHRDL1 | 0.453787964 | 0.001292413 | 0.017304706 |
| GPRC5A | 5.383530647 | 0.001293752 | 0.017305076 |
| EXTL3 | 0.648028928 | 0.001299858 | 0.017369149 |
| DAP | 0.475676826 | 0.001302606 | 0.017388279 |
| ARRDC3 | -0.654261957 | 0.001304936 | 0.017401787 |
| AIMP1 | -0.563735431 | 0.001306603 | 0.017406433 |
| TRPC7 | 5.377329379 | 0.001317215 | 0.017530115 |
| CHD4 | -0.597758147 | 0.001321311 | 0.017549246 |
| FAP | -0.527893425 | 0.001321066 | 0.017549246 |
| ENSACYG00000006979 | 4.937651154 | 0.001323617 | 0.017562198 |
| LOC118246339 | 2.060417971 | 0.0013386 | 0.017743174 |
| DDX10 | -0.643809688 | 0.001340867 | 0.017755386 |
| RUFY1 | -0.534301443 | 0.001342752 | 0.017762539 |
| ENOX2 | 0.585861689 | 0.00134629 | 0.017791508 |
| TRIB2 | -0.912847106 | 0.001347878 | 0.017794691 |
| LOC118246505 | 1.61093871 | 0.001350594 | 0.017794947 |
| HSP90B1 | -0.44386302 | 0.001349978 | 0.017794947 |
| NMRK2 | -0.638978361 | 0.001355561 | 0.017842582 |
| KLB | 3.808116773 | 0.001368743 | 0.017998157 |
| ALG3 | -0.73075289 | 0.001375212 | 0.018065227 |
| TCF7 | 1.292420077 | 0.001378045 | 0.018084438 |
| PICK1 | -0.699917813 | 0.001379642 | 0.018087423 |
| BDNF | -0.493358235 | 0.001384423 | 0.018132093 |
| EXT1 | 0.611019702 | 0.001395553 | 0.018259747 |
| NR1H3 | 0.624918013 | 0.001404699 | 0.018361218 |
| RNF111 | 0.671866576 | 0.001415315 | 0.018481692 |
| IDS | -0.509329926 | 0.001417469 | 0.018491526 |
| IKBIP | -0.535243015 | 0.001420277 | 0.018503256 |
| ATP8B2 | 0.91212736 | 0.001421605 | 0.018503256 |
| ENSACYG00000015774 | 0.631116893 | 0.001422573 | 0.018503256 |
| SGO1 | -0.659193688 | 0.001451962 | 0.018866936 |
| ENSACYG00000004515 | 5.42612661 | 0.001455293 | 0.018873059 |
| PWWP2A | -0.478068319 | 0.001455259 | 0.018873059 |
| PSMB3 | -0.568554546 | 0.001456909 | 0.018875474 |
| KLHL31 | 1.40971134 | 0.001463029 | 0.018884569 |
| LOC118253663 | -0.53073572 | 0.001463332 | 0.018884569 |
| WBP1 | 0.815216962 | 0.001461079 | 0.018884569 |
| GPR89B | -0.519477906 | 0.001462631 | 0.018884569 |
| CDC42EP2 | -0.847434254 | 0.001470122 | 0.018947451 |
| CCT5 | -0.443631217 | 0.001471075 | 0.018947451 |
| CUNH19orf47 | 0.593069527 | 0.001481856 | 0.019067701 |
| MYOF | -0.584791941 | 0.001483975 | 0.019076374 |
| FKBP4 | -0.430110322 | 0.001486323 | 0.019087968 |
| TMEM140 | 0.555426273 | 0.001488751 | 0.01910057 |
| TOB2 | -0.724674029 | 0.001493543 | 0.019143445 |
| ENSACYG00000006681 | 4.220332326 | 0.001504733 | 0.019268171 |
| CCNB3 | -0.929278914 | 0.001510491 | 0.019323162 |
| LOC118260588 | -0.976398903 | 0.001512464 | 0.019329677 |
| CDCA7L | 0.475129756 | 0.001528638 | 0.019512654 |
| GMPPB | -0.618451541 | 0.001529738 | 0.019512654 |
| LOC118244251 | 3.075727747 | 0.001534327 | 0.019552308 |
| ENSACYG00000006881 | 1.394733211 | 0.001542794 | 0.019641244 |
| MTFR1 | 0.516873873 | 0.00154958 | 0.019708632 |
| CDC5L | -0.563847612 | 0.001555796 | 0.019768636 |
| PITPNM2 | 0.844803996 | 0.001561527 | 0.019815941 |
| CGREF1 | -0.699162781 | 0.00156252 | 0.019815941 |
| TUB | 0.530957739 | 0.001575342 | 0.019942166 |
| TMTC1 | 0.602500972 | 0.001575495 | 0.019942166 |
| LYAR | -0.645156875 | 0.001578895 | 0.019966068 |
| SRP72 | -0.422200198 | 0.001584847 | 0.020022158 |
| RGL1 | 0.62396604 | 0.001602349 | 0.020105082 |
| ING2 | -0.694668382 | 0.001601316 | 0.020105082 |
| RNF130 | 0.440493048 | 0.001593391 | 0.020105082 |
| DOT1L | 0.460579295 | 0.001597912 | 0.020105082 |
| BCL9L | 0.836744509 | 0.00159529 | 0.020105082 |
| RBSN | 0.63155016 | 0.001603594 | 0.020105082 |
| LOC118261520 | 2.246702969 | 0.001601776 | 0.020105082 |
| VPS45 | -0.489688549 | 0.001603304 | 0.020105082 |
| CCT4 | -0.474416463 | 0.001620979 | 0.020303764 |
| GUCY1A2 | 0.679693711 | 0.001622571 | 0.020304443 |
| DYNC2H1 | -0.672991391 | 0.001627311 | 0.020344464 |
| ARHGEF4 | -0.631114183 | 0.0016337 | 0.020405019 |
| ZNF362 | 0.685027062 | 0.001636946 | 0.020426233 |
| SRSF3 | -0.481004287 | 0.001641791 | 0.020448044 |
| NAT9 | -0.59373009 | 0.001641716 | 0.020448044 |
| LOC118250417 | -0.497463856 | 0.001654655 | 0.020588832 |
| ATP5PO | -0.552596778 | 0.001657868 | 0.020609394 |
| XXYLT1 | 0.481245434 | 0.001667143 | 0.020705193 |
| CCDC47 | -0.446670191 | 0.001670355 | 0.020725595 |
| EFNB2 | 0.743058419 | 0.001675051 | 0.020744858 |
| XPO6 | 0.774487504 | 0.001673541 | 0.020744858 |
| EXOSC2 | -0.503661162 | 0.00168141 | 0.020804098 |
| ENSACYG00000003391 | 0.572434942 | 0.001683006 | 0.020804349 |
| FAM102A | 0.621610786 | 0.001686598 | 0.020829249 |
| PTGDR | -0.940467404 | 0.001694783 | 0.020910769 |
| PIM1 | -0.86617884 | 0.00170164 | 0.020975773 |
| SMPD2 | -0.727786847 | 0.001711977 | 0.02108351 |
| TPMT | -0.598271672 | 0.001719023 | 0.021146842 |
| TTC30B | -0.674348831 | 0.001720482 | 0.021146842 |
| PAIP1 | -0.524855454 | 0.001721925 | 0.021146842 |
| ENSACYG00000015447 | 2.18396992 | 0.001726025 | 0.021177494 |
| DCTN1 | -0.902701166 | 0.001727959 | 0.021181533 |
| POMGNT1 | 0.678151928 | 0.001729941 | 0.021186158 |
| SQOR | -0.459125593 | 0.001741416 | 0.021306923 |
| LOC118244463 | -0.450181164 | 0.001756528 | 0.021432236 |
| GNAI1 | -0.455085135 | 0.001756478 | 0.021432236 |
| YWHAH | -0.445723612 | 0.001754337 | 0.021432236 |
| GPATCH4 | -0.753265059 | 0.001763708 | 0.021499976 |
| CCPG1 | -0.545155191 | 0.001765894 | 0.021506759 |
| LOC118251084 | -0.51211121 | 0.001772922 | 0.021552593 |
| GABRR2 | 4.823151033 | 0.001772226 | 0.021552593 |
| IPMK | 0.687912869 | 0.001781919 | 0.021622143 |
| ENSACYG00000004338 | 3.59354444 | 0.001780319 | 0.021622143 |
| TAP2 | -0.598311876 | 0.001784218 | 0.021630156 |
| HRAS | 0.462981549 | 0.001786464 | 0.021637526 |
| LYPD6 | 1.191671154 | 0.001793613 | 0.021688545 |
| MPP3 | -0.984216916 | 0.001793962 | 0.021688545 |
| EIF4E3 | 0.436490678 | 0.001800045 | 0.021742174 |
| GTF3C1 | -0.521147997 | 0.00181832 | 0.02194283 |
| REXO5 | -0.749187805 | 0.001823124 | 0.021980714 |
| TMEM185A | 0.530402189 | 0.001834442 | 0.022096995 |
| SPTAN1 | -0.610623039 | 0.001841378 | 0.02216032 |
| LOC118247154 | 2.176127352 | 0.001849933 | 0.022243003 |
| LOC118247056 | -0.454677893 | 0.001869596 | 0.022458974 |
| VPS37C | 0.535737051 | 0.001871421 | 0.02246046 |
| LOC118248278 | -0.703268108 | 0.00187742 | 0.022511987 |
| ERI2 | 0.77938153 | 0.00188327 | 0.022553041 |
| ADGRD1 | 0.560960627 | 0.00188426 | 0.022553041 |
| ENSACYG00000005690 | 4.239362558 | 0.001888949 | 0.022568381 |
| LOC118256513 | -0.551153767 | 0.001888961 | 0.022568381 |
| TRMT10C | -0.579602105 | 0.001893386 | 0.022580376 |
| ENSACYG00000004717 | 0.518513296 | 0.001892319 | 0.022580376 |
| PPWD1 | 0.451186499 | 0.001917332 | 0.022845325 |
| ENSACYG00000005147 | 3.697055032 | 0.001924729 | 0.022912777 |
| INTS12 | -0.806497676 | 0.001944501 | 0.023127296 |
| ENSACYG00000008470 | 1.94125499 | 0.001961558 | 0.023309169 |
| JDP2 | -0.653911334 | 0.001965965 | 0.023340533 |
| YLPM1 | -1.059698637 | 0.001968721 | 0.023352245 |
| LOC118243466 | 4.223950727 | 0.001976765 | 0.023426616 |
| KCNS1 | 3.473449114 | 0.001988225 | 0.023541295 |
| PRELID2 | 0.451797252 | 0.001994152 | 0.023590316 |
| MAP2K5 | 0.432034933 | 0.002007848 | 0.023716272 |
| IST1 | -0.497147954 | 0.002008392 | 0.023716272 |
| DUSP12 | -0.601519465 | 0.002016005 | 0.023784894 |
| MYNN | -0.73808236 | 0.002024363 | 0.023862174 |
| RPS6KA5 | -0.578437384 | 0.002029917 | 0.023887974 |
| FARP2 | 0.599450741 | 0.00203198 | 0.023887974 |
| TBC1D16 | 0.587574548 | 0.002030201 | 0.023887974 |
| MLST8 | -0.465893383 | 0.002033954 | 0.023889912 |
| LOC118255688 | 0.493490092 | 0.002044241 | 0.023989392 |
| LOC118252466 | 4.210819041 | 0.002050183 | 0.02402085 |
| NOP14 | -0.638575655 | 0.00205056 | 0.02402085 |
| RAB11FIP3 | 0.920208527 | 0.002054069 | 0.02404062 |
| FBRSL1 | 0.715951537 | 0.002070428 | 0.024210618 |
| HMMR | -0.572572039 | 0.00207413 | 0.024232447 |
| RNASEH2B | -0.492382129 | 0.002077112 | 0.024245828 |
| DNMBP | -0.754238414 | 0.002081189 | 0.024271959 |
| ENSACYG00000002918 | -1.316491878 | 0.002086502 | 0.024312449 |
| MARCHF1 | 0.691529704 | 0.002113753 | 0.024598417 |
| RNF114 | 0.440510041 | 0.002114771 | 0.024598417 |
| BAZ1A | -0.628352381 | 0.002124774 | 0.024673138 |
| PAQR5 | 0.538755969 | 0.002124932 | 0.024673138 |
| ENSACYG00000003333 | -0.721492378 | 0.002138169 | 0.024783236 |
| NAA35 | -0.436322454 | 0.002137417 | 0.024783236 |
| CLIP1 | -0.443165955 | 0.002142266 | 0.024808938 |
| NLN | -0.453610442 | 0.002157105 | 0.024937967 |
| LOC118258134 | 0.814721257 | 0.002157185 | 0.024937967 |
| PGM2 | -0.453328841 | 0.002168461 | 0.025046387 |
| METTL5 | -0.57018592 | 0.00217302 | 0.02507711 |
| HSP90AB1 | -1.02694157 | 0.002181783 | 0.025112374 |
| ENSACYG00000008861 | 0.594828984 | 0.002181319 | 0.025112374 |
| PEPD | -0.483062187 | 0.002181682 | 0.025112374 |
| PER2 | 0.698851352 | 0.002187712 | 0.025158683 |
| RAB19 | -0.753521095 | 0.002190226 | 0.025165674 |
| LOC118260427 | -0.531329125 | 0.002202105 | 0.025280171 |
| ENSACYG00000010584 | 0.703250963 | 0.002216078 | 0.025418476 |
| NFKBIE | 0.468855098 | 0.002222293 | 0.025445547 |
| ENSACYG00000009408 | 3.747696542 | 0.002221919 | 0.025445547 |
| DOP1B | -0.62507145 | 0.002242446 | 0.025654047 |
| SNX17 | 0.422562712 | 0.002265642 | 0.025896975 |
| PSMC2 | -0.476526356 | 0.002269319 | 0.025916564 |
| CIITA | 3.079775331 | 0.002277125 | 0.02594258 |
| PIGM | -0.641528379 | 0.002277492 | 0.02594258 |
| ACER3 | 0.516280553 | 0.002276061 | 0.02594258 |
| ENSACYG00000006849 | 0.89569441 | 0.002294478 | 0.026113538 |
| SMC4 | -0.52853723 | 0.002300133 | 0.026145385 |
| SRPRA | 0.466891031 | 0.002301238 | 0.026145385 |
| DAP3 | -0.4211491 | 0.002316014 | 0.026262768 |
| GJC2 | 0.674051611 | 0.002317537 | 0.026262768 |
| ENSACYG00000011468 | 1.031020489 | 0.002314972 | 0.026262768 |
| NUF2 | -0.496767155 | 0.00232785 | 0.026357012 |
| IRF2 | -0.502095538 | 0.002341638 | 0.026464297 |
| ROPN1L | -0.592011696 | 0.002343339 | 0.026464297 |
| KIAA0319L | 0.779809171 | 0.002340748 | 0.026464297 |
| ENSACYG00000003889 | 1.968696169 | 0.002346511 | 0.026465897 |
| LOC118261652 | -0.490617038 | 0.00234749 | 0.026465897 |
| LOC118258353 | -0.646472862 | 0.002350195 | 0.02647378 |
| LOC118259110 | -0.589243888 | 0.002356314 | 0.026520081 |
| CABIN1 | -0.508093404 | 0.002360236 | 0.026541595 |
| LOC118245372 | -2.144069584 | 0.002367983 | 0.026583433 |
| ENSACYG00000003588 | 0.685708671 | 0.002366046 | 0.026583433 |
| A1CF | 4.724344993 | 0.002372366 | 0.026588 |
| ENSACYG00000003384 | -1.213220566 | 0.002373838 | 0.026588 |
| GALNT17 | -4.829657379 | 0.00237531 | 0.026588 |
| EP300 | 0.595943323 | 0.002376446 | 0.026588 |
| FZD8 | -1.141093402 | 0.002384197 | 0.026645567 |
| RNF20 | -0.460541693 | 0.002385628 | 0.026645567 |
| CDCA4 | -0.496845114 | 0.002404265 | 0.026763441 |
| LSM1 | -0.547996174 | 0.002401933 | 0.026763441 |
| TMEM9 | -0.516959239 | 0.00240429 | 0.026763441 |
| ENSACYG00000014400 | -0.996284895 | 0.002399236 | 0.026763441 |
| MSH2 | -0.503860569 | 0.002407312 | 0.026774499 |
| DNAAF4 | -0.560719923 | 0.002409535 | 0.026776663 |
| GADD45G | -0.690363259 | 0.002413017 | 0.02679281 |
| RILP | -0.776988136 | 0.002415062 | 0.026792977 |
| ENSACYG00000011163 | -1.801120594 | 0.002422783 | 0.026833543 |
| ASPM | -0.741095513 | 0.002421002 | 0.026833543 |
| AHRR | 0.628790864 | 0.002429185 | 0.026881899 |
| ASCC3 | -0.523361586 | 0.002431429 | 0.026884192 |
| P3H3 | -0.712800903 | 0.002434 | 0.026890095 |
| B3GNT7 | -0.688574347 | 0.002446664 | 0.026983407 |
| MRPS35 | -0.545914937 | 0.002448578 | 0.026983407 |
| POLR3H | -0.519311373 | 0.002447953 | 0.026983407 |
| GDAP2 | 0.465806203 | 0.002464568 | 0.027120572 |
| MAP1A | -0.671317588 | 0.002465133 | 0.027120572 |
| MFSD10 | -0.511714686 | 0.002469422 | 0.02714514 |
| MGAT5 | 0.592547898 | 0.002482818 | 0.027269688 |
| GABPB1 | -0.541159719 | 0.002487909 | 0.027302893 |
| ACVR2A | 0.52633316 | 0.002506421 | 0.0274832 |
| PRRT1B | 0.443739084 | 0.002509337 | 0.02749234 |
| AP2A2 | -0.472744195 | 0.0025131 | 0.027510735 |
| POC5 | -0.483044617 | 0.002517353 | 0.027534461 |
| SEC14L1 | 0.459126449 | 0.002523906 | 0.027560464 |
| MFSD5 | 0.485728007 | 0.002523777 | 0.027560464 |
| TLE5 | 0.570109812 | 0.002526727 | 0.027568471 |
| TMEM11 | -0.587661913 | 0.002545553 | 0.02775094 |
| BEND5 | -0.996149171 | 0.002548572 | 0.027760931 |
| CDKN1C | -0.62599093 | 0.002556645 | 0.027825907 |
| LCT | 2.315859256 | 0.002579888 | 0.028032664 |
| UBB | -0.528754718 | 0.002579819 | 0.028032664 |
| GLB1 | 0.725064302 | 0.002598031 | 0.028206579 |
| RASSF6 | 0.468873125 | 0.00261779 | 0.028304723 |
| CHN1 | 0.431885548 | 0.002610165 | 0.028304723 |
| RTKN2 | 0.659327351 | 0.002614321 | 0.028304723 |
| BTD | -0.537605902 | 0.002616613 | 0.028304723 |
| SEC14L2 | -0.574625274 | 0.002613086 | 0.028304723 |
| YJU2 | -0.549334462 | 0.002620351 | 0.028309222 |
| TLCD2 | -0.612606026 | 0.002632092 | 0.02841282 |
| BABAM2 | 0.429144134 | 0.002642372 | 0.028500486 |
| CHUK | -0.950142564 | 0.002649258 | 0.028551433 |
| TTC4 | -0.494994385 | 0.002679731 | 0.028845523 |
| PPIL1 | -0.674859729 | 0.002680916 | 0.028845523 |
| CTNND2 | 0.616605467 | 0.002685208 | 0.028868174 |
| PCDH19 | 0.786659298 | 0.002693143 | 0.028929917 |
| TRIM46 | -1.662483176 | 0.00270365 | 0.02901918 |
| NUDT13 | -0.898610229 | 0.002719635 | 0.029167031 |
| CCDC80 | -0.4103546 | 0.002726038 | 0.029188282 |
| LOC118243257 | 0.446340565 | 0.002724494 | 0.029188282 |
| TMEM132B | 4.71770758 | 0.002739823 | 0.029288379 |
| LOC118244827 | 0.562834232 | 0.002738417 | 0.029288379 |
| SPRY3 | 0.583173274 | 0.002744074 | 0.029302573 |
| ATP6AP1 | -0.514001366 | 0.002747256 | 0.029302573 |
| CUNH16orf87 | -0.624463901 | 0.00274781 | 0.029302573 |
| ALG1 | -0.51833114 | 0.002790771 | 0.029736687 |
| SNX31 | 4.747299195 | 0.002815663 | 0.029879894 |
| ENSACYG00000001739 | 0.629370454 | 0.002817354 | 0.029879894 |
| AIDA | 0.431685117 | 0.00281429 | 0.029879894 |
| LOC118245898 | 3.05141426 | 0.002812675 | 0.029879894 |
| TTBK2 | 0.766971853 | 0.00281779 | 0.029879894 |
| SEPTIN9 | 0.781282056 | 0.002812699 | 0.029879894 |
| R3HCC1L | -0.777964293 | 0.00282426 | 0.029924465 |
| MDN1 | -0.594818695 | 0.002830865 | 0.029964687 |
| CDK11A | -0.535911225 | 0.002832596 | 0.029964687 |
| LOC118254088 | -0.843248715 | 0.00285091 | 0.030134277 |
| BMP4 | 0.545780996 | 0.002858766 | 0.030193142 |
| LOC118258128 | -0.619809181 | 0.002871413 | 0.030302477 |
| MVB12B | 0.55698371 | 0.00287603 | 0.030326955 |
| FLVCR2 | 0.56588621 | 0.002880421 | 0.030349014 |
| SHPRH | -0.61735175 | 0.002892027 | 0.030443199 |
| WNT5B | -0.538572805 | 0.002893972 | 0.030443199 |
| DMAC2L | -0.53899914 | 0.002906134 | 0.030546796 |
| B3GNT9 | 0.606238671 | 0.002926917 | 0.030740775 |
| GTF2H1 | 0.49311921 | 0.002939032 | 0.030843481 |
| GUSB | -0.437702326 | 0.002957083 | 0.031008268 |
| MRPS27 | -0.446979071 | 0.002965905 | 0.031076094 |
| UBN2 | 0.752709369 | 0.002971094 | 0.031105774 |
| ENSACYG00000012367 | 4.11691378 | 0.002978593 | 0.031159577 |
| IL11RA | 0.422201158 | 0.00298804 | 0.031233652 |
| FASTKD2 | -0.437787466 | 0.003009912 | 0.031425638 |
| NDUFAF5 | -0.589902828 | 0.003011167 | 0.031425638 |
| LIN52 | 0.47306508 | 0.003016419 | 0.031455578 |
| GRB10 | -0.499595125 | 0.00305032 | 0.031783995 |
| ARFGEF1 | -0.672566192 | 0.003052734 | 0.031784063 |
| RHOBTB2 | 0.732432725 | 0.003074834 | 0.031988937 |
| ENSACYG00000015280 | 0.674371432 | 0.003080538 | 0.032023047 |
| BTBD10 | -0.411462682 | 0.003083593 | 0.032029583 |
| TSGA10 | 2.965588391 | 0.003096215 | 0.032135406 |
| MEX3B | -0.588032017 | 0.003100902 | 0.032158764 |
| ENSACYG00000007005 | 1.746071542 | 0.003109655 | 0.032224223 |
| TIFA | -0.694516582 | 0.003126053 | 0.032368745 |
| PSPH | -0.63872403 | 0.003157961 | 0.032622379 |
| NBAS | -0.536548652 | 0.003153116 | 0.032622379 |
| RDM1 | -0.513138991 | 0.003156637 | 0.032622379 |
| OAF | 0.5330868 | 0.003179631 | 0.032820551 |
| DHX29 | -0.597195991 | 0.003186753 | 0.032868372 |
| BCAN | -0.598492503 | 0.003213181 | 0.033115078 |
| LOC118243167 | -0.566123823 | 0.003221778 | 0.033177783 |
| HIVEP3 | 0.580588115 | 0.003225326 | 0.033188432 |
| TMEM177 | -0.485111149 | 0.003232857 | 0.033216828 |
| ENSACYG00000008044 | -0.706485063 | 0.003233118 | 0.033216828 |
| RRS1 | -0.786882156 | 0.003249699 | 0.033353792 |
| NDUFA11 | -0.612170042 | 0.003251502 | 0.033353792 |
| IER5 | -1.111728351 | 0.003254836 | 0.033362071 |
| PDZD8 | 0.409753893 | 0.003264878 | 0.033439038 |
| PKIA | 0.461963098 | 0.003276958 | 0.033536746 |
| CPD | -0.555431102 | 0.003281705 | 0.033559306 |
| EFL1 | -0.474559477 | 0.003307519 | 0.033707143 |
| BMS1 | -0.545700121 | 0.003304672 | 0.033707143 |
| KIF20B | -0.514749291 | 0.003301666 | 0.033707143 |
| CCNJ | -0.601384146 | 0.003308927 | 0.033707143 |
| ENSACYG00000014743 | 0.653998516 | 0.003305789 | 0.033707143 |
| ADAR | 0.699951422 | 0.003316347 | 0.033726555 |
| LYSMD4 | -0.594305977 | 0.003315064 | 0.033726555 |
| LOC118248016 | -0.433734576 | 0.003318497 | 0.033726555 |
| CRTAP | -0.433258871 | 0.003328683 | 0.033804054 |
| LRRC36 | 0.802869713 | 0.003337203 | 0.033864532 |
| PDE4B | 0.492096972 | 0.003345776 | 0.033925448 |
| NTNG2 | 0.731466783 | 0.003362493 | 0.034068789 |
| EPB41L4A | 0.535030683 | 0.003367475 | 0.034093106 |
| SYNCRIP | -0.393622498 | 0.003380797 | 0.034201753 |
| ENSACYG00000011174 | 0.51933285 | 0.003390837 | 0.034277057 |
| STRADB | 0.551322706 | 0.003425815 | 0.034474662 |
| ANGPT1 | -0.419498364 | 0.003431276 | 0.034474662 |
| LOC118244586 | 3.643991691 | 0.003430639 | 0.034474662 |
| NIP7 | -0.52908857 | 0.003422264 | 0.034474662 |
| LOC118252907 | -0.575404973 | 0.003425585 | 0.034474662 |
| GFM1 | -0.443084936 | 0.003424548 | 0.034474662 |
| IGSF9B | 0.615956252 | 0.003428861 | 0.034474662 |
| TBCCD1 | -0.525421199 | 0.003422905 | 0.034474662 |
| SP1 | 0.770422348 | 0.003441291 | 0.034548992 |
| SLC2A8 | -0.428272734 | 0.00344749 | 0.034584921 |
| COLQ | 3.004538294 | 0.003455662 | 0.034640585 |
| POLH | -0.703259929 | 0.003467473 | 0.034706274 |
| SLAIN2 | 0.618562729 | 0.003465107 | 0.034706274 |
| ENSACYG00000005083 | 0.548632123 | 0.003470206 | 0.034707317 |
| TNFRSF21 | 0.648852897 | 0.003494159 | 0.034920428 |
| LOC118259705 | 0.528930413 | 0.003514181 | 0.035067999 |
| KIF4A | -0.50200867 | 0.003514237 | 0.035067999 |
| THOC2 | -0.429982087 | 0.003518922 | 0.035088227 |
| TSC22D2 | 0.569488226 | 0.003525709 | 0.035129364 |
| APRT | -0.582871783 | 0.003542855 | 0.035273582 |
| RPL24 | -0.501432297 | 0.003602635 | 0.035814752 |
| TMPRSS4 | -1.028776944 | 0.003600317 | 0.035814752 |
| GABPA | 0.608959283 | 0.003606439 | 0.035825593 |
| ENSACYG00000012379 | 1.065691939 | 0.003618121 | 0.035912092 |
| ENSACYG00000013189 | 4.034169463 | 0.003620587 | 0.035912092 |
| ARHGAP17 | 0.475202082 | 0.003628682 | 0.03596536 |
| SMAD4 | 0.45725768 | 0.003644279 | 0.036092856 |
| IL16 | 1.024760421 | 0.003648804 | 0.036110579 |
| TAGLN3 | 3.605864 | 0.003675701 | 0.03634952 |
| BTC | -0.890177876 | 0.00368308 | 0.036395223 |
| ENSACYG00000016135 | -0.771865836 | 0.003720061 | 0.036733171 |
| TET2 | 0.612403657 | 0.003731254 | 0.03681616 |
| CANT1 | -0.503382955 | 0.003734325 | 0.036818935 |
| ENSACYG00000009875 | 0.948715266 | 0.003738462 | 0.036832222 |
| WDR26 | -0.44578751 | 0.003749145 | 0.036909928 |
| PRRC2C | -0.621110794 | 0.00376683 | 0.037045683 |
| HAT1 | -0.458269684 | 0.003768547 | 0.037045683 |
| CBLB | -0.468947438 | 0.003787259 | 0.037174271 |
| TTC29 | 1.024764478 | 0.003786253 | 0.037174271 |
| SLC35B4 | 0.517694567 | 0.003795021 | 0.037222782 |
| FAM193A | -0.635830514 | 0.003801207 | 0.037255782 |
| CEP164 | 0.579180254 | 0.003809846 | 0.037285091 |
| LOC118255703 | -0.495325533 | 0.003807275 | 0.037285091 |
| GEMIN2 | -0.497288738 | 0.003813023 | 0.037288543 |
| MTHFD2 | -0.41936312 | 0.003823068 | 0.037359095 |
| C1S | 0.791137474 | 0.00383404 | 0.037438608 |
| DSE | 0.568025253 | 0.003836974 | 0.037439563 |
| ABI1 | 0.403984102 | 0.003856499 | 0.037586344 |
| ENSACYG00000016566 | 0.54418589 | 0.003857711 | 0.037586344 |
| MXD1 | 0.457164841 | 0.003868679 | 0.037665417 |
| ARHGEF10 | -0.486555265 | 0.003882575 | 0.037717257 |
| MRPL22 | -0.545785158 | 0.003878145 | 0.037717257 |
| SNAI2 | -0.69348292 | 0.003880234 | 0.037717257 |
| ASL | -0.460030229 | 0.003888741 | 0.037721643 |
| XYLB | -0.5769652 | 0.003885945 | 0.037721643 |
| PCBD2 | 0.922355446 | 0.003905379 | 0.037855221 |
| IFT20 | -0.506053315 | 0.0039256 | 0.037995432 |
| GATA2 | 1.673944349 | 0.003923621 | 0.037995432 |
| PTGER2 | -0.694159918 | 0.00393091 | 0.038015783 |
| TUBB6 | -0.397755586 | 0.003933462 | 0.038015783 |
| SLC7A3 | -0.547727756 | 0.003938599 | 0.038021637 |
| NFE2L1 | 0.656232986 | 0.003939827 | 0.038021637 |
| STK26 | 0.479559169 | 0.003943888 | 0.038033027 |
| ASF1A | -0.534110291 | 0.003963883 | 0.03817032 |
| CUNH18orf21 | -0.526821971 | 0.003963908 | 0.03817032 |
| CACTIN | 0.4801574 | 0.004002538 | 0.038514221 |
| SFT2D2 | 0.655865856 | 0.004016923 | 0.038596375 |
| MSRB3 | 0.525991193 | 0.004014766 | 0.038596375 |
| SNN | -0.62814102 | 0.004030125 | 0.038672768 |
| HTRA1 | -0.400719991 | 0.004030732 | 0.038672768 |
| FAM76B | -0.506784193 | 0.00405051 | 0.038834298 |
| SEPTIN6 | -0.421920845 | 0.004057592 | 0.038873968 |
| DDX46 | -0.476494647 | 0.004070785 | 0.038972081 |
| HS2ST1 | 0.492097288 | 0.004081613 | 0.039047435 |
| NUDT16L1 | -0.482509229 | 0.004095437 | 0.039151306 |
| OTOGL | 4.02520838 | 0.00410608 | 0.039224652 |
| TFCP2L1 | 1.771698782 | 0.004116215 | 0.039264643 |
| ENSACYG00000006221 | -0.713405293 | 0.004115661 | 0.039264643 |
| ENSACYG00000009714 | 0.530867511 | 0.004123589 | 0.039306582 |
| LOC118254226 | 2.900319087 | 0.0041295 | 0.039334527 |
| LOC118260146 | 0.573537479 | 0.004154002 | 0.039539386 |
| PHF14 | -0.555455252 | 0.004158816 | 0.039556692 |
| GLS | -0.378234443 | 0.004161927 | 0.039557786 |
| LOC118248563 | -0.534022002 | 0.004174043 | 0.03961886 |
| ARHGEF3 | -0.456470305 | 0.004174355 | 0.03961886 |
| CUNH10orf71 | 0.4613188 | 0.004191341 | 0.039740674 |
| ZBTB26 | 0.44972556 | 0.00419321 | 0.039740674 |
| CALCRL | -0.75251068 | 0.004198923 | 0.039766271 |
| IRF2BP2 | -0.569143908 | 0.004211376 | 0.039855616 |
| LOC118244275 | -0.541337798 | 0.004215503 | 0.039866096 |
| ARHGDIA | 0.390452508 | 0.004219199 | 0.039872486 |
| SMNDC1 | -0.490982201 | 0.004246732 | 0.040075312 |
| ENSACYG00000012312 | 1.942318719 | 0.004245871 | 0.040075312 |
| BRICD5 | 1.3217615 | 0.0042638 | 0.040207632 |
| ADCY5 | 0.711814533 | 0.004293713 | 0.040460811 |
| SPAG9 | -0.460032336 | 0.004323278 | 0.040710352 |
| VPS13A | -0.418922874 | 0.004327907 | 0.040724896 |
| DBNDD1 | -0.706077194 | 0.004342626 | 0.040834295 |
| KAZN | 0.643169856 | 0.004349536 | 0.040870157 |
| LOC118257323 | 3.995912588 | 0.004358149 | 0.040921965 |
| LOC118243739 | 3.166003986 | 0.004372442 | 0.041026995 |
| HHIPL1 | 0.58801319 | 0.004386071 | 0.041125649 |
| LOC118245540 | 0.394310234 | 0.004419099 | 0.041405923 |
| MAN1A2 | 0.56682443 | 0.004423021 | 0.04141328 |
| NECAP2 | 0.399503656 | 0.004426546 | 0.041416913 |
| ENSACYG00000007646 | 4.533112181 | 0.004431529 | 0.041434168 |
| WDR3 | -0.409311907 | 0.004437433 | 0.041460011 |
| KIFBP | -0.414721102 | 0.00449904 | 0.042005888 |
| CILK1 | 0.643448313 | 0.00450421 | 0.042024437 |
| LOC118248104 | -0.675823147 | 0.004513947 | 0.042085546 |
| COMMD1 | -0.650955728 | 0.004521417 | 0.042125441 |
| PML | 0.629748145 | 0.004531235 | 0.042187143 |
| GCNT3 | 4.094882459 | 0.004535107 | 0.042193431 |
| ENSACYG00000001024 | 3.504301367 | 0.004547339 | 0.042277446 |
| UBA6 | -0.647417815 | 0.004610377 | 0.042833357 |
| ARHGEF6 | 0.438667833 | 0.004622584 | 0.042916567 |
| MAP1B | -0.522580126 | 0.004646117 | 0.043044238 |
| TRIP13 | -0.557544453 | 0.00464461 | 0.043044238 |
| FICD | 0.608624723 | 0.004641509 | 0.043044238 |
| ENSACYG00000003157 | 3.505837685 | 0.004659247 | 0.043135607 |
| SMC5 | -0.55292884 | 0.004680954 | 0.043306201 |
| NTAQ1 | -0.520986136 | 0.004694911 | 0.043404913 |
| ENSACYG00000003901 | 0.51457165 | 0.004732782 | 0.043632814 |
| TMEM71 | 0.519250502 | 0.004727048 | 0.043632814 |
| PRKD3 | 0.717965683 | 0.004730361 | 0.043632814 |
| STC1 | -0.608719012 | 0.004723069 | 0.043632814 |
| CRAMP1 | 0.73383453 | 0.004737673 | 0.04364466 |
| RBPMS2 | 0.414151951 | 0.004740679 | 0.04364466 |
| SLC25A33 | -0.568550848 | 0.004748584 | 0.043686972 |
| STRIP2 | 1.233908712 | 0.004765601 | 0.043812999 |
| ARL11 | -0.473621325 | 0.004770123 | 0.043824052 |
| ASPG | -0.449836003 | 0.004788308 | 0.043960532 |
| PDSS1 | -0.431607151 | 0.004796721 | 0.044007163 |
| SERINC5 | 0.577041568 | 0.004805778 | 0.04402906 |
| FKBP7 | -0.502048442 | 0.004804088 | 0.04402906 |
| DDX20 | -0.433497923 | 0.004816246 | 0.044094367 |
| LOC118251281 | 0.479854113 | 0.004822105 | 0.044117414 |
| ADAM33 | -0.621777613 | 0.004840695 | 0.044256822 |
| ACAP3 | 0.531397651 | 0.00485635 | 0.04436923 |
| ENSACYG00000003677 | -0.810729603 | 0.004874883 | 0.044507745 |
| LOC118250227 | -0.476150142 | 0.004885729 | 0.044570314 |
| ZDHHC13 | -0.453318288 | 0.004890545 | 0.044570314 |
| CHRM4 | 0.868520793 | 0.004891864 | 0.044570314 |
| MSTO1 | -0.478796363 | 0.004900174 | 0.044615238 |
| TMEM184B | 0.504154989 | 0.004942864 | 0.044972909 |
| RND3 | -0.452629972 | 0.004955251 | 0.045054564 |
| TRMT2B | -0.75826861 | 0.004971078 | 0.045166262 |
| SDHAF2 | -0.511270232 | 0.004974379 | 0.045166262 |
| CCT3 | -0.407782374 | 0.004980515 | 0.045190901 |
| MRPS12 | -0.561630709 | 0.005000545 | 0.045310365 |
| STK25 | 0.716929535 | 0.00499734 | 0.045310365 |
| RNF38 | 0.473648299 | 0.005023232 | 0.045484714 |
| ENSACYG00000002590 | 0.608090444 | 0.005043886 | 0.045588082 |
| LHX6 | 0.701790381 | 0.00504846 | 0.045588082 |
| BRIX1 | -0.500444472 | 0.00504625 | 0.045588082 |
| HDLBP | 0.649613732 | 0.005045611 | 0.045588082 |
| EBI3 | -0.900003867 | 0.005060339 | 0.045638047 |
| YARS1 | -0.419808622 | 0.005060908 | 0.045638047 |
| STOML3 | 2.284000211 | 0.005070129 | 0.045689999 |
| TTC1 | -0.431858394 | 0.005079412 | 0.04572232 |
| RMND5A | -0.465481869 | 0.005080643 | 0.04572232 |
| ENSACYG00000016889 | 1.385280192 | 0.005087366 | 0.045751637 |
| LOC118247600 | -0.421373191 | 0.005093952 | 0.045779679 |
| ITGA2 | -0.532453304 | 0.005101723 | 0.045818331 |
| FRMD3 | 0.523610413 | 0.005115524 | 0.045911042 |
| DENND10 | -0.410752331 | 0.005128358 | 0.045970925 |
| RNF128 | -0.415227869 | 0.00512916 | 0.045970925 |
| TP53INP2 | 0.654749637 | 0.005143008 | 0.04606377 |
| MRPL18 | -0.541547207 | 0.005156703 | 0.046142161 |
| VILL | 1.351912074 | 0.005158751 | 0.046142161 |
| ING4 | -0.468566923 | 0.005170675 | 0.046193467 |
| SLC25A16 | 0.448996485 | 0.005171485 | 0.046193467 |
| FZD9 | 0.769726843 | 0.005191126 | 0.046337556 |
| UVSSA | -0.641923119 | 0.005247535 | 0.046809433 |
| DCAF10 | 0.461000537 | 0.005257112 | 0.046831576 |
| ING3 | -0.436673421 | 0.005254393 | 0.046831576 |
| LFNG | -0.568255199 | 0.005263247 | 0.046854607 |
| ABCB10 | 0.475176408 | 0.005297361 | 0.047126524 |
| IK | -0.443953154 | 0.005325795 | 0.047347568 |
| TCP1 | -0.423135297 | 0.005358039 | 0.047602173 |
| CASP2 | -0.891848103 | 0.005378891 | 0.047755292 |
| EVC | -0.638222319 | 0.005395798 | 0.0478732 |
| LRP1 | -0.465891386 | 0.00540966 | 0.04793177 |
| WDR73 | 0.446956865 | 0.005408197 | 0.04793177 |
| FADD | -0.496532813 | 0.005430514 | 0.048019861 |
| SHANK2 | 1.392798027 | 0.005428893 | 0.048019861 |
| RALBP1 | 0.394699309 | 0.005426002 | 0.048019861 |
| PHPT1 | -0.497556828 | 0.005459459 | 0.04824349 |
| LOC118245392 | 2.739148259 | 0.005465569 | 0.04826518 |
| RAE1 | -0.400849549 | 0.005474166 | 0.04830878 |
| ST6GAL1 | 0.608988859 | 0.005493606 | 0.048447954 |
| NOX3 | 0.826591953 | 0.005525765 | 0.048699027 |
| GPSM1 | 0.67425096 | 0.005533491 | 0.048734588 |
| CUNH5orf34 | -0.562944757 | 0.005576671 | 0.04908214 |
| MRPS11 | -0.507947093 | 0.005582869 | 0.049103959 |
| MRPL50 | -0.537728933 | 0.005586819 | 0.049105978 |
| ENSACYG00000003044 | 1.604343674 | 0.005600462 | 0.049160439 |
| PTCD3 | -0.504793543 | 0.005598608 | 0.049160439 |
| ENSACYG00000004263 | 1.867911119 | 0.005619215 | 0.049194216 |
| CAV1 | 0.484602933 | 0.005618444 | 0.049194216 |
| GCC2 | -0.419029424 | 0.005614095 | 0.049194216 |
| ZHX3 | 0.822495572 | 0.005608717 | 0.049194216 |
| PRDM4 | 0.476235781 | 0.005627445 | 0.049233617 |
| LOC118251891 | 0.421819219 | 0.005636166 | 0.049277262 |
| ZBTB6 | -0.86554822 | 0.005646554 | 0.04933541 |
| PDP1 | -0.621875607 | 0.005657561 | 0.049398888 |
| SRSF11 | -0.487989576 | 0.005672106 | 0.049493158 |
| TRAPPC2L | -0.507521602 | 0.005677196 | 0.049501358 |
| TMEM200C | 0.926736825 | 0.005680545 | 0.049501358 |
| RAB11FIP2 | 0.422412125 | 0.005698855 | 0.049593797 |
| ANKRD34B | 2.647281542 | 0.005699752 | 0.049593797 |
| CALB1 | 3.498203167 | 0.005702423 | 0.049593797 |
| ELOVL4 | -0.502044588 | 0.005726873 | 0.049773651 |
| LOC118249154 | 4.048936612 | 0.005735949 | 0.049819737 |
| ENSACYG00000002353 | -4.695020176 | 0.005751406 | 0.049895575 |
| ENSACYG00000005196 | -2.277470068 | 0.005776086 | 0.049895575 |
| FOXP1 | 0.431556238 | 0.005775097 | 0.049895575 |
| TUBGCP5 | -0.749777241 | 0.005762284 | 0.049895575 |
| LOC118258018 | -0.988384832 | 0.005760636 | 0.049895575 |
| FOXP4 | 0.589574223 | 0.005778695 | 0.049895575 |
| CEP19 | 0.482294511 | 0.005764385 | 0.049895575 |
| CHST11 | 1.506771891 | 0.005778427 | 0.049895575 |
| ESD | -0.514770258 | 0.005775315 | 0.049895575 |
