# Supplementary material for: The swan genome and transcriptome, it is not all black and white
Source: Genome Biol. 2023 Jan 23;24:13. doi: 10.1186/s13059-022-02838-0 (PMC9867998; doi:10.1186/s13059-022-02838-0)
Supplement: Supplementary file 12 — Additional file 12: Supplementary Table S10. Differentially expressed genes in infected duck endothelial cells. [file 13059_2022_2838_MOESM12_ESM.docx]

**Supplementary Table S10: Differentially expressed genes in infected duck endothelial cells**

| Gene name | log2FoldChange | pvalue | padj |
| --- | --- | --- | --- |
| BCOR | 4.810686 | 4.19E-106 | 4.57E-102 |
| PDE4B | 2.934439 | 1.90E-66 | 1.03E-62 |
| ENSAPLG00000031170 | 2.353503 | 2.90E-42 | 1.05E-38 |
| SOX8 | 2.215518 | 1.47E-41 | 3.99E-38 |
| MMP1 | -4.50723 | 1.64E-40 | 3.56E-37 |
| SCX | 2.310445 | 1.46E-38 | 2.65E-35 |
| RGS16 | 4.549672 | 2.30E-38 | 3.58E-35 |
| PCF11 | 2.449039 | 1.24E-37 | 1.69E-34 |
| ENSAPLG00000008725 | 2.601815 | 1.05E-36 | 1.27E-33 |
| DNAH10 | 4.816031 | 2.47E-36 | 2.70E-33 |
| HIC1 | 2.705134 | 3.04E-36 | 3.01E-33 |
| RBBP6 | 2.065838 | 1.75E-34 | 1.58E-31 |
| ENSAPLG00000016836 | 6.280844 | 2.31E-32 | 1.93E-29 |
| ENSAPLG00000007834 | 2.78189 | 5.52E-32 | 4.29E-29 |
| NR4A3 | 2.38368 | 1.16E-30 | 8.42E-28 |
| PRICKLE2 | 1.891648 | 1.38E-30 | 9.43E-28 |
| DIS3L | 1.795107 | 9.18E-30 | 5.88E-27 |
| ABCA12 | 5.66765 | 1.48E-28 | 8.96E-26 |
| TNFRSF21 | 1.777043 | 1.48E-26 | 8.46E-24 |
| ENSAPLG00000018309 | 1.670103 | 2.24E-26 | 1.22E-23 |
| ENSAPLG00000022613 | 4.854975 | 2.82E-26 | 1.46E-23 |
| HSPA5 | -1.7945 | 7.69E-26 | 3.81E-23 |
| LSMEM1 | 3.751869 | 9.63E-26 | 4.56E-23 |
| PDIA4 | -1.97121 | 1.31E-25 | 5.71E-23 |
| TIPARP | 1.805563 | 1.30E-25 | 5.71E-23 |
| ENSAPLG00000012692 | 2.332517 | 1.52E-24 | 6.35E-22 |
| FBLN1 | -1.76955 | 2.71E-24 | 1.09E-21 |
| ABHD5 | 1.604006 | 5.22E-24 | 2.03E-21 |
| CCN2 | -1.64509 | 5.43E-24 | 2.04E-21 |
| SPI1 | 2.528119 | 1.87E-23 | 6.80E-21 |
| HERPUD1 | -1.97792 | 5.23E-23 | 1.84E-20 |
| UPF3A | 3.692505 | 1.00E-22 | 3.41E-20 |
| ZNF821 | 1.71516 | 1.56E-22 | 5.15E-20 |
| SPHK1 | 1.637424 | 2.43E-22 | 7.78E-20 |
| ENSAPLG00000028586 | 8.516654 | 2.91E-22 | 9.06E-20 |
| CAMTA1 | 2.051929 | 4.93E-22 | 1.49E-19 |
| ENSAPLG00000013728 | 2.347574 | 5.46E-22 | 1.61E-19 |
| MCM5 | 2.092079 | 1.13E-21 | 3.24E-19 |
| ENSAPLG00000011493 | 3.474897 | 1.29E-21 | 3.60E-19 |
| RYR3 | 2.131532 | 2.01E-21 | 5.47E-19 |
| TRIM8 | 1.817933 | 7.57E-21 | 2.01E-18 |
| CCNI | 1.63774 | 1.48E-20 | 3.84E-18 |
| RBM20 | 7.226371 | 1.82E-20 | 4.61E-18 |
| ENSAPLG00000023444 | 1.98318 | 4.52E-20 | 1.12E-17 |
| PHGDH | -2.20133 | 6.42E-20 | 1.55E-17 |
| NAIF1 | 1.647313 | 9.08E-20 | 2.15E-17 |
| SERTAD2 | 2.232292 | 1.37E-19 | 3.17E-17 |
| CSTF3 | 1.95822 | 1.70E-19 | 3.86E-17 |
| RASGRP3 | 3.430584 | 2.41E-19 | 5.37E-17 |
| ENSAPLG00000013720 | 1.983787 | 3.17E-19 | 6.91E-17 |
| AKAP13 | 1.745062 | 7.54E-19 | 1.61E-16 |
| HSP90B1 | -1.46901 | 1.38E-18 | 2.90E-16 |
| ABHD17A | 1.760709 | 2.13E-18 | 4.34E-16 |
| ENSAPLG00000024296 | 1.735451 | 2.15E-18 | 4.34E-16 |
| HYOU1 | -1.38186 | 3.11E-18 | 6.16E-16 |
| CPZ | -1.95219 | 3.49E-18 | 6.79E-16 |
| POU2F1 | 1.598825 | 5.03E-18 | 9.44E-16 |
| ITGB4 | 4.387276 | 5.02E-18 | 9.44E-16 |
| AHDC1 | 2.846925 | 7.12E-18 | 1.31E-15 |
| ENSAPLG00000007205 | 1.827747 | 1.15E-17 | 2.07E-15 |
| NRAP | 3.989021 | 1.16E-17 | 2.07E-15 |
| ENSAPLG00000008620 | 6.82055 | 1.32E-17 | 2.32E-15 |
| CITED4 | -1.61848 | 1.64E-17 | 2.83E-15 |
| PRR7 | 2.068587 | 4.69E-17 | 7.98E-15 |
| HSD11B2 | 2.702323 | 5.14E-17 | 8.61E-15 |
| CREB5 | 1.39319 | 5.31E-17 | 8.76E-15 |
| ZNF628 | 2.237488 | 6.55E-17 | 1.06E-14 |
| FGGY | 1.746143 | 8.31E-17 | 1.32E-14 |
| SARS1 | -1.4909 | 8.39E-17 | 1.32E-14 |
| BCL2L11 | 2.594726 | 9.82E-17 | 1.53E-14 |
| ENSAPLG00000022960 | -1.73333 | 9.95E-17 | 1.53E-14 |
| GCLC | 1.273943 | 1.20E-16 | 1.81E-14 |
| PIGT | 1.443444 | 1.21E-16 | 1.81E-14 |
| ENSAPLG00000017312 | 3.002753 | 1.24E-16 | 1.83E-14 |
| PRRX1 | 1.823967 | 2.19E-16 | 3.18E-14 |
| MAGT1 | -1.63039 | 2.22E-16 | 3.18E-14 |
| ANAPC7 | -1.53106 | 2.78E-16 | 3.94E-14 |
| ENSAPLG00000006823 | 1.470652 | 4.20E-16 | 5.86E-14 |
| SAMD4A | 1.687709 | 4.42E-16 | 6.02E-14 |
| CD44 | -1.33815 | 4.38E-16 | 6.02E-14 |
| STK40 | 1.442348 | 4.71E-16 | 6.33E-14 |
| ASNS | -1.43873 | 5.31E-16 | 7.05E-14 |
| BCL6 | 1.74783 | 5.44E-16 | 7.14E-14 |
| ENSAPLG00000029778 | 1.440778 | 6.40E-16 | 8.30E-14 |
| ENSAPLG00000011089 | 1.41499 | 6.66E-16 | 8.53E-14 |
| FST | -1.53634 | 6.75E-16 | 8.55E-14 |
| KCNJ2 | 1.807775 | 7.67E-16 | 9.61E-14 |
| PENK | 2.088784 | 7.93E-16 | 9.82E-14 |
| PPP2R2B | 1.712104 | 9.68E-16 | 1.18E-13 |
| TMEM251 | 1.377445 | 1.25E-15 | 1.51E-13 |
| SLC35E3 | 1.277646 | 1.44E-15 | 1.73E-13 |
| FAM222B | 1.923048 | 1.77E-15 | 2.09E-13 |
| DSP | 4.366519 | 1.95E-15 | 2.29E-13 |
| HCN1 | 2.458019 | 2.49E-15 | 2.88E-13 |
| P4HB | -1.25625 | 2.69E-15 | 3.09E-13 |
| MAPK6 | 1.492345 | 3.05E-15 | 3.46E-13 |
| TET2 | 1.814368 | 5.51E-15 | 6.19E-13 |
| PDE8A | 1.713582 | 5.60E-15 | 6.23E-13 |
| PEPD | -1.42732 | 6.69E-15 | 7.36E-13 |
| COPB1 | -1.21658 | 7.55E-15 | 8.22E-13 |
| GYS2 | 8.38027 | 8.25E-15 | 8.90E-13 |
| UQCRC2 | -1.42094 | 8.62E-15 | 9.17E-13 |
| ARID1A | 1.89519 | 8.67E-15 | 9.17E-13 |
| GUSB | -1.43357 | 1.07E-14 | 1.12E-12 |
| ABHD6 | 2.349445 | 1.09E-14 | 1.13E-12 |
| HTR2A | 1.302675 | 1.23E-14 | 1.27E-12 |
| ANKRD11 | 1.541441 | 1.29E-14 | 1.31E-12 |
| NCKAP5 | 1.239249 | 1.40E-14 | 1.41E-12 |
| ENSAPLG00000011970 | 2.715789 | 1.85E-14 | 1.85E-12 |
| PSAT1 | -1.66829 | 2.10E-14 | 2.08E-12 |
| LINGO1 | 1.528429 | 2.23E-14 | 2.18E-12 |
| MAFB | 1.765966 | 2.36E-14 | 2.30E-12 |
| MECOM | 1.619938 | 2.67E-14 | 2.58E-12 |
| B4GALT2 | 1.563807 | 2.92E-14 | 2.79E-12 |
| ARSB | -1.44755 | 3.14E-14 | 2.97E-12 |
| SNTB2 | -1.64652 | 4.09E-14 | 3.84E-12 |
| SRF | 1.311632 | 4.34E-14 | 4.04E-12 |
| ZBTB34 | 1.39469 | 4.40E-14 | 4.07E-12 |
| TMCC1 | 1.347714 | 4.54E-14 | 4.09E-12 |
| SIX1 | 1.742615 | 4.55E-14 | 4.09E-12 |
| PREP | -1.21272 | 4.51E-14 | 4.09E-12 |
| ANGPTL4 | 1.553283 | 4.85E-14 | 4.33E-12 |
| KMT5B | 1.244823 | 4.96E-14 | 4.39E-12 |
| PCSK5 | -1.39302 | 5.00E-14 | 4.39E-12 |
| CRTAP | -1.15852 | 6.27E-14 | 5.46E-12 |
| HES6 | 2.238781 | 6.53E-14 | 5.64E-12 |
| TARS1 | -1.27035 | 7.29E-14 | 6.25E-12 |
| ELL2 | 1.570416 | 9.61E-14 | 8.18E-12 |
| GPR39 | 1.204097 | 1.01E-13 | 8.52E-12 |
| ZNF652 | 1.33209 | 1.05E-13 | 8.77E-12 |
| FBN1 | -1.92345 | 1.15E-13 | 9.56E-12 |
| SEMA3D | -1.38 | 1.24E-13 | 1.02E-11 |
| MIPEP | -1.38499 | 1.35E-13 | 1.10E-11 |
| DECR1 | -1.67862 | 1.47E-13 | 1.20E-11 |
| R3HDM1 | 1.52304 | 1.51E-13 | 1.22E-11 |
| ENSAPLG00000026952 | 1.518703 | 1.57E-13 | 1.26E-11 |
| ENSAPLG00000001129 | 1.306828 | 1.63E-13 | 1.29E-11 |
| AHCY | -1.49218 | 1.85E-13 | 1.46E-11 |
| AGO3 | 1.24288 | 2.02E-13 | 1.58E-11 |
| PLOD1 | -1.3778 | 2.22E-13 | 1.73E-11 |
| ST3GAL4 | 1.266506 | 2.30E-13 | 1.78E-11 |
| GLB1 | -1.36671 | 2.50E-13 | 1.92E-11 |
| ENSAPLG00000019985 | 1.698288 | 2.63E-13 | 2.00E-11 |
| MMP2 | -1.16016 | 2.88E-13 | 2.18E-11 |
| IL1R1 | 1.189979 | 3.03E-13 | 2.28E-11 |
| DPP7 | -1.3283 | 3.17E-13 | 2.36E-11 |
| CTSC | -1.1873 | 3.42E-13 | 2.54E-11 |
| RTN4RL2 | 1.651925 | 3.57E-13 | 2.62E-11 |
| KLF9 | 1.617019 | 4.06E-13 | 2.97E-11 |
| PTP4A1 | 1.14704 | 4.21E-13 | 3.06E-11 |
| DUSP16 | 1.373302 | 4.45E-13 | 3.21E-11 |
| SPSB1 | 1.301972 | 4.52E-13 | 3.24E-11 |
| CMIP | 1.241148 | 4.55E-13 | 3.24E-11 |
| P3H3 | -1.60448 | 4.85E-13 | 3.41E-11 |
| DUSP6 | 1.237996 | 4.83E-13 | 3.41E-11 |
| DLGAP2 | 2.044335 | 5.18E-13 | 3.62E-11 |
| BEST1 | 5.006478 | 5.24E-13 | 3.64E-11 |
| ENSAPLG00000003693 | 2.920012 | 5.38E-13 | 3.71E-11 |
| FBN2 | 1.723536 | 6.09E-13 | 4.14E-11 |
| SPRED2 | 1.399396 | 6.07E-13 | 4.14E-11 |
| HTR4 | 1.569229 | 6.23E-13 | 4.21E-11 |
| ENSAPLG00000010116 | 5.580268 | 6.67E-13 | 4.48E-11 |
| SMAD6 | 1.472805 | 8.37E-13 | 5.59E-11 |
| HSD17B4 | -1.27617 | 8.43E-13 | 5.60E-11 |
| FOS | 1.666095 | 8.72E-13 | 5.76E-11 |
| PRELID1 | 1.389188 | 9.44E-13 | 6.19E-11 |
| IP6K2 | 1.331802 | 1.04E-12 | 6.78E-11 |
| PHOSPHO1 | 2.135349 | 1.10E-12 | 7.11E-11 |
| CRELD2 | -1.51016 | 1.24E-12 | 7.97E-11 |
| ENSAPLG00000006052 | 5.028184 | 1.26E-12 | 8.07E-11 |
| EFNB1 | 1.280833 | 1.31E-12 | 8.34E-11 |
| ENSAPLG00000010314 | -1.81859 | 1.33E-12 | 8.40E-11 |
| HEXB | -1.25151 | 1.48E-12 | 9.31E-11 |
| APP | -1.13696 | 1.52E-12 | 9.50E-11 |
| PAICS | -1.18261 | 1.57E-12 | 9.80E-11 |
| DCAF5 | 1.282327 | 1.58E-12 | 9.80E-11 |
| KIAA0232 | 1.34288 | 1.90E-12 | 1.17E-10 |
| CYP24A1 | -1.44042 | 2.07E-12 | 1.27E-10 |
| PAX7 | 5.494022 | 2.12E-12 | 1.29E-10 |
| OPRD1 | 8.586176 | 2.52E-12 | 1.53E-10 |
| ENSAPLG00000015803 | 1.746749 | 3.06E-12 | 1.84E-10 |
| WNT2B | 2.257319 | 3.18E-12 | 1.90E-10 |
| GLI3 | 1.220048 | 3.24E-12 | 1.93E-10 |
| ENSAPLG00000015474 | -1.44229 | 3.68E-12 | 2.18E-10 |
| G0S2 | 1.937415 | 3.79E-12 | 2.23E-10 |
| VEGFA | 1.335876 | 3.80E-12 | 2.23E-10 |
| ENSAPLG00000009880 | 1.67683 | 3.91E-12 | 2.28E-10 |
| RHOQ | 1.603618 | 4.50E-12 | 2.61E-10 |
| LHX2 | 1.59003 | 4.57E-12 | 2.62E-10 |
| CDKN1B | 1.466906 | 4.55E-12 | 2.62E-10 |
| ENSAPLG00000008458 | 1.351327 | 4.85E-12 | 2.77E-10 |
| FRS2 | 1.44956 | 5.17E-12 | 2.94E-10 |
| GRK3 | 1.98258 | 5.77E-12 | 3.26E-10 |
| ENSAPLG00000022579 | 7.430762 | 5.81E-12 | 3.26E-10 |
| GLT8D1 | -1.18414 | 6.15E-12 | 3.44E-10 |
| STRADB | 1.372349 | 6.45E-12 | 3.58E-10 |
| NAB1 | 1.386411 | 6.49E-12 | 3.59E-10 |
| HMOX1 | 1.776531 | 6.53E-12 | 3.59E-10 |
| SHISA2 | 1.246787 | 8.23E-12 | 4.50E-10 |
| PHF21A | 1.49028 | 9.14E-12 | 4.98E-10 |
| PIK3R6 | 3.089578 | 9.35E-12 | 5.07E-10 |
| FAM214A | 1.218574 | 9.40E-12 | 5.07E-10 |
| RCL1 | -1.37611 | 1.06E-11 | 5.71E-10 |
| SLC34A2 | 2.704814 | 1.26E-11 | 6.72E-10 |
| GSN | -1.48655 | 1.28E-11 | 6.76E-10 |
| ENSAPLG00000008450 | -1.24372 | 1.28E-11 | 6.76E-10 |
| MEIS1 | 1.394996 | 1.28E-11 | 6.76E-10 |
| RNF165 | 1.430701 | 1.29E-11 | 6.78E-10 |
| SLC15A4 | -1.36679 | 1.37E-11 | 7.14E-10 |
| HSPB8 | 1.510295 | 1.39E-11 | 7.23E-10 |
| FOSL2 | 2.300961 | 1.62E-11 | 8.36E-10 |
| SPEN | 1.346661 | 1.74E-11 | 8.94E-10 |
| PDIA6 | -1.15429 | 1.79E-11 | 9.14E-10 |
| LPCAT2 | -1.21244 | 1.82E-11 | 9.29E-10 |
| ENSAPLG00000003290 | 3.381127 | 1.87E-11 | 9.46E-10 |
| RAB31 | 1.068927 | 1.95E-11 | 9.85E-10 |
| ELL | 1.352736 | 2.11E-11 | 1.06E-09 |
| GOT1 | -1.28127 | 2.13E-11 | 1.07E-09 |
| RFX5 | 1.224472 | 2.23E-11 | 1.11E-09 |
| NDUFS1 | -1.17362 | 2.25E-11 | 1.11E-09 |
| MEF2A | 1.198362 | 2.29E-11 | 1.13E-09 |
| ENSAPLG00000009135 | 1.482093 | 2.31E-11 | 1.13E-09 |
| STT3B | -1.09496 | 2.39E-11 | 1.17E-09 |
| ENSAPLG00000016465 | -1.5845 | 2.43E-11 | 1.18E-09 |
| GARS1 | -1.0965 | 2.57E-11 | 1.24E-09 |
| PDIA5 | -1.12264 | 2.70E-11 | 1.30E-09 |
| TM2D3 | -1.24887 | 2.70E-11 | 1.30E-09 |
| ENSAPLG00000013296 | 1.420731 | 2.82E-11 | 1.35E-09 |
| ALDH9A1 | -1.20123 | 2.98E-11 | 1.42E-09 |
| ENSAPLG00000022525 | 1.208154 | 2.99E-11 | 1.42E-09 |
| ATP5F1A | -1.20572 | 3.04E-11 | 1.43E-09 |
| SDF4 | -1.07712 | 3.22E-11 | 1.51E-09 |
| BMF | 1.826836 | 3.34E-11 | 1.56E-09 |
| ENOX1 | 1.296652 | 3.63E-11 | 1.69E-09 |
| PPARD | 1.111175 | 3.96E-11 | 1.84E-09 |
| GMDS | 1.102636 | 4.00E-11 | 1.85E-09 |
| PER2 | 1.123843 | 4.07E-11 | 1.87E-09 |
| FN1 | -1.19224 | 4.43E-11 | 2.03E-09 |
| CKAP4 | -1.13824 | 4.66E-11 | 2.12E-09 |
| SBNO2 | 1.763534 | 4.71E-11 | 2.14E-09 |
| MGAT4B | 1.216512 | 4.84E-11 | 2.19E-09 |
| COLEC11 | 4.335909 | 5.60E-11 | 2.52E-09 |
| TMEM203 | 1.392406 | 5.64E-11 | 2.53E-09 |
| YWHAG | 1.06906 | 6.55E-11 | 2.93E-09 |
| ENSAPLG00000000589 | -1.89071 | 6.62E-11 | 2.94E-09 |
| ENSAPLG00000015132 | -1.35599 | 6.80E-11 | 3.01E-09 |
| SDF2L1 | -1.56647 | 7.46E-11 | 3.29E-09 |
| DARS1 | -1.12452 | 7.91E-11 | 3.47E-09 |
| ENSAPLG00000031224 | -1.46066 | 8.16E-11 | 3.57E-09 |
| TRPV1 | 4.785972 | 8.22E-11 | 3.58E-09 |
| GFOD1 | 1.21008 | 8.98E-11 | 3.90E-09 |
| TRMT61A | -1.12888 | 9.07E-11 | 3.92E-09 |
| UTS2R | 2.882884 | 9.22E-11 | 3.97E-09 |
| SUCLG2 | -1.58696 | 9.35E-11 | 4.01E-09 |
| HEY1 | 1.506604 | 9.60E-11 | 4.10E-09 |
| ENSAPLG00000005553 | 3.520602 | 9.69E-11 | 4.12E-09 |
| LRRC61 | 2.690482 | 9.80E-11 | 4.15E-09 |
| ENSAPLG00000000660 | 1.812879 | 1.01E-10 | 4.25E-09 |
| NSMF | 1.297669 | 1.06E-10 | 4.46E-09 |
| ENSAPLG00000014035 | 3.590203 | 1.08E-10 | 4.53E-09 |
| ITGB1 | -1.04038 | 1.12E-10 | 4.67E-09 |
| LBR | -1.2294 | 1.26E-10 | 5.24E-09 |
| MDFI | 2.966063 | 1.29E-10 | 5.33E-09 |
| ENSAPLG00000007073 | 1.203418 | 1.33E-10 | 5.48E-09 |
| ZNF362 | 1.925684 | 1.33E-10 | 5.48E-09 |
| SPON1 | -1.18259 | 1.34E-10 | 5.49E-09 |
| AMACR | -1.68488 | 1.35E-10 | 5.51E-09 |
| MEGF9 | 1.847934 | 1.43E-10 | 5.82E-09 |
| IDH1 | -1.04705 | 1.48E-10 | 5.98E-09 |
| ENSAPLG00000024789 | -1.44166 | 1.59E-10 | 6.38E-09 |
| DES | 1.45144 | 1.59E-10 | 6.38E-09 |
| GPR162 | 1.3462 | 1.59E-10 | 6.38E-09 |
| SUMO2 | 1.367224 | 1.68E-10 | 6.72E-09 |
| ENSAPLG00000024440 | 1.075826 | 1.72E-10 | 6.82E-09 |
| TOR1B | -1.2429 | 1.76E-10 | 6.99E-09 |
| SLC25A37 | 1.74365 | 1.78E-10 | 7.04E-09 |
| GABRR2 | 5.151723 | 1.81E-10 | 7.13E-09 |
| SCPEP1 | -1.11476 | 1.91E-10 | 7.47E-09 |
| NFYC | 1.08502 | 1.96E-10 | 7.65E-09 |
| CYP4V2 | -1.7246 | 1.97E-10 | 7.65E-09 |
| PDZRN3 | -1.52735 | 2.01E-10 | 7.81E-09 |
| CERS6 | -1.26312 | 2.04E-10 | 7.88E-09 |
| RUNX1 | 1.251548 | 2.12E-10 | 8.15E-09 |
| FGF19 | 3.200464 | 2.17E-10 | 8.31E-09 |
| TACC2 | 1.174391 | 2.27E-10 | 8.68E-09 |
| PDGFA | 1.092633 | 2.33E-10 | 8.86E-09 |
| TPST1 | 1.127346 | 2.39E-10 | 9.05E-09 |
| YBX1 | 1.353157 | 2.42E-10 | 9.16E-09 |
| HIPK2 | 1.090639 | 2.50E-10 | 9.41E-09 |
| BAG5 | 1.169921 | 2.59E-10 | 9.74E-09 |
| PANX3 | 2.634459 | 2.70E-10 | 1.01E-08 |
| GDAP1L1 | 2.675983 | 2.82E-10 | 1.05E-08 |
| ATXN7 | 1.267922 | 2.85E-10 | 1.06E-08 |
| ECD | 1.251366 | 2.85E-10 | 1.06E-08 |
| ACO1 | -1.21571 | 2.89E-10 | 1.07E-08 |
| SCCPDH | -1.13015 | 3.02E-10 | 1.11E-08 |
| CDYL2 | 1.589222 | 3.16E-10 | 1.16E-08 |
| ENSAPLG00000027620 | 1.660157 | 3.16E-10 | 1.16E-08 |
| PMEPA1 | 1.324281 | 3.20E-10 | 1.17E-08 |
| KHDRBS1 | -1.10901 | 3.34E-10 | 1.21E-08 |
| APC | 1.29939 | 3.46E-10 | 1.25E-08 |
| TBCCD1 | -1.95949 | 3.48E-10 | 1.25E-08 |
| ENSAPLG00000007544 | 1.372245 | 3.51E-10 | 1.26E-08 |
| ERRFI1 | 1.412675 | 3.54E-10 | 1.27E-08 |
| TRERF1 | 1.443029 | 3.55E-10 | 1.27E-08 |
| GTF3C6 | 1.298418 | 3.63E-10 | 1.29E-08 |
| ENSAPLG00000002513 | -1.78753 | 3.65E-10 | 1.29E-08 |
| KIF14 | 4.279406 | 3.68E-10 | 1.30E-08 |
| PDIA3 | -1.24067 | 4.03E-10 | 1.42E-08 |
| BSX | 8.051357 | 4.02E-10 | 1.42E-08 |
| ENSAPLG00000027582 | 2.758299 | 4.10E-10 | 1.44E-08 |
| RNF38 | 1.176957 | 4.18E-10 | 1.46E-08 |
| DNAJC3 | -1.14667 | 4.40E-10 | 1.53E-08 |
| DOT1L | 1.284702 | 4.46E-10 | 1.55E-08 |
| SERPINI1 | -1.29222 | 4.51E-10 | 1.56E-08 |
| TAB3 | 1.375387 | 4.57E-10 | 1.57E-08 |
| HTRA1 | -1.23214 | 4.75E-10 | 1.63E-08 |
| MYO5C | 2.030945 | 4.75E-10 | 1.63E-08 |
| RPLP1 | 1.322761 | 4.88E-10 | 1.67E-08 |
| ENSAPLG00000010079 | -1.38142 | 5.00E-10 | 1.70E-08 |
| RASL11A | 3.398441 | 5.58E-10 | 1.89E-08 |
| PTEN | 1.062374 | 5.72E-10 | 1.93E-08 |
| RAB30 | 1.126871 | 5.83E-10 | 1.97E-08 |
| LANCL2 | -1.12939 | 6.15E-10 | 2.07E-08 |
| CHD7 | 1.173348 | 6.50E-10 | 2.18E-08 |
| JMJD4 | 1.305893 | 6.58E-10 | 2.20E-08 |
| EVL | 1.15524 | 6.60E-10 | 2.20E-08 |
| TKT | -1.09915 | 6.65E-10 | 2.21E-08 |
| LGMN | -1.01899 | 6.72E-10 | 2.22E-08 |
| SINHCAF | 1.015176 | 6.98E-10 | 2.30E-08 |
| ENSAPLG00000010843 | 5.364875 | 7.12E-10 | 2.34E-08 |
| ENSAPLG00000028209 | 3.009353 | 7.26E-10 | 2.38E-08 |
| SLC7A3 | -1.22909 | 7.79E-10 | 2.55E-08 |
| B3GNT9 | 1.511164 | 7.99E-10 | 2.60E-08 |
| NPEPL1 | -1.04044 | 7.98E-10 | 2.60E-08 |
| CTSH | -1.21328 | 8.12E-10 | 2.63E-08 |
| RNF126 | 1.017308 | 8.68E-10 | 2.81E-08 |
| TBCK | -1.44676 | 8.92E-10 | 2.88E-08 |
| SCN8A | 1.239641 | 8.98E-10 | 2.88E-08 |
| PLBD1 | -1.85124 | 9.69E-10 | 3.10E-08 |
| ATP1A1 | -0.98421 | 9.80E-10 | 3.13E-08 |
| WFS1 | -1.22587 | 9.92E-10 | 3.16E-08 |
| CPQ | -1.44181 | 1.01E-09 | 3.20E-08 |
| CCPG1 | -1.15366 | 1.05E-09 | 3.32E-08 |
| FSTL3 | 1.041923 | 1.07E-09 | 3.36E-08 |
| SLC43A3 | -1.33743 | 1.09E-09 | 3.44E-08 |
| ENSAPLG00000005477 | 1.487016 | 1.13E-09 | 3.53E-08 |
| ENSAPLG00000006627 | -1.07958 | 1.20E-09 | 3.73E-08 |
| HIPK3 | 1.211327 | 1.20E-09 | 3.73E-08 |
| UBE2D2 | 1.014843 | 1.20E-09 | 3.73E-08 |
| AARS1 | -1.03237 | 1.22E-09 | 3.79E-08 |
| ENSAPLG00000030885 | -1.22578 | 1.22E-09 | 3.79E-08 |
| TAF7 | -1.03975 | 1.24E-09 | 3.84E-08 |
| ATF3 | 1.407242 | 1.25E-09 | 3.84E-08 |
| XPA | 1.935231 | 1.32E-09 | 4.05E-08 |
| ACTL6A | -1.03825 | 1.39E-09 | 4.26E-08 |
| TNN | 1.829687 | 1.45E-09 | 4.41E-08 |
| SLC30A4 | 1.084553 | 1.50E-09 | 4.55E-08 |
| PRKX | 1.2244 | 1.67E-09 | 5.08E-08 |
| SUMF2 | -1.19268 | 1.68E-09 | 5.09E-08 |
| C18orf25 | 1.080385 | 1.75E-09 | 5.27E-08 |
| ENSAPLG00000029963 | 7.835947 | 1.76E-09 | 5.30E-08 |
| DNER | -1.07388 | 1.82E-09 | 5.47E-08 |
| ARHGEF33 | 7.833736 | 1.84E-09 | 5.49E-08 |
| ENSAPLG00000018355 | 1.840106 | 1.84E-09 | 5.49E-08 |
| TET1 | 1.18313 | 1.93E-09 | 5.76E-08 |
| MTNR1B | 2.002738 | 1.99E-09 | 5.90E-08 |
| ACAA2 | -1.13731 | 2.08E-09 | 6.15E-08 |
| MIDN | 1.95257 | 2.11E-09 | 6.22E-08 |
| UGDH | -1.07382 | 2.14E-09 | 6.29E-08 |
| RAI2 | 1.270443 | 2.14E-09 | 6.29E-08 |
| KPNA2 | -1.83746 | 2.21E-09 | 6.47E-08 |
| DDOST | -1.0628 | 2.34E-09 | 6.84E-08 |
| RHOBTB2 | 1.784743 | 2.44E-09 | 7.12E-08 |
| PSPH | -1.34239 | 2.59E-09 | 7.52E-08 |
| ENSAPLG00000017386 | -1.4097 | 2.60E-09 | 7.52E-08 |
| ELK3 | 1.056363 | 2.68E-09 | 7.71E-08 |
| SERPINE2 | -1.21179 | 2.67E-09 | 7.71E-08 |
| XPR1 | 1.079284 | 2.73E-09 | 7.84E-08 |
| ST3GAL1 | 0.976283 | 2.81E-09 | 8.05E-08 |
| RAB11FIP2 | 1.133487 | 3.13E-09 | 8.94E-08 |
| PPAT | -1.20024 | 3.15E-09 | 8.99E-08 |
| RIOX2 | -1.2807 | 3.39E-09 | 9.65E-08 |
| ATP2A2 | -1.27153 | 3.73E-09 | 1.06E-07 |
| ENSAPLG00000026694 | -1.11148 | 4.16E-09 | 1.18E-07 |
| TTL | 1.075837 | 4.31E-09 | 1.22E-07 |
| ACTR10 | -1.10303 | 4.38E-09 | 1.23E-07 |
| GPS1 | -0.97018 | 4.68E-09 | 1.31E-07 |
| ENSAPLG00000022307 | 4.339601 | 4.75E-09 | 1.33E-07 |
| ENSAPLG00000002309 | 1.959564 | 5.20E-09 | 1.45E-07 |
| ENSAPLG00000001088 | 5.206842 | 5.24E-09 | 1.46E-07 |
| CSRNP1 | 1.301524 | 5.28E-09 | 1.47E-07 |
| KCNB1 | 3.555418 | 5.32E-09 | 1.48E-07 |
| PXK | 1.401155 | 5.40E-09 | 1.49E-07 |
| PODN | -1.01033 | 5.78E-09 | 1.59E-07 |
| ENSAPLG00000015195 | -1.00046 | 5.79E-09 | 1.59E-07 |
| MNAT1 | -1.31686 | 5.81E-09 | 1.59E-07 |
| PTPN21 | 1.076727 | 6.09E-09 | 1.67E-07 |
| FEM1B | 1.313149 | 6.32E-09 | 1.73E-07 |
| KRT15 | 7.378309 | 6.53E-09 | 1.77E-07 |
| KLHL25 | 1.497813 | 6.50E-09 | 1.77E-07 |
| BCL9 | 1.507905 | 6.53E-09 | 1.77E-07 |
| MATN4 | 4.760661 | 6.74E-09 | 1.82E-07 |
| RPN2 | -0.99591 | 6.81E-09 | 1.84E-07 |
| BUD23 | -1.13571 | 6.96E-09 | 1.87E-07 |
| GALNT17 | 1.278567 | 7.06E-09 | 1.89E-07 |
| LPL | -1.01142 | 7.39E-09 | 1.97E-07 |
| DACT1 | -0.96773 | 7.38E-09 | 1.97E-07 |
| PTPN5 | -3.90601 | 7.44E-09 | 1.98E-07 |
| ANAPC5 | -1.15837 | 7.54E-09 | 2.00E-07 |
| PRELID3B | 0.959232 | 7.70E-09 | 2.04E-07 |
| SLC25A25 | 1.077891 | 8.20E-09 | 2.17E-07 |
| DNAAF5 | -1.86353 | 8.35E-09 | 2.20E-07 |
| PARP1 | -1.12574 | 8.34E-09 | 2.20E-07 |
| AIFM1 | -0.9606 | 8.38E-09 | 2.20E-07 |
| GLMN | -1.16417 | 8.40E-09 | 2.20E-07 |
| ENSAPLG00000017513 | -1.15695 | 8.56E-09 | 2.24E-07 |
| KIAA1522 | 1.688898 | 8.75E-09 | 2.28E-07 |
| SKP2 | -1.0626 | 8.87E-09 | 2.31E-07 |
| VWA8 | -1.82543 | 9.01E-09 | 2.34E-07 |
| SLC46A2 | -1.1905 | 9.06E-09 | 2.34E-07 |
| C1orf43 | 0.976981 | 9.26E-09 | 2.38E-07 |
| MYBPC3 | 2.193647 | 9.25E-09 | 2.38E-07 |
| CREBBP | 1.322856 | 9.44E-09 | 2.42E-07 |
| GAL3ST1 | 7.61192 | 9.46E-09 | 2.42E-07 |
| RRH | 3.064412 | 9.66E-09 | 2.47E-07 |
| PRKAR1A | -1.08304 | 9.92E-09 | 2.53E-07 |
| PDGFB | 1.525663 | 9.98E-09 | 2.54E-07 |
| MDH1 | -0.99644 | 1.01E-08 | 2.56E-07 |
| ENSAPLG00000018174 | -1.00667 | 1.01E-08 | 2.56E-07 |
| DUSP10 | 0.906331 | 1.03E-08 | 2.61E-07 |
| ENSAPLG00000011075 | -1.62589 | 1.08E-08 | 2.73E-07 |
| SOCS1 | -1.76212 | 1.11E-08 | 2.80E-07 |
| UBA2 | -1.03009 | 1.12E-08 | 2.82E-07 |
| MTFR1 | 1.254055 | 1.15E-08 | 2.88E-07 |
| NOC4L | -1.30107 | 1.16E-08 | 2.89E-07 |
| HTRA3 | -1.02099 | 1.16E-08 | 2.89E-07 |
| AK4 | 1.278805 | 1.19E-08 | 2.94E-07 |
| BAMBI | -1.13994 | 1.19E-08 | 2.94E-07 |
| TMEM204 | 1.464942 | 1.21E-08 | 3.00E-07 |
| ENSAPLG00000018141 | 1.067568 | 1.22E-08 | 3.01E-07 |
| MCM6 | -1.37081 | 1.28E-08 | 3.16E-07 |
| PIGG | -1.48142 | 1.28E-08 | 3.16E-07 |
| PAPPA | 1.747396 | 1.29E-08 | 3.16E-07 |
| ENSAPLG00000010658 | -0.9281 | 1.32E-08 | 3.24E-07 |
| NT5DC2 | 1.081797 | 1.34E-08 | 3.26E-07 |
| NCOR2 | 1.039002 | 1.35E-08 | 3.28E-07 |
| TARS3 | -1.27615 | 1.43E-08 | 3.48E-07 |
| CNOT7 | 0.895225 | 1.47E-08 | 3.58E-07 |
| EXOC1 | -1.0477 | 1.56E-08 | 3.76E-07 |
| DHRS7 | -1.31427 | 1.65E-08 | 3.98E-07 |
| MAN2B2 | -1.11865 | 1.83E-08 | 4.41E-07 |
| RPL39 | 1.409176 | 1.90E-08 | 4.56E-07 |
| ENSAPLG00000027783 | 1.119421 | 1.90E-08 | 4.57E-07 |
| CSNK1G2 | 1.044011 | 1.92E-08 | 4.60E-07 |
| SRRM3 | 7.198966 | 1.93E-08 | 4.60E-07 |
| YARS2 | -1.17636 | 1.94E-08 | 4.61E-07 |
| HES4 | 1.45325 | 1.96E-08 | 4.67E-07 |
| C1orf21 | 1.018647 | 2.00E-08 | 4.74E-07 |
| ECHS1 | -1.55878 | 2.05E-08 | 4.85E-07 |
| CASTOR2 | 1.161395 | 2.07E-08 | 4.90E-07 |
| MEF2D | 1.705494 | 2.08E-08 | 4.90E-07 |
| RLF | 1.033721 | 2.09E-08 | 4.92E-07 |
| YTHDF2 | 1.21883 | 2.11E-08 | 4.95E-07 |
| B3GNT7 | 2.823738 | 2.12E-08 | 4.97E-07 |
| ACADL | -0.99699 | 2.13E-08 | 4.99E-07 |
| HS6ST1 | 0.916413 | 2.15E-08 | 5.01E-07 |
| CMKLR1 | 2.013898 | 2.18E-08 | 5.07E-07 |
| GOT2 | -0.94619 | 2.22E-08 | 5.16E-07 |
| WDR33 | 1.029171 | 2.29E-08 | 5.30E-07 |
| SLC6A15 | -1.17756 | 2.33E-08 | 5.39E-07 |
| ENSAPLG00000027047 | -0.95223 | 2.35E-08 | 5.42E-07 |
| KDM7A | 1.00106 | 2.45E-08 | 5.65E-07 |
| AP2A2 | -0.89208 | 2.50E-08 | 5.74E-07 |
| ENSAPLG00000009852 | -1.3876 | 2.55E-08 | 5.84E-07 |
| ENSAPLG00000015291 | 1.54049 | 2.55E-08 | 5.84E-07 |
| ZBTB26 | 1.000098 | 2.56E-08 | 5.84E-07 |
| FAM126B | -1.24604 | 2.63E-08 | 6.00E-07 |
| KLHL5 | 1.199551 | 2.68E-08 | 6.08E-07 |
| GPR68 | -1.16365 | 2.69E-08 | 6.10E-07 |
| SLC35G2 | 1.013307 | 2.73E-08 | 6.17E-07 |
| ENSAPLG00000022050 | -1.38979 | 2.79E-08 | 6.31E-07 |
| MCM4 | -1.12387 | 2.82E-08 | 6.36E-07 |
| IRF1 | 2.458533 | 2.89E-08 | 6.50E-07 |
| ENSAPLG00000019156 | 1.716495 | 3.03E-08 | 6.80E-07 |
| ENSAPLG00000021952 | -1.38099 | 3.05E-08 | 6.84E-07 |
| ARHGAP12 | 0.940371 | 3.12E-08 | 6.97E-07 |
| DIPK2B | 3.780551 | 3.12E-08 | 6.97E-07 |
| NCF2 | 4.808054 | 3.42E-08 | 7.62E-07 |
| VIM | -0.96773 | 3.43E-08 | 7.63E-07 |
| ENSAPLG00000014831 | -1.01185 | 3.46E-08 | 7.67E-07 |
| GHITM | -0.9395 | 3.54E-08 | 7.83E-07 |
| UBE2E3 | 1.063118 | 3.56E-08 | 7.86E-07 |
| IGFBP7 | -1.66428 | 3.56E-08 | 7.86E-07 |
| MSH2 | -1.09261 | 3.70E-08 | 8.15E-07 |
| STOX2 | 1.178895 | 3.73E-08 | 8.19E-07 |
| CNDP2 | -0.91529 | 3.76E-08 | 8.24E-07 |
| EXOSC9 | -1.01706 | 3.78E-08 | 8.27E-07 |
| OLFML3 | -1.36191 | 3.80E-08 | 8.29E-07 |
| MTA3 | -1.14854 | 3.90E-08 | 8.49E-07 |
| ENSAPLG00000026806 | 4.223115 | 3.92E-08 | 8.52E-07 |
| SCARB2 | -1.00796 | 3.94E-08 | 8.56E-07 |
| PARP6 | 1.128217 | 3.97E-08 | 8.59E-07 |
| DUSP5 | 1.083977 | 4.03E-08 | 8.64E-07 |
| PKNOX2 | 1.372819 | 4.03E-08 | 8.64E-07 |
| CHRNA5 | 1.849625 | 4.01E-08 | 8.64E-07 |
| CSNK2A2 | 0.986912 | 4.00E-08 | 8.64E-07 |
| AZI2 | 1.035179 | 4.03E-08 | 8.64E-07 |
| ENSAPLG00000028920 | 7.058956 | 4.11E-08 | 8.79E-07 |
| PDE7B | 1.085694 | 4.37E-08 | 9.34E-07 |
| FBLN2 | -1.2421 | 4.41E-08 | 9.39E-07 |
| ARRDC3 | 1.182346 | 4.46E-08 | 9.49E-07 |
| THBS2 | -1.44249 | 4.50E-08 | 9.55E-07 |
| RASL11B | -1.48858 | 4.58E-08 | 9.71E-07 |
| YWHAQ | -0.85775 | 4.60E-08 | 9.72E-07 |
| UCHL1 | -1.34056 | 4.63E-08 | 9.74E-07 |
| ENSAPLG00000019314 | 1.450461 | 4.63E-08 | 9.74E-07 |
| CASTOR1 | -1.67153 | 4.62E-08 | 9.74E-07 |
| EIF4G2 | 1.278713 | 4.75E-08 | 9.96E-07 |
| SLC25A16 | 0.989932 | 4.84E-08 | 1.01E-06 |
| SLC30A1 | 1.058048 | 5.01E-08 | 1.05E-06 |
| ACAA1 | -1.21438 | 5.03E-08 | 1.05E-06 |
| P4HA1 | -0.93882 | 5.05E-08 | 1.05E-06 |
| PHIP | 1.013572 | 5.25E-08 | 1.09E-06 |
| EI24 | 0.979005 | 5.31E-08 | 1.10E-06 |
| MCU | -1.06351 | 5.33E-08 | 1.10E-06 |
| ENSAPLG00000015846 | 1.192228 | 5.41E-08 | 1.12E-06 |
| CDK13 | 1.00936 | 5.46E-08 | 1.13E-06 |
| ENSAPLG00000006980 | 2.327928 | 5.64E-08 | 1.16E-06 |
| FGFRL1 | 1.037491 | 5.82E-08 | 1.20E-06 |
| CYP2U1 | -1.4641 | 6.02E-08 | 1.23E-06 |
| NF2 | 0.917201 | 6.05E-08 | 1.24E-06 |
| MAT1A | -1.46421 | 6.09E-08 | 1.25E-06 |
| SH3D19 | 1.742366 | 6.21E-08 | 1.27E-06 |
| ASPH | -1.06536 | 6.35E-08 | 1.29E-06 |
| RAD54L2 | 1.195812 | 6.42E-08 | 1.30E-06 |
| CTH | -1.33409 | 6.47E-08 | 1.31E-06 |
| WDR20 | 1.008583 | 6.55E-08 | 1.33E-06 |
| ABCB6 | -1.21951 | 6.63E-08 | 1.34E-06 |
| SYNC | 0.980401 | 6.67E-08 | 1.34E-06 |
| ENSAPLG00000009942 | 6.985933 | 6.79E-08 | 1.37E-06 |
| MAS1 | 7.329796 | 6.85E-08 | 1.38E-06 |
| TPM1 | -1.03562 | 6.98E-08 | 1.40E-06 |
| PRIM2 | -1.28297 | 6.97E-08 | 1.40E-06 |
| TSPAN3 | -0.93162 | 7.18E-08 | 1.44E-06 |
| ENSAPLG00000016936 | 1.106911 | 7.21E-08 | 1.44E-06 |
| CA2 | 1.166853 | 7.31E-08 | 1.46E-06 |
| ENSAPLG00000007028 | -0.94216 | 7.33E-08 | 1.46E-06 |
| PTX3 | -1.60582 | 7.64E-08 | 1.51E-06 |
| HEXD | -1.55985 | 7.62E-08 | 1.51E-06 |
| DYSF | 1.184174 | 7.69E-08 | 1.52E-06 |
| HTR2B | 1.072081 | 7.83E-08 | 1.54E-06 |
| ENSAPLG00000020473 | -1.29369 | 7.85E-08 | 1.55E-06 |
| TRIB2 | 0.867807 | 7.95E-08 | 1.56E-06 |
| TUFT1 | 0.894881 | 8.18E-08 | 1.61E-06 |
| LONRF3 | 1.06042 | 8.42E-08 | 1.65E-06 |
| DEDD | 1.259279 | 8.45E-08 | 1.65E-06 |
| HMBOX1 | 1.011651 | 8.59E-08 | 1.68E-06 |
| ENSAPLG00000030007 | -1.23386 | 8.61E-08 | 1.68E-06 |
| ABHD13 | 0.890778 | 8.69E-08 | 1.69E-06 |
| INHBB | 2.357534 | 8.75E-08 | 1.70E-06 |
| ENSAPLG00000030664 | 2.544803 | 8.74E-08 | 1.70E-06 |
| PHF12 | 1.325582 | 9.30E-08 | 1.80E-06 |
| HRAS | 1.007233 | 9.37E-08 | 1.81E-06 |
| COMTD1 | -1.05317 | 9.61E-08 | 1.85E-06 |
| LONP2 | -1.03198 | 9.73E-08 | 1.87E-06 |
| ACSL3 | -0.8843 | 9.76E-08 | 1.87E-06 |
| RCAN3 | 0.886022 | 9.83E-08 | 1.88E-06 |
| MDM4 | 1.212153 | 9.94E-08 | 1.90E-06 |
| ENSAPLG00000017191 | 1.207365 | 9.94E-08 | 1.90E-06 |
| GNAI1 | -0.93157 | 1.02E-07 | 1.94E-06 |
| PIP4K2A | 0.951489 | 1.03E-07 | 1.97E-06 |
| MYG1 | -1.34253 | 1.05E-07 | 2.00E-06 |
| ENSAPLG00000021054 | -1.12798 | 1.07E-07 | 2.04E-06 |
| FSTL1 | -0.99389 | 1.08E-07 | 2.04E-06 |
| ENDOG | -1.01451 | 1.10E-07 | 2.08E-06 |
| CTSO | -1.20317 | 1.12E-07 | 2.11E-06 |
| AKR7L | -1.07158 | 1.12E-07 | 2.11E-06 |
| ENSAPLG00000005235 | 1.075018 | 1.13E-07 | 2.13E-06 |
| PLTP | -1.26737 | 1.13E-07 | 2.13E-06 |
| CCN1 | -0.94425 | 1.14E-07 | 2.14E-06 |
| ATP6V1H | -0.95522 | 1.16E-07 | 2.17E-06 |
| PFKP | -0.88395 | 1.19E-07 | 2.23E-06 |
| BCAP29 | -1.01263 | 1.20E-07 | 2.25E-06 |
| HELLS | -1.26107 | 1.25E-07 | 2.32E-06 |
| SMYD2 | -1.04305 | 1.27E-07 | 2.37E-06 |
| CXCL12 | 1.144468 | 1.28E-07 | 2.37E-06 |
| TNS1 | 1.35479 | 1.35E-07 | 2.50E-06 |
| FKBP5 | 1.058852 | 1.43E-07 | 2.65E-06 |
| ENSAPLG00000008364 | -0.87981 | 1.45E-07 | 2.68E-06 |
| VPS54 | -1.09384 | 1.48E-07 | 2.72E-06 |
| ERP29 | -1.03469 | 1.51E-07 | 2.77E-06 |
| WNT5B | -0.88 | 1.55E-07 | 2.84E-06 |
| NAE1 | -0.96293 | 1.57E-07 | 2.89E-06 |
| ENSAPLG00000018235 | 1.479785 | 1.60E-07 | 2.93E-06 |
| ATP5F1B | -0.89316 | 1.64E-07 | 2.99E-06 |
| SEPTIN9 | 1.40871 | 1.66E-07 | 3.02E-06 |
| SURF1 | -1.12697 | 1.66E-07 | 3.03E-06 |
| MYBL2 | 1.193528 | 1.68E-07 | 3.05E-06 |
| POLR3B | -1.0747 | 1.69E-07 | 3.07E-06 |
| NMD3 | -0.8972 | 1.71E-07 | 3.09E-06 |
| GSE1 | 1.499305 | 1.71E-07 | 3.09E-06 |
| PCCA | -1.09761 | 1.72E-07 | 3.11E-06 |
| CHSY1 | 1.424534 | 1.72E-07 | 3.11E-06 |
| BCHE | -1.42427 | 1.74E-07 | 3.14E-06 |
| RAP1GDS1 | -0.84284 | 1.77E-07 | 3.18E-06 |
| ENSAPLG00000003517 | 0.903545 | 1.78E-07 | 3.20E-06 |
| FOLH1 | -1.1648 | 1.83E-07 | 3.27E-06 |
| PAPSS1 | -0.87775 | 1.85E-07 | 3.31E-06 |
| MTF1 | 0.882125 | 1.86E-07 | 3.31E-06 |
| ENSAPLG00000017536 | 3.517483 | 1.85E-07 | 3.31E-06 |
| NUCB2 | -0.96206 | 1.90E-07 | 3.39E-06 |
| TAX1BP3 | 0.931912 | 1.91E-07 | 3.40E-06 |
| ENSAPLG00000020033 | 1.372992 | 1.92E-07 | 3.41E-06 |
| TMEM184B | 1.093545 | 1.95E-07 | 3.46E-06 |
| EHBP1 | 0.98956 | 1.97E-07 | 3.48E-06 |
| RHOU | 1.490485 | 1.97E-07 | 3.48E-06 |
| ENSAPLG00000019053 | 1.322291 | 1.98E-07 | 3.48E-06 |
| KXD1 | 1.082681 | 2.00E-07 | 3.52E-06 |
| MGAT2 | -0.99179 | 2.00E-07 | 3.52E-06 |
| OTOR | 7.189284 | 2.01E-07 | 3.53E-06 |
| SLC25A39 | 0.913294 | 2.02E-07 | 3.54E-06 |
| ENSAPLG00000028455 | 1.518277 | 2.05E-07 | 3.58E-06 |
| MAFK | 1.273174 | 2.07E-07 | 3.61E-06 |
| ENSAPLG00000021067 | 2.689363 | 2.10E-07 | 3.66E-06 |
| ENSAPLG00000003255 | -1.25299 | 2.14E-07 | 3.72E-06 |
| AGL | -1.15348 | 2.14E-07 | 3.72E-06 |
| DGKD | 0.901039 | 2.15E-07 | 3.74E-06 |
| SPRED1 | 0.890141 | 2.19E-07 | 3.80E-06 |
| SENP5 | 0.814167 | 2.21E-07 | 3.82E-06 |
| CDHR1 | 7.104399 | 2.23E-07 | 3.84E-06 |
| ENSAPLG00000007778 | -1.02837 | 2.23E-07 | 3.85E-06 |
| USP53 | 1.21498 | 2.24E-07 | 3.86E-06 |
| GATB | -1.78646 | 2.29E-07 | 3.93E-06 |
| POFUT1 | -1.06506 | 2.29E-07 | 3.93E-06 |
| RIC3 | -1.24155 | 2.31E-07 | 3.96E-06 |
| KCNS1 | 4.526742 | 2.38E-07 | 4.07E-06 |
| KANSL1 | 1.537603 | 2.41E-07 | 4.11E-06 |
| P3H1 | -1.0731 | 2.46E-07 | 4.20E-06 |
| MORC2 | 1.285283 | 2.47E-07 | 4.20E-06 |
| PI4KB | 1.219723 | 2.50E-07 | 4.24E-06 |
| WDR77 | -0.93442 | 2.50E-07 | 4.24E-06 |
| UBL3 | 0.98474 | 2.50E-07 | 4.24E-06 |
| WDR53 | -1.05558 | 2.51E-07 | 4.25E-06 |
| DLX5 | 1.220578 | 2.52E-07 | 4.26E-06 |
| FGFR1 | 0.86915 | 2.56E-07 | 4.31E-06 |
| WEE1 | 1.342896 | 2.57E-07 | 4.32E-06 |
| TRIM45 | -1.8778 | 2.58E-07 | 4.34E-06 |
| ENSAPLG00000007227 | 3.26385 | 2.62E-07 | 4.40E-06 |
| MCM2 | -1.25984 | 2.67E-07 | 4.47E-06 |
| ZPLD1 | 1.34412 | 2.67E-07 | 4.47E-06 |
| TRRAP | 1.107088 | 2.72E-07 | 4.55E-06 |
| TMEM260 | -1.31438 | 2.75E-07 | 4.59E-06 |
| ACAT1 | -0.95919 | 2.77E-07 | 4.61E-06 |
| SERINC1 | -0.85505 | 2.78E-07 | 4.61E-06 |
| MPI | -1.01573 | 2.78E-07 | 4.61E-06 |
| MLLT6 | 1.493835 | 2.86E-07 | 4.74E-06 |
| EP300 | 1.041331 | 2.87E-07 | 4.75E-06 |
| KCNJ4 | 4.052922 | 2.87E-07 | 4.75E-06 |
| CDC23 | -0.96718 | 2.88E-07 | 4.75E-06 |
| ACAT2 | -1.06491 | 2.88E-07 | 4.75E-06 |
| ENSAPLG00000016374 | 1.007931 | 3.00E-07 | 4.94E-06 |
| COLGALT1 | -1.17463 | 3.04E-07 | 5.00E-06 |
| TACC1 | 1.08106 | 3.05E-07 | 5.01E-06 |
| ENSAPLG00000018153 | 0.937401 | 3.07E-07 | 5.03E-06 |
| ENSAPLG00000008838 | 0.851701 | 3.09E-07 | 5.05E-06 |
| HSD17B12 | -0.86446 | 3.10E-07 | 5.06E-06 |
| ATAD1 | -0.9349 | 3.13E-07 | 5.10E-06 |
| APC2 | 7.051962 | 3.15E-07 | 5.13E-06 |
| FAM149B1 | 1.369512 | 3.15E-07 | 5.13E-06 |
| RAB3IL1 | 1.043085 | 3.20E-07 | 5.19E-06 |
| SDR42E1 | -1.54387 | 3.23E-07 | 5.23E-06 |
| NDC1 | -0.98497 | 3.23E-07 | 5.23E-06 |
| RTCA | -0.97718 | 3.22E-07 | 5.23E-06 |
| WIPI1 | 0.833873 | 3.28E-07 | 5.30E-06 |
| DYRK2 | 1.020367 | 3.34E-07 | 5.38E-06 |
| EMC7 | -0.96513 | 3.36E-07 | 5.40E-06 |
| TNFSF8 | 7.085821 | 3.38E-07 | 5.43E-06 |
| EXTL3 | 1.10499 | 3.51E-07 | 5.62E-06 |
| ZFP36L1 | 1.297999 | 3.51E-07 | 5.62E-06 |
| NNT | -1.31111 | 3.56E-07 | 5.70E-06 |
| SERPINF1 | -1.15686 | 3.63E-07 | 5.80E-06 |
| HP1BP3 | -0.9144 | 3.64E-07 | 5.80E-06 |
| ENSAPLG00000008343 | 1.88152 | 3.72E-07 | 5.92E-06 |
| SLC35A3 | -1.01268 | 3.75E-07 | 5.97E-06 |
| PCYT2 | 0.867384 | 3.80E-07 | 6.03E-06 |
| RNF182 | 2.256905 | 3.99E-07 | 6.32E-06 |
| DDX49 | -1.01257 | 3.99E-07 | 6.32E-06 |
| WWP1 | 1.153107 | 4.04E-07 | 6.39E-06 |
| ENSAPLG00000006443 | -0.86461 | 4.05E-07 | 6.39E-06 |
| SPTLC1 | -0.93492 | 4.07E-07 | 6.41E-06 |
| DAB2IP | 1.474468 | 4.08E-07 | 6.42E-06 |
| NKX3-2 | 1.477992 | 4.09E-07 | 6.42E-06 |
| PGM2 | -0.89216 | 4.15E-07 | 6.51E-06 |
| RSRP1 | 1.241557 | 4.23E-07 | 6.64E-06 |
| ENSAPLG00000013227 | 0.986428 | 4.25E-07 | 6.66E-06 |
| FAP | -0.96383 | 4.34E-07 | 6.79E-06 |
| TK2 | 1.006369 | 4.38E-07 | 6.83E-06 |
| PLAGL1 | 1.030352 | 4.39E-07 | 6.84E-06 |
| EIF4EBP1 | 1.13815 | 4.49E-07 | 6.99E-06 |
| PCMTD1 | 0.92915 | 4.51E-07 | 7.01E-06 |
| FIG4 | -1.28187 | 4.54E-07 | 7.05E-06 |
| HADHA | -0.80122 | 4.56E-07 | 7.07E-06 |
| WDR75 | -0.94505 | 4.80E-07 | 7.43E-06 |
| CTDSPL | 1.137636 | 4.81E-07 | 7.44E-06 |
| EBAG9 | 1.010105 | 4.89E-07 | 7.54E-06 |
| GPI | -0.89672 | 4.99E-07 | 7.68E-06 |
| ENSAPLG00000008579 | -0.91623 | 5.09E-07 | 7.82E-06 |
| HADH | -0.93208 | 5.17E-07 | 7.94E-06 |
| SKI | 1.272718 | 5.18E-07 | 7.94E-06 |
| RPL3L | 2.641828 | 5.19E-07 | 7.95E-06 |
| RAMP3 | 1.204367 | 5.23E-07 | 8.01E-06 |
| AGPAT4 | -1.06954 | 5.32E-07 | 8.13E-06 |
| DLD | -0.85974 | 5.37E-07 | 8.19E-06 |
| PTGER3 | 3.482524 | 5.54E-07 | 8.44E-06 |
| ENSAPLG00000026048 | -1.09906 | 5.71E-07 | 8.68E-06 |
| ENSAPLG00000001512 | 1.933583 | 5.87E-07 | 8.90E-06 |
| GFPT2 | -1.04098 | 5.86E-07 | 8.90E-06 |
| TXNRD3 | -0.91651 | 5.93E-07 | 8.99E-06 |
| ZCCHC24 | 0.951758 | 5.97E-07 | 9.04E-06 |
| ACTG2 | -1.19001 | 5.99E-07 | 9.05E-06 |
| TMEM250 | 1.143405 | 6.02E-07 | 9.09E-06 |
| PCYOX1L | -1.15507 | 6.06E-07 | 9.14E-06 |
| EGFLAM | -1.11741 | 6.15E-07 | 9.25E-06 |
| ENSAPLG00000013374 | 0.914096 | 6.23E-07 | 9.36E-06 |
| SYT17 | 0.953335 | 6.27E-07 | 9.40E-06 |
| WTIP | 1.07919 | 6.28E-07 | 9.41E-06 |
| KIN | -1.25656 | 6.30E-07 | 9.42E-06 |
| ENSAPLG00000016336 | 4.09568 | 6.51E-07 | 9.73E-06 |
| MCF2L2 | 3.005826 | 6.56E-07 | 9.79E-06 |
| MMUT | -0.97527 | 6.66E-07 | 9.93E-06 |
| ASPG | -1.20329 | 6.75E-07 | 1.00E-05 |
| USP4 | -0.98587 | 6.86E-07 | 1.02E-05 |
| FRS3 | 1.650909 | 6.85E-07 | 1.02E-05 |
| ENSAPLG00000004580 | 1.374434 | 6.89E-07 | 1.02E-05 |
| SMIM14 | 0.93703 | 6.88E-07 | 1.02E-05 |
| CTNNA1 | -0.79063 | 6.91E-07 | 1.02E-05 |
| RUNX2 | 1.335763 | 6.93E-07 | 1.02E-05 |
| PSAP | -1.04793 | 6.93E-07 | 1.02E-05 |
| LPAR4 | 1.461678 | 6.97E-07 | 1.03E-05 |
| ALDH18A1 | -0.84094 | 7.01E-07 | 1.03E-05 |
| CRB2 | 1.633466 | 7.06E-07 | 1.04E-05 |
| ZC3H12B | 1.182363 | 7.09E-07 | 1.04E-05 |
| SPG7 | 0.806375 | 7.12E-07 | 1.04E-05 |
| CBR4 | -1.0766 | 7.18E-07 | 1.05E-05 |
| ENSAPLG00000013478 | -0.95789 | 7.46E-07 | 1.09E-05 |
| JAM3 | -1.07084 | 7.54E-07 | 1.10E-05 |
| LTA4H | -0.82011 | 7.55E-07 | 1.10E-05 |
| SRPX2 | -1.15632 | 7.59E-07 | 1.10E-05 |
| AFAP1 | -0.85084 | 7.90E-07 | 1.15E-05 |
| KRT75 | -2.28053 | 7.89E-07 | 1.15E-05 |
| AGK | -1.06558 | 7.96E-07 | 1.15E-05 |
| NUMA1 | 1.233519 | 8.03E-07 | 1.16E-05 |
| ZDHHC13 | -0.99977 | 8.07E-07 | 1.16E-05 |
| UTP25 | -0.94794 | 8.07E-07 | 1.16E-05 |
| ABLIM2 | 1.100469 | 8.09E-07 | 1.17E-05 |
| IMPDH2 | -0.80691 | 8.20E-07 | 1.18E-05 |
| ANXA6 | -0.82704 | 8.23E-07 | 1.18E-05 |
| SHROOM3 | 1.182453 | 8.25E-07 | 1.18E-05 |
| ZFHX4 | 0.938002 | 8.38E-07 | 1.20E-05 |
| GRIK3 | 4.537488 | 8.67E-07 | 1.24E-05 |
| ENSAPLG00000024348 | -1.2155 | 8.82E-07 | 1.26E-05 |
| SEL1L | -1.22478 | 8.84E-07 | 1.26E-05 |
| ARNTL | -1.07438 | 8.87E-07 | 1.27E-05 |
| GXYLT2 | 0.964242 | 8.98E-07 | 1.28E-05 |
| ENSAPLG00000006772 | 2.499659 | 9.03E-07 | 1.28E-05 |
| YTHDF1 | 1.226615 | 9.16E-07 | 1.30E-05 |
| ABCC4 | -1.09247 | 9.25E-07 | 1.31E-05 |
| PSMD10 | -1.09041 | 9.31E-07 | 1.32E-05 |
| GALNT12 | -1.64947 | 9.65E-07 | 1.37E-05 |
| CAT | -1.05162 | 9.79E-07 | 1.38E-05 |
| ENSAPLG00000016255 | 0.811821 | 9.84E-07 | 1.39E-05 |
| FBRSL1 | 0.942027 | 9.90E-07 | 1.40E-05 |
| UBE2H | 0.990654 | 1.00E-06 | 1.41E-05 |
| ZDHHC3 | 0.886714 | 1.03E-06 | 1.45E-05 |
| AIMP2 | -0.9394 | 1.06E-06 | 1.48E-05 |
| FMN1 | 1.051137 | 1.06E-06 | 1.48E-05 |
| PEX13 | -1.05075 | 1.10E-06 | 1.54E-05 |
| PMS2 | -0.90529 | 1.11E-06 | 1.56E-05 |
| NUDT3 | 1.621045 | 1.13E-06 | 1.58E-05 |
| MSRB3 | 1.046385 | 1.13E-06 | 1.58E-05 |
| EZR | -0.77608 | 1.15E-06 | 1.60E-05 |
| TXNDC15 | -0.98671 | 1.15E-06 | 1.60E-05 |
| PLK2 | -0.79839 | 1.16E-06 | 1.61E-05 |
| IDH3A | -0.8144 | 1.16E-06 | 1.61E-05 |
| SIDT2 | 1.102888 | 1.16E-06 | 1.61E-05 |
| GMPS | -0.81361 | 1.17E-06 | 1.63E-05 |
| SUMO1 | 0.922385 | 1.18E-06 | 1.64E-05 |
| GJA1 | -0.85037 | 1.21E-06 | 1.67E-05 |
| FZD9 | 0.993975 | 1.24E-06 | 1.71E-05 |
| RHBDD1 | -1.52498 | 1.26E-06 | 1.74E-05 |
| GFM1 | -0.86617 | 1.27E-06 | 1.74E-05 |
| TRMT10A | -1.0954 | 1.27E-06 | 1.75E-05 |
| GGT7 | 3.323362 | 1.27E-06 | 1.75E-05 |
| PTP4A2 | 0.908005 | 1.28E-06 | 1.76E-05 |
| SPSB4 | -0.98715 | 1.29E-06 | 1.76E-05 |
| RWDD4 | 0.940792 | 1.30E-06 | 1.77E-05 |
| ENSAPLG00000009022 | 0.907177 | 1.31E-06 | 1.79E-05 |
| OGFOD1 | -0.95445 | 1.32E-06 | 1.79E-05 |
| DRG1 | -0.89962 | 1.35E-06 | 1.83E-05 |
| ALG12 | -0.7777 | 1.36E-06 | 1.85E-05 |
| PYROXD1 | -1.30048 | 1.39E-06 | 1.89E-05 |
| BLMH | -0.78559 | 1.41E-06 | 1.92E-05 |
| SHOC2 | 0.848084 | 1.43E-06 | 1.93E-05 |
| NCLN | -0.80922 | 1.43E-06 | 1.93E-05 |
| LMBRD1 | -0.88889 | 1.43E-06 | 1.93E-05 |
| MTUS2 | 2.867421 | 1.44E-06 | 1.95E-05 |
| SLC25A4 | -1.04671 | 1.44E-06 | 1.95E-05 |
| ENSAPLG00000029546 | -0.83319 | 1.46E-06 | 1.96E-05 |
| VAV2 | 0.932308 | 1.47E-06 | 1.97E-05 |
| MTTP | 6.848201 | 1.47E-06 | 1.98E-05 |
| USPL1 | 1.55018 | 1.48E-06 | 1.98E-05 |
| PRKCD | -0.87801 | 1.49E-06 | 1.99E-05 |
| VWF | 1.016526 | 1.49E-06 | 1.99E-05 |
| PLRG1 | -0.91069 | 1.48E-06 | 1.99E-05 |
| ODF2 | 1.035552 | 1.49E-06 | 1.99E-05 |
| ENSAPLG00000008168 | 1.4438 | 1.50E-06 | 2.00E-05 |
| CTNNBL1 | -0.8478 | 1.51E-06 | 2.01E-05 |
| C10orf71 | 1.006177 | 1.51E-06 | 2.01E-05 |
| IPO5 | -0.8223 | 1.52E-06 | 2.02E-05 |
| HEXIM1 | 1.321665 | 1.53E-06 | 2.03E-05 |
| CD109 | -2.13262 | 1.54E-06 | 2.04E-05 |
| ENSAPLG00000005387 | 1.048329 | 1.55E-06 | 2.06E-05 |
| PHF6 | -0.85037 | 1.58E-06 | 2.08E-05 |
| INTS14 | -0.96637 | 1.61E-06 | 2.13E-05 |
| AP5M1 | 0.926868 | 1.62E-06 | 2.13E-05 |
| AVL9 | -1.09567 | 1.62E-06 | 2.13E-05 |
| HSDL2 | -1.04045 | 1.62E-06 | 2.14E-05 |
| MTERF3 | -0.96803 | 1.63E-06 | 2.14E-05 |
| ARNTL2 | 0.961376 | 1.64E-06 | 2.16E-05 |
| ENSAPLG00000002228 | -0.98264 | 1.65E-06 | 2.16E-05 |
| DCP1A | 0.934907 | 1.65E-06 | 2.16E-05 |
| MYH10 | -0.86066 | 1.66E-06 | 2.17E-05 |
| BMPER | -1.1173 | 1.67E-06 | 2.19E-05 |
| RGL1 | 1.175118 | 1.69E-06 | 2.21E-05 |
| UNC50 | -0.98785 | 1.72E-06 | 2.24E-05 |
| PHLDA2 | 1.137312 | 1.76E-06 | 2.28E-05 |
| ADA2 | -1.83803 | 1.77E-06 | 2.30E-05 |
| SERPINB1 | 1.631446 | 1.78E-06 | 2.31E-05 |
| BDNF | 0.994638 | 1.81E-06 | 2.34E-05 |
| ENSAPLG00000026183 | -1.13443 | 1.81E-06 | 2.35E-05 |
| MRPS27 | -0.97661 | 1.83E-06 | 2.37E-05 |
| MAP2K3 | 0.885778 | 1.83E-06 | 2.37E-05 |
| ENSAPLG00000009366 | 1.104712 | 1.86E-06 | 2.40E-05 |
| PIP5K1B | 1.322112 | 1.87E-06 | 2.41E-05 |
| SEC61A2 | -0.83377 | 1.87E-06 | 2.41E-05 |
| TNRC6A | 1.206249 | 1.90E-06 | 2.44E-05 |
| COQ6 | -0.93683 | 1.90E-06 | 2.44E-05 |
| SLC17A5 | -1.03517 | 1.91E-06 | 2.45E-05 |
| ENSAPLG00000024724 | 1.083223 | 1.93E-06 | 2.47E-05 |
| ENSAPLG00000011828 | 0.968065 | 1.93E-06 | 2.48E-05 |
| PRDM4 | 0.922412 | 1.94E-06 | 2.48E-05 |
| FNIP1 | 1.262075 | 1.95E-06 | 2.50E-05 |
| CNOT4 | 0.994955 | 1.98E-06 | 2.52E-05 |
| ELP3 | -0.87878 | 2.01E-06 | 2.56E-05 |
| ENSAPLG00000013634 | -0.86844 | 2.04E-06 | 2.60E-05 |
| SIL1 | -1.21067 | 2.06E-06 | 2.61E-05 |
| HIVEP3 | 1.183418 | 2.06E-06 | 2.61E-05 |
| KLF13 | 1.220399 | 2.07E-06 | 2.62E-05 |
| ENSAPLG00000015183 | 0.991234 | 2.09E-06 | 2.65E-05 |
| ENSAPLG00000029052 | 1.00332 | 2.10E-06 | 2.66E-05 |
| ITGAV | -1.11976 | 2.11E-06 | 2.67E-05 |
| INSR | 1.688753 | 2.18E-06 | 2.75E-05 |
| GSS | -1.27252 | 2.18E-06 | 2.75E-05 |
| KRT12 | 6.387996 | 2.22E-06 | 2.79E-05 |
| COPG2 | -0.83925 | 2.22E-06 | 2.80E-05 |
| RD3 | 3.865547 | 2.24E-06 | 2.82E-05 |
| RRBP1 | -0.82684 | 2.26E-06 | 2.84E-05 |
| ENSAPLG00000009692 | 1.062581 | 2.27E-06 | 2.84E-05 |
| ENSAPLG00000026526 | -0.84513 | 2.27E-06 | 2.84E-05 |
| ERP44 | -0.90815 | 2.28E-06 | 2.85E-05 |
| RSL1D1 | -0.90992 | 2.30E-06 | 2.87E-05 |
| GRIA2 | -1.13677 | 2.31E-06 | 2.89E-05 |
| WT1 | 1.772425 | 2.33E-06 | 2.90E-05 |
| ANKRD12 | 1.175797 | 2.36E-06 | 2.94E-05 |
| ITGA4 | -1.26788 | 2.43E-06 | 3.02E-05 |
| ENSAPLG00000013064 | 0.960377 | 2.43E-06 | 3.02E-05 |
| EPHX1 | -1.28719 | 2.50E-06 | 3.10E-05 |
| PPIL4 | -1.23868 | 2.50E-06 | 3.10E-05 |
| ENSAPLG00000018690 | 0.990926 | 2.51E-06 | 3.11E-05 |
| RRAGD | 0.77978 | 2.52E-06 | 3.11E-05 |
| CAV3 | 6.71647 | 2.54E-06 | 3.13E-05 |
| ZWILCH | -0.97352 | 2.56E-06 | 3.16E-05 |
| SPPL2A | -0.747 | 2.58E-06 | 3.18E-05 |
| PHETA2 | 1.589535 | 2.60E-06 | 3.20E-05 |
| ZDHHC17 | 0.802549 | 2.65E-06 | 3.26E-05 |
| ENSAPLG00000002850 | -0.90855 | 2.66E-06 | 3.26E-05 |
| ZNF384 | 1.420644 | 2.71E-06 | 3.33E-05 |
| TOMM40L | 1.242896 | 2.74E-06 | 3.35E-05 |
| STAR | 4.357496 | 2.76E-06 | 3.37E-05 |
| OTX2 | 6.337214 | 2.76E-06 | 3.37E-05 |
| CCN3 | -1.58118 | 2.80E-06 | 3.42E-05 |
| LRPAP1 | -1.17431 | 2.80E-06 | 3.42E-05 |
| TMEM131L | -1.07722 | 2.82E-06 | 3.44E-05 |
| SALL4 | 1.614535 | 2.85E-06 | 3.47E-05 |
| ENSAPLG00000006104 | 1.652091 | 2.87E-06 | 3.49E-05 |
| GALNS | -0.79775 | 2.91E-06 | 3.54E-05 |
| CERT1 | -1.00458 | 2.99E-06 | 3.63E-05 |
| CYP51A1 | -0.94283 | 3.00E-06 | 3.63E-05 |
| ENSAPLG00000024644 | 6.297489 | 3.03E-06 | 3.67E-05 |
| RPL37 | 0.903622 | 3.06E-06 | 3.70E-05 |
| ENSAPLG00000028526 | -1.01042 | 3.06E-06 | 3.70E-05 |
| STC2 | 0.890649 | 3.07E-06 | 3.71E-05 |
| MAML1 | 1.841911 | 3.11E-06 | 3.75E-05 |
| ENSAPLG00000026428 | 1.11112 | 3.13E-06 | 3.77E-05 |
| ENSAPLG00000007015 | -0.81819 | 3.14E-06 | 3.78E-05 |
| GADL1 | 2.085491 | 3.15E-06 | 3.78E-05 |
| ACSS2 | -1.24459 | 3.16E-06 | 3.79E-05 |
| ENSAPLG00000011402 | -0.77934 | 3.17E-06 | 3.80E-05 |
| DLC1 | 0.884477 | 3.25E-06 | 3.89E-05 |
| ATG9B | 1.12057 | 3.31E-06 | 3.96E-05 |
| COG6 | -0.96371 | 3.32E-06 | 3.97E-05 |
| C1QTNF4 | -1.61831 | 3.33E-06 | 3.97E-05 |
| TAB1 | -0.91573 | 3.34E-06 | 3.98E-05 |
| RDH10 | -0.90466 | 3.36E-06 | 4.00E-05 |
| MSX1 | 0.994283 | 3.37E-06 | 4.00E-05 |
| TBC1D19 | -1.37956 | 3.43E-06 | 4.07E-05 |
| PRTG | 2.450934 | 3.58E-06 | 4.24E-05 |
| SLC25A46 | -0.95056 | 3.59E-06 | 4.25E-05 |
| EIF4A3 | -0.85067 | 3.63E-06 | 4.29E-05 |
| CERCAM | -0.86637 | 3.63E-06 | 4.29E-05 |
| GTF3C3 | -1.07198 | 3.68E-06 | 4.35E-05 |
| ITM2B | -0.83205 | 3.70E-06 | 4.36E-05 |
| ALG6 | -0.92444 | 3.70E-06 | 4.36E-05 |
| TNFAIP6 | -1.04933 | 3.71E-06 | 4.36E-05 |
| NIBAN2 | 0.969237 | 3.79E-06 | 4.45E-05 |
| FBXO30 | -1.10794 | 3.79E-06 | 4.45E-05 |
| FAM120B | -1.29423 | 3.81E-06 | 4.47E-05 |
| ENOPH1 | -0.93095 | 3.85E-06 | 4.51E-05 |
| IVD | -0.89645 | 3.97E-06 | 4.65E-05 |
| ENSAPLG00000003957 | 5.544549 | 3.99E-06 | 4.67E-05 |
| LRIT1 | 6.643794 | 4.01E-06 | 4.68E-05 |
| CIAO3 | -0.92241 | 4.00E-06 | 4.68E-05 |
| PLXND1 | 1.472683 | 4.04E-06 | 4.72E-05 |
| PLAA | -0.84768 | 4.12E-06 | 4.80E-05 |
| POC1A | -1.17793 | 4.13E-06 | 4.81E-05 |
| GSTCD | -1.27404 | 4.20E-06 | 4.89E-05 |
| MRPL3 | -1.05429 | 4.22E-06 | 4.90E-05 |
| ARID3B | 1.478305 | 4.23E-06 | 4.91E-05 |
| VPS35 | -0.81804 | 4.25E-06 | 4.93E-05 |
| TRMT2A | -1.0197 | 4.32E-06 | 5.00E-05 |
| TEKT5 | -1.70084 | 4.37E-06 | 5.06E-05 |
| API5 | -0.79695 | 4.41E-06 | 5.09E-05 |
| ENSAPLG00000018136 | -1.15435 | 4.41E-06 | 5.09E-05 |
| EED | -0.84069 | 4.43E-06 | 5.10E-05 |
| ERGIC2 | 0.700726 | 4.45E-06 | 5.12E-05 |
| ADAM10 | -0.76737 | 4.45E-06 | 5.12E-05 |
| RPS11 | 0.997041 | 4.51E-06 | 5.18E-05 |
| SLCO4A1 | -1.07219 | 4.53E-06 | 5.20E-05 |
| SERINC3 | -0.7238 | 4.53E-06 | 5.20E-05 |
| SCAF4 | 0.895717 | 4.54E-06 | 5.20E-05 |
| SATB1 | 1.072445 | 4.55E-06 | 5.21E-05 |
| GPBP1L1 | 0.773085 | 4.61E-06 | 5.27E-05 |
| PRELID3A | 0.801415 | 4.66E-06 | 5.31E-05 |
| ENSAPLG00000011334 | 2.395381 | 4.65E-06 | 5.31E-05 |
| MAEA | -0.85274 | 4.65E-06 | 5.31E-05 |
| SLC1A4 | -1.00319 | 4.69E-06 | 5.33E-05 |
| RASSF9 | -1.05076 | 4.75E-06 | 5.39E-05 |
| CAB39L | 0.903909 | 4.75E-06 | 5.39E-05 |
| OTUD6B | 0.738656 | 4.74E-06 | 5.39E-05 |
| SLC9A2 | 6.274938 | 4.77E-06 | 5.41E-05 |
| SLC25A33 | -0.8978 | 4.79E-06 | 5.43E-05 |
| GEMIN5 | -0.85162 | 4.83E-06 | 5.46E-05 |
| EXOSC3 | -1.19677 | 4.89E-06 | 5.52E-05 |
| APLP2 | -0.74229 | 4.90E-06 | 5.53E-05 |
| MTFR1L | 0.850708 | 4.90E-06 | 5.53E-05 |
| LANCL1 | -0.83194 | 4.95E-06 | 5.58E-05 |
| TCF7L2 | 1.153538 | 4.98E-06 | 5.60E-05 |
| ENSAPLG00000031160 | 1.002255 | 4.98E-06 | 5.60E-05 |
| NASP | -1.10422 | 5.06E-06 | 5.69E-05 |
| NRG1 | 1.439929 | 5.15E-06 | 5.78E-05 |
| TBX18 | 1.112352 | 5.24E-06 | 5.87E-05 |
| NPAS2 | 1.229225 | 5.36E-06 | 6.00E-05 |
| ENSAPLG00000025088 | 1.819655 | 5.41E-06 | 6.05E-05 |
| MMP23B | 0.89776 | 5.44E-06 | 6.08E-05 |
| LONRF1 | 0.866083 | 5.47E-06 | 6.10E-05 |
| TUBD1 | 1.272367 | 5.47E-06 | 6.10E-05 |
| EDEM2 | -0.83719 | 5.51E-06 | 6.14E-05 |
| C7orf26 | -0.84713 | 5.67E-06 | 6.31E-05 |
| CHRNA4 | 6.589601 | 5.68E-06 | 6.31E-05 |
| MICU1 | -1.1517 | 5.73E-06 | 6.36E-05 |
| JMJD7 | -1.61306 | 5.73E-06 | 6.36E-05 |
| SEH1L | -0.89633 | 5.75E-06 | 6.37E-05 |
| POLL | 1.019082 | 5.85E-06 | 6.47E-05 |
| SLC9A4 | 6.579105 | 5.90E-06 | 6.53E-05 |
| LRRC45 | -0.90838 | 5.92E-06 | 6.54E-05 |
| TNRC18 | 1.676193 | 6.03E-06 | 6.65E-05 |
| SULF1 | -1.62501 | 6.09E-06 | 6.70E-05 |
| NDNF | -0.75898 | 6.08E-06 | 6.70E-05 |
| ENSAPLG00000008403 | -1.57555 | 6.11E-06 | 6.72E-05 |
| HGSNAT | -0.8909 | 6.16E-06 | 6.77E-05 |
| FBXO41 | 4.894795 | 6.17E-06 | 6.78E-05 |
| CPLX4 | 6.597564 | 6.18E-06 | 6.78E-05 |
| ASPM | 3.033274 | 6.24E-06 | 6.84E-05 |
| FZD7 | 0.951057 | 6.26E-06 | 6.85E-05 |
| HELZ2 | 4.201798 | 6.26E-06 | 6.85E-05 |
| ENSAPLG00000003769 | -1.68233 | 6.33E-06 | 6.91E-05 |
| PGD | -0.88996 | 6.33E-06 | 6.91E-05 |
| RPA1 | -0.80414 | 6.40E-06 | 6.98E-05 |
| SIRT7 | 1.467996 | 6.41E-06 | 6.99E-05 |
| ENSAPLG00000012647 | 1.18822 | 6.51E-06 | 7.09E-05 |
| AKAP10 | 0.924646 | 6.58E-06 | 7.14E-05 |
| ZMPSTE24 | -0.76303 | 6.57E-06 | 7.14E-05 |
| DUSP1 | 1.128211 | 6.60E-06 | 7.16E-05 |
| ARMC6 | -0.743 | 6.64E-06 | 7.20E-05 |
| ENSAPLG00000030318 | 1.394812 | 6.65E-06 | 7.20E-05 |
| XKR6 | 1.365476 | 6.93E-06 | 7.49E-05 |
| CHST10 | 0.855498 | 6.98E-06 | 7.55E-05 |
| RTCB | -0.72016 | 7.02E-06 | 7.58E-05 |
| TALDO1 | -0.90371 | 7.04E-06 | 7.58E-05 |
| XRCC5 | -0.81691 | 7.04E-06 | 7.58E-05 |
| YARS1 | -0.7669 | 7.05E-06 | 7.59E-05 |
| ENSAPLG00000006258 | -0.87288 | 7.06E-06 | 7.59E-05 |
| TMEM138 | 1.163496 | 7.07E-06 | 7.60E-05 |
| OTOGL | 2.58034 | 7.14E-06 | 7.67E-05 |
| TMEM171 | -2.1344 | 7.23E-06 | 7.74E-05 |
| TNNT3 | 2.849326 | 7.23E-06 | 7.74E-05 |
| CCN5 | -2.50618 | 7.23E-06 | 7.74E-05 |
| ZNF469 | 1.306711 | 7.34E-06 | 7.85E-05 |
| ENSAPLG00000005283 | -1.07946 | 7.36E-06 | 7.86E-05 |
| ENSAPLG00000011759 | 3.708942 | 7.45E-06 | 7.95E-05 |
| ATXN1 | 1.399667 | 7.65E-06 | 8.16E-05 |
| EIF1 | 1.072811 | 7.67E-06 | 8.17E-05 |
| SLC38A9 | -1.16502 | 7.70E-06 | 8.19E-05 |
| RPL4 | -0.88503 | 7.86E-06 | 8.35E-05 |
| ENSAPLG00000021973 | 2.948388 | 7.92E-06 | 8.41E-05 |
| CMAS | -0.94198 | 8.01E-06 | 8.49E-05 |
| ORC3 | -0.99396 | 8.07E-06 | 8.55E-05 |
| PAIP1 | -0.85869 | 8.08E-06 | 8.56E-05 |
| CLK1 | 0.92732 | 8.15E-06 | 8.62E-05 |
| BYSL | -0.85897 | 8.18E-06 | 8.65E-05 |
| MTHFR | 0.784292 | 8.33E-06 | 8.79E-05 |
| LAMP1 | -0.78556 | 8.37E-06 | 8.83E-05 |
| RBBP7 | -0.88624 | 8.53E-06 | 8.98E-05 |
| ADD1 | -0.79267 | 8.54E-06 | 8.98E-05 |
| EEF1AKNMT | -0.9865 | 8.55E-06 | 8.99E-05 |
| PAK3 | 1.227012 | 8.61E-06 | 9.04E-05 |
| SLC13A1 | 6.581967 | 8.63E-06 | 9.06E-05 |
| PFKFB3 | 1.303768 | 8.66E-06 | 9.07E-05 |
| NR1H3 | 0.768538 | 8.66E-06 | 9.07E-05 |
| RECQL | -0.86762 | 8.68E-06 | 9.08E-05 |
| ATXN7L1 | 1.062263 | 8.79E-06 | 9.19E-05 |
| VASH1 | 1.045922 | 8.84E-06 | 9.23E-05 |
| TCF24 | 2.086152 | 8.84E-06 | 9.23E-05 |
| ABI1 | 0.796516 | 8.90E-06 | 9.28E-05 |
| ATF4 | 0.721636 | 8.95E-06 | 9.32E-05 |
| G3BP1 | -0.68791 | 8.98E-06 | 9.34E-05 |
| CORO1C | -0.83824 | 9.00E-06 | 9.35E-05 |
| SCUBE1 | -1.9828 | 9.02E-06 | 9.37E-05 |
| ENSAPLG00000007649 | -0.98452 | 9.24E-06 | 9.57E-05 |
| ENSAPLG00000009836 | 0.952874 | 9.23E-06 | 9.57E-05 |
| RPS24 | 0.876412 | 9.24E-06 | 9.57E-05 |
| TRIM66 | 2.946288 | 9.27E-06 | 9.59E-05 |
| ENSAPLG00000003990 | -1.40752 | 9.29E-06 | 9.60E-05 |
| MRPL39 | -0.90423 | 9.35E-06 | 9.65E-05 |
| TASOR | 0.875888 | 9.41E-06 | 9.71E-05 |
| ENSAPLG00000029441 | 1.187227 | 9.67E-06 | 9.96E-05 |
| LZTS3 | 1.778795 | 1.03E-05 | 0.000106 |
| ZFAND2A | 0.995378 | 1.05E-05 | 0.000108 |
| MRPS35 | -0.91837 | 1.05E-05 | 0.000108 |
| ADH5 | -0.78809 | 1.06E-05 | 0.000109 |
| NGF | -1.58192 | 1.06E-05 | 0.000109 |
| GREM1 | 5.388891 | 1.07E-05 | 0.000109 |
| WDR12 | -0.74919 | 1.07E-05 | 0.00011 |
| RFK | 0.930376 | 1.08E-05 | 0.000111 |
| ELK4 | 1.072804 | 1.09E-05 | 0.000111 |
| LIPG | -1.01457 | 1.09E-05 | 0.000111 |
| ENSAPLG00000007323 | 0.706082 | 1.09E-05 | 0.000112 |
| SH3BP4 | -1.07478 | 1.10E-05 | 0.000112 |
| DYNC1I2 | -0.79666 | 1.11E-05 | 0.000113 |
| NPPB | 1.104508 | 1.11E-05 | 0.000113 |
| LRRC59 | -0.78997 | 1.12E-05 | 0.000114 |
| ENSAPLG00000016403 | -0.81781 | 1.14E-05 | 0.000115 |
| LRRC38 | 1.743237 | 1.14E-05 | 0.000116 |
| ENSAPLG00000008997 | 2.301485 | 1.15E-05 | 0.000117 |
| ENSAPLG00000015422 | 2.835705 | 1.16E-05 | 0.000117 |
| RNH1 | -0.71031 | 1.16E-05 | 0.000118 |
| DENND2C | 0.912207 | 1.17E-05 | 0.000118 |
| TUBGCP2 | -0.90195 | 1.17E-05 | 0.000118 |
| ENSAPLG00000021975 | 0.933831 | 1.17E-05 | 0.000118 |
| SLC22A3 | -1.93654 | 1.19E-05 | 0.00012 |
| SLC2A10 | -1.01566 | 1.20E-05 | 0.00012 |
| GPC4 | -0.80787 | 1.21E-05 | 0.000121 |
| ENSAPLG00000004985 | -1.07481 | 1.21E-05 | 0.000122 |
| SLC29A4 | 2.015866 | 1.24E-05 | 0.000125 |
| NCSTN | -0.80925 | 1.24E-05 | 0.000125 |
| ALDH1L2 | -1.86192 | 1.25E-05 | 0.000126 |
| ACTA2 | -1.04069 | 1.26E-05 | 0.000126 |
| JARID2 | 1.017572 | 1.26E-05 | 0.000127 |
| GTPBP8 | -1.04706 | 1.27E-05 | 0.000127 |
| FGF4 | 6.52137 | 1.27E-05 | 0.000127 |
| IKBKB | -0.80346 | 1.27E-05 | 0.000127 |
| ACAD11 | -0.97678 | 1.28E-05 | 0.000128 |
| RNF19B | 1.184754 | 1.29E-05 | 0.000128 |
| CRHR2 | -0.81636 | 1.30E-05 | 0.000129 |
| SPAST | -0.69227 | 1.30E-05 | 0.00013 |
| ENSAPLG00000001044 | 1.966022 | 1.31E-05 | 0.00013 |
| TTC28 | 0.994711 | 1.31E-05 | 0.00013 |
| PIK3R5 | 1.436449 | 1.32E-05 | 0.000131 |
| ZFHX3 | 1.390728 | 1.32E-05 | 0.000131 |
| EPB41L2 | -1.14467 | 1.32E-05 | 0.000131 |
| EVC | -1.27628 | 1.33E-05 | 0.000132 |
| PTGES2 | -0.88326 | 1.35E-05 | 0.000134 |
| TPCN1 | 0.986727 | 1.37E-05 | 0.000135 |
| FBXL14 | 1.396502 | 1.37E-05 | 0.000135 |
| GCK | 1.114289 | 1.37E-05 | 0.000135 |
| ENSAPLG00000026953 | 4.293122 | 1.39E-05 | 0.000136 |
| SETD7 | 0.867311 | 1.40E-05 | 0.000137 |
| SDC1 | -0.72005 | 1.40E-05 | 0.000138 |
| ENSAPLG00000009313 | -0.82296 | 1.40E-05 | 0.000138 |
| ELFN1 | 0.882157 | 1.41E-05 | 0.000138 |
| ARID5B | 0.916635 | 1.41E-05 | 0.000138 |
| BRSK2 | 2.062967 | 1.41E-05 | 0.000138 |
| SETBP1 | 1.257559 | 1.43E-05 | 0.000139 |
| PKD2 | -1.12119 | 1.43E-05 | 0.00014 |
| NMT1 | -0.73744 | 1.45E-05 | 0.000141 |
| CCNB2 | -1.05567 | 1.48E-05 | 0.000144 |
| PPP2R3C | -0.83134 | 1.48E-05 | 0.000144 |
| ENSAPLG00000016555 | -0.7382 | 1.52E-05 | 0.000148 |
| ENSAPLG00000014266 | -0.84214 | 1.53E-05 | 0.000149 |
| ENSAPLG00000016418 | 6.470635 | 1.53E-05 | 0.000149 |
| ECM2 | -0.9592 | 1.54E-05 | 0.00015 |
| MMGT1 | 0.716323 | 1.55E-05 | 0.00015 |
| SDCBP2 | 4.736122 | 1.55E-05 | 0.00015 |
| CDK7 | -0.88996 | 1.56E-05 | 0.000151 |
| TNR | 5.2706 | 1.56E-05 | 0.000151 |
| ENSAPLG00000004897 | -1.44135 | 1.57E-05 | 0.000152 |
| ATF7IP | 0.868226 | 1.60E-05 | 0.000155 |
| TP53INP2 | 2.875474 | 1.60E-05 | 0.000155 |
| FNDC3A | 0.863428 | 1.61E-05 | 0.000155 |
| PSMA4 | -0.83152 | 1.61E-05 | 0.000156 |
| SEC23B | -0.70119 | 1.62E-05 | 0.000156 |
| ENSAPLG00000003696 | 6.467645 | 1.62E-05 | 0.000156 |
| UQCRC1 | -0.74799 | 1.63E-05 | 0.000157 |
| MTCH2 | -0.83947 | 1.64E-05 | 0.000157 |
| SAG | 6.195746 | 1.65E-05 | 0.000158 |
| NPY | -1.48082 | 1.66E-05 | 0.000159 |
| PARP3 | -1.50436 | 1.66E-05 | 0.000159 |
| COPB2 | -0.6889 | 1.68E-05 | 0.000161 |
| UTP18 | -0.77309 | 1.69E-05 | 0.000161 |
| CEP85L | 0.947977 | 1.68E-05 | 0.000161 |
| PURG | 1.856172 | 1.70E-05 | 0.000163 |
| USP5 | -0.86175 | 1.71E-05 | 0.000163 |
| FAM43A | 1.370385 | 1.71E-05 | 0.000163 |
| BCLAF3 | 0.768904 | 1.75E-05 | 0.000166 |
| PIK3IP1 | -0.81753 | 1.76E-05 | 0.000167 |
| HECW2 | 0.968263 | 1.76E-05 | 0.000167 |
| FUCA2 | -0.78982 | 1.77E-05 | 0.000168 |
| BUB3 | -0.78076 | 1.79E-05 | 0.00017 |
| LMAN1 | -0.77215 | 1.80E-05 | 0.000171 |
| C1GALT1 | 0.94407 | 1.83E-05 | 0.000173 |
| ZCCHC2 | 0.951401 | 1.87E-05 | 0.000177 |
| MAP3K13 | 1.07426 | 1.87E-05 | 0.000177 |
| SCFD1 | -0.76555 | 1.87E-05 | 0.000177 |
| SEC63 | -0.66831 | 1.89E-05 | 0.000179 |
| PPP2R2D | 0.678205 | 1.91E-05 | 0.00018 |
| CEMIP | 1.097054 | 1.92E-05 | 0.00018 |
| ARL14 | -2.10642 | 1.92E-05 | 0.000181 |
| ENSAPLG00000026807 | 1.125234 | 1.93E-05 | 0.000181 |
| UNG | -1.37655 | 1.93E-05 | 0.000182 |
| NOTUM | -1.01833 | 1.94E-05 | 0.000182 |
| ENSAPLG00000003866 | 1.669749 | 1.96E-05 | 0.000184 |
| CIT | 1.974614 | 1.96E-05 | 0.000184 |
| PROM2 | 4.2323 | 1.96E-05 | 0.000184 |
| DIPK2A | -1.23557 | 1.99E-05 | 0.000186 |
| PEX16 | -0.81492 | 2.00E-05 | 0.000186 |
| HS3ST3A1 | 1.807366 | 2.00E-05 | 0.000186 |
| FAM180B | 4.249611 | 2.03E-05 | 0.000189 |
| PITRM1 | -0.81534 | 2.04E-05 | 0.00019 |
| ENSAPLG00000030024 | -1.08551 | 2.06E-05 | 0.000192 |
| ENSAPLG00000006561 | 0.846006 | 2.07E-05 | 0.000192 |
| SLC5A1 | 2.578003 | 2.07E-05 | 0.000192 |
| ENSAPLG00000020029 | 1.110493 | 2.07E-05 | 0.000192 |
| SUMF1 | -0.86518 | 2.10E-05 | 0.000195 |
| HSDL1 | -0.84594 | 2.10E-05 | 0.000195 |
| AGAP1 | 0.91127 | 2.13E-05 | 0.000197 |
| PLK4 | 1.101782 | 2.13E-05 | 0.000197 |
| PLPP5 | -1.12675 | 2.13E-05 | 0.000197 |
| PANK3 | 0.78732 | 2.14E-05 | 0.000197 |
| ST3GAL3 | 0.891474 | 2.14E-05 | 0.000197 |
| ENSAPLG00000018551 | 0.811608 | 2.14E-05 | 0.000197 |
| ENSAPLG00000028904 | 1.191985 | 2.14E-05 | 0.000197 |
| ENSAPLG00000008352 | 1.756698 | 2.15E-05 | 0.000198 |
| CDC20 | -1.31463 | 2.17E-05 | 0.000199 |
| ABCE1 | -0.75481 | 2.19E-05 | 0.000201 |
| FAM114A1 | -0.79676 | 2.19E-05 | 0.000201 |
| KLHL40 | 0.913278 | 2.21E-05 | 0.000202 |
| SEMA3G | 1.096422 | 2.22E-05 | 0.000203 |
| PITX1 | 1.011481 | 2.22E-05 | 0.000203 |
| INPP5A | 0.818995 | 2.23E-05 | 0.000204 |
| MTHFD1 | -0.88747 | 2.24E-05 | 0.000204 |
| CAND1 | -0.87194 | 2.23E-05 | 0.000204 |
| ENSAPLG00000028020 | 0.970777 | 2.24E-05 | 0.000205 |
| PARN | -0.72575 | 2.28E-05 | 0.000208 |
| PRMT3 | -0.73318 | 2.29E-05 | 0.000208 |
| DUSP8 | 1.274387 | 2.31E-05 | 0.00021 |
| VSTM4 | -1.27876 | 2.31E-05 | 0.00021 |
| ENSAPLG00000008185 | -1.08571 | 2.33E-05 | 0.000212 |
| TUBGCP3 | -0.97226 | 2.33E-05 | 0.000212 |
| GLOD4 | -0.84157 | 2.39E-05 | 0.000217 |
| ATL1 | -0.71112 | 2.44E-05 | 0.000221 |
| ITPRID2 | 0.931117 | 2.44E-05 | 0.000221 |
| ZBTB18 | 0.934758 | 2.45E-05 | 0.000222 |
| RAB11FIP3 | 1.154311 | 2.48E-05 | 0.000224 |
| PTGES3 | 0.752059 | 2.48E-05 | 0.000224 |
| SH3PXD2A | 0.904303 | 2.51E-05 | 0.000226 |
| AKR1D1 | -1.34047 | 2.52E-05 | 0.000227 |
| MCM3 | -2.0165 | 2.51E-05 | 0.000227 |
| OAT | -0.71775 | 2.51E-05 | 0.000227 |
| FHOD1 | 1.003975 | 2.52E-05 | 0.000227 |
| XPNPEP1 | -0.73584 | 2.54E-05 | 0.000228 |
| RBPMS | 0.943934 | 2.56E-05 | 0.00023 |
| PLSCR1 | -0.93698 | 2.56E-05 | 0.00023 |
| RABL6 | 0.874783 | 2.60E-05 | 0.000233 |
| NSMCE1 | -1.00367 | 2.62E-05 | 0.000235 |
| TMEM41A | -1.0483 | 2.63E-05 | 0.000235 |
| CCT7 | -0.72811 | 2.64E-05 | 0.000237 |
| TLR6 | -1.21173 | 2.66E-05 | 0.000238 |
| PLA2G4A | -0.73789 | 2.66E-05 | 0.000238 |
| ORAI1 | -0.86332 | 2.67E-05 | 0.000238 |
| ALG5 | -0.77947 | 2.67E-05 | 0.000238 |
| HKDC1 | 4.573737 | 2.67E-05 | 0.000238 |
| ANGEL1 | 0.889539 | 2.70E-05 | 0.000241 |
| KCNC2 | 0.753165 | 2.72E-05 | 0.000242 |
| DDR2 | -0.68398 | 2.72E-05 | 0.000242 |
| VPS36 | -0.94636 | 2.73E-05 | 0.000242 |
| BACE1 | 0.747899 | 2.74E-05 | 0.000243 |
| MON1A | 1.00794 | 2.75E-05 | 0.000244 |
| FKBP10 | -0.72526 | 2.75E-05 | 0.000244 |
| JADE2 | 1.565753 | 2.76E-05 | 0.000245 |
| MB | 3.707927 | 2.77E-05 | 0.000245 |
| RASD2 | 5.197599 | 2.77E-05 | 0.000245 |
| ERCC4 | -0.92594 | 2.77E-05 | 0.000245 |
| LIG3 | -0.86168 | 2.78E-05 | 0.000246 |
| RTN4 | -0.73144 | 2.82E-05 | 0.000249 |
| SUCLA2 | -0.72302 | 2.83E-05 | 0.000249 |
| FASTKD1 | -1.67621 | 2.86E-05 | 0.000252 |
| ENSAPLG00000012949 | -1.41228 | 2.87E-05 | 0.000252 |
| ADAMTSL3 | -0.67323 | 2.88E-05 | 0.000253 |
| ERBB4 | 1.249799 | 2.89E-05 | 0.000254 |
| MEMO1 | 0.774508 | 2.91E-05 | 0.000255 |
| SETD5 | 0.959012 | 2.95E-05 | 0.000259 |
| ENSAPLG00000004084 | -0.84412 | 2.96E-05 | 0.000259 |
| MANEA | 0.732985 | 2.96E-05 | 0.000259 |
| TXNDC5 | -0.78447 | 2.96E-05 | 0.000259 |
| AKTIP | -0.72801 | 2.96E-05 | 0.000259 |
| HIPK1 | 1.081345 | 2.97E-05 | 0.000259 |
| ENSAPLG00000001060 | 3.735088 | 2.98E-05 | 0.00026 |
| WBP2NL | 0.784287 | 3.02E-05 | 0.000263 |
| TYW5 | -0.98952 | 3.03E-05 | 0.000264 |
| ENSAPLG00000016881 | 0.935459 | 3.05E-05 | 0.000266 |
| HACD1 | 0.829322 | 3.08E-05 | 0.000268 |
| SRPX | -1.09973 | 3.08E-05 | 0.000268 |
| TSHZ1 | 1.17978 | 3.14E-05 | 0.000273 |
| MED27 | -0.77625 | 3.14E-05 | 0.000273 |
| PNLDC1 | 3.706432 | 3.19E-05 | 0.000277 |
| NUP88 | -0.83444 | 3.23E-05 | 0.00028 |
| ZCCHC10 | 0.776331 | 3.24E-05 | 0.000281 |
| ATRN | -1.01782 | 3.25E-05 | 0.000281 |
| ENSAPLG00000008933 | -0.8207 | 3.25E-05 | 0.000281 |
| ENSAPLG00000023864 | 1.709042 | 3.28E-05 | 0.000283 |
| SSR2 | -0.74894 | 3.28E-05 | 0.000283 |
| SREK1 | -0.95414 | 3.30E-05 | 0.000284 |
| SETD3 | -0.74805 | 3.31E-05 | 0.000284 |
| KAT6A | 0.835009 | 3.31E-05 | 0.000284 |
| EXT2 | -0.89297 | 3.30E-05 | 0.000284 |
| KIAA0895 | 0.802932 | 3.30E-05 | 0.000284 |
| GUCY1B1 | -0.93417 | 3.36E-05 | 0.000289 |
| ENSAPLG00000011675 | 1.076716 | 3.37E-05 | 0.000289 |
| FAM76A | 0.868888 | 3.39E-05 | 0.000291 |
| CCNE1 | -0.84479 | 3.42E-05 | 0.000293 |
| POP4 | -1.02932 | 3.46E-05 | 0.000297 |
| ENSAPLG00000018678 | 1.013715 | 3.52E-05 | 0.000301 |
| IARS2 | -0.77377 | 3.53E-05 | 0.000302 |
| ENSAPLG00000002847 | -0.69116 | 3.54E-05 | 0.000302 |
| CKB | -0.86642 | 3.58E-05 | 0.000306 |
| OVOL2 | 1.949735 | 3.58E-05 | 0.000306 |
| TENT5A | 0.680862 | 3.59E-05 | 0.000306 |
| PTCHD1 | 0.918558 | 3.66E-05 | 0.000311 |
| PPT1 | -0.90764 | 3.67E-05 | 0.000313 |
| QDPR | -0.82109 | 3.68E-05 | 0.000313 |
| MYLIP | 0.748758 | 3.68E-05 | 0.000313 |
| ZNF644 | 0.65013 | 3.74E-05 | 0.000317 |
| ENSAPLG00000024538 | 1.049442 | 3.75E-05 | 0.000318 |
| SMPD1 | -0.75335 | 3.79E-05 | 0.000321 |
| ACSS3 | -1.54769 | 3.80E-05 | 0.000322 |
| SH3RF3 | 0.98598 | 3.82E-05 | 0.000323 |
| RPP30 | -0.76658 | 3.83E-05 | 0.000324 |
| MYO5A | 0.903017 | 3.85E-05 | 0.000325 |
| ENSAPLG00000000857 | -1.7198 | 3.85E-05 | 0.000325 |
| ARHGAP21 | 0.926433 | 3.87E-05 | 0.000326 |
| CALCB | -1.28092 | 3.89E-05 | 0.000328 |
| SLC39A6 | -0.74883 | 3.89E-05 | 0.000328 |
| CAMSAP2 | 0.878169 | 3.91E-05 | 0.000329 |
| MYOCD | 1.119789 | 3.91E-05 | 0.000329 |
| ENSAPLG00000016352 | -0.80456 | 3.92E-05 | 0.000329 |
| MMD | 0.807483 | 3.95E-05 | 0.000332 |
| TBL1XR1 | 0.650969 | 3.99E-05 | 0.000335 |
| MAP7 | 0.738061 | 4.00E-05 | 0.000336 |
| PRKAG1 | 1.551573 | 4.02E-05 | 0.000337 |
| DNAJC17 | -0.92443 | 4.04E-05 | 0.000338 |
| HK1 | -0.71955 | 4.04E-05 | 0.000338 |
| CLDN20 | 6.228054 | 4.05E-05 | 0.000338 |
| RNF11 | 0.704347 | 4.07E-05 | 0.00034 |
| RPN1 | -0.66844 | 4.11E-05 | 0.000343 |
| NOX4 | 0.763884 | 4.17E-05 | 0.000348 |
| BCAT1 | -0.78405 | 4.18E-05 | 0.000348 |
| NCOA7 | 0.813852 | 4.24E-05 | 0.000353 |
| ENSAPLG00000018956 | 4.641761 | 4.28E-05 | 0.000356 |
| ENSAPLG00000009553 | 1.233603 | 4.30E-05 | 0.000358 |
| GPR27 | 0.837495 | 4.31E-05 | 0.000358 |
| PLEKHB2 | 0.799132 | 4.32E-05 | 0.000359 |
| RPL22L1 | 1.025903 | 4.34E-05 | 0.000359 |
| ENSAPLG00000021234 | -1.57031 | 4.33E-05 | 0.000359 |
| HMGB2 | -1.21795 | 4.36E-05 | 0.000361 |
| RGP1 | -1.17062 | 4.36E-05 | 0.000361 |
| TCF12 | 0.681159 | 4.38E-05 | 0.000362 |
| DCN | -0.84925 | 4.41E-05 | 0.000364 |
| NKX2-5 | 6.203565 | 4.44E-05 | 0.000367 |
| ALG8 | -0.85638 | 4.50E-05 | 0.000371 |
| EEF2 | -0.64334 | 4.52E-05 | 0.000373 |
| ABCA4 | 2.497476 | 4.53E-05 | 0.000373 |
| ENSAPLG00000020680 | 0.73261 | 4.59E-05 | 0.000378 |
| LRTM2 | 5.788753 | 4.66E-05 | 0.000383 |
| ENSAPLG00000020417 | -0.83399 | 4.66E-05 | 0.000383 |
| ENSAPLG00000010727 | -0.97028 | 4.70E-05 | 0.000386 |
| ATIC | -0.71359 | 4.74E-05 | 0.000389 |
| GCNT4 | 1.148779 | 4.76E-05 | 0.00039 |
| NUP133 | -0.91122 | 4.77E-05 | 0.000391 |
| MVP | -0.87656 | 4.83E-05 | 0.000396 |
| AGO4 | 0.92842 | 4.90E-05 | 0.000401 |
| ZNF236 | 0.79996 | 5.05E-05 | 0.000413 |
| PRKAA1 | -0.8709 | 5.08E-05 | 0.000415 |
| ENSAPLG00000016876 | 4.490849 | 5.11E-05 | 0.000417 |
| BAK1 | 0.744213 | 5.12E-05 | 0.000417 |
| BLOC1S6 | 0.724247 | 5.14E-05 | 0.000419 |
| CLPX | -0.75143 | 5.23E-05 | 0.000426 |
| TSPAN13 | 2.894119 | 5.26E-05 | 0.000429 |
| CPSF7 | 1.473949 | 5.28E-05 | 0.00043 |
| ENSAPLG00000011141 | 1.277955 | 5.33E-05 | 0.000433 |
| LDLRAP1 | -0.81309 | 5.34E-05 | 0.000434 |
| ENSAPLG00000029258 | 2.21107 | 5.35E-05 | 0.000435 |
| FHL2 | -1.05813 | 5.38E-05 | 0.000436 |
| QTRT2 | -0.75097 | 5.41E-05 | 0.000438 |
| CBFA2T3 | 1.907867 | 5.41E-05 | 0.000438 |
| CUBN | 0.900593 | 5.43E-05 | 0.000439 |
| ENSAPLG00000016202 | -0.88863 | 5.43E-05 | 0.000439 |
| EMILIN2 | -0.60646 | 5.48E-05 | 0.000443 |
| NUDT9 | -1.01798 | 5.49E-05 | 0.000443 |
| ENSAPLG00000001882 | -0.91028 | 5.49E-05 | 0.000443 |
| LATS2 | 0.928917 | 5.57E-05 | 0.000449 |
| ACADS | -0.90548 | 5.58E-05 | 0.00045 |
| RFLNA | 6.188028 | 5.60E-05 | 0.000451 |
| SLC5A7 | 0.964501 | 5.62E-05 | 0.000452 |
| EIF2B1 | -0.77259 | 5.66E-05 | 0.000455 |
| BZW1 | -0.68175 | 5.72E-05 | 0.000459 |
| PSMD5 | -0.68717 | 5.73E-05 | 0.00046 |
| CD47 | -1.00696 | 5.74E-05 | 0.00046 |
| ENSAPLG00000008466 | -0.80504 | 5.74E-05 | 0.00046 |
| RMND5A | -0.69123 | 5.79E-05 | 0.000463 |
| NR0B2 | 2.010454 | 5.83E-05 | 0.000466 |
| UBE2O | 1.255954 | 5.86E-05 | 0.000468 |
| IMPA2 | 1.01323 | 5.86E-05 | 0.000468 |
| YOD1 | 0.946836 | 5.92E-05 | 0.000473 |
| LEKR1 | 2.17647 | 5.96E-05 | 0.000476 |
| ENSAPLG00000023154 | 1.034718 | 5.97E-05 | 0.000476 |
| SAT1 | 0.83529 | 5.98E-05 | 0.000476 |
| EFCAB7 | -1.7187 | 5.99E-05 | 0.000477 |
| SLC10A4 | -0.81807 | 5.99E-05 | 0.000477 |
| ENSAPLG00000020378 | 0.686342 | 6.01E-05 | 0.000478 |
| ENSAPLG00000003903 | -0.68156 | 6.03E-05 | 0.000479 |
| ANO10 | -0.9326 | 6.06E-05 | 0.000481 |
| NELL1 | -0.88721 | 6.06E-05 | 0.000481 |
| ENSAPLG00000005363 | -0.7374 | 6.09E-05 | 0.000483 |
| ENSAPLG00000013069 | -0.69587 | 6.17E-05 | 0.000489 |
| PHF20 | 0.871159 | 6.24E-05 | 0.000493 |
| BAG2 | 0.883913 | 6.23E-05 | 0.000493 |
| APOOL | -0.96832 | 6.23E-05 | 0.000493 |
| ATP1B3 | -0.65667 | 6.27E-05 | 0.000495 |
| IKZF5 | 0.993028 | 6.28E-05 | 0.000496 |
| DCUN1D3 | 0.775802 | 6.33E-05 | 0.0005 |
| LPAR3 | 5.020967 | 6.40E-05 | 0.000504 |
| URM1 | 0.949999 | 6.41E-05 | 0.000505 |
| ACO2 | -0.67737 | 6.44E-05 | 0.000507 |
| NUP35 | -0.81849 | 6.48E-05 | 0.00051 |
| COG7 | -0.74287 | 6.49E-05 | 0.00051 |
| ENSAPLG00000010185 | -0.80563 | 6.50E-05 | 0.000511 |
| NABP1 | -0.66921 | 6.56E-05 | 0.000515 |
| RXFP1 | 6.118434 | 6.57E-05 | 0.000515 |
| LAMP2 | -0.64966 | 6.58E-05 | 0.000515 |
| ENSAPLG00000017902 | 3.190421 | 6.58E-05 | 0.000515 |
| ENSAPLG00000014346 | -1.02274 | 6.60E-05 | 0.000517 |
| MYO3B | 6.208048 | 6.73E-05 | 0.000527 |
| THAP4 | 0.682481 | 6.79E-05 | 0.00053 |
| CALD1 | -0.88093 | 6.86E-05 | 0.000536 |
| PACS2 | 0.730057 | 6.90E-05 | 0.000538 |
| SLC7A11 | -0.87572 | 6.91E-05 | 0.000539 |
| STK39 | -0.99472 | 6.98E-05 | 0.000544 |
| TPM4 | -1.07867 | 6.99E-05 | 0.000544 |
| TLK2 | 0.85006 | 7.00E-05 | 0.000545 |
| ALAS1 | -0.73766 | 7.04E-05 | 0.000547 |
| INTS11 | -0.64557 | 7.06E-05 | 0.000549 |
| PTCH1 | 1.752605 | 7.11E-05 | 0.000552 |
| ANXA1 | -0.88554 | 7.14E-05 | 0.000554 |
| INTS13 | 0.689881 | 7.26E-05 | 0.000563 |
| SLC16A5 | 6.1034 | 7.27E-05 | 0.000563 |
| MRM1 | -1.08609 | 7.28E-05 | 0.000564 |
| STX18 | -1.47061 | 7.37E-05 | 0.00057 |
| ATP5MF | 0.889665 | 7.39E-05 | 0.000571 |
| PRRG3 | 2.040169 | 7.40E-05 | 0.000571 |
| FABP6 | 1.691897 | 7.42E-05 | 0.000573 |
| TM9SF2 | -0.64128 | 7.45E-05 | 0.000575 |
| ACTA1 | -0.96069 | 7.47E-05 | 0.000576 |
| ACTR6 | 0.669411 | 7.47E-05 | 0.000576 |
| ITFG1 | -0.64003 | 7.49E-05 | 0.000577 |
| RAPGEF2 | 0.757464 | 7.50E-05 | 0.000577 |
| STK11 | 1.123286 | 7.54E-05 | 0.00058 |
| TXNL4B | -1.11107 | 7.59E-05 | 0.000583 |
| CWF19L1 | -0.68617 | 7.62E-05 | 0.000585 |
| ENSAPLG00000004100 | 1.432924 | 7.67E-05 | 0.000587 |
| CCT8 | -0.68145 | 7.66E-05 | 0.000587 |
| ZC3H18 | 0.946356 | 7.66E-05 | 0.000587 |
| ENSAPLG00000028144 | -0.79269 | 7.72E-05 | 0.000591 |
| PXDN | -1.19121 | 7.75E-05 | 0.000593 |
| SMG1 | 0.924881 | 7.76E-05 | 0.000593 |
| SLC49A4 | -0.72268 | 7.79E-05 | 0.000595 |
| WLS | -0.66318 | 7.80E-05 | 0.000595 |
| NCKIPSD | 0.983503 | 7.83E-05 | 0.000597 |
| SOX10 | 6.117251 | 7.83E-05 | 0.000597 |
| ENSAPLG00000010396 | -0.95534 | 7.87E-05 | 0.0006 |
| ENSAPLG00000024194 | -1.48851 | 7.88E-05 | 0.0006 |
| ENSAPLG00000004542 | -1.03067 | 7.89E-05 | 0.0006 |
| ACTR3 | -0.65113 | 7.94E-05 | 0.000604 |
| COQ9 | -0.82196 | 7.97E-05 | 0.000605 |
| GLIS2 | 1.478616 | 8.02E-05 | 0.000608 |
| ST7L | -0.7361 | 8.03E-05 | 0.000609 |
| ENSAPLG00000021525 | -1.15938 | 8.06E-05 | 0.000611 |
| MOB2 | 0.725067 | 8.08E-05 | 0.000612 |
| VCP | -0.60964 | 8.13E-05 | 0.000615 |
| ENSAPLG00000003190 | 1.068437 | 8.18E-05 | 0.000619 |
| CSDC2 | 2.477717 | 8.34E-05 | 0.00063 |
| SIAH1 | -0.73392 | 8.40E-05 | 0.000635 |
| ENSAPLG00000011920 | 1.061739 | 8.43E-05 | 0.000636 |
| LRRC47 | -0.90699 | 8.45E-05 | 0.000637 |
| ENSAPLG00000027821 | 1.147596 | 8.46E-05 | 0.000638 |
| FRY | 1.56979 | 8.50E-05 | 0.00064 |
| LRFN5 | 1.262183 | 8.50E-05 | 0.00064 |
| MARCHF4 | 2.045574 | 8.54E-05 | 0.000642 |
| TRIM24 | 0.756422 | 8.55E-05 | 0.000643 |
| ADCK1 | -0.92941 | 8.57E-05 | 0.000644 |
| FAAH | -1.87026 | 8.60E-05 | 0.000646 |
| HADHB | -0.6972 | 8.64E-05 | 0.000648 |
| ENSAPLG00000009803 | -1.17432 | 8.68E-05 | 0.000651 |
| GRAMD1C | -0.83429 | 8.70E-05 | 0.000652 |
| CCNH | -0.92909 | 8.74E-05 | 0.000654 |
| ENSAPLG00000007453 | 3.00298 | 8.74E-05 | 0.000654 |
| RANGAP1 | -0.70022 | 8.82E-05 | 0.000659 |
| KIFBP | -0.79376 | 8.90E-05 | 0.000665 |
| ENSAPLG00000008356 | 1.053726 | 8.92E-05 | 0.000666 |
| SLC19A1 | -1.118 | 8.94E-05 | 0.000667 |
| RFLNB | 1.220291 | 9.05E-05 | 0.000675 |
| PCDH8 | 1.064756 | 9.05E-05 | 0.000675 |
| SGCE | -0.90375 | 9.09E-05 | 0.000677 |
| EXPH5 | 1.412939 | 9.10E-05 | 0.000677 |
| HMGXB4 | 0.859441 | 9.21E-05 | 0.000685 |
| HACD2 | -0.82053 | 9.25E-05 | 0.000687 |
| FGF12 | 1.601212 | 9.28E-05 | 0.000689 |
| ASB7 | 0.713273 | 9.42E-05 | 0.000699 |
| ENSAPLG00000020873 | 2.381192 | 9.43E-05 | 0.000699 |
| CYP20A1 | -0.67824 | 9.55E-05 | 0.000707 |
| SLC24A1 | 6.234836 | 9.59E-05 | 0.00071 |
| NUP37 | -0.70784 | 9.66E-05 | 0.000715 |
| EEPD1 | -0.77328 | 9.82E-05 | 0.000727 |
| FAM219A | 1.036643 | 9.87E-05 | 0.000729 |
| ENSAPLG00000008568 | -0.74572 | 0.000101 | 0.000743 |
| PCNA | -0.75076 | 0.000101 | 0.000748 |
| COPS4 | -0.67023 | 0.000102 | 0.000753 |
| KARS1 | -0.66651 | 0.000102 | 0.000753 |
| USP6NL | 0.753335 | 0.000103 | 0.00076 |
| ENSAPLG00000001119 | 2.86115 | 0.000104 | 0.000762 |
| OSGIN2 | -1.18608 | 0.000104 | 0.000765 |
| LACTB2 | -0.73223 | 0.000104 | 0.000765 |
| GOLPH3 | 0.67358 | 0.000104 | 0.000765 |
| ENSAPLG00000002946 | -0.76029 | 0.000105 | 0.000768 |
| PLBD2 | -0.79584 | 0.000105 | 0.000769 |
| TARDBP | -0.65092 | 0.000105 | 0.000771 |
| ENSAPLG00000009118 | 0.986755 | 0.000105 | 0.000771 |
| STYK1 | 3.088683 | 0.000106 | 0.000774 |
| TES | -0.61022 | 0.000107 | 0.000782 |
| PCCB | -0.93518 | 0.000107 | 0.000785 |
| ENSAPLG00000010383 | -0.98107 | 0.000108 | 0.000787 |
| SPTLC2 | -0.71866 | 0.000108 | 0.000787 |
| NGLY1 | -0.93294 | 0.000108 | 0.000788 |
| DHCR7 | -0.69335 | 0.000108 | 0.000791 |
| ENSAPLG00000002824 | 4.437089 | 0.000109 | 0.000792 |
| VTA1 | -0.72706 | 0.000109 | 0.000796 |
| ENSAPLG00000004058 | -0.81959 | 0.000109 | 0.000796 |
| ASPHD2 | 1.894829 | 0.000109 | 0.000796 |
| STYX | 0.768899 | 0.00011 | 0.000797 |
| TBC1D8B | -1.08437 | 0.00011 | 0.000799 |
| TIPIN | -0.9545 | 0.00011 | 0.0008 |
| GPR153 | 1.481743 | 0.00011 | 0.0008 |
| DYNLRB1 | 0.79256 | 0.00011 | 0.0008 |
| ENSAPLG00000002035 | 1.193933 | 0.000113 | 0.000818 |
| ENSAPLG00000019624 | 1.79198 | 0.000114 | 0.000826 |
| SMAP1 | -0.84082 | 0.000115 | 0.000832 |
| ENSAPLG00000007040 | 0.846316 | 0.000116 | 0.000837 |
| GLIS3 | -1.31841 | 0.000116 | 0.000841 |
| ZMIZ1 | 1.327911 | 0.000116 | 0.000841 |
| COL1A2 | -0.88444 | 0.000118 | 0.000852 |
| ENSAPLG00000017178 | 1.16856 | 0.00012 | 0.000863 |
| NAGA | -1.12783 | 0.00012 | 0.000865 |
| NR1D2 | 0.626113 | 0.00012 | 0.000865 |
| FAM13B | 0.815548 | 0.000121 | 0.00087 |
| ENSAPLG00000030799 | -1.05692 | 0.000121 | 0.000872 |
| ENSAPLG00000008335 | 0.657899 | 0.000122 | 0.000879 |
| BTBD10 | -0.64174 | 0.000123 | 0.000881 |
| MDH2 | -0.74667 | 0.000123 | 0.000886 |
| ENSAPLG00000021858 | 0.927705 | 0.000124 | 0.000893 |
| PKP4 | 0.695781 | 0.000126 | 0.000901 |
| CTNNB1 | -0.66999 | 0.000126 | 0.000901 |
| MSL2 | 1.245399 | 0.000126 | 0.000902 |
| MSH6 | -1.10523 | 0.000126 | 0.000903 |
| PNRC2 | 0.703957 | 0.000127 | 0.000907 |
| KIFAP3 | -0.66181 | 0.000127 | 0.000908 |
| SNX17 | 0.62892 | 0.000128 | 0.000911 |
| VEZF1 | 0.987571 | 0.000128 | 0.000914 |
| ENSAPLG00000023116 | 1.441064 | 0.000128 | 0.000914 |
| ENSAPLG00000011673 | 0.758339 | 0.000129 | 0.000919 |
| SAMD11 | 1.15535 | 0.000129 | 0.000921 |
| BEND5 | -0.82749 | 0.00013 | 0.000921 |
| SSX2IP | 1.834053 | 0.00013 | 0.000926 |
| KIF5B | -0.61425 | 0.00013 | 0.000926 |
| RECK | -0.78605 | 0.000132 | 0.000934 |
| NCDN | -0.92409 | 0.000132 | 0.000934 |
| LGALS3 | 0.937939 | 0.000132 | 0.000934 |
| SCLY | -1.27729 | 0.000133 | 0.00094 |
| ENSAPLG00000025225 | 0.714662 | 0.000133 | 0.00094 |
| CPE | -1.01444 | 0.000134 | 0.000948 |
| ENSAPLG00000008170 | -1.12215 | 0.000134 | 0.000948 |
| PER3 | 1.082265 | 0.000134 | 0.00095 |
| ERO1A | -0.87364 | 0.000135 | 0.000951 |
| MRAS | 0.993642 | 0.000135 | 0.000951 |
| OPHN1 | 1.297682 | 0.000135 | 0.000952 |
| NISCH | 0.824964 | 0.000136 | 0.000956 |
| CCT4 | -0.67386 | 0.000137 | 0.000967 |
| MBD5 | 0.766569 | 0.000138 | 0.000971 |
| TNFAIP3 | 0.94833 | 0.000139 | 0.000977 |
| TRIB1 | 0.756083 | 0.00014 | 0.000987 |
| ENSAPLG00000006355 | 0.892095 | 0.00014 | 0.000987 |
| ADTRP | 1.643242 | 0.000141 | 0.000991 |
| CREBRF | 0.714496 | 0.000141 | 0.000991 |
| CLTA | -0.92523 | 0.000141 | 0.000991 |
| PYGL | -0.7386 | 0.000141 | 0.000991 |
| KLF6 | 0.66559 | 0.000142 | 0.000996 |
| ENSAPLG00000004341 | 0.856309 | 0.000143 | 0.000997 |
| TAF1B | -1.10248 | 0.000142 | 0.000997 |
| IRAK2 | 0.654787 | 0.000143 | 0.000998 |
| ENSAPLG00000008465 | 4.326798 | 0.000143 | 0.000998 |
| APPBP2 | -0.66878 | 0.000144 | 0.001009 |
| ENSAPLG00000016924 | 1.639997 | 0.000146 | 0.001017 |
| ENSAPLG00000024554 | 1.248147 | 0.000147 | 0.001024 |
| TOB1 | 0.73257 | 0.000147 | 0.001025 |
| ZDHHC6 | 0.631904 | 0.000147 | 0.001025 |
| MTRF1L | -1.02496 | 0.000148 | 0.001027 |
| GTF2IRD1 | 0.711004 | 0.000148 | 0.001029 |
| ENSAPLG00000015887 | -0.84827 | 0.000148 | 0.001029 |
| PTP4A3 | 0.836452 | 0.000148 | 0.001029 |
| ENSAPLG00000026915 | -0.75608 | 0.000149 | 0.001035 |
| FYN | 0.588132 | 0.00015 | 0.001039 |
| TEX11 | 1.26952 | 0.00015 | 0.001041 |
| ENSAPLG00000018666 | 2.570651 | 0.00015 | 0.001041 |
| RAC2 | 1.148775 | 0.000151 | 0.001045 |
| DHDDS | -0.68559 | 0.000152 | 0.001052 |
| PLPP6 | -1.08994 | 0.000153 | 0.001059 |
| ZBTB20 | 0.95135 | 0.000154 | 0.001063 |
| LLGL1 | 0.732157 | 0.000154 | 0.001063 |
| NLRX1 | 0.867801 | 0.000155 | 0.001069 |
| NRARP | 1.289429 | 0.000155 | 0.00107 |
| AXIN1 | 0.784994 | 0.000158 | 0.001091 |
| SLC7A1 | -1.14423 | 0.000159 | 0.001093 |
| POGLUT2 | -0.73147 | 0.00016 | 0.001102 |
| RORB | 0.72721 | 0.00016 | 0.001102 |
| ENSAPLG00000002445 | -0.84624 | 0.00016 | 0.001102 |
| AJM1 | 2.439989 | 0.00016 | 0.001102 |
| RPL36AL | 0.790624 | 0.000161 | 0.001104 |
| EXOC6 | -0.76708 | 0.000161 | 0.001106 |
| BTD | -1.19093 | 0.000162 | 0.001109 |
| RAP2C | 0.792354 | 0.000162 | 0.001109 |
| SNAP29 | -0.77869 | 0.000162 | 0.001109 |
| VPS45 | -0.93372 | 0.000162 | 0.001109 |
| ID2 | 0.838346 | 0.000163 | 0.001112 |
| ENSAPLG00000028568 | 1.123328 | 0.000164 | 0.001124 |
| SMU1 | -0.62941 | 0.000165 | 0.001126 |
| SEMA3C | -0.66806 | 0.000165 | 0.001127 |
| DHTKD1 | -0.88648 | 0.000165 | 0.001127 |
| ALDH7A1 | -0.67944 | 0.000165 | 0.001127 |
| EIF5 | 0.69004 | 0.000165 | 0.001127 |
| GRK7 | 2.170874 | 0.000166 | 0.001127 |
| TSR1 | -0.70391 | 0.000166 | 0.001127 |
| LHFPL2 | 0.625059 | 0.000166 | 0.001127 |
| ENSAPLG00000004266 | -0.95896 | 0.000171 | 0.00116 |
| CCDC92 | 0.947768 | 0.000171 | 0.00116 |
| ENSAPLG00000023707 | 1.468292 | 0.000171 | 0.001164 |
| ATF1 | 0.68655 | 0.000172 | 0.001168 |
| ARF6 | 0.748995 | 0.000174 | 0.001183 |
| CUL2 | -0.58704 | 0.000175 | 0.00119 |
| BACH1 | 0.793736 | 0.000177 | 0.001197 |
| MTPN | 0.706923 | 0.000177 | 0.0012 |
| FAR1 | -0.75619 | 0.000178 | 0.001205 |
| GCDH | -0.8329 | 0.000178 | 0.001206 |
| MBNL3 | 0.759883 | 0.000178 | 0.001206 |
| MOSMO | 1.001786 | 0.000181 | 0.001219 |
| ANXA7 | -0.80197 | 0.000181 | 0.001219 |
| ENSAPLG00000004823 | 0.695067 | 0.000181 | 0.001219 |
| ENSAPLG00000008315 | 1.194506 | 0.000181 | 0.001219 |
| ACOX3 | -0.81696 | 0.000181 | 0.001219 |
| ENSAPLG00000014933 | 1.202922 | 0.000181 | 0.001219 |
| SELENOM | 0.789069 | 0.000181 | 0.001219 |
| WDR1 | -0.56931 | 0.000181 | 0.001219 |
| HMG20B | -0.69009 | 0.000182 | 0.001222 |
| FGF8 | 4.913751 | 0.000182 | 0.001222 |
| ANAPC13 | 1.083206 | 0.000182 | 0.001224 |
| NAA35 | -0.66309 | 0.000182 | 0.001224 |
| STARD13 | 0.870338 | 0.000183 | 0.001224 |
| RIPOR1 | 0.897378 | 0.000183 | 0.001226 |
| HPD | 1.102502 | 0.000184 | 0.001229 |
| ZNF704 | 0.776757 | 0.000184 | 0.00123 |
| TMEM39A | 0.649358 | 0.000187 | 0.001249 |
| ENSAPLG00000014620 | -1.0979 | 0.000187 | 0.001249 |
| TADA2A | -0.91163 | 0.000189 | 0.001261 |
| PNPLA8 | -0.73765 | 0.000189 | 0.001261 |
| LRRC40 | -0.87231 | 0.00019 | 0.001269 |
| ACSL1 | -0.71941 | 0.00019 | 0.001269 |
| ENSAPLG00000003176 | 0.886482 | 0.000191 | 0.001273 |
| MACROH2A1 | -0.72353 | 0.000191 | 0.001274 |
| ENSAPLG00000029768 | 1.177387 | 0.000191 | 0.001274 |
| GTF3C4 | -1.03363 | 0.000192 | 0.001275 |
| ADSL | -0.72573 | 0.000192 | 0.001275 |
| LPAR2 | 0.791947 | 0.000192 | 0.001276 |
| ALDH1A3 | -1.35903 | 0.000193 | 0.001284 |
| SNAP47 | 0.797741 | 0.000194 | 0.001285 |
| KALRN | -0.86826 | 0.000194 | 0.001286 |
| HMGCR | -0.64391 | 0.000194 | 0.001287 |
| ENSAPLG00000024805 | 0.926424 | 0.000196 | 0.001297 |
| HINT3 | 0.735654 | 0.000196 | 0.001297 |
| SCO1 | -0.6512 | 0.000196 | 0.001297 |
| ASAH1 | -0.67126 | 0.000197 | 0.0013 |
| SYNPO | 1.22558 | 0.000199 | 0.001312 |
| ENSAPLG00000021254 | 1.117394 | 0.000199 | 0.001316 |
| CLBA1 | 0.874423 | 0.0002 | 0.001319 |
| TMEM214 | -0.6894 | 0.0002 | 0.001319 |
| SLC2A9 | 4.880371 | 0.000201 | 0.001325 |
| RECQL5 | -0.99378 | 0.000202 | 0.001331 |
| ENSAPLG00000004586 | -0.95765 | 0.000202 | 0.001331 |
| ATP2B1 | -0.824 | 0.000203 | 0.001332 |
| ENSAPLG00000013405 | -0.65948 | 0.000204 | 0.00134 |
| HEYL | 1.953255 | 0.000205 | 0.001346 |
| GPT2 | -1.09762 | 0.00021 | 0.001376 |
| ARHGAP29 | -0.73734 | 0.00021 | 0.00138 |
| PPP1R3C | -0.85748 | 0.000211 | 0.001381 |
| EPRS1 | -0.7021 | 0.000211 | 0.001382 |
| HSPA9 | -0.59888 | 0.000211 | 0.001382 |
| IQUB | 2.5576 | 0.000211 | 0.001382 |
| TFPI2 | -0.8199 | 0.000211 | 0.001382 |
| ESRRG | 0.769441 | 0.000212 | 0.001389 |
| SEC14L1 | 0.649979 | 0.000213 | 0.001395 |
| ENSAPLG00000007797 | -1.05434 | 0.000214 | 0.001395 |
| TK1 | -1.22218 | 0.000215 | 0.001402 |
| TMEM177 | -1.2121 | 0.000215 | 0.001402 |
| DNAJB5 | 1.577215 | 0.000215 | 0.001403 |
| SRSF4 | 0.961916 | 0.000215 | 0.001403 |
| ENSAPLG00000030894 | 1.035306 | 0.000216 | 0.001409 |
| ENSAPLG00000016592 | 0.965793 | 0.000217 | 0.001415 |
| HIVEP2 | 1.00271 | 0.000218 | 0.001418 |
| NIPA2 | 0.611076 | 0.000218 | 0.001418 |
| RPL13 | 0.775831 | 0.000219 | 0.001422 |
| SENP6 | 0.664314 | 0.00022 | 0.001429 |
| ENSAPLG00000008423 | 1.061557 | 0.00022 | 0.001429 |
| CHAMP1 | 1.063741 | 0.000221 | 0.00143 |
| ENSAPLG00000019499 | 0.756851 | 0.000222 | 0.001436 |
| DOK7 | 0.740397 | 0.000223 | 0.001442 |
| TMEM245 | -1.00246 | 0.000223 | 0.001446 |
| ENSAPLG00000000577 | -0.89037 | 0.000224 | 0.001446 |
| DRAM2 | 0.753409 | 0.000225 | 0.001455 |
| ASAP1 | 0.740119 | 0.000225 | 0.001455 |
| TAF5L | 0.745472 | 0.000227 | 0.001466 |
| SLC4A3 | 0.928473 | 0.000228 | 0.001467 |
| SPATA5L1 | -1.04113 | 0.000227 | 0.001467 |
| SIX2 | 1.188198 | 0.000228 | 0.001467 |
| AP3M2 | -0.66045 | 0.000228 | 0.001471 |
| HNF1B | 3.396397 | 0.000229 | 0.001473 |
| NSD2 | 0.653941 | 0.000229 | 0.001476 |
| DPH5 | -0.91202 | 0.00023 | 0.001476 |
| MARK1 | 0.596256 | 0.000231 | 0.001482 |
| ENSAPLG00000028972 | 1.526787 | 0.000232 | 0.001489 |
| CDK5RAP3 | -1.32275 | 0.000233 | 0.001498 |
| RBMX2 | 0.786202 | 0.000234 | 0.0015 |
| P3H2 | -1.39839 | 0.000235 | 0.001507 |
| MRPL37 | -1.01673 | 0.000235 | 0.001509 |
| SUCLG1 | -0.6772 | 0.000236 | 0.00151 |
| GPC1 | -0.70879 | 0.000238 | 0.00152 |
| SLC25A6 | -0.66192 | 0.000238 | 0.001521 |
| SELENOP | 0.663537 | 0.000238 | 0.001522 |
| CMC4 | 2.441849 | 0.00024 | 0.001532 |
| CRYBB2 | 2.121198 | 0.000241 | 0.001538 |
| CACNA1I | 5.996643 | 0.000241 | 0.001538 |
| JOSD1 | 1.034807 | 0.000242 | 0.001545 |
| ERMARD | -1.10796 | 0.000243 | 0.001546 |
| VTI1B | -0.80869 | 0.000243 | 0.001548 |
| OBI1 | -0.81195 | 0.000244 | 0.001552 |
| ENSAPLG00000003199 | 0.798033 | 0.000245 | 0.001561 |
| ENSAPLG00000009757 | 0.785393 | 0.000247 | 0.001568 |
| BNC2 | 0.985369 | 0.000248 | 0.001579 |
| DNM1L | -0.60871 | 0.00025 | 0.00159 |
| DHCR24 | -1.25857 | 0.000251 | 0.001591 |
| TPT1 | 0.731413 | 0.000253 | 0.001605 |
| ENSAPLG00000018470 | 5.915732 | 0.000254 | 0.001608 |
| TUB | 0.612867 | 0.000254 | 0.00161 |
| SP3 | 0.618502 | 0.000255 | 0.001616 |
| ERLEC1 | -0.66254 | 0.000257 | 0.001628 |
| RNF166 | 1.490832 | 0.000257 | 0.001628 |
| ENSAPLG00000003368 | -1.07913 | 0.000258 | 0.001629 |
| ENSAPLG00000027896 | 1.015229 | 0.000258 | 0.001629 |
| ZBTB1 | 0.576269 | 0.000258 | 0.00163 |
| ENSAPLG00000013205 | 0.80581 | 0.000259 | 0.001633 |
| NECTIN3 | -0.69037 | 0.00026 | 0.001639 |
| BPGM | 0.748335 | 0.000261 | 0.001645 |
| ADK | -0.63875 | 0.000262 | 0.001652 |
| SLC41A2 | 0.792029 | 0.000267 | 0.001679 |
| ENSAPLG00000015531 | -0.78274 | 0.000267 | 0.001681 |
| ENSAPLG00000011770 | -1.05859 | 0.000268 | 0.001682 |
| LAMTOR5 | 0.874785 | 0.000267 | 0.001682 |
| ARHGEF12 | 0.878582 | 0.000268 | 0.001685 |
| SLC7A5 | -0.65766 | 0.00027 | 0.001698 |
| EIF2B2 | -0.79347 | 0.000271 | 0.001703 |
| PPARG | -0.79478 | 0.000274 | 0.001718 |
| TBX22 | 5.821919 | 0.000274 | 0.001718 |
| ELF1 | 0.686289 | 0.000275 | 0.001721 |
| SLC25A3 | -0.59523 | 0.000275 | 0.001721 |
| PRDM2 | 0.704972 | 0.000278 | 0.00174 |
| TULP4 | 1.64775 | 0.00028 | 0.001751 |
| PFKL | -0.72356 | 0.000282 | 0.001763 |
| CTSS | -1.36706 | 0.000284 | 0.001772 |
| HIVEP1 | 0.741824 | 0.000285 | 0.001779 |
| COL6A2 | -0.76959 | 0.000286 | 0.001785 |
| ADAM8 | -0.66573 | 0.000287 | 0.001789 |
| GBE1 | -0.69227 | 0.000288 | 0.001792 |
| ID4 | 0.699439 | 0.000291 | 0.001811 |
| GFOD2 | 1.240554 | 0.000291 | 0.001814 |
| MXI1 | 0.651134 | 0.000293 | 0.001823 |
| FDPS | -0.83393 | 0.000294 | 0.00183 |
| PRKD3 | 0.891841 | 0.000296 | 0.00184 |
| PBX1 | 0.957037 | 0.000296 | 0.001841 |
| TIMP3 | -1.02685 | 0.000299 | 0.001855 |
| TEKT2 | 3.151486 | 0.000301 | 0.001867 |
| PGRMC2 | -0.66267 | 0.000301 | 0.001867 |
| TMCC2 | 1.411446 | 0.000302 | 0.001869 |
| D2HGDH | -1.38377 | 0.000303 | 0.001874 |
| TRA2B | -0.6741 | 0.000305 | 0.001886 |
| PKHD1 | 1.438269 | 0.000305 | 0.001886 |
| TPBG | -1.08046 | 0.000305 | 0.001886 |
| ENSAPLG00000027187 | -1.20082 | 0.000305 | 0.001886 |
| GNAO1 | 0.918558 | 0.000306 | 0.001887 |
| ACAP2 | -0.7049 | 0.000307 | 0.001896 |
| BMP2 | -0.62787 | 0.000308 | 0.0019 |
| MARCHF2 | 0.741455 | 0.000309 | 0.001906 |
| EEFSEC | -0.9445 | 0.000311 | 0.001917 |
| ENSAPLG00000011112 | 1.621596 | 0.000313 | 0.001926 |
| UTP6 | -0.65848 | 0.000316 | 0.001944 |
| TRMT9B | -0.77588 | 0.000316 | 0.001944 |
| TYR | 5.86467 | 0.000316 | 0.001944 |
| FAM168A | 0.949907 | 0.000321 | 0.001974 |
| RPP40 | -0.82522 | 0.000322 | 0.001976 |
| ENSAPLG00000006069 | 0.611224 | 0.000324 | 0.001988 |
| PSEN1 | -0.76009 | 0.000326 | 0.002002 |
| MBTD1 | 0.71215 | 0.000328 | 0.002011 |
| FKBP9 | -0.66566 | 0.000329 | 0.002016 |
| SEC22B | -0.62453 | 0.000329 | 0.002016 |
| G3BP2 | -0.6244 | 0.00033 | 0.002022 |
| SCYL3 | -0.64577 | 0.000331 | 0.002025 |
| ENSAPLG00000005706 | 0.849375 | 0.000332 | 0.002029 |
| TWSG1 | -0.60459 | 0.000333 | 0.002035 |
| IPO11 | -0.79363 | 0.000333 | 0.002036 |
| ENSAPLG00000003459 | 1.656942 | 0.000334 | 0.002039 |
| HERC4 | -0.6439 | 0.000334 | 0.002039 |
| COL8A1 | -1.01834 | 0.000335 | 0.002044 |
| RERG | 0.679536 | 0.000337 | 0.002051 |
| NR3C2 | 0.872886 | 0.000337 | 0.002051 |
| GRAMD4 | 0.659286 | 0.000337 | 0.002051 |
| SLC6A20 | 5.847416 | 0.000337 | 0.002051 |
| ENSAPLG00000025635 | 1.378926 | 0.000339 | 0.002062 |
| ERO1B | -0.68875 | 0.000341 | 0.00207 |
| BRD8 | -0.77787 | 0.000341 | 0.002072 |
| SLC25A13 | -0.67997 | 0.000342 | 0.002074 |
| TRPV3 | 1.276781 | 0.000346 | 0.002099 |
| RPS15A | 0.662039 | 0.000346 | 0.002099 |
| FIGNL1 | -1.77539 | 0.000347 | 0.002105 |
| EIF2S1 | -0.63137 | 0.000348 | 0.002108 |
| EIF2A | -0.63757 | 0.000349 | 0.002109 |
| ENSAPLG00000007729 | -1.42734 | 0.000349 | 0.002113 |
| ADGRV1 | 0.950851 | 0.00035 | 0.002114 |
| FAM20C | 0.88112 | 0.000351 | 0.002119 |
| ENSAPLG00000025723 | -0.80386 | 0.000356 | 0.002152 |
| MRPS22 | -0.73948 | 0.000358 | 0.002159 |
| TAF3 | 0.732368 | 0.000358 | 0.002161 |
| ENSAPLG00000002923 | 0.806742 | 0.000365 | 0.002198 |
| METTL22 | 0.795873 | 0.000365 | 0.002198 |
| RARB | 1.423139 | 0.000366 | 0.002207 |
| MICU2 | -0.66822 | 0.000368 | 0.002215 |
| ENSAPLG00000013751 | -0.68686 | 0.000373 | 0.002241 |
| ZDHHC7 | 0.656643 | 0.000373 | 0.002244 |
| ENSAPLG00000026691 | -0.57058 | 0.000374 | 0.002244 |
| TMEM129 | -0.85703 | 0.000374 | 0.002248 |
| STK25 | 0.702387 | 0.000375 | 0.002252 |
| L3HYPDH | -1.13728 | 0.000376 | 0.002255 |
| STX1A | 0.990309 | 0.000376 | 0.002256 |
| MID1IP1 | 0.805593 | 0.000379 | 0.002271 |
| NR4A1 | 1.587461 | 0.000379 | 0.002272 |
| CASK | -0.70687 | 0.000383 | 0.002291 |
| ZYG11A | -0.76714 | 0.000383 | 0.002294 |
| SERHL2 | -0.76429 | 0.000384 | 0.002297 |
| ENSAPLG00000007708 | -1.23393 | 0.000385 | 0.002299 |
| RFNG | -0.81359 | 0.000385 | 0.002299 |
| ENSAPLG00000030527 | 0.688971 | 0.000386 | 0.002301 |
| ENSAPLG00000003869 | 0.59375 | 0.000389 | 0.002319 |
| DYRK3 | 0.831524 | 0.000389 | 0.002321 |
| DAB2 | 0.6691 | 0.00039 | 0.002327 |
| STON2 | 1.161204 | 0.000391 | 0.002329 |
| BRWD3 | 0.674014 | 0.000394 | 0.002344 |
| XYLB | -0.79708 | 0.000397 | 0.002364 |
| PCNP | 0.665288 | 0.000399 | 0.002373 |
| NFATC2 | 0.817418 | 0.000404 | 0.002401 |
| ITGA1 | -0.7319 | 0.000406 | 0.002408 |
| F2RL2 | 0.735732 | 0.000406 | 0.002409 |
| UBE2QL1 | -1.69889 | 0.000406 | 0.002409 |
| ENSAPLG00000009918 | -0.62224 | 0.000409 | 0.002423 |
| GALC | -0.83579 | 0.00041 | 0.002431 |
| RNF141 | 0.676933 | 0.00041 | 0.002431 |
| NAV2 | 1.085833 | 0.000412 | 0.002435 |
| ENSAPLG00000015238 | -0.71932 | 0.000412 | 0.002435 |
| ENSAPLG00000016521 | 1.611142 | 0.000412 | 0.002435 |
| AFG3L2 | -0.58404 | 0.000415 | 0.002455 |
| TCF3 | 0.586897 | 0.000416 | 0.002459 |
| UQCC1 | -0.61827 | 0.000421 | 0.002485 |
| ENSAPLG00000020305 | 1.029945 | 0.000422 | 0.002489 |
| MANEAL | -1.28918 | 0.000422 | 0.002489 |
| ENSAPLG00000024437 | -0.6871 | 0.000424 | 0.002496 |
| MCCC1 | -1.33092 | 0.000429 | 0.002525 |
| PPP4R2 | 0.667281 | 0.000429 | 0.002529 |
| MEGF11 | 1.990317 | 0.00043 | 0.002529 |
| ENSAPLG00000024581 | 0.636437 | 0.00043 | 0.002529 |
| SLC9A9 | -1.19721 | 0.000432 | 0.002541 |
| TDP1 | -0.87615 | 0.000436 | 0.002562 |
| ENSAPLG00000010480 | 1.042201 | 0.000437 | 0.002565 |
| MKKS | 0.722729 | 0.000437 | 0.002566 |
| TSC22D3 | 1.630527 | 0.000438 | 0.002568 |
| MCM9 | -1.7062 | 0.000439 | 0.00257 |
| TMEM184A | 1.54966 | 0.000439 | 0.00257 |
| TSC22D2 | 0.796116 | 0.000439 | 0.00257 |
| ENSAPLG00000023711 | 0.963852 | 0.000442 | 0.00259 |
| OSCP1 | -0.88091 | 0.000444 | 0.002595 |
| GGH | -0.70997 | 0.000444 | 0.002595 |
| ZBTB47 | 1.578171 | 0.000445 | 0.002602 |
| SPRY4 | 3.806124 | 0.000446 | 0.002604 |
| SMARCAD1 | -0.72555 | 0.000447 | 0.002609 |
| BCL9L | 1.890711 | 0.000447 | 0.002609 |
| SARAF | -0.69851 | 0.00045 | 0.002627 |
| TXLNB | 4.084845 | 0.000451 | 0.002628 |
| MRPL28 | -1.18992 | 0.000451 | 0.002628 |
| ADPGK | -0.74162 | 0.000455 | 0.002647 |
| SGCB | -0.62116 | 0.000456 | 0.002654 |
| KDM2A | 0.755864 | 0.000457 | 0.002659 |
| PRICKLE1 | -0.95285 | 0.000457 | 0.002659 |
| TMEM248 | 0.673746 | 0.00046 | 0.002673 |
| ENSAPLG00000029535 | 1.016275 | 0.000461 | 0.002677 |
| LRRC8C | -0.66388 | 0.000465 | 0.002697 |
| PKIA | 1.057923 | 0.000466 | 0.002704 |
| SEPTIN6 | -0.58727 | 0.000468 | 0.002711 |
| METTL6 | 0.633093 | 0.000469 | 0.002715 |
| ENSAPLG00000004588 | 0.638719 | 0.000469 | 0.002715 |
| KIF4A | 1.348401 | 0.000469 | 0.002715 |
| BTRC | -0.8504 | 0.000471 | 0.002723 |
| POMK | -0.7024 | 0.000471 | 0.002725 |
| CELF2 | -0.72494 | 0.000472 | 0.002725 |
| TUBB1 | 1.165123 | 0.000473 | 0.00273 |
| TTK | -0.9414 | 0.000475 | 0.002743 |
| ROR2 | -0.99328 | 0.000476 | 0.002744 |
| ZNF827 | 1.945179 | 0.000477 | 0.00275 |
| ENSAPLG00000005608 | 1.082994 | 0.000479 | 0.002761 |
| FAM185A | -0.72328 | 0.000479 | 0.002761 |
| SMPDL3A | -0.66164 | 0.00048 | 0.002762 |
| XDH | 3.841662 | 0.00048 | 0.002762 |
| ENSAPLG00000002886 | -1.02103 | 0.000481 | 0.002766 |
| NT5C1A | 3.656902 | 0.000484 | 0.00278 |
| PPP4R1 | 0.658607 | 0.000486 | 0.002794 |
| HOXD8 | 1.685029 | 0.000487 | 0.002797 |
| RNF220 | 0.723902 | 0.000489 | 0.002805 |
| MMP24 | 0.819106 | 0.00049 | 0.002806 |
| UNC93B1 | -0.93863 | 0.00049 | 0.002806 |
| DNAJC24 | -1.07245 | 0.00049 | 0.002806 |
| ENSAPLG00000031231 | 5.744408 | 0.000492 | 0.002816 |
| BDH1 | -0.70985 | 0.000496 | 0.002841 |
| ENSAPLG00000019504 | 0.879716 | 0.000498 | 0.002849 |
| KRAS | 0.765717 | 0.000502 | 0.002871 |
| ENSAPLG00000008830 | -1.1973 | 0.000503 | 0.002875 |
| ENSAPLG00000029764 | 1.828137 | 0.000503 | 0.002876 |
| ENSAPLG00000013916 | 4.620164 | 0.000505 | 0.002885 |
| TMEM178B | 0.944023 | 0.000507 | 0.002892 |
| RSPRY1 | 0.795858 | 0.000507 | 0.002892 |
| ST6GALNAC4 | 0.585538 | 0.000508 | 0.002895 |
| DLGAP4 | 0.629677 | 0.000511 | 0.002912 |
| ADCK2 | -1.83378 | 0.000513 | 0.002923 |
| ENSAPLG00000025150 | -0.75249 | 0.000514 | 0.002923 |
| ZDHHC16 | -0.78825 | 0.000514 | 0.002924 |
| SPECC1 | -0.63199 | 0.000517 | 0.002937 |
| LMOD3 | 1.816258 | 0.000517 | 0.002937 |
| PHEX | -1.14804 | 0.000524 | 0.002975 |
| GSDME | -0.78971 | 0.000525 | 0.002978 |
| ENSAPLG00000015426 | -0.78681 | 0.000526 | 0.002983 |
| ENSAPLG00000021773 | 0.737215 | 0.000526 | 0.002983 |
| CREM | 0.832859 | 0.000528 | 0.002991 |
| ENSAPLG00000017604 | 1.558485 | 0.000528 | 0.002991 |
| CLIC2 | 0.706926 | 0.000528 | 0.002992 |
| NEDD9 | 0.679019 | 0.000529 | 0.002992 |
| SCAMP1 | -0.60212 | 0.000529 | 0.002992 |
| ENSAPLG00000004765 | -1.48585 | 0.00053 | 0.002992 |
| MAB21L1 | 1.097584 | 0.000529 | 0.002992 |
| HAL | 3.041824 | 0.000533 | 0.003007 |
| ENSAPLG00000004064 | 1.476164 | 0.000533 | 0.003007 |
| LOXL1 | 0.731811 | 0.000533 | 0.003009 |
| MYEF2 | -0.75359 | 0.000534 | 0.003012 |
| CDK14 | 0.638478 | 0.000536 | 0.003021 |
| TUBA8 | 3.588729 | 0.000536 | 0.003021 |
| NUP93 | 0.58186 | 0.000537 | 0.003022 |
| CACTIN | 0.586615 | 0.00054 | 0.003037 |
| CILK1 | 1.019144 | 0.00054 | 0.003038 |
| SYNGR1 | 0.838078 | 0.000542 | 0.003047 |
| DELE1 | -1.82698 | 0.000543 | 0.003053 |
| DIAPH2 | -0.64355 | 0.000548 | 0.003076 |
| ARFIP2 | 0.649893 | 0.000548 | 0.003077 |
| HMGCS1 | -0.55702 | 0.000555 | 0.003112 |
| STXBP1 | 0.83913 | 0.000559 | 0.003134 |
| RAB9A | 0.637536 | 0.000561 | 0.00314 |
| CTSK | -0.87393 | 0.000561 | 0.00314 |
| LRIT2 | 5.721531 | 0.000561 | 0.003142 |
| GSK3B | 0.666148 | 0.000564 | 0.003155 |
| MPRIP | 0.900306 | 0.000566 | 0.003167 |
| NBR1 | -0.98344 | 0.000568 | 0.003175 |
| ARSK | -1.13596 | 0.000571 | 0.003191 |
| ZC3H13 | 0.600331 | 0.000575 | 0.00321 |
| ENSAPLG00000028225 | 0.834777 | 0.000581 | 0.00324 |
| CCT6B | -0.57808 | 0.000583 | 0.003252 |
| MCMBP | -0.54331 | 0.000585 | 0.003263 |
| ENSAPLG00000010086 | 0.730292 | 0.000586 | 0.003265 |
| LINS1 | -1.97052 | 0.000603 | 0.003357 |
| TENT4B | 0.641761 | 0.000605 | 0.003368 |
| GATA3 | 3.703621 | 0.000606 | 0.003373 |
| ENSAPLG00000025867 | -0.56544 | 0.000609 | 0.003384 |
| CRISPLD2 | 1.185234 | 0.000609 | 0.003386 |
| YWHAZ | -0.65808 | 0.00061 | 0.003387 |
| SLC25A5 | -0.82104 | 0.000617 | 0.003424 |
| NARS2 | -0.83628 | 0.000618 | 0.003431 |
| CAPN9 | 0.791051 | 0.000621 | 0.003445 |
| ENSAPLG00000009361 | 1.757698 | 0.000622 | 0.003448 |
| ENSAPLG00000004917 | 1.096828 | 0.000625 | 0.003464 |
| PTGER4 | 1.276819 | 0.000627 | 0.003475 |
| ENSAPLG00000025374 | -0.64636 | 0.000629 | 0.003479 |
| ENSAPLG00000021250 | 5.705613 | 0.00063 | 0.003488 |
| CANX | -0.54801 | 0.000633 | 0.003497 |
| TPRKB | -0.69741 | 0.000633 | 0.003499 |
| CEP57 | -0.75179 | 0.000636 | 0.003513 |
| ENSAPLG00000005367 | 1.850319 | 0.000638 | 0.00352 |
| FPGT | -0.86875 | 0.00064 | 0.00353 |
| IL1R2 | 0.678617 | 0.000643 | 0.003544 |
| DARS2 | -0.87661 | 0.000644 | 0.00355 |
| ENSAPLG00000015505 | 0.973451 | 0.000645 | 0.003551 |
| NR6A1 | 0.927275 | 0.000645 | 0.003551 |
| ENSAPLG00000004424 | 5.799511 | 0.000646 | 0.003557 |
| RUVBL1 | -0.60568 | 0.00065 | 0.003576 |
| EGLN3 | 1.791932 | 0.000651 | 0.003582 |
| ENSAPLG00000018316 | 1.541253 | 0.000652 | 0.003582 |
| CYP39A1 | -1.00977 | 0.000655 | 0.0036 |
| FBXO44 | -1.1771 | 0.000658 | 0.00361 |
| MRPL41 | -0.72991 | 0.000664 | 0.003646 |
| ENSAPLG00000030810 | -1.02558 | 0.000665 | 0.003646 |
| SMARCA2 | -0.60252 | 0.000666 | 0.003654 |
| GPRC5B | 1.476514 | 0.000669 | 0.003663 |
| ENSAPLG00000028706 | -0.64405 | 0.00067 | 0.003671 |
| ENSAPLG00000012588 | -0.8137 | 0.000676 | 0.0037 |
| NSG1 | -0.82158 | 0.000677 | 0.003701 |
| ENSAPLG00000014522 | -0.7545 | 0.00068 | 0.003721 |
| ARL9 | -0.71962 | 0.000682 | 0.003729 |
| HTATSF1 | -0.65984 | 0.000683 | 0.003729 |
| FRMD3 | 2.531124 | 0.000683 | 0.003729 |
| ENSAPLG00000030819 | -1.17375 | 0.000683 | 0.003729 |
| APOO | -0.6735 | 0.000686 | 0.003738 |
| EPN2 | 0.631014 | 0.000686 | 0.003738 |
| ENSAPLG00000014299 | 1.151136 | 0.000691 | 0.003767 |
| CYP2R1 | -0.88227 | 0.000693 | 0.003773 |
| UGGT2 | -0.80425 | 0.000697 | 0.003797 |
| DCK | -0.83054 | 0.000698 | 0.003798 |
| FH | -0.61817 | 0.000701 | 0.00381 |
| ENSAPLG00000004770 | 0.598109 | 0.000706 | 0.003835 |
| FZD1 | 0.79075 | 0.000706 | 0.003837 |
| MSL1 | 0.824 | 0.00071 | 0.003854 |
| NAT10 | -0.70635 | 0.000712 | 0.003865 |
| RERE | 0.758055 | 0.000714 | 0.003872 |
| TP53BP2 | 0.798215 | 0.000714 | 0.003872 |
| SAV1 | 0.575564 | 0.000715 | 0.003872 |
| C1S | -0.85727 | 0.000719 | 0.003892 |
| OGDHL | 3.70102 | 0.000727 | 0.003934 |
| ENSAPLG00000016289 | -0.67254 | 0.000727 | 0.003935 |
| UBE2I | 0.623089 | 0.00073 | 0.003946 |
| RAPGEF1 | 0.702333 | 0.000732 | 0.003958 |
| CYTIP | 1.659208 | 0.00074 | 0.003999 |
| ENSAPLG00000021626 | 0.780995 | 0.00074 | 0.003999 |
| ATG7 | -0.63862 | 0.000742 | 0.004003 |
| ARMC10 | -1.00921 | 0.000742 | 0.004003 |
| KATNA1 | -0.92438 | 0.000743 | 0.004005 |
| ARHGAP28 | -0.66069 | 0.000747 | 0.004026 |
| SEC61G | 0.763918 | 0.000748 | 0.00403 |
| EPS15L1 | -0.70392 | 0.000755 | 0.004063 |
| ENSAPLG00000016639 | -1.04363 | 0.000755 | 0.004063 |
| TUSC2 | 1.115686 | 0.000755 | 0.004063 |
| PSMC3 | -0.60205 | 0.000756 | 0.004065 |
| LASP1 | 0.758641 | 0.000757 | 0.004066 |
| SGPP1 | -0.99058 | 0.000759 | 0.004076 |
| CCNDBP1 | -0.59566 | 0.000765 | 0.004107 |
| GEN1 | 0.871511 | 0.000766 | 0.00411 |
| KLHL36 | 0.620232 | 0.000766 | 0.00411 |
| GPATCH2L | 0.875452 | 0.000767 | 0.00411 |
| RAPGEF5 | 1.164875 | 0.000768 | 0.00411 |
| LRP6 | 0.689525 | 0.000767 | 0.00411 |
| ENSAPLG00000023413 | 4.510283 | 0.000768 | 0.00411 |
| SESTD1 | -0.59605 | 0.000771 | 0.004125 |
| AKR1A1 | -0.79978 | 0.000771 | 0.004125 |
| USP14 | -0.64496 | 0.000773 | 0.00413 |
| ENSAPLG00000007074 | 0.734871 | 0.000777 | 0.004152 |
| HACL1 | -0.87155 | 0.00078 | 0.004165 |
| C16orf72 | 0.655426 | 0.00078 | 0.004165 |
| METAP1 | -0.62788 | 0.000782 | 0.004172 |
| CNPPD1 | -0.65517 | 0.000785 | 0.004186 |
| PHC2 | 0.745739 | 0.000786 | 0.004189 |
| SLC12A2 | -1.01635 | 0.000787 | 0.004191 |
| LCT | 4.553998 | 0.000789 | 0.004197 |
| MED21 | 0.680512 | 0.000789 | 0.004197 |
| ENSAPLG00000030678 | -0.90716 | 0.000792 | 0.004214 |
| C2orf69 | -0.72185 | 0.000802 | 0.004263 |
| HDLBP | 0.848042 | 0.000809 | 0.004297 |
| GOLGA5 | -0.57836 | 0.000816 | 0.004333 |
| HEATR3 | -0.61817 | 0.000816 | 0.004333 |
| INPP5D | 1.93139 | 0.000817 | 0.004335 |
| KIF5C | -0.90858 | 0.000818 | 0.004337 |
| LGR5 | 2.309514 | 0.00082 | 0.004348 |
| ENSAPLG00000005803 | -0.83336 | 0.000821 | 0.00435 |
| NEPRO | -1.02865 | 0.000823 | 0.004357 |
| ENSAPLG00000029667 | 0.739416 | 0.000823 | 0.004359 |
| ENSAPLG00000010306 | -0.99162 | 0.000825 | 0.004364 |
| ENSAPLG00000011857 | 3.539164 | 0.000826 | 0.004366 |
| INTS10 | -0.57951 | 0.000832 | 0.004399 |
| RPL34 | 0.683968 | 0.000833 | 0.004401 |
| GNG2 | 0.782348 | 0.000833 | 0.004401 |
| CDK10 | -0.72364 | 0.000839 | 0.004429 |
| ENSAPLG00000023922 | 1.495541 | 0.00084 | 0.004429 |
| FUBP1 | -0.56787 | 0.000845 | 0.004455 |
| XPO7 | 0.669482 | 0.000847 | 0.004464 |
| SMARCAL1 | -0.87342 | 0.000849 | 0.00447 |
| ZDHHC9 | 0.560203 | 0.000855 | 0.004501 |
| CYTH1 | 0.614282 | 0.000856 | 0.004505 |
| UBE2V2 | 0.606967 | 0.000862 | 0.004535 |
| ARHGEF3 | -0.63816 | 0.000865 | 0.004547 |
| CLUAP1 | -0.68993 | 0.000866 | 0.004551 |
| BACH2 | 1.307649 | 0.000867 | 0.004556 |
| GALNT11 | 0.552351 | 0.000868 | 0.004558 |
| ENSAPLG00000021804 | 0.740942 | 0.00087 | 0.004563 |
| RAI14 | -0.557 | 0.00087 | 0.004564 |
| WDR70 | -0.63367 | 0.000872 | 0.004569 |
| FNDC4 | 1.005234 | 0.000873 | 0.004574 |
| ASB5 | 0.649376 | 0.000876 | 0.004586 |
| COL12A1 | -0.60378 | 0.000877 | 0.004587 |
| TTC9 | 1.362286 | 0.000877 | 0.004587 |
| IL13RA1 | -1.302 | 0.000885 | 0.004626 |
| PUM2 | 0.577673 | 0.000885 | 0.004626 |
| NARF | 0.893909 | 0.000892 | 0.004659 |
| BCS1L | -0.69421 | 0.000896 | 0.00468 |
| C1orf112 | -1.01538 | 0.000898 | 0.004684 |
| EDN1 | -0.65313 | 0.000898 | 0.004684 |
| ENSAPLG00000016181 | -1.07296 | 0.000898 | 0.004685 |
| PGR | 2.392561 | 0.000899 | 0.004688 |
| DNALI1 | -1.12945 | 0.000904 | 0.004708 |
| RRM2 | -2.00914 | 0.000905 | 0.004714 |
| BEGAIN | 4.042158 | 0.00091 | 0.004734 |
| SEC16A | 0.920418 | 0.00091 | 0.004734 |
| NIT2 | -0.88996 | 0.00091 | 0.004734 |
| ENSAPLG00000022704 | 1.146295 | 0.000913 | 0.004744 |
| ENSAPLG00000030287 | -1.15624 | 0.000914 | 0.004748 |
| NELFA | 0.907873 | 0.000917 | 0.004756 |
| ENSAPLG00000009060 | 0.962458 | 0.000916 | 0.004756 |
| ATP6V1B2 | -0.51821 | 0.000917 | 0.004756 |
| CRNKL1 | -0.54857 | 0.00092 | 0.004768 |
| NAALADL2 | -0.79455 | 0.000922 | 0.004778 |
| PPM1E | 0.66602 | 0.000922 | 0.004778 |
| NR2C2 | 0.682829 | 0.000925 | 0.004791 |
| RFXANK | 0.606248 | 0.000926 | 0.004791 |
| RAPGEF4 | -1.69492 | 0.00093 | 0.00481 |
| ARHGAP10 | -0.61375 | 0.000934 | 0.004826 |
| HSPD1 | -0.60281 | 0.000934 | 0.004827 |
| SOS2 | 0.941427 | 0.00094 | 0.004857 |
| TMEM43 | -0.52827 | 0.000945 | 0.004881 |
| ENSAPLG00000007885 | -1.57724 | 0.000947 | 0.004886 |
| LGALS1 | 0.772654 | 0.000947 | 0.004886 |
| SERP1 | 0.826355 | 0.000954 | 0.004917 |
| MAP3K7CL | 0.8335 | 0.000957 | 0.00493 |
| GABRR1 | 4.457808 | 0.000959 | 0.004938 |
| ENSAPLG00000015060 | 0.755622 | 0.000961 | 0.004949 |
| ENSAPLG00000003643 | -0.52225 | 0.000964 | 0.004959 |
| ENSAPLG00000006994 | 3.588895 | 0.000972 | 0.005 |
| METTL4 | -0.75414 | 0.000974 | 0.005009 |
| TTC26 | -0.67852 | 0.000986 | 0.005068 |
| RTKN2 | 1.662137 | 0.000994 | 0.005105 |
| FILIP1L | 0.698685 | 0.000998 | 0.005122 |
| ENSAPLG00000017150 | 1.7989 | 0.000998 | 0.005122 |
| CCNK | 0.679949 | 0.001 | 0.005128 |
| FAHD1 | -0.82746 | 0.001009 | 0.005172 |
| BPNT1 | -0.77849 | 0.001015 | 0.005202 |
| ENSAPLG00000008904 | 1.285669 | 0.001016 | 0.005202 |
| LCLAT1 | 0.620376 | 0.001017 | 0.005204 |
| TJP1 | -0.72358 | 0.00102 | 0.005219 |
| C2orf76 | 0.828303 | 0.001029 | 0.005261 |
| MPST | -0.6439 | 0.001029 | 0.005261 |
| ENSAPLG00000010371 | -0.62716 | 0.001034 | 0.005279 |
| TBCD | -0.82555 | 0.001033 | 0.005279 |
| ENSAPLG00000003521 | -0.89168 | 0.001035 | 0.005281 |
| SGMS2 | 0.616189 | 0.001035 | 0.005281 |
| ENSAPLG00000007825 | -0.82882 | 0.001037 | 0.005288 |
| OAZ2 | 0.672935 | 0.001038 | 0.005292 |
| CREBL2 | 0.736055 | 0.001046 | 0.005327 |
| ALDOC | -0.58207 | 0.001046 | 0.005327 |
| ENSAPLG00000029063 | -1.14227 | 0.001049 | 0.005338 |
| KPNA4 | -0.51719 | 0.001054 | 0.005364 |
| PDGFRL | -0.70174 | 0.001056 | 0.005371 |
| GIT1 | 1.450605 | 0.001057 | 0.005373 |
| ENSAPLG00000006173 | -0.71018 | 0.00106 | 0.005383 |
| CCDC126 | 0.548823 | 0.00106 | 0.005383 |
| RFX6 | 4.549559 | 0.001063 | 0.005393 |
| ENSAPLG00000008051 | 0.584824 | 0.001068 | 0.005419 |
| PIK3C3 | -0.69368 | 0.001073 | 0.005442 |
| C6orf62 | 0.600983 | 0.001076 | 0.005453 |
| SDF2 | -0.86068 | 0.001077 | 0.005457 |
| ENSAPLG00000016133 | -0.77013 | 0.00108 | 0.005467 |
| TRUB1 | -0.85208 | 0.001081 | 0.005468 |
| PHB | -0.57506 | 0.001081 | 0.005468 |
| LSM5 | 0.643578 | 0.001081 | 0.005469 |
| MAT2B | -0.56226 | 0.001091 | 0.005513 |
| CIDEA | -0.95704 | 0.001093 | 0.005521 |
| ID3 | 0.796661 | 0.001098 | 0.005545 |
| TP53I3 | -0.89137 | 0.001101 | 0.005558 |
| USP31 | 1.067597 | 0.001103 | 0.005563 |
| AGAP3 | 0.603413 | 0.001103 | 0.005563 |
| ENSAPLG00000012564 | -0.62617 | 0.001112 | 0.005605 |
| PM20D1 | -0.83464 | 0.001114 | 0.005614 |
| IGF2BP2 | 0.60284 | 0.001119 | 0.005635 |
| MCEE | -0.94758 | 0.001122 | 0.005644 |
| IREB2 | -0.58456 | 0.001122 | 0.005644 |
| STK17B | -0.68093 | 0.001122 | 0.005645 |
| MAP3K5 | 0.70352 | 0.001124 | 0.00565 |
| HMGA2 | 0.837138 | 0.00113 | 0.005678 |
| CTSB | -0.56576 | 0.001143 | 0.005741 |
| PHETA1 | -0.91373 | 0.001144 | 0.005745 |
| LATS1 | 0.694309 | 0.001149 | 0.005764 |
| IGF2BP3 | -0.72222 | 0.00115 | 0.005765 |
| FRMD4B | 0.82792 | 0.001152 | 0.005772 |
| PACC1 | -0.72483 | 0.001152 | 0.005772 |
| NOC3L | -0.67042 | 0.001154 | 0.00578 |
| RPS6KC1 | -0.80421 | 0.001166 | 0.005836 |
| TMEM169 | 0.722163 | 0.001166 | 0.005836 |
| IFT57 | -0.67283 | 0.00117 | 0.00585 |
| BPNT2 | -0.56191 | 0.00118 | 0.005898 |
| SNCG | 3.119962 | 0.001181 | 0.005899 |
| CRACD | 1.117668 | 0.001186 | 0.005922 |
| ZEB2 | 0.676732 | 0.00119 | 0.005938 |
| ARPP19 | 0.508658 | 0.00119 | 0.005938 |
| URI1 | 0.537957 | 0.001196 | 0.005966 |
| PCOLCE2 | -0.6076 | 0.0012 | 0.005981 |
| FNDC7 | 2.31697 | 0.001202 | 0.00599 |
| EPHB1 | 0.866366 | 0.001206 | 0.006001 |
| PEX5 | -0.72722 | 0.001206 | 0.006001 |
| STRAP | -0.542 | 0.001206 | 0.006001 |
| MCOLN1 | 0.600189 | 0.001208 | 0.006009 |
| CDCA4 | 0.640737 | 0.001212 | 0.006024 |
| ENSAPLG00000023996 | -0.90619 | 0.001212 | 0.006024 |
| SLC4A7 | -0.58182 | 0.001214 | 0.006031 |
| RNF10 | 0.840711 | 0.001218 | 0.006045 |
| SPTLC3 | -0.9301 | 0.001221 | 0.006059 |
| FAM98A | -0.67987 | 0.001222 | 0.006061 |
| ENSAPLG00000019789 | -0.66659 | 0.001227 | 0.006082 |
| FJX1 | 1.558374 | 0.001228 | 0.006084 |
| UQCRB | 0.70457 | 0.001229 | 0.00609 |
| MAMLD1 | 0.712505 | 0.00123 | 0.006092 |
| ENSAPLG00000005846 | 0.786064 | 0.001232 | 0.006095 |
| ENSAPLG00000018473 | -0.73421 | 0.001234 | 0.006104 |
| ENSAPLG00000027541 | 0.849639 | 0.001238 | 0.006121 |
| PPFIBP1 | 0.649911 | 0.001239 | 0.006125 |
| FAM204A | 0.693609 | 0.001244 | 0.006143 |
| ARHGEF6 | 0.611373 | 0.001244 | 0.006143 |
| MAP3K7 | 0.565335 | 0.00125 | 0.006164 |
| GTF2B | -0.69053 | 0.00125 | 0.006164 |
| LMF2 | -1.13857 | 0.001249 | 0.006164 |
| PELO | -0.5955 | 0.001251 | 0.006166 |
| CDC42BPA | 0.681743 | 0.001253 | 0.006172 |
| BBS12 | -0.77279 | 0.001257 | 0.006186 |
| ENSAPLG00000006553 | 1.199023 | 0.001257 | 0.006186 |
| CCT5 | -0.54999 | 0.001258 | 0.006188 |
| SIRT5 | -1.03755 | 0.001263 | 0.006208 |
| CHRNB2 | 1.166889 | 0.001263 | 0.006208 |
| AIMP1 | -0.821 | 0.001269 | 0.006237 |
| SORBS1 | -1.65672 | 0.001283 | 0.006303 |
| RWDD3 | -0.80166 | 0.001285 | 0.006306 |
| GRIA3 | 2.452745 | 0.001293 | 0.006346 |
| SEPTIN11 | -0.54743 | 0.0013 | 0.006377 |
| ACSBG1 | 0.701235 | 0.00131 | 0.00642 |
| BOC | 0.74301 | 0.00131 | 0.00642 |
| DHX40 | -0.74919 | 0.001313 | 0.006429 |
| ME1 | -0.68491 | 0.001317 | 0.006447 |
| PABPC4 | 0.686397 | 0.001323 | 0.006473 |
| OTUD1 | 0.725047 | 0.001323 | 0.006473 |
| DUSP12 | -0.85204 | 0.00133 | 0.006503 |
| AIDA | 0.579119 | 0.001333 | 0.006513 |
| MYLK | -0.7286 | 0.001335 | 0.006517 |
| ENSAPLG00000026904 | 0.946473 | 0.001334 | 0.006517 |
| DUS1L | -0.57643 | 0.001338 | 0.006532 |
| MSN | -0.54186 | 0.001341 | 0.006539 |
| ENSAPLG00000013698 | 0.652038 | 0.001344 | 0.00655 |
| ZNF608 | 1.884045 | 0.001343 | 0.00655 |
| DCAF13 | -0.55914 | 0.00135 | 0.00657 |
| MYO1E | 0.660508 | 0.00135 | 0.00657 |
| NIF3L1 | -0.80102 | 0.00135 | 0.00657 |
| SNX1 | -0.53432 | 0.001351 | 0.006571 |
| ENSAPLG00000021285 | 2.31637 | 0.001351 | 0.006571 |
| ETV4 | 0.561851 | 0.001354 | 0.006583 |
| ATP6AP2 | -0.54578 | 0.001358 | 0.006597 |
| SPATA7 | -1.02087 | 0.001362 | 0.006613 |
| EDNRA | -0.67595 | 0.001364 | 0.006623 |
| BCKDHB | -0.79501 | 0.001373 | 0.006662 |
| ENSAPLG00000002685 | -2.10387 | 0.001376 | 0.006668 |
| SLC9A5 | 1.16351 | 0.001376 | 0.006668 |
| SKIL | 0.65264 | 0.001376 | 0.006668 |
| ABHD17B | -0.60436 | 0.001379 | 0.006678 |
| ENSAPLG00000002945 | -0.95535 | 0.001386 | 0.006709 |
| ZDHHC14 | 0.635774 | 0.001393 | 0.006743 |
| STT3A | -0.57005 | 0.0014 | 0.006772 |
| HTR2C | 0.878357 | 0.001407 | 0.006802 |
| PAPOLA | 0.513645 | 0.00141 | 0.006813 |
| IGF2R | -0.89677 | 0.001411 | 0.006817 |
| ADAM22 | 3.459978 | 0.001412 | 0.006818 |
| ATG13 | 0.73238 | 0.001416 | 0.006835 |
| RCC1 | 1.045122 | 0.001418 | 0.006839 |
| MVK | -0.6285 | 0.001423 | 0.006859 |
| RPAP3 | -0.6297 | 0.001423 | 0.006859 |
| EMC3 | -0.6327 | 0.001426 | 0.006866 |
| MYO9A | 0.941729 | 0.001426 | 0.006866 |
| GLA | -0.742 | 0.00144 | 0.00693 |
| EFNA5 | 1.064403 | 0.001441 | 0.006935 |
| IL1RL2 | 0.704469 | 0.001449 | 0.006969 |
| RGS2 | -0.82333 | 0.00145 | 0.006969 |
| CCDC47 | -0.54551 | 0.001453 | 0.006981 |
| KCNS2 | 2.162344 | 0.00147 | 0.007059 |
| VPS26A | -0.55184 | 0.001476 | 0.007085 |
| ENSAPLG00000018541 | 0.753018 | 0.00148 | 0.007102 |
| TGFB3 | -0.58026 | 0.001483 | 0.007112 |
| ENSAPLG00000020546 | 0.658909 | 0.001483 | 0.007112 |
| ENSAPLG00000018877 | 0.719975 | 0.001487 | 0.007124 |
| LIPA | -0.55261 | 0.001496 | 0.007168 |
| TAB2 | 0.711636 | 0.001497 | 0.007169 |
| GNA12 | 0.671716 | 0.0015 | 0.007177 |
| ENSAPLG00000003727 | 1.084286 | 0.001506 | 0.007197 |
| ENSAPLG00000011995 | -0.60418 | 0.001506 | 0.007197 |
| PIM3 | -0.54426 | 0.001505 | 0.007197 |
| MARK3 | 0.597262 | 0.001511 | 0.007217 |
| RPLP0 | 0.632765 | 0.001512 | 0.007221 |
| SLC37A4 | -0.83515 | 0.001515 | 0.007233 |
| KTN1 | -0.60391 | 0.00152 | 0.007253 |
| TNFAIP8L1 | -1.21879 | 0.001531 | 0.0073 |
| ENSAPLG00000001261 | 0.544459 | 0.001532 | 0.007303 |
| ADNP2 | 0.723895 | 0.001533 | 0.007303 |
| PSPC1 | -0.56356 | 0.001533 | 0.007303 |
| WNT4 | 1.59684 | 0.001535 | 0.007307 |
| HDHD2 | -0.66105 | 0.001539 | 0.007322 |
| COG2 | -0.59524 | 0.00154 | 0.007325 |
| IFFO2 | 0.636889 | 0.001544 | 0.00734 |
| ENSAPLG00000009809 | 0.552372 | 0.001557 | 0.007398 |
| EML5 | 2.752532 | 0.001559 | 0.007404 |
| ETV3 | 0.998193 | 0.001564 | 0.007428 |
| IARS1 | -0.64144 | 0.001569 | 0.007448 |
| COPS9 | 4.301755 | 0.001571 | 0.007451 |
| NADSYN1 | -0.60123 | 0.001574 | 0.007464 |
| YPEL5 | 0.55116 | 0.001575 | 0.007467 |
| UBA5 | -0.72673 | 0.001579 | 0.007484 |
| MED13 | 0.769463 | 0.00158 | 0.007484 |
| ENSAPLG00000008902 | -0.68538 | 0.001582 | 0.007487 |
| TRIO | 0.766701 | 0.001582 | 0.007488 |
| ENSAPLG00000013039 | 0.693853 | 0.001589 | 0.007518 |
| CBFA2T2 | 0.692117 | 0.001593 | 0.00753 |
| STAU2 | -0.66021 | 0.001598 | 0.007552 |
| ENSAPLG00000018685 | 1.369135 | 0.001599 | 0.007552 |
| ALKBH3 | 0.617948 | 0.0016 | 0.007553 |
| ENSAPLG00000003298 | 0.889583 | 0.001611 | 0.007605 |
| ENSAPLG00000011877 | 0.709218 | 0.001612 | 0.007607 |
| CHTOP | 0.658974 | 0.001616 | 0.007621 |
| ENSAPLG00000005376 | 3.88211 | 0.001621 | 0.007642 |
| APPL2 | -0.67845 | 0.001623 | 0.007649 |
| SHANK3 | 1.194045 | 0.001625 | 0.007652 |
| ENSAPLG00000009571 | -1.0292 | 0.001626 | 0.007656 |
| NTPCR | -0.90449 | 0.001638 | 0.007706 |
| ECHDC3 | -1.067 | 0.001639 | 0.007706 |
| ENSAPLG00000030814 | -0.89084 | 0.001639 | 0.007706 |
| MXRA5 | 2.050029 | 0.001648 | 0.007742 |
| PHF3 | 0.588013 | 0.001655 | 0.007773 |
| TWIST2 | 2.051408 | 0.001664 | 0.007811 |
| PTCHD3 | -1.05165 | 0.001669 | 0.007831 |
| ENSAPLG00000004124 | -0.5497 | 0.001681 | 0.007884 |
| ENSAPLG00000006340 | 2.600828 | 0.001687 | 0.00791 |
| CASP3 | -0.58988 | 0.001692 | 0.007931 |
| GAS6 | -0.92758 | 0.001702 | 0.007974 |
| ARV1 | -0.71248 | 0.001705 | 0.007985 |
| RBMS3 | 0.562132 | 0.001712 | 0.008014 |
| GPC3 | -0.81375 | 0.001723 | 0.00806 |
| MB21D2 | -0.88629 | 0.001736 | 0.008118 |
| TGFBR1 | -0.51385 | 0.001744 | 0.008151 |
| TRA2A | -0.56327 | 0.001744 | 0.008151 |
| ENSAPLG00000008001 | -0.53761 | 0.001755 | 0.008197 |
| RPSA | 0.634364 | 0.001761 | 0.008223 |
| ENSAPLG00000003498 | -0.82777 | 0.001766 | 0.00824 |
| SLC12A4 | -0.75162 | 0.001778 | 0.008294 |
| MAP1LC3A | 0.727016 | 0.001779 | 0.008294 |
| TCEANC2 | -0.87009 | 0.001786 | 0.008326 |
| BMP15 | 4.927037 | 0.001793 | 0.008354 |
| GPX7 | -0.91873 | 0.001804 | 0.008402 |
| ALOX5 | -0.9388 | 0.001809 | 0.008417 |
| RFC4 | -0.6569 | 0.001808 | 0.008417 |
| CHRNA3 | 3.846357 | 0.00181 | 0.008419 |
| SLC13A4 | 1.35175 | 0.001812 | 0.008424 |
| SGMS1 | 0.577777 | 0.001815 | 0.008425 |
| WARS1 | -0.54708 | 0.001813 | 0.008425 |
| PHACTR2 | 1.021347 | 0.001814 | 0.008425 |
| GRIP2 | 1.047969 | 0.001817 | 0.008431 |
| TLE3 | 1.573239 | 0.001822 | 0.008454 |
| PALD1 | 0.630265 | 0.001829 | 0.008472 |
| ENSAPLG00000018454 | 0.733289 | 0.001827 | 0.008472 |
| ENSAPLG00000019298 | -0.82611 | 0.001828 | 0.008472 |
| TOP1MT | -0.58915 | 0.001836 | 0.0085 |
| NPRL2 | -0.62334 | 0.001836 | 0.0085 |
| MIIP | -1.02719 | 0.001842 | 0.008523 |
| GFUS | -0.88339 | 0.001846 | 0.008537 |
| MBNL2 | 0.567617 | 0.001849 | 0.008548 |
| ENSAPLG00000006357 | 2.275405 | 0.00185 | 0.008552 |
| DTWD1 | -0.9135 | 0.001861 | 0.008598 |
| ENSAPLG00000018683 | 0.784391 | 0.001862 | 0.008598 |
| RSBN1 | 0.629091 | 0.001868 | 0.008624 |
| AMIGO2 | 1.523225 | 0.001869 | 0.008625 |
| ENSAPLG00000004582 | -1.41681 | 0.001873 | 0.008632 |
| ARFGAP2 | -0.68805 | 0.001872 | 0.008632 |
| PRRC2B | 0.812347 | 0.001876 | 0.008645 |
| ENSAPLG00000022799 | 1.538449 | 0.00188 | 0.008661 |
| MAX | 2.015022 | 0.001891 | 0.008707 |
| ITPRIPL2 | 0.717217 | 0.001895 | 0.008721 |
| POLR2K | 0.784286 | 0.001901 | 0.008745 |
| TUBE1 | 0.743237 | 0.001903 | 0.008752 |
| RALBP1 | 0.5905 | 0.001914 | 0.008794 |
| ENSAPLG00000015697 | 0.81248 | 0.001914 | 0.008794 |
| UROD | -0.80491 | 0.001917 | 0.008804 |
| ENSAPLG00000007167 | 0.678894 | 0.001923 | 0.008825 |
| PFDN4 | 0.613854 | 0.001923 | 0.008825 |
| INTS12 | -0.65898 | 0.001925 | 0.008827 |
| ENSAPLG00000012987 | 0.623753 | 0.001926 | 0.008827 |
| ENSAPLG00000019806 | 1.998742 | 0.001926 | 0.008827 |
| TFB1M | -0.78601 | 0.001944 | 0.008903 |
| AHRR | 0.844139 | 0.001961 | 0.008978 |
| IFT46 | 0.615164 | 0.001963 | 0.008984 |
| HDAC10 | -0.69366 | 0.001968 | 0.009001 |
| NLGN1 | -1.37939 | 0.001971 | 0.009015 |
| ENSAPLG00000024173 | -0.92273 | 0.001974 | 0.009024 |
| ALAD | -1.07628 | 0.001988 | 0.009084 |
| TLK1 | -0.64371 | 0.001991 | 0.009093 |
| DNAJA2 | -0.52605 | 0.001993 | 0.009098 |
| ENSAPLG00000012312 | -0.83413 | 0.001996 | 0.009105 |
| NEK7 | -0.57366 | 0.001996 | 0.009105 |
| AGO1 | 0.794074 | 0.002004 | 0.009138 |
| ENSAPLG00000003513 | 0.801022 | 0.002011 | 0.009165 |
| TBC1D15 | -0.5349 | 0.002013 | 0.009172 |
| LBH | 0.466389 | 0.002025 | 0.009221 |
| SOX5 | -0.89544 | 0.002039 | 0.009279 |
| MMP27 | -1.14586 | 0.002039 | 0.009279 |
| CEP350 | 0.793416 | 0.00204 | 0.00928 |
| ULK2 | 0.660769 | 0.002042 | 0.009282 |
| GTF2H1 | 0.587655 | 0.002046 | 0.009296 |
| IQSEC1 | 0.696347 | 0.002048 | 0.009301 |
| CCNF | 0.667739 | 0.002058 | 0.009342 |
| ENSAPLG00000013790 | -0.63945 | 0.002063 | 0.009361 |
| DLL1 | 0.864561 | 0.002064 | 0.009363 |
| PPP2CB | 0.539812 | 0.002066 | 0.009369 |
| DDX59 | 0.593891 | 0.002068 | 0.009375 |
| SLC25A21 | -1.12263 | 0.00207 | 0.009381 |
| PSMD1 | -0.48232 | 0.002078 | 0.009408 |
| OTUB1 | -0.58774 | 0.002078 | 0.009408 |
| CHCHD7 | 0.806723 | 0.002093 | 0.00947 |
| PKM | -0.53781 | 0.002101 | 0.009483 |
| MBTPS1 | 0.619041 | 0.002097 | 0.009483 |
| ENSAPLG00000006354 | 1.541889 | 0.0021 | 0.009483 |
| SLC4A5 | 3.751908 | 0.0021 | 0.009483 |
| FAM13C | -1.16803 | 0.0021 | 0.009483 |
| ENSAPLG00000027048 | 1.613964 | 0.0021 | 0.009483 |
| TRMT1L | -0.55772 | 0.002102 | 0.009485 |
| WSB2 | 0.524154 | 0.00211 | 0.009515 |
| SNX11 | -0.69656 | 0.002116 | 0.00954 |
| SERTM1 | 1.015 | 0.002123 | 0.009569 |
| ENSAPLG00000005844 | -0.64644 | 0.002127 | 0.009581 |
| FDX1 | -0.71407 | 0.002129 | 0.009587 |
| POLR2F | 0.740272 | 0.002137 | 0.009619 |
| NSUN2 | -0.54702 | 0.002141 | 0.009634 |
| MAPK12 | -1.19956 | 0.002142 | 0.009634 |
| FLAD1 | -0.89742 | 0.002144 | 0.009638 |
| ENSAPLG00000006799 | -0.56567 | 0.002146 | 0.009642 |
| UBAC2 | 0.576198 | 0.002151 | 0.00966 |
| PABIR2 | 0.531174 | 0.002151 | 0.00966 |
| ENSAPLG00000024940 | 0.815085 | 0.002152 | 0.00966 |
| GYPC | 0.940926 | 0.002157 | 0.009676 |
| EFEMP1 | -0.54746 | 0.002159 | 0.009678 |
| FKBP4 | -0.52168 | 0.002159 | 0.009678 |
| ENSAPLG00000004972 | 0.687635 | 0.002162 | 0.009689 |
| ENSAPLG00000005834 | -0.6097 | 0.002166 | 0.009701 |
| ANKRD13D | 0.645038 | 0.002167 | 0.009702 |
| HIKESHI | 0.670689 | 0.002168 | 0.009705 |
| LRRC3B | -1.0833 | 0.002178 | 0.009741 |
| CETP | 1.525825 | 0.002178 | 0.009741 |
| CHRM4 | 1.937811 | 0.002182 | 0.009751 |
| RNF14 | -0.68122 | 0.002187 | 0.00977 |
| CDS2 | -0.58637 | 0.002189 | 0.009776 |
| MFSD11 | 0.579124 | 0.002199 | 0.009816 |
| MRPL21 | 0.597247 | 0.0022 | 0.009817 |
| TADA1 | -0.65437 | 0.002206 | 0.00984 |
| FAM78B | 1.587781 | 0.002216 | 0.009882 |
| ISCA1 | 0.698253 | 0.002219 | 0.009891 |
| GJC2 | 0.767123 | 0.002226 | 0.009917 |
| MBP | 0.793312 | 0.002227 | 0.009917 |
| PPM1K | -0.64079 | 0.00223 | 0.009927 |
| AK2 | -0.57173 | 0.002231 | 0.009927 |
| GP1BB | 2.34595 | 0.002234 | 0.009934 |
| SPARC | -0.60331 | 0.002234 | 0.009934 |
| SLC9A7 | 0.63019 | 0.002239 | 0.009951 |
| STK38L | 0.58681 | 0.002241 | 0.009954 |
| KANSL3 | 0.56867 | 0.002246 | 0.009973 |
| WASF2 | 1.04457 | 0.002253 | 0.010001 |
| ANKLE2 | 0.652812 | 0.002254 | 0.010003 |
| SNRNP40 | -0.54742 | 0.00226 | 0.010019 |
| UFSP2 | -0.51337 | 0.002259 | 0.010019 |
| FERMT1 | -0.73148 | 0.002262 | 0.010026 |
| NRBF2 | -0.64338 | 0.002276 | 0.010084 |
| MRPL1 | -0.79521 | 0.002285 | 0.01012 |
| ENSAPLG00000001572 | 1.478495 | 0.002299 | 0.010177 |
| ARNT | 0.968046 | 0.002302 | 0.010184 |
| SWAP70 | 0.548549 | 0.002306 | 0.0102 |
| BEND7 | 0.982724 | 0.002311 | 0.010215 |
| RELN | -1.11121 | 0.002315 | 0.010231 |
| ENPP4 | -0.7478 | 0.002318 | 0.01024 |
| NKD1 | 1.369929 | 0.002333 | 0.0103 |
| CEPT1 | -0.53038 | 0.002338 | 0.010314 |
| PIGU | -0.59009 | 0.002337 | 0.010314 |
| ACOT11 | 0.65025 | 0.002344 | 0.010339 |
| RSAD2 | 2.54187 | 0.002356 | 0.010387 |
| CYB5R2 | -0.71082 | 0.002364 | 0.010415 |
| ENSAPLG00000014992 | -0.88664 | 0.00237 | 0.010437 |
| AURKA | -1.04871 | 0.002371 | 0.010439 |
| CLTC | -0.71251 | 0.002372 | 0.010439 |
| HMBS | -0.61379 | 0.002376 | 0.010451 |
| ARHGEF7 | 0.543744 | 0.002377 | 0.010454 |
| ENSAPLG00000020051 | -0.6756 | 0.002385 | 0.010484 |
| PNISR | 0.633344 | 0.002388 | 0.010489 |
| ETNK2 | 1.753511 | 0.002387 | 0.010489 |
| SMAD3 | 0.738641 | 0.002389 | 0.01049 |
| UHRF1BP1L | 0.602504 | 0.002395 | 0.010513 |
| LIMCH1 | -0.85422 | 0.002404 | 0.010545 |
| EOMES | -2.03523 | 0.00241 | 0.010567 |
| SMOX | 0.533475 | 0.00242 | 0.010606 |
| ACTR5 | -0.71335 | 0.00242 | 0.010606 |
| AP3D1 | 0.544164 | 0.002422 | 0.01061 |
| CNP | 0.831852 | 0.002429 | 0.010631 |
| MTHFD1L | -0.82324 | 0.002428 | 0.010631 |
| GLTP | -0.88183 | 0.002433 | 0.010643 |
| LRP2 | 1.940055 | 0.002456 | 0.010741 |
| PACSIN2 | -0.54088 | 0.002458 | 0.010745 |
| ATP5PB | -0.62091 | 0.002465 | 0.01077 |
| ENSAPLG00000020705 | -0.86297 | 0.002467 | 0.010776 |
| CRIPT | 0.597105 | 0.00247 | 0.010786 |
| COL8A2 | -0.71973 | 0.002483 | 0.010838 |
| TNFSF13B | 2.933492 | 0.002487 | 0.010848 |
| GALM | -0.66528 | 0.002488 | 0.01085 |
| HOXA3 | 1.308142 | 0.002503 | 0.01091 |
| ENSAPLG00000009349 | -0.48837 | 0.002504 | 0.010912 |
| ENSAPLG00000002088 | 4.191438 | 0.002513 | 0.010946 |
| ENSAPLG00000011319 | 0.829572 | 0.002528 | 0.011004 |
| HNRNPM | -0.52968 | 0.002535 | 0.011034 |
| ADGRL2 | -0.98614 | 0.002537 | 0.011037 |
| ENSAPLG00000029470 | 3.262023 | 0.002542 | 0.011052 |
| SIAH3 | 4.272015 | 0.002549 | 0.01108 |
| YPEL1 | 0.612396 | 0.002559 | 0.011117 |
| WDR26 | -0.69045 | 0.002563 | 0.011131 |
| PHB2 | -0.50157 | 0.002569 | 0.011155 |
| ITGA11 | -0.65904 | 0.002572 | 0.011159 |
| PPA1 | -0.55229 | 0.002572 | 0.011159 |
| SMARCE1 | 0.518252 | 0.002575 | 0.011166 |
| ZSWIM5 | 0.668467 | 0.00259 | 0.011226 |
| SCARF1 | 3.044322 | 0.0026 | 0.011266 |
| ENSAPLG00000011821 | 0.670614 | 0.002604 | 0.011277 |
| TMEM33 | -0.49179 | 0.002626 | 0.011371 |
| LZTS2 | 0.767605 | 0.002634 | 0.0114 |
| EPAS1 | 0.796453 | 0.002643 | 0.011435 |
| SLC25A38 | 0.948739 | 0.00266 | 0.011502 |
| SCRN3 | -0.63914 | 0.002666 | 0.011522 |
| ENSAPLG00000012742 | -0.56563 | 0.002671 | 0.011541 |
| ENSAPLG00000027775 | -0.65508 | 0.002676 | 0.011557 |
| DLEC1 | 0.711764 | 0.002677 | 0.01156 |
| ENSAPLG00000020856 | -0.52623 | 0.002681 | 0.011571 |
| THRB | 0.580234 | 0.002686 | 0.01159 |
| COX18 | -0.62652 | 0.002699 | 0.011637 |
| ENSAPLG00000008548 | 0.641233 | 0.002715 | 0.011704 |
| UVRAG | 0.559776 | 0.002717 | 0.011706 |
| PLAGL2 | 0.748738 | 0.002719 | 0.011711 |
| INO80D | 0.797157 | 0.002738 | 0.011788 |
| PRPF40A | -0.53583 | 0.002746 | 0.011813 |
| PLEKHM2 | 0.827921 | 0.002746 | 0.011813 |
| TSPAN14 | -0.55229 | 0.002751 | 0.01183 |
| NFYB | -0.60532 | 0.002763 | 0.011878 |
| TECTA | 3.43907 | 0.00277 | 0.011905 |
| MAP4K4 | 0.654327 | 0.002779 | 0.011936 |
| TMEM59 | -0.68282 | 0.002782 | 0.011946 |
| USP13 | 2.711901 | 0.002788 | 0.011966 |
| MARF1 | 0.718818 | 0.002791 | 0.011975 |
| RIOX1 | -0.67775 | 0.002794 | 0.011982 |
| TPRA1 | -0.75725 | 0.002796 | 0.011985 |
| TRABD | -0.49285 | 0.002797 | 0.011986 |
| ENSAPLG00000011046 | 0.541848 | 0.002815 | 0.012058 |
| MBNL1 | 0.793332 | 0.002819 | 0.012071 |
| NOS2 | 0.977988 | 0.002822 | 0.012079 |
| CDH20 | 1.555176 | 0.002834 | 0.012107 |
| NDUFAF1 | 0.504887 | 0.002832 | 0.012107 |
| LRIG1 | 1.016491 | 0.002834 | 0.012107 |
| ENSAPLG00000028515 | -1.47128 | 0.002832 | 0.012107 |
| ENSAPLG00000029553 | -0.63234 | 0.002833 | 0.012107 |
| GATD1 | -0.80201 | 0.00284 | 0.012127 |
| ADCY3 | 2.964317 | 0.002842 | 0.012128 |
| RHOJ | 0.973192 | 0.002843 | 0.012128 |
| AP5Z1 | -0.62574 | 0.002852 | 0.012164 |
| BSG | -0.53847 | 0.002858 | 0.012186 |
| PUM3 | -0.54143 | 0.002879 | 0.01227 |
| ENSAPLG00000030615 | 1.189076 | 0.00288 | 0.01227 |
| SPATS2L | -0.66875 | 0.002902 | 0.012355 |
| ZNF518A | 0.620663 | 0.002903 | 0.012355 |
| NSUN4 | -0.68137 | 0.002906 | 0.012366 |
| SARDH | -0.94257 | 0.002908 | 0.01237 |
| RNF185 | 0.506178 | 0.002916 | 0.0124 |
| PARD6A | 0.740682 | 0.002918 | 0.012403 |
| TMEM87A | -0.47915 | 0.002931 | 0.012452 |
| KDM4C | -0.79901 | 0.002944 | 0.012503 |
| SLC35F5 | -0.57542 | 0.002945 | 0.012503 |
| NEFL | 1.065946 | 0.002966 | 0.012585 |
| RDH14 | -0.70108 | 0.002976 | 0.012625 |
| ENSAPLG00000014617 | 4.745055 | 0.00298 | 0.012634 |
| SLC40A1 | 0.750133 | 0.002987 | 0.012661 |
| CLIC6 | 4.297648 | 0.002989 | 0.012662 |
| ENSAPLG00000010495 | -0.59334 | 0.002993 | 0.012675 |
| NAA25 | -0.51867 | 0.003007 | 0.012729 |
| METTL15 | -0.79233 | 0.003012 | 0.012748 |
| HDC | 3.881392 | 0.003014 | 0.012752 |
| MFSD8 | -0.82927 | 0.003018 | 0.012762 |
| TLE4 | 0.627648 | 0.00303 | 0.012806 |
| FRMPD3 | 3.234187 | 0.003049 | 0.012882 |
| GPX1 | 0.73366 | 0.003057 | 0.012913 |
| SLC7A14 | 3.719809 | 0.003073 | 0.012973 |
| SMARCA1 | -0.61601 | 0.003086 | 0.013024 |
| EIPR1 | -0.57252 | 0.003087 | 0.013024 |
| CANT1 | -0.63592 | 0.003098 | 0.013065 |
| ENSAPLG00000019867 | 0.655661 | 0.003112 | 0.013119 |
| CCSER1 | -0.77265 | 0.003118 | 0.013139 |
| KIF20A | -1.406 | 0.003123 | 0.013156 |
| HMOX2 | -0.58238 | 0.003141 | 0.013226 |
| CCDC12 | 0.635841 | 0.003157 | 0.01329 |
| ENSAPLG00000004098 | 0.685236 | 0.003168 | 0.013329 |
| CAPG | -0.70156 | 0.003173 | 0.013344 |
| FAM3C | -0.56015 | 0.00318 | 0.013371 |
| UBE2D3 | 0.530515 | 0.003193 | 0.013417 |
| SLC25A14 | 0.509091 | 0.003195 | 0.013422 |
| TRIP13 | -0.81809 | 0.003198 | 0.013428 |
| ENSAPLG00000001730 | -0.87374 | 0.003215 | 0.01349 |
| PHACTR3 | 1.104012 | 0.003214 | 0.01349 |
| PLS3 | -0.45519 | 0.00322 | 0.013506 |
| PTCD3 | -0.68376 | 0.003226 | 0.013522 |
| TMEM266 | 1.364 | 0.003226 | 0.013522 |
| ENSAPLG00000004298 | 1.096123 | 0.003231 | 0.013538 |
| PWP1 | -0.53858 | 0.003239 | 0.013565 |
| FSTL5 | 1.454365 | 0.00326 | 0.013649 |
| ENSAPLG00000014237 | 0.790436 | 0.003262 | 0.013651 |
| ENSAPLG00000019315 | 0.795587 | 0.003282 | 0.013729 |
| AUP1 | -0.53924 | 0.003291 | 0.01376 |
| ENSAPLG00000003490 | 1.065803 | 0.003297 | 0.013782 |
| ENSAPLG00000007401 | 0.807344 | 0.003327 | 0.0139 |
| LUM | -0.79951 | 0.003328 | 0.0139 |
| ENSAPLG00000016512 | 0.86471 | 0.003338 | 0.013938 |
| TRMT44 | -0.65201 | 0.003342 | 0.013947 |
| PDZRN4 | -1.14379 | 0.00335 | 0.013975 |
| RNF130 | 0.475703 | 0.003352 | 0.01398 |
| FAM222A | 4.784149 | 0.003358 | 0.013999 |
| RAB33B | 0.602481 | 0.003373 | 0.014057 |
| ENSAPLG00000009817 | 1.717289 | 0.003393 | 0.014135 |
| FNDC5 | 1.004532 | 0.003402 | 0.014165 |
| ENSAPLG00000009985 | -0.67015 | 0.003404 | 0.014167 |
| TOPBP1 | -0.87928 | 0.003405 | 0.014168 |
| GHR | 0.845798 | 0.003411 | 0.014187 |
| CXXC5 | 0.927797 | 0.003414 | 0.014196 |
| AKAP6 | 0.866892 | 0.00342 | 0.014212 |
| ENSAPLG00000017524 | -0.5658 | 0.003422 | 0.014219 |
| ACOT7 | 0.5267 | 0.003434 | 0.014259 |
| ENSAPLG00000005925 | 0.647739 | 0.003439 | 0.014276 |
| DNAJB9 | -0.6519 | 0.003441 | 0.01428 |
| KIAA1328 | 0.843496 | 0.00345 | 0.014312 |
| RHEB | 0.508741 | 0.003464 | 0.014364 |
| WRAP73 | -0.77484 | 0.003473 | 0.014388 |
| ERAP1 | -0.907 | 0.003474 | 0.014388 |
| ZNF207 | -0.50144 | 0.003471 | 0.014388 |
| ENSAPLG00000012884 | -0.84384 | 0.003493 | 0.014461 |
| SNX21 | 0.54121 | 0.003507 | 0.014516 |
| CAPN2 | -0.52856 | 0.003514 | 0.014536 |
| TRAPPC13 | -0.56222 | 0.003517 | 0.014545 |
| ZBTB14 | 0.829859 | 0.003522 | 0.014558 |
| ENSAPLG00000017467 | 0.573255 | 0.003536 | 0.014611 |
| TMED4 | -0.51551 | 0.003537 | 0.014611 |
| ENSAPLG00000013000 | -0.48174 | 0.00354 | 0.014618 |
| POU2F3 | 2.537396 | 0.003544 | 0.014627 |
| ENSAPLG00000003738 | 0.86498 | 0.00358 | 0.01477 |
| NSMCE2 | 0.609629 | 0.003586 | 0.01479 |
| ATN1 | 1.167689 | 0.003604 | 0.014857 |
| SLC16A8 | 4.125483 | 0.003617 | 0.014906 |
| DAGLA | 0.925646 | 0.003627 | 0.014941 |
| ENSAPLG00000003917 | -0.58251 | 0.003631 | 0.014953 |
| RAD51 | -0.89791 | 0.00364 | 0.014987 |
| CALML3 | 4.093136 | 0.003646 | 0.015005 |
| HNRNPDL | -0.56161 | 0.003651 | 0.015018 |
| SLC7A6 | -0.52443 | 0.003658 | 0.015037 |
| ITGA6 | -0.73398 | 0.003657 | 0.015037 |
| GRHL2 | 0.995117 | 0.003673 | 0.015091 |
| ENSAPLG00000011298 | -0.5957 | 0.003695 | 0.015176 |
| TMEM230 | 0.550244 | 0.003702 | 0.0152 |
| ENSAPLG00000003871 | -1.23215 | 0.00371 | 0.01522 |
| CPD | -0.82136 | 0.003709 | 0.01522 |
| DPY19L3 | -0.61488 | 0.00372 | 0.015256 |
| CPSF3 | -0.48516 | 0.003726 | 0.015276 |
| ENSAPLG00000029307 | 1.078498 | 0.003768 | 0.015442 |
| CADM1 | -1.29603 | 0.003786 | 0.015511 |
| MEX3B | 0.534767 | 0.003793 | 0.015532 |
| HYAL2 | -0.76103 | 0.003796 | 0.01554 |
| ZCCHC17 | 0.626277 | 0.003806 | 0.015575 |
| PDE9A | -0.74471 | 0.003826 | 0.015651 |
| ZNF622 | -0.50015 | 0.003832 | 0.015667 |
| ENSAPLG00000027094 | -0.6508 | 0.003842 | 0.015703 |
| ENSAPLG00000009678 | 1.498971 | 0.003848 | 0.015723 |
| SNX14 | -0.50926 | 0.00385 | 0.015724 |
| XXYLT1 | 0.504387 | 0.003868 | 0.015791 |
| ELOVL6 | -0.72884 | 0.003874 | 0.015813 |
| GRHPR | -0.91548 | 0.003877 | 0.015816 |
| TGFBR2 | 0.605368 | 0.003882 | 0.015831 |
| CDC42SE2 | 0.592136 | 0.00389 | 0.015858 |
| CDCA7L | -0.48982 | 0.0039 | 0.015895 |
| SURF4 | -0.48992 | 0.003905 | 0.01591 |
| RBM24 | 0.670229 | 0.003909 | 0.015919 |
| C17orf75 | -0.75404 | 0.003913 | 0.015926 |
| RIOK3 | -0.47332 | 0.00392 | 0.015949 |
| VRK2 | -0.68178 | 0.003935 | 0.016004 |
| RTKN | 1.025237 | 0.003941 | 0.016026 |
| ITPK1 | -0.68606 | 0.003944 | 0.016031 |
| CARNMT1 | 0.496286 | 0.003946 | 0.016031 |
| UNC119 | 0.71606 | 0.003953 | 0.016057 |
| ARPC1B | -0.57916 | 0.003965 | 0.0161 |
| ZBTB33 | 0.838685 | 0.003969 | 0.016109 |
| ENSAPLG00000006877 | -0.75707 | 0.003972 | 0.016115 |
| STOM | -0.74063 | 0.003977 | 0.016123 |
| ATP9A | 1.02924 | 0.003977 | 0.016123 |
| WNK2 | 0.868012 | 0.003997 | 0.016198 |
| SOS1 | 0.768776 | 0.004004 | 0.01622 |
| FAM241A | 0.963443 | 0.004006 | 0.016222 |
| CDC37L1 | -0.63657 | 0.004014 | 0.016249 |
| BMPR2 | 0.758379 | 0.004022 | 0.016264 |
| PPP1CA | -0.56541 | 0.004022 | 0.016264 |
| LMOD1 | -0.7551 | 0.004023 | 0.016264 |
| SZRD1 | 1.245902 | 0.004024 | 0.016264 |
| ENSAPLG00000019645 | -1.01799 | 0.004036 | 0.016307 |
| GALNT2 | -1.87244 | 0.004077 | 0.016465 |
| ITM2C | 3.001709 | 0.00408 | 0.016471 |
| ENSAPLG00000000604 | 0.769444 | 0.004096 | 0.016533 |
| METAP2 | -0.47826 | 0.004125 | 0.016643 |
| IMPA1 | -0.54221 | 0.004133 | 0.01666 |
| SRM | -0.4651 | 0.004131 | 0.01666 |
| FGFR3 | 0.694702 | 0.004134 | 0.01666 |
| SLC4A2 | 0.714005 | 0.004144 | 0.016695 |
| SNX2 | -0.54526 | 0.004147 | 0.0167 |
| ENSAPLG00000008759 | -0.62697 | 0.004156 | 0.016718 |
| PARK7 | -0.56107 | 0.004156 | 0.016718 |
| BNIP1 | -0.64251 | 0.004154 | 0.016718 |
| FAM114A2 | -0.54561 | 0.004167 | 0.016754 |
| ENSAPLG00000010147 | -0.54755 | 0.004172 | 0.016768 |
| RAB40C | 0.837953 | 0.004174 | 0.016773 |
| ENSAPLG00000020237 | 4.69467 | 0.004181 | 0.016793 |
| ICE2 | 0.887117 | 0.004187 | 0.016809 |
| WASHC4 | -0.66648 | 0.004188 | 0.016809 |
| TENM4 | 4.732299 | 0.004195 | 0.01683 |
| RAB12 | 0.473228 | 0.004196 | 0.01683 |
| OXSM | -0.76079 | 0.0042 | 0.016839 |
| EFHD2 | -0.73955 | 0.004207 | 0.016859 |
| ASB3 | -0.70572 | 0.004208 | 0.01686 |
| TRAPPC12 | -0.54941 | 0.004212 | 0.016867 |
| GHSR | 4.114787 | 0.004217 | 0.01688 |
| SHQ1 | -0.53923 | 0.004226 | 0.016911 |
| ENSAPLG00000002508 | 0.939661 | 0.00423 | 0.016922 |
| PDXK | 0.646784 | 0.004243 | 0.016966 |
| GLCCI1 | 0.815557 | 0.004259 | 0.017025 |
| PLIN2 | -0.46746 | 0.004273 | 0.017076 |
| MBTPS2 | -0.81783 | 0.004276 | 0.01708 |
| ENSAPLG00000023537 | 0.566626 | 0.004286 | 0.017116 |
| PKP1 | 1.993923 | 0.00429 | 0.017123 |
| LDHA | -0.56473 | 0.004293 | 0.017128 |
| WDR11 | -0.75708 | 0.004297 | 0.017137 |
| GIN1 | -0.91747 | 0.004324 | 0.017241 |
| GNS | -0.75522 | 0.004332 | 0.017266 |
| LPCAT1 | -0.57659 | 0.004335 | 0.017271 |
| RGS4 | 2.755801 | 0.004338 | 0.017279 |
| RPL8 | -0.53627 | 0.004343 | 0.017288 |
| DCUN1D2 | -0.73305 | 0.004344 | 0.017288 |
| USP34 | 0.574723 | 0.004359 | 0.017341 |
| RCAN2 | 0.852462 | 0.004361 | 0.017344 |
| RTF1 | -0.57796 | 0.004365 | 0.017355 |
| SOD2 | -0.52126 | 0.004392 | 0.017456 |
| NONO | -0.43247 | 0.004398 | 0.017473 |
| SOSTDC1 | 4.789843 | 0.004402 | 0.017482 |
| PDLIM5 | -0.47359 | 0.004412 | 0.017511 |
| ZNF503 | 0.818881 | 0.004413 | 0.017511 |
| SDE2 | -0.58922 | 0.004415 | 0.017513 |
| HIC2 | 0.674909 | 0.004422 | 0.017536 |
| PLEK2 | 0.587854 | 0.004432 | 0.017569 |
| MREG | 0.828499 | 0.004441 | 0.017599 |
| USH2A | 2.773111 | 0.004447 | 0.017616 |
| ENSAPLG00000011659 | 0.632327 | 0.00445 | 0.017622 |
| NEK3 | -1.05819 | 0.004454 | 0.017626 |
| ENSAPLG00000022853 | 0.661739 | 0.004455 | 0.017626 |
| ENSAPLG00000008521 | -0.49083 | 0.004463 | 0.017653 |
| FAM118B | -0.50208 | 0.004468 | 0.017658 |
| YIPF4 | 0.453172 | 0.004466 | 0.017658 |
| ENSAPLG00000002443 | -0.94217 | 0.004471 | 0.017665 |
| SLC27A4 | -0.54252 | 0.004474 | 0.017672 |
| PKNOX1 | 0.499291 | 0.00449 | 0.017726 |
| APPL1 | -0.64966 | 0.004495 | 0.017735 |
| MMP16 | -0.61875 | 0.004494 | 0.017735 |
| FOXK1 | 0.592648 | 0.004519 | 0.017822 |
| CAPN10 | -0.65885 | 0.004525 | 0.01784 |
| WDR41 | -0.75859 | 0.004527 | 0.017841 |
| ADAT1 | 0.873392 | 0.004534 | 0.01786 |
| ENSAPLG00000003614 | 0.625451 | 0.004544 | 0.017883 |
| DUSP7 | 0.523664 | 0.004542 | 0.017883 |
| C3orf14 | 0.666123 | 0.004544 | 0.017883 |
| ENSAPLG00000005509 | 1.479408 | 0.004547 | 0.017887 |
| ENSAPLG00000002748 | 1.41789 | 0.004549 | 0.017889 |
| TLN1 | -0.58153 | 0.004556 | 0.017909 |
| GTF3C5 | -0.66211 | 0.004572 | 0.017967 |
| CEP19 | 0.68807 | 0.004576 | 0.017977 |
| DDX24 | -0.66063 | 0.00461 | 0.018105 |
| RPL27A | 0.551554 | 0.004618 | 0.01812 |
| YES1 | 0.57676 | 0.004618 | 0.01812 |
| NEURL1 | 0.924055 | 0.004624 | 0.018138 |
| ENSAPLG00000013325 | 0.79392 | 0.004626 | 0.018139 |
| ENSAPLG00000027780 | 0.698447 | 0.004635 | 0.018166 |
| FARSB | -0.47224 | 0.004642 | 0.01817 |
| SALL3 | 1.340981 | 0.004643 | 0.01817 |
| ENSAPLG00000013932 | -0.81439 | 0.004644 | 0.01817 |
| ENSAPLG00000021679 | 0.678451 | 0.004637 | 0.01817 |
| EIF4EBP2 | 0.951809 | 0.004641 | 0.01817 |
| DKK3 | -0.62055 | 0.004654 | 0.018203 |
| ENSAPLG00000017564 | -1.13659 | 0.004656 | 0.018203 |
| TANC1 | 0.673635 | 0.004666 | 0.018235 |
| CDR2 | 0.514114 | 0.004706 | 0.018381 |
| TXK | 4.626811 | 0.004706 | 0.018381 |
| HPSE | -3.97675 | 0.004718 | 0.018416 |
| CLDN5 | 0.601873 | 0.004718 | 0.018416 |
| GPR107 | -0.47997 | 0.004725 | 0.018433 |
| GALNT5 | 0.871832 | 0.004726 | 0.018433 |
| RABL3 | -0.60127 | 0.004729 | 0.018436 |
| ENSAPLG00000030207 | 0.693256 | 0.00474 | 0.018475 |
| FAM53B | 1.567656 | 0.004759 | 0.018542 |
| UST | 0.573864 | 0.004763 | 0.01855 |
| STRN | -0.55926 | 0.004776 | 0.018595 |
| ENSAPLG00000021321 | -0.51812 | 0.004782 | 0.01861 |
| ENSAPLG00000007746 | -0.44096 | 0.004784 | 0.018612 |
| PIGC | -0.63859 | 0.00479 | 0.018628 |
| HSF2 | -0.45291 | 0.004792 | 0.018628 |
| MTREX | -0.50944 | 0.004794 | 0.018628 |
| NPR3 | -1.35525 | 0.004795 | 0.018628 |
| IPMK | 0.626636 | 0.004809 | 0.018664 |
| DUSP4 | 0.696557 | 0.004809 | 0.018664 |
| RBM22 | -0.63538 | 0.004808 | 0.018664 |
| HDGFL3 | 0.728686 | 0.004816 | 0.018681 |
| HSPBAP1 | -0.71521 | 0.004826 | 0.018713 |
| SEC14L5 | 1.039225 | 0.004829 | 0.01872 |
| ENSAPLG00000022070 | 0.655581 | 0.004849 | 0.018792 |
| CDK5RAP2 | 0.615168 | 0.004854 | 0.018805 |
| TNRC6B | 0.756059 | 0.004865 | 0.018837 |
| ENSAPLG00000019878 | -0.66611 | 0.004879 | 0.018886 |
| CLTCL1 | -0.71313 | 0.0049 | 0.01896 |
| MT4 | 0.735518 | 0.004915 | 0.019013 |
| SRP54 | -0.45717 | 0.004923 | 0.019036 |
| ENSAPLG00000011790 | -0.49221 | 0.004928 | 0.019049 |
| ENSAPLG00000001741 | -0.5569 | 0.004935 | 0.01907 |
| ENSAPLG00000008180 | 0.618867 | 0.004941 | 0.019085 |
| KIF21A | 1.036219 | 0.004944 | 0.019092 |
| LIPT1 | -0.5749 | 0.004949 | 0.019096 |
| MATK | 1.314489 | 0.004947 | 0.019096 |
| ENSAPLG00000020795 | -0.73941 | 0.004959 | 0.019129 |
| BCL2L13 | 0.596381 | 0.00498 | 0.0192 |
| KLB | 4.602653 | 0.004983 | 0.0192 |
| ENSAPLG00000022197 | 4.603744 | 0.004982 | 0.0192 |
| ENSAPLG00000013772 | -0.63371 | 0.005012 | 0.019305 |
| CEP41 | -0.97243 | 0.005016 | 0.019314 |
| TPI1 | -0.5222 | 0.005032 | 0.019364 |
| DDX1 | -0.43892 | 0.005033 | 0.019364 |
| ITGA8 | -0.76029 | 0.005041 | 0.019388 |
| CNGA3 | 4.59686 | 0.005049 | 0.019415 |
| CCSER2 | 0.525383 | 0.005079 | 0.019521 |
| ENSAPLG00000023658 | 2.777828 | 0.005085 | 0.019539 |
| GJD4 | 4.588333 | 0.005116 | 0.01965 |
| CFAP58 | 4.594027 | 0.005119 | 0.019655 |
| MTX2 | -0.5103 | 0.005127 | 0.019671 |
| UBASH3B | 0.881306 | 0.005125 | 0.019671 |
| SLC26A2 | -1.27778 | 0.005146 | 0.019737 |
| ADAL | -0.63023 | 0.005172 | 0.019831 |
| PCARE | 4.591638 | 0.005174 | 0.019832 |
| TM9SF4 | -0.48604 | 0.00518 | 0.019846 |
| ATPAF1 | -0.7327 | 0.005196 | 0.0199 |
| MDN1 | 0.59067 | 0.005218 | 0.019978 |
| CREG1 | -0.78427 | 0.005225 | 0.01999 |
| SH3BGRL | 0.616094 | 0.005225 | 0.01999 |
| THAP1 | 1.169779 | 0.005227 | 0.019993 |
| ENSAPLG00000030815 | 1.026519 | 0.005242 | 0.020044 |
| FIGNL2 | 1.752545 | 0.005247 | 0.020053 |
| ENSAPLG00000004813 | 0.632842 | 0.00525 | 0.02006 |
| SLC9B2 | -0.90759 | 0.005252 | 0.02006 |
| PUS7 | -0.48226 | 0.005271 | 0.020124 |
| ENSAPLG00000031181 | -1.57003 | 0.005277 | 0.020141 |
| LAPTM4A | -0.52254 | 0.005282 | 0.020155 |
| HDAC1 | -0.45315 | 0.005298 | 0.020208 |
| VPS39 | -0.79133 | 0.005307 | 0.020234 |
| MCOLN3 | 0.836736 | 0.00532 | 0.020269 |
| LSS | -0.50561 | 0.005318 | 0.020269 |
| CERS1 | 1.325944 | 0.00533 | 0.020302 |
| ENSAPLG00000008971 | -0.57025 | 0.005334 | 0.02031 |
| ESRRB | 2.606526 | 0.00535 | 0.020362 |
| ACP6 | -0.64476 | 0.005364 | 0.020407 |
| CRYL1 | -1.03903 | 0.005381 | 0.020464 |
| ENSAPLG00000017182 | 0.512997 | 0.005383 | 0.020467 |
| GATA6 | 0.509716 | 0.00539 | 0.020485 |
| ENSAPLG00000007524 | 0.481117 | 0.005392 | 0.020485 |
| TMC6 | -0.9651 | 0.005395 | 0.02049 |
| PPP1R21 | -0.59474 | 0.00541 | 0.020539 |
| SPIRE1 | 0.588222 | 0.005424 | 0.020586 |
| PARG | 0.642396 | 0.005449 | 0.020676 |
| CPEB2 | 0.923522 | 0.005452 | 0.02068 |
| FRRS1 | -0.71929 | 0.005465 | 0.020719 |
| CCNY | 1.318655 | 0.005476 | 0.020755 |
| SETD6 | -0.48235 | 0.005495 | 0.020819 |
| TRPS1 | 0.547528 | 0.005509 | 0.020858 |
| ENSAPLG00000028687 | 0.758493 | 0.005508 | 0.020858 |
| ENSAPLG00000013301 | -0.55936 | 0.005519 | 0.020889 |
| XPO6 | 0.670008 | 0.00553 | 0.020924 |
| AMPD3 | -0.63033 | 0.005534 | 0.02093 |
| TBC1D4 | -0.72682 | 0.005542 | 0.020934 |
| INPP4A | 0.843816 | 0.005539 | 0.020934 |
| RIPK1 | -0.63236 | 0.005542 | 0.020934 |
| ENSAPLG00000017972 | 2.273937 | 0.005542 | 0.020934 |
| ENSAPLG00000018261 | 2.755281 | 0.005549 | 0.020951 |
| ELOVL5 | -0.4473 | 0.005553 | 0.020958 |
| RNASEH2B | -0.56218 | 0.005564 | 0.020993 |
| LZIC | 0.48969 | 0.005579 | 0.021042 |
| ENSAPLG00000015141 | 0.826148 | 0.005585 | 0.021057 |
| ENSAPLG00000030677 | -0.75365 | 0.005598 | 0.021101 |
| CASP8AP2 | 0.695666 | 0.005613 | 0.021148 |
| NYX | 2.223672 | 0.005626 | 0.021184 |
| ENSAPLG00000027307 | 0.511974 | 0.005626 | 0.021184 |
| S100Z | 3.001441 | 0.005641 | 0.021234 |
| TBC1D12 | 0.636732 | 0.005643 | 0.021234 |
| ENSAPLG00000006338 | 0.454123 | 0.005658 | 0.021275 |
| GET1 | -0.50235 | 0.005657 | 0.021275 |
| PHLDA1 | 0.8466 | 0.00566 | 0.021276 |
| CEP43 | -0.62536 | 0.005679 | 0.021339 |
| ENSAPLG00000012071 | 0.846591 | 0.005685 | 0.021346 |
| ENSAPLG00000016444 | 3.227792 | 0.005685 | 0.021346 |
| ENSAPLG00000013847 | 0.576897 | 0.005692 | 0.021367 |
| PTK7 | -0.67183 | 0.005705 | 0.021408 |
| OAZ1 | 0.618747 | 0.00571 | 0.021417 |
| ARMC9 | -0.83077 | 0.00572 | 0.021449 |
| NUP62 | -0.58304 | 0.00573 | 0.02148 |
| MLST8 | 0.467459 | 0.005739 | 0.021504 |
| GNAS | 0.55921 | 0.005751 | 0.021542 |
| TECPR2 | -0.55508 | 0.005776 | 0.021629 |
| GNG11 | 1.337778 | 0.005781 | 0.02164 |
| MAPKAPK3 | 1.073294 | 0.005792 | 0.021673 |
| JMJD1C | 0.52482 | 0.005824 | 0.021787 |
| ZBTB41 | 0.558626 | 0.005826 | 0.021787 |
| KCNC1 | 4.517428 | 0.005839 | 0.021822 |
| MLYCD | -1.29141 | 0.00584 | 0.021822 |
| EFNB2 | 0.605585 | 0.005848 | 0.021847 |
| FAM110B | -0.65952 | 0.005889 | 0.021993 |
| ARIH2 | -0.46045 | 0.005903 | 0.022035 |
| C1QTNF5 | -1.3514 | 0.005924 | 0.022108 |
| GNA13 | 0.535312 | 0.005934 | 0.022135 |
| RNF149 | -0.47341 | 0.005936 | 0.022138 |
| PIGS | -1.0047 | 0.005948 | 0.022174 |
| ITM2A | -0.47071 | 0.005956 | 0.022194 |
| NID2 | -0.95938 | 0.005957 | 0.022194 |
| SLC8A3 | 1.957357 | 0.00597 | 0.022235 |
| SESN1 | -0.56955 | 0.005981 | 0.022266 |
| CTPS2 | -0.81013 | 0.005989 | 0.022288 |
| LRRN2 | 4.740387 | 0.005995 | 0.022303 |
| ENDOV | -0.82217 | 0.006014 | 0.022367 |
| ATF2 | 0.459332 | 0.006035 | 0.022435 |
| BCL11B | 0.806708 | 0.006047 | 0.022473 |
| ENSAPLG00000014048 | -0.52707 | 0.006052 | 0.022486 |
| KLF5 | 0.477344 | 0.006063 | 0.022518 |
| ENSAPLG00000000603 | 0.592456 | 0.006071 | 0.022539 |
| FECH | -0.5717 | 0.006089 | 0.022598 |
| RBM5 | 0.462456 | 0.00612 | 0.022708 |
| BTBD7 | 0.705567 | 0.006136 | 0.022756 |
| DEGS1 | -0.48754 | 0.006143 | 0.022776 |
| ITPA | -0.67111 | 0.006151 | 0.022798 |
| C6orf120 | 0.511641 | 0.006173 | 0.02287 |
| KIFC3 | 0.683053 | 0.006178 | 0.022876 |
| TIPRL | -0.45364 | 0.006177 | 0.022876 |
| PUM1 | 0.566511 | 0.006195 | 0.022931 |
| ENSAPLG00000011608 | 0.865532 | 0.006211 | 0.022983 |
| ENSAPLG00000026440 | 0.555616 | 0.006216 | 0.02299 |
| GMNN | -0.80926 | 0.006228 | 0.023027 |
| SMPD3 | 3.469842 | 0.006236 | 0.023048 |
| HIBADH | -0.62295 | 0.006244 | 0.02307 |
| OSR2 | 1.023223 | 0.006266 | 0.023144 |
| GOLM1 | -0.78534 | 0.00629 | 0.023225 |
| PRRC1 | -0.49852 | 0.006316 | 0.023316 |
| IDE | -0.64136 | 0.006342 | 0.023404 |
| CDK19 | 0.640697 | 0.006361 | 0.023465 |
| INTS8 | -0.50053 | 0.006367 | 0.023478 |
| SGSH | -0.57474 | 0.006388 | 0.02354 |
| SACM1L | -0.50763 | 0.006386 | 0.02354 |
| RNF123 | -0.76692 | 0.006393 | 0.023551 |
| BMERB1 | 0.635398 | 0.006401 | 0.023573 |
| NPC1 | -0.66842 | 0.006404 | 0.023574 |
| YME1L1 | -0.60364 | 0.006409 | 0.023585 |
| ENSAPLG00000004797 | 0.473065 | 0.006425 | 0.023637 |
| LTBP1 | -0.6583 | 0.006431 | 0.023652 |
| DUSP19 | 0.53294 | 0.006434 | 0.023653 |
| TMCO1 | -0.54576 | 0.006456 | 0.023725 |
| EGFR | -0.52038 | 0.006495 | 0.023862 |
| ENSAPLG00000021637 | 1.050208 | 0.006499 | 0.023868 |
| RSPH1 | 2.221724 | 0.006505 | 0.023881 |
| AOPEP | 0.600316 | 0.006513 | 0.023886 |
| RIMKLB | -0.70119 | 0.00651 | 0.023886 |
| UHRF1 | -0.45693 | 0.006512 | 0.023886 |
| AAK1 | 0.698888 | 0.006529 | 0.023932 |
| MANF | -0.65587 | 0.006529 | 0.023932 |
| ENSAPLG00000010941 | 2.358655 | 0.006565 | 0.024056 |
| SYNGR3 | 2.16861 | 0.006582 | 0.024108 |
| TBL3 | -0.50117 | 0.006601 | 0.024169 |
| CCN4 | -0.66974 | 0.006622 | 0.024239 |
| MOCS2 | -0.68841 | 0.00663 | 0.024258 |
| ZFAND4 | 0.534403 | 0.006657 | 0.024343 |
| TMEM144 | -0.54947 | 0.006656 | 0.024343 |
| ZNF800 | 1.140592 | 0.006687 | 0.024445 |
| MRPL13 | 0.528352 | 0.006694 | 0.024452 |
| RUSC2 | 0.910057 | 0.006692 | 0.024452 |
| PEX6 | -1.293 | 0.006746 | 0.024633 |
| MORN2 | 1.539515 | 0.006749 | 0.024637 |
| AP4B1 | -0.60961 | 0.006764 | 0.024685 |
| RFC3 | -0.62451 | 0.006771 | 0.024701 |
| ENSAPLG00000003863 | -2.04368 | 0.006781 | 0.024729 |
| ENSAPLG00000026309 | -1.02773 | 0.006789 | 0.024752 |
| ASXL2 | 0.616856 | 0.006797 | 0.024772 |
| BTG1 | -0.53407 | 0.006817 | 0.024836 |
| CORIN | 3.620217 | 0.00682 | 0.024837 |
| PHKA2 | -0.77107 | 0.006827 | 0.024857 |
| UBXN2A | 0.550658 | 0.00684 | 0.024895 |
| NET1 | -0.50404 | 0.006853 | 0.024932 |
| ENSAPLG00000006872 | -0.5346 | 0.006861 | 0.024954 |
| SSTR3 | 1.822718 | 0.006868 | 0.024972 |
| SLC35E2B | 0.537049 | 0.006893 | 0.025046 |
| OSBPL3 | 0.583691 | 0.006893 | 0.025046 |
| ENSAPLG00000012085 | -0.46088 | 0.006918 | 0.025123 |
| EIF3L | -0.45151 | 0.006919 | 0.025123 |
| TAF5 | 0.539336 | 0.00695 | 0.025226 |
| CLCC1 | -0.74129 | 0.006955 | 0.025237 |
| ENSAPLG00000011792 | 0.46173 | 0.006988 | 0.02535 |
| SRP72 | -0.45541 | 0.006992 | 0.025356 |
| ATG4B | -0.49274 | 0.006997 | 0.025363 |
| ENSAPLG00000027201 | 3.961264 | 0.007012 | 0.025411 |
| ADPRM | -0.86137 | 0.007068 | 0.025605 |
| PANK2 | 0.544697 | 0.007073 | 0.025613 |
| MGST1 | -0.70983 | 0.007075 | 0.025613 |
| ENSAPLG00000012655 | -0.52167 | 0.007082 | 0.025629 |
| TCEA1 | -0.54398 | 0.007131 | 0.025789 |
| DGKZ | 0.916378 | 0.007129 | 0.025789 |
| TRAPPC6B | -0.64196 | 0.007134 | 0.025791 |
| IL18RAP | 2.007391 | 0.007145 | 0.025824 |
| CHN1 | 0.539943 | 0.007155 | 0.025843 |
| ASH1L | 0.542228 | 0.007153 | 0.025843 |
| LONP1 | -0.53748 | 0.00716 | 0.02585 |
| EIF1B | 0.516846 | 0.007172 | 0.025872 |
| ENSAPLG00000005550 | 0.596498 | 0.007173 | 0.025872 |
| CLSTN3 | -0.61814 | 0.00717 | 0.025872 |
| ENSAPLG00000002657 | 0.454571 | 0.00718 | 0.025891 |
| ENSAPLG00000019377 | 0.96565 | 0.007189 | 0.025913 |
| NAGLU | -0.73083 | 0.007224 | 0.026031 |
| PCSK6 | -0.90169 | 0.007248 | 0.02611 |
| ENSAPLG00000025986 | 0.843396 | 0.00726 | 0.026145 |
| ENSAPLG00000009253 | 0.72587 | 0.007265 | 0.026154 |
| ENSAPLG00000028746 | 1.369989 | 0.007274 | 0.026176 |
| ATP9B | -0.49244 | 0.007282 | 0.026196 |
| TYW3 | -0.82206 | 0.007284 | 0.026196 |
| ENSAPLG00000004283 | -0.56277 | 0.007311 | 0.026283 |
| HPRT1 | 0.492816 | 0.007349 | 0.026411 |
| SRGAP3 | 0.679042 | 0.007358 | 0.026437 |
| NUP85 | -0.48297 | 0.0074 | 0.026576 |
| ETV5 | -0.61429 | 0.007405 | 0.026585 |
| EVC2 | -0.76403 | 0.007412 | 0.026585 |
| ENSAPLG00000006235 | -0.48613 | 0.007411 | 0.026585 |
| STARD3 | 0.775756 | 0.007409 | 0.026585 |
| SAP30 | 0.587011 | 0.007421 | 0.0266 |
| SLC25A20 | 0.459388 | 0.007421 | 0.0266 |
| NFKB1 | -0.62364 | 0.007453 | 0.026705 |
| SLC44A3 | -0.74652 | 0.007466 | 0.026744 |
| S100A4 | 0.556789 | 0.007473 | 0.026754 |
| IL1RAP | -0.52155 | 0.007474 | 0.026754 |
| NOLC1 | -0.53241 | 0.007478 | 0.026761 |
| NOL11 | -0.45315 | 0.007486 | 0.02678 |
| SHMT1 | -0.96602 | 0.007493 | 0.026786 |
| MYO9B | 0.627373 | 0.00749 | 0.026786 |
| ENSAPLG00000006408 | 0.497456 | 0.00752 | 0.026865 |
| ENSAPLG00000019239 | -1.27003 | 0.007519 | 0.026865 |
| SEMA7A | 1.139929 | 0.007524 | 0.026868 |
| ENSAPLG00000017897 | 0.730286 | 0.007525 | 0.026868 |
| EYA2 | -1.21251 | 0.007543 | 0.026908 |
| SMG7 | 0.694134 | 0.007544 | 0.026908 |
| ENSAPLG00000026321 | -1.06651 | 0.00754 | 0.026908 |
| CDC5L | -0.50748 | 0.00757 | 0.026993 |
| TMEM132C | -0.77405 | 0.007591 | 0.02705 |
| ENSAPLG00000012475 | -0.64555 | 0.007589 | 0.02705 |
| TST | -0.63779 | 0.007657 | 0.027275 |
| MPPE1 | -0.61998 | 0.007662 | 0.027283 |
| RAB9B | 0.68371 | 0.007709 | 0.027444 |
| CUX1 | 0.527093 | 0.007723 | 0.027475 |
| NOL10 | -0.47587 | 0.007723 | 0.027475 |
| DLAT | -0.5118 | 0.007742 | 0.027534 |
| CYBC1 | -0.86499 | 0.007746 | 0.027538 |
| PIBF1 | -0.68308 | 0.007755 | 0.027563 |
| TRIM23 | 0.835118 | 0.00777 | 0.027604 |
| ABCD3 | -0.54486 | 0.007782 | 0.027639 |
| FAM207A | -0.59176 | 0.007787 | 0.027647 |
| SORCS2 | 4.556562 | 0.007805 | 0.027703 |
| C11orf54 | -0.64725 | 0.007817 | 0.027736 |
| REXO4 | -0.59671 | 0.00783 | 0.027775 |
| TMEM167A | 0.733286 | 0.007849 | 0.027831 |
| TAF8 | -0.54765 | 0.007863 | 0.027872 |
| MN1 | 2.276217 | 0.007906 | 0.028015 |
| KIF23 | -1.27094 | 0.007924 | 0.028072 |
| TBK1 | -0.63897 | 0.007969 | 0.028222 |
| LARP4B | 0.637661 | 0.00802 | 0.028384 |
| ENSAPLG00000017636 | -0.59389 | 0.00802 | 0.028384 |
| ENSAPLG00000022082 | -0.51528 | 0.008054 | 0.028495 |
| TTC7A | -0.65492 | 0.0081 | 0.028648 |
| ZUP1 | -0.59288 | 0.00811 | 0.028674 |
| GRIK1 | 0.814168 | 0.00814 | 0.028759 |
| ENSAPLG00000026608 | 0.807741 | 0.008138 | 0.028759 |
| ACSL4 | -0.46993 | 0.008149 | 0.028784 |
| RASL10B | 3.621084 | 0.008154 | 0.028792 |
| NRF1 | 0.713194 | 0.008183 | 0.028885 |
| NELFB | -0.43703 | 0.008198 | 0.028927 |
| ACSF3 | -1.96183 | 0.008215 | 0.02898 |
| MTFP1 | 0.83366 | 0.008264 | 0.029142 |
| JDP2 | 0.640379 | 0.008314 | 0.029309 |
| COBL | -0.7163 | 0.008321 | 0.029324 |
| BRI3 | 0.631158 | 0.008328 | 0.02934 |
| SLK | 0.503373 | 0.008335 | 0.029355 |
| SMARCD1 | -0.54326 | 0.008341 | 0.029366 |
| DCP1B | -0.5338 | 0.008347 | 0.029376 |
| GINM1 | -0.55817 | 0.008388 | 0.029513 |
| ENSAPLG00000019197 | 0.656333 | 0.008397 | 0.029535 |
| FLT4 | 1.062901 | 0.008427 | 0.029621 |
| ANKRD46 | -0.51595 | 0.008425 | 0.029621 |
| PES1 | -0.41889 | 0.008486 | 0.029819 |
| EFCAB14 | 0.583905 | 0.008502 | 0.029865 |
| CTR9 | -0.4877 | 0.00851 | 0.029885 |
| ENSAPLG00000014082 | -0.48504 | 0.008515 | 0.02989 |
| DNAJB4 | 0.410269 | 0.008529 | 0.029912 |
| ENSAPLG00000014428 | 1.908827 | 0.008524 | 0.029912 |
| ENSAPLG00000020023 | 0.810718 | 0.008528 | 0.029912 |
| SNAPC3 | -0.81008 | 0.008534 | 0.029919 |
| LYAR | -0.57615 | 0.00854 | 0.029932 |
| PIGK | -0.46551 | 0.008554 | 0.029972 |
| NCOA6 | 0.487049 | 0.008563 | 0.029993 |
| QSOX1 | -0.67991 | 0.008572 | 0.030015 |
| SFRP2 | -0.76274 | 0.008598 | 0.030095 |
| WWC1 | 0.536092 | 0.008621 | 0.030167 |
| CTHRC1 | -0.69826 | 0.008633 | 0.030198 |
| ECHDC1 | -1.6273 | 0.008637 | 0.030204 |
| GEMIN8 | -0.7255 | 0.008641 | 0.030208 |
| PDIK1L | 0.601602 | 0.008652 | 0.030235 |
| SLC39A8 | -0.89056 | 0.008667 | 0.030279 |
| GCH1 | 0.804167 | 0.008672 | 0.030286 |
| IBTK | 0.466725 | 0.00868 | 0.030305 |
| SEPTIN8 | -0.48405 | 0.008692 | 0.030336 |
| DUT | -0.81907 | 0.008702 | 0.030358 |
| PSMG4 | 0.738842 | 0.008704 | 0.030358 |
| SEPSECS | -0.64573 | 0.008716 | 0.030391 |
| MACROH2A2 | -0.58562 | 0.00872 | 0.030396 |
| FBXL2 | 0.751853 | 0.008731 | 0.030426 |
| ENSAPLG00000006105 | -0.63073 | 0.00875 | 0.03048 |
| NEDD4L | 0.614017 | 0.008752 | 0.03048 |
| ENSAPLG00000008574 | -0.74124 | 0.008782 | 0.030571 |
| ECI2 | -0.67929 | 0.008793 | 0.0306 |
| QRSL1 | -0.52497 | 0.008835 | 0.03072 |
| CBLB | 0.534622 | 0.008836 | 0.03072 |
| PTPDC1 | -0.85003 | 0.008838 | 0.03072 |
| MINDY1 | 0.600767 | 0.008838 | 0.03072 |
| NAF1 | -0.70154 | 0.008844 | 0.030731 |
| IPO13 | -0.78773 | 0.008877 | 0.030835 |
| ENSAPLG00000003922 | 0.70792 | 0.008899 | 0.03089 |
| CEP89 | -0.65822 | 0.008897 | 0.03089 |
| SNAPC1 | -0.54751 | 0.008929 | 0.030987 |
| IFT27 | -0.6127 | 0.008936 | 0.031001 |
| ENSAPLG00000004830 | 3.854353 | 0.008964 | 0.031086 |
| CACNA1C | 0.477296 | 0.008977 | 0.031123 |
| PSMB7 | -0.52529 | 0.009 | 0.031191 |
| PSTPIP2 | 2.75202 | 0.009009 | 0.031212 |
| ENSAPLG00000013508 | 0.586487 | 0.009016 | 0.031227 |
| CACNA1D | 0.71488 | 0.009025 | 0.031248 |
| GSR | 0.462972 | 0.009028 | 0.03125 |
| TRAPPC11 | -0.65196 | 0.009056 | 0.031335 |
| CCDC125 | 3.126889 | 0.009069 | 0.031342 |
| EPC1 | 0.554717 | 0.009062 | 0.031342 |
| ENSAPLG00000025146 | 0.537168 | 0.009066 | 0.031342 |
| ENSAPLG00000029386 | -0.76958 | 0.009067 | 0.031342 |
| PRKG2 | 1.557392 | 0.009092 | 0.031412 |
| SLX4 | 0.915861 | 0.009107 | 0.031452 |
| RBM45 | -0.43392 | 0.009133 | 0.031534 |
| DVL1 | 0.994122 | 0.009144 | 0.03156 |
| RSRC1 | -0.69902 | 0.009168 | 0.031632 |
| ZNF598 | 0.480384 | 0.009173 | 0.031639 |
| ENSAPLG00000025801 | -1.49687 | 0.009202 | 0.031731 |
| SCNN1G | 0.620893 | 0.009227 | 0.031806 |
| CNOT1 | 0.66036 | 0.009242 | 0.03185 |
| TRIM71 | 2.316297 | 0.009278 | 0.031964 |
| GOLT1B | 0.476286 | 0.009289 | 0.031991 |
| DNAJC6 | 1.411675 | 0.009292 | 0.031992 |
| TAF2 | 0.584591 | 0.009297 | 0.031997 |
| RPA2 | -0.49195 | 0.009301 | 0.032001 |
| ANGEL2 | -0.45594 | 0.009315 | 0.032039 |
| VDR | 0.851421 | 0.009323 | 0.032056 |
| SLC66A3 | -1.03578 | 0.00933 | 0.032072 |
| ENSAPLG00000008728 | 0.540547 | 0.009334 | 0.032075 |
| ENSAPLG00000015024 | 1.058888 | 0.009351 | 0.032122 |
| CASP8 | -0.44006 | 0.009375 | 0.032196 |
| CSE1L | -0.47607 | 0.009386 | 0.032224 |
| EIF3A | -0.55968 | 0.009434 | 0.032374 |
| ZFAND6 | 0.48977 | 0.009436 | 0.032374 |
| ENSAPLG00000020340 | -0.52759 | 0.009451 | 0.032415 |
| PEAK1 | 0.653899 | 0.009458 | 0.03243 |
| SERPINB8 | -0.54929 | 0.009469 | 0.032455 |
| JUP | -0.45267 | 0.009501 | 0.032547 |
| ENSAPLG00000023763 | 0.690926 | 0.009501 | 0.032547 |
| ENSAPLG00000027469 | 0.54067 | 0.009512 | 0.032573 |
| P2RY1 | 2.426466 | 0.00952 | 0.032589 |
| PSMC2 | -0.46482 | 0.009523 | 0.03259 |
| ENSAPLG00000018959 | 0.520557 | 0.009542 | 0.032644 |
| NSMCE4A | -0.59792 | 0.00958 | 0.032763 |
| MIEF1 | 1.029843 | 0.009594 | 0.032801 |
| DYNLT2 | 0.863919 | 0.009634 | 0.03293 |
| LIF | 0.708072 | 0.00964 | 0.03294 |
| ENSAPLG00000005093 | 2.497729 | 0.009698 | 0.033126 |
| MAD1L1 | -0.57132 | 0.009734 | 0.033229 |
| CTBS | -0.58213 | 0.009733 | 0.033229 |
| ENSAPLG00000016934 | -0.83393 | 0.009744 | 0.033253 |
| MTR | -0.89046 | 0.009784 | 0.03338 |
| WNK1 | 0.562582 | 0.009809 | 0.033454 |
| HAT1 | -0.46579 | 0.009824 | 0.033492 |
| HOXA7 | 0.784714 | 0.009839 | 0.033534 |
| ENSAPLG00000015499 | 0.527578 | 0.009857 | 0.033584 |
| C11orf49 | -0.97456 | 0.009867 | 0.033608 |
| CDC123 | -0.64618 | 0.0099 | 0.033711 |
| ENSAPLG00000009638 | 2.158497 | 0.009919 | 0.033761 |
| AP3B1 | -0.48856 | 0.009921 | 0.033761 |
| ENSAPLG00000005997 | -0.45878 | 0.009948 | 0.03384 |
| CHST3 | -0.51957 | 0.00995 | 0.03384 |
| PRKD1 | 0.472302 | 0.010055 | 0.034186 |
| PMP22 | 0.651276 | 0.01006 | 0.03419 |
| JCAD | 1.491512 | 0.010069 | 0.034212 |
| LAMA5 | 0.898054 | 0.010084 | 0.034252 |
| LAMB1 | -0.67524 | 0.010096 | 0.034281 |
| ZFYVE26 | 0.899732 | 0.01011 | 0.03432 |
| MED7 | -0.54732 | 0.01015 | 0.034444 |
| PCBD1 | 0.877804 | 0.010159 | 0.034465 |
| RBKS | 0.603896 | 0.010186 | 0.034545 |
| OTX1 | 2.779316 | 0.010226 | 0.034669 |
| KYAT3 | -0.53705 | 0.010257 | 0.034764 |
| PGAP2 | 0.568212 | 0.010291 | 0.034867 |
| TTC27 | -0.42645 | 0.010316 | 0.034941 |
| TMTC2 | -0.47904 | 0.010327 | 0.034968 |
| NDRG1 | -0.4637 | 0.010338 | 0.034994 |
| DYNC1LI1 | -0.43206 | 0.010349 | 0.03501 |
| ENSAPLG00000019153 | 1.316909 | 0.010348 | 0.03501 |
| NVL | -0.50402 | 0.01038 | 0.035103 |
| ENSAPLG00000014622 | -0.67272 | 0.010421 | 0.035231 |
| ENSAPLG00000006149 | 3.436281 | 0.01043 | 0.03525 |
| ENSAPLG00000002871 | 2.694071 | 0.010468 | 0.035362 |
| ENSAPLG00000018624 | 1.337138 | 0.010469 | 0.035362 |
| ENSAPLG00000001151 | 2.396403 | 0.010498 | 0.035448 |
| JADE3 | 0.6826 | 0.010518 | 0.035505 |
| ENSAPLG00000004420 | -0.57974 | 0.010523 | 0.035511 |
| CD81 | -0.66182 | 0.010553 | 0.035597 |
| ENSAPLG00000025387 | -0.80375 | 0.010555 | 0.035597 |
| TNFAIP8L3 | 0.645942 | 0.010581 | 0.035673 |
| NEMP1 | -0.90059 | 0.010591 | 0.035696 |
| BTBD2 | -0.94173 | 0.010605 | 0.03573 |
| GLB1L | -0.52436 | 0.010632 | 0.035811 |
| PDRG1 | 0.580546 | 0.010637 | 0.035819 |
| PTCHD4 | 3.855395 | 0.010647 | 0.035841 |
| SNX9 | -0.48161 | 0.010673 | 0.035917 |
| DOP1A | -0.52477 | 0.010681 | 0.035932 |
| RAB22A | 0.542744 | 0.010688 | 0.035946 |
| ENSAPLG00000008204 | 3.043188 | 0.010696 | 0.035961 |
| ENSAPLG00000028703 | 0.468714 | 0.01074 | 0.036098 |
| TIAL1 | -0.45844 | 0.010749 | 0.036117 |
| ENSAPLG00000020030 | 0.573422 | 0.01076 | 0.036144 |
| ENSAPLG00000006701 | -0.4851 | 0.010781 | 0.036198 |
| NUDT12 | -0.95309 | 0.010785 | 0.036198 |
| ENSAPLG00000019038 | -1.82095 | 0.01079 | 0.036198 |
| C7orf50 | -0.9428 | 0.010788 | 0.036198 |
| MTDH | -0.45339 | 0.010804 | 0.036235 |
| MGP | -0.49123 | 0.010835 | 0.036325 |
| NDUFV2 | -0.51651 | 0.010901 | 0.036536 |
| ZNF488 | 0.566624 | 0.010943 | 0.036668 |
| FGF14 | 0.739894 | 0.01097 | 0.036745 |
| ADCY9 | 0.628615 | 0.011046 | 0.036989 |
| PXDC1 | 1.242956 | 0.011072 | 0.037063 |
| ENSAPLG00000005277 | -0.90592 | 0.011075 | 0.037063 |
| DNAH17 | 0.590739 | 0.011099 | 0.037131 |
| SYT4 | 0.745063 | 0.011123 | 0.037202 |
| MAPKAPK2 | 0.720493 | 0.011127 | 0.037203 |
| TLNRD1 | 0.51224 | 0.011144 | 0.037248 |
| MAP3K14 | 0.807922 | 0.011155 | 0.037262 |
| TMEM183A | 0.471111 | 0.011155 | 0.037262 |
| THOC3 | -0.46838 | 0.01117 | 0.037301 |
| CUL5 | 0.50196 | 0.011189 | 0.037351 |
| SPPL3 | 0.467312 | 0.011228 | 0.03747 |
| PSMG3 | 0.437826 | 0.011274 | 0.037601 |
| NIPSNAP1 | -0.67454 | 0.011272 | 0.037601 |
| CNGB1 | 1.5211 | 0.011281 | 0.037615 |
| UBN1 | 0.574905 | 0.011309 | 0.037694 |
| ENSAPLG00000023268 | 0.847827 | 0.011351 | 0.037823 |
| ORC5 | -0.48433 | 0.011415 | 0.038026 |
| TAOK3 | 0.484251 | 0.011429 | 0.038059 |
| CRHBP | 2.571197 | 0.011466 | 0.038171 |
| ARRDC1 | 0.492685 | 0.011519 | 0.038337 |
| CD276 | -0.64707 | 0.011542 | 0.038403 |
| RAB39A | 0.85455 | 0.011563 | 0.038461 |
| MAPK8IP3 | 0.699172 | 0.011572 | 0.038478 |
| ARL6IP5 | 0.435315 | 0.011661 | 0.038762 |
| AGPAT5 | -0.52343 | 0.011681 | 0.038816 |
| MFAP1 | -0.54185 | 0.011693 | 0.038843 |
| ENSAPLG00000025790 | 0.571504 | 0.011712 | 0.038895 |
| ENSAPLG00000004837 | 0.747737 | 0.011744 | 0.038992 |
| ENSAPLG00000010203 | -0.60172 | 0.011827 | 0.039254 |
| EIF3D | -0.43176 | 0.011833 | 0.039262 |
| ENSAPLG00000009201 | -0.39284 | 0.011839 | 0.039271 |
| GPD1L | -0.57079 | 0.011863 | 0.039337 |
| CHAF1B | -0.65458 | 0.01187 | 0.039348 |
| MPP5 | -0.49398 | 0.011881 | 0.039373 |
| RFC2 | -0.5909 | 0.011906 | 0.039444 |
| MYO6 | -0.42242 | 0.011914 | 0.039458 |
| XRCC2 | -0.64395 | 0.011918 | 0.039459 |
| NUF2 | -0.6981 | 0.011955 | 0.03957 |
| CDCP2 | 3.243425 | 0.011965 | 0.039591 |
| ADAMTS18 | 0.957838 | 0.012056 | 0.039871 |
| ENSAPLG00000027932 | -1.93048 | 0.012057 | 0.039871 |
| ENSAPLG00000008994 | -0.64808 | 0.012081 | 0.039938 |
| ENSAPLG00000018906 | 0.473086 | 0.012105 | 0.040005 |
| CPN1 | -0.65868 | 0.01211 | 0.04001 |
| NTNG2 | 1.23247 | 0.01213 | 0.040064 |
| AP3S1 | 0.478541 | 0.01217 | 0.040185 |
| ENSAPLG00000008559 | 0.428301 | 0.01219 | 0.040231 |
| ENSAPLG00000008916 | 0.672866 | 0.012192 | 0.040231 |
| SPOPL | 0.440951 | 0.012203 | 0.040255 |
| SRPK1 | 0.430661 | 0.012221 | 0.040304 |
| ENSAPLG00000008369 | -0.43514 | 0.012254 | 0.0404 |
| TTBK2 | 0.79404 | 0.012302 | 0.040546 |
| WWTR1 | 0.886181 | 0.01231 | 0.040559 |
| PGGHG | -0.96504 | 0.012319 | 0.040579 |
| SESN2 | 1.294647 | 0.01233 | 0.040602 |
| TFG | -0.4778 | 0.012356 | 0.040675 |
| PSD | 1.181264 | 0.012362 | 0.040684 |
| CASR | 3.757896 | 0.012371 | 0.0407 |
| SRSF5 | -0.71352 | 0.012408 | 0.040808 |
| ENSAPLG00000018082 | -0.72429 | 0.012462 | 0.040976 |
| COPE | -0.43955 | 0.012474 | 0.041003 |
| ISOC1 | -0.52463 | 0.012497 | 0.041064 |
| MTCL1 | 0.529725 | 0.012516 | 0.041114 |
| CHID1 | -0.6806 | 0.012541 | 0.041184 |
| ACTN1 | -0.42933 | 0.012558 | 0.041229 |
| HSF1 | 1.022811 | 0.012587 | 0.04131 |
| RNF207 | 0.894666 | 0.012645 | 0.041489 |
| RGCC | -0.71419 | 0.012662 | 0.041531 |
| ENSAPLG00000028437 | -0.74974 | 0.012688 | 0.041604 |
| FAM160A1 | 0.578842 | 0.012696 | 0.041619 |
| ENSAPLG00000006348 | -0.70568 | 0.01271 | 0.041652 |
| ST13 | -0.40161 | 0.012732 | 0.04171 |
| MXD4 | 0.570161 | 0.012764 | 0.041804 |
| FBXO42 | 0.600444 | 0.012786 | 0.041861 |
| TPR | -0.46777 | 0.012844 | 0.042041 |
| SUPV3L1 | -0.41416 | 0.012858 | 0.042075 |
| MALRD1 | 1.058779 | 0.012898 | 0.042192 |
| MAPRE1 | -0.52807 | 0.012925 | 0.042266 |
| ENSAPLG00000026602 | 0.5259 | 0.012944 | 0.042316 |
| FAM217B | 0.530117 | 0.012955 | 0.042339 |
| CLUH | -0.44048 | 0.012984 | 0.042422 |
| RGS3 | 0.55803 | 0.012997 | 0.042452 |
| MYOD1 | 1.730273 | 0.013007 | 0.042472 |
| IRF7 | 0.770466 | 0.01304 | 0.042567 |
| ENSAPLG00000020571 | 1.266892 | 0.013048 | 0.042569 |
| ENSAPLG00000022933 | 1.802787 | 0.013049 | 0.042569 |
| ENSAPLG00000021761 | 0.934684 | 0.013065 | 0.04261 |
| CENPN | -0.94928 | 0.013072 | 0.042619 |
| ENSAPLG00000007461 | -0.59849 | 0.013089 | 0.042664 |
| IRAK4 | -0.59251 | 0.013102 | 0.042693 |
| SPATS2 | 0.459117 | 0.013128 | 0.042764 |
| ENSAPLG00000006575 | -0.61209 | 0.013148 | 0.042809 |
| AGA | -0.51882 | 0.01315 | 0.042809 |
| TMEM259 | 0.531746 | 0.013162 | 0.042837 |
| OXTR | 3.367915 | 0.013202 | 0.042953 |
| SBNO1 | 0.413566 | 0.013208 | 0.042961 |
| DCTN4 | -0.48734 | 0.013214 | 0.042968 |
| ENSAPLG00000003325 | -0.47119 | 0.013267 | 0.043123 |
| FSBP | -0.72856 | 0.01327 | 0.043123 |
| ERCC8 | 0.549864 | 0.013282 | 0.04315 |
| DNM3 | 0.99979 | 0.01329 | 0.043164 |
| KDF1 | 2.193238 | 0.013302 | 0.043177 |
| FNIP2 | -0.44648 | 0.013302 | 0.043177 |
| APMAP | -0.4441 | 0.013309 | 0.043187 |
| PDHX | -0.49278 | 0.013325 | 0.043225 |
| ENSAPLG00000018609 | -0.59774 | 0.013335 | 0.043244 |
| PLEKHA2 | 2.827645 | 0.01335 | 0.043281 |
| TMEM128 | -0.56774 | 0.013357 | 0.04329 |
| MYB | 0.475349 | 0.013363 | 0.043295 |
| DERL3 | -0.66813 | 0.013385 | 0.043356 |
| ENSAPLG00000009244 | 3.488203 | 0.013435 | 0.043503 |
| ENSAPLG00000015538 | 1.983243 | 0.013439 | 0.043503 |
| HPS5 | -0.7044 | 0.013484 | 0.043637 |
| CCNL1 | 0.519358 | 0.013547 | 0.043829 |
| SLC52A3 | -0.54395 | 0.013552 | 0.043831 |
| KLHL30 | 3.719106 | 0.013572 | 0.043881 |
| ATP6V1C1 | -0.43524 | 0.01359 | 0.043927 |
| MED9 | 0.623211 | 0.013607 | 0.043969 |
| ENSAPLG00000013548 | 0.544248 | 0.013629 | 0.044028 |
| FEN1 | -0.50838 | 0.013666 | 0.044135 |
| KAT2B | 0.478196 | 0.013679 | 0.044164 |
| LSM3 | 0.538043 | 0.013688 | 0.044177 |
| ENSAPLG00000017976 | 0.895395 | 0.013713 | 0.044247 |
| TMED3 | -0.50447 | 0.01376 | 0.044386 |
| CRADD | 0.836471 | 0.013792 | 0.044474 |
| VPS51 | -0.67676 | 0.013797 | 0.044478 |
| SNRPC | 0.687938 | 0.013865 | 0.044685 |
| ENSAPLG00000011088 | -0.72968 | 0.013989 | 0.04507 |
| MSANTD3 | -0.78526 | 0.013995 | 0.045076 |
| TMEM120A | -0.46658 | 0.014031 | 0.045178 |
| NMU | 0.48337 | 0.014058 | 0.045253 |
| ENSAPLG00000030115 | -0.50466 | 0.014129 | 0.045468 |
| SMAD7 | 0.718917 | 0.014153 | 0.045533 |
| MYH9 | -0.5905 | 0.014159 | 0.045538 |
| ENSAPLG00000010464 | 2.182404 | 0.014244 | 0.045797 |
| NR4A2 | 0.774138 | 0.014286 | 0.045918 |
| ZBTB10 | -0.46872 | 0.014302 | 0.045957 |
| PSMD14 | -0.42533 | 0.014335 | 0.046047 |
| ZW10 | -0.53077 | 0.014344 | 0.046065 |
| AVEN | -0.60916 | 0.01438 | 0.046152 |
| ZNF148 | 0.498434 | 0.014378 | 0.046152 |
| GRIN3B | 1.219564 | 0.014416 | 0.04624 |
| ABL1 | 1.257016 | 0.014413 | 0.04624 |
| IL6ST | -0.60058 | 0.014424 | 0.046253 |
| STAMBPL1 | -0.73116 | 0.014481 | 0.046422 |
| CDKAL1 | -0.47186 | 0.014516 | 0.04652 |
| STXBP3 | -0.45619 | 0.014534 | 0.046537 |
| MAN1A1 | -0.73984 | 0.014527 | 0.046537 |
| ENSAPLG00000027237 | 0.521063 | 0.014534 | 0.046537 |
| NDUFS6 | 0.626454 | 0.014585 | 0.046686 |
| VPS50 | -0.52128 | 0.014593 | 0.046699 |
| ENSAPLG00000022299 | -0.55338 | 0.014616 | 0.046757 |
| MRPS2 | 0.561065 | 0.014636 | 0.046809 |
| GPATCH3 | 0.619771 | 0.014662 | 0.046866 |
| ENSAPLG00000027519 | 0.598722 | 0.014662 | 0.046866 |
| SLC25A15 | -0.69613 | 0.0147 | 0.046968 |
| WAPL | 0.40739 | 0.014703 | 0.046968 |
| ENSAPLG00000004192 | -0.59469 | 0.014709 | 0.04697 |
| OSTM1 | -0.62675 | 0.014712 | 0.04697 |
| SIK2 | 0.753213 | 0.014719 | 0.046978 |
| ZNF711 | 0.411559 | 0.014765 | 0.047111 |
| TOR4A | 1.514839 | 0.014777 | 0.047132 |
| CSMD2 | 2.816788 | 0.014782 | 0.047132 |
| ENSAPLG00000026011 | -0.85025 | 0.014785 | 0.047132 |
| SOX6 | 1.10889 | 0.014794 | 0.047147 |
| ZBED4 | 0.562908 | 0.01481 | 0.047184 |
| STN1 | -1.36819 | 0.014834 | 0.047244 |
| BMPR1A | -0.55863 | 0.014837 | 0.047244 |
| SUN1 | -0.54388 | 0.014858 | 0.047296 |
| CYTH4 | 0.651635 | 0.014894 | 0.047398 |
| MSH3 | -0.5826 | 0.014912 | 0.04744 |
| SEC61A1 | -0.42224 | 0.015007 | 0.047729 |
| RASSF8 | 0.493366 | 0.015044 | 0.047834 |
| NFYA | -0.49838 | 0.015054 | 0.047838 |
| WDR91 | -0.44305 | 0.015054 | 0.047838 |
| DRAXIN | 2.074086 | 0.015071 | 0.047876 |
| ENSAPLG00000020498 | 0.655926 | 0.015133 | 0.048058 |
| DPY30 | 0.502086 | 0.015146 | 0.048086 |
| ENSAPLG00000004132 | -0.55667 | 0.015157 | 0.048107 |
| NOB1 | -0.44354 | 0.015185 | 0.048183 |
| CDC14A | -0.60243 | 0.0152 | 0.048216 |
| STAG2 | 0.444892 | 0.015234 | 0.048308 |
| UBN2 | 0.636536 | 0.015272 | 0.048414 |
| TRAP1 | -0.42628 | 0.015276 | 0.048414 |
| APIP | -0.48436 | 0.015296 | 0.048465 |
| ENSAPLG00000006590 | -0.44072 | 0.015312 | 0.048502 |
| ENSAPLG00000001050 | -0.69251 | 0.015347 | 0.048596 |
| DTNBP1 | -0.55416 | 0.01542 | 0.048814 |
| ENSAPLG00000002870 | -0.43973 | 0.015446 | 0.048865 |
| RAB41 | 0.683983 | 0.015449 | 0.048865 |
| MPP4 | 3.274114 | 0.015447 | 0.048865 |
| MCAT | -0.93246 | 0.015503 | 0.04902 |
| PHKA1 | 0.784205 | 0.015518 | 0.049054 |
| MLLT1 | 0.694336 | 0.01553 | 0.049078 |
| ELP2 | -0.44166 | 0.015549 | 0.049124 |
| HOMER1 | 0.578858 | 0.015567 | 0.049167 |
| DCAF7 | -0.47687 | 0.015585 | 0.049208 |
| INKA2 | -0.56667 | 0.015601 | 0.049244 |
| AKT3 | 0.579858 | 0.015634 | 0.049335 |
| SUGCT | -1.44941 | 0.015665 | 0.049403 |
| ENSAPLG00000011360 | 0.553071 | 0.015665 | 0.049403 |
| RFX3 | -0.65637 | 0.01568 | 0.049435 |
| NFATC1 | 0.621084 | 0.0157 | 0.049486 |
| DYRK1A | 0.658405 | 0.0158 | 0.049787 |
| ENSAPLG00000002087 | -0.58036 | 0.015808 | 0.049797 |
| TMEM38B | -0.8883 | 0.015827 | 0.049842 |
| ENSAPLG00000014904 | 0.591742 | 0.015845 | 0.049885 |
