# Supplementary material for: The swan genome and transcriptome, it is not all black and white
Source: Genome Biol. 2023 Jan 23;24:13. doi: 10.1186/s13059-022-02838-0 (PMC9867998; doi:10.1186/s13059-022-02838-0)
Supplement: Supplementary file 13 — Additional file 13: Supplementary Table S11. Differentially expressed cytokine or cytokine related genes in infected avian endothelial cells. [file 13059_2022_2838_MOESM13_ESM.docx]

**Supplementary Table S11: Differentially expressed cytokine or cytokine related genes in infected avian endothelial cells**

| **Cytokines** | **Black swan** | | **Chicken** | | **Duck** | |
| --- | --- | --- | --- | --- | --- | --- |
| **Chemokine** | **Up**  **regulated** | **Down**  **regulated** | **Up**  **regulated** | **Down**  **regulated** | **Up**  **regulated** | **Down**  **regulated** |
| CC subfamily | *CCL20, CXCL1,* |  | *CCL4, CCL4L1, CCL4L2, CCL17, CCR5, CCL20* |  | *CCR5* |  |
| CXC subfamily | *CXCL8, CXCR5,* | *CXCR4* | *CXCL8, CXCL12, CXCL14, CX3CL1* | *CXCR2, CXCR4, CXCR7* | *CXCL8, CXCL12,* |  |
| **The class I helical cytokines** |  |  |  |  |  |  |
| ɤ-chain-utilizing | *IL21R* |  | *IL7, IL7R, IL9R, IL15* |  |  |  |
| IL4-like | *CSF2RA* |  | *CSF2RB, CSF2RB, CSF2,* | *IL2RB* |  | *IL13RA1* |
| Prolactin family |  |  | *GHR* |  | *GHR* |  |
| IL6/12-like | *IL6, IL11RA, IL12RB2, LIF* |  | *IL6, IL11RA, IL12, IL35, CLCF1, OSMR* | *IL6ST, IL12RB1* | *LIF* | *IL6ST* |
| **The class II helical cytokines** |  |  |  |  |  |  |
| IL10/28-like |  |  | *IL10RA, IL10RB, IL20, IL20RA, IL28A, IL28B* |  |  |  |
| Interferon family | *IFNB1* |  | *IFNAR2, IFNGR2* | *IFNAR1* |  |  |
| **IL1-like cytokines** | *IL18R2, ST2* |  | *IL1R1, IL1RAP, IL1R2, IL1RL2, IL18, IL18R1, IL18RAP* |  | *IL1R1, IL1R2, IL18RAP* |  |
| **IL17-like** |  |  | *IL17RA* |  |  |  |
| **Non-classified** | *IL16* |  | *IL34* | *IL16, CSFR1* |  |  |
| **TNF family** | *DR6, EDAR, CD30L, GITRL, BAFF, (TNSF8, TNFAIP2, TNSF13B, TNFSF21)* | *CD40* | *VEGI, DR4, DR5, DR6, EDA, CD30L, BAFF* | *TNFR1, DCR3, FAS, XEDAR, RANK, RANKL, OPG, CD40, BAFFR, TROY* | *DR6, CD30L, BAFF* | *TNFR1, TRAIL, NGF* |
| **TGF-β family** | *ACVR2A, BMPR2, BPMR1B* |  | *TGFB1, TGFB2, BMP10, ACVR2A, INHBB, AMH AMHR2, BMPR1B, ACVR1, BMP8,* | *TGFBR1, TGFBR2,*  *BMPR1A, GDF9, BMP15* | *TGFBR2, GDF2, BMPR2, INHBB, NODAL, BMP15* | *TGFBR1, TGFB3, BMPR1A, BMPR1B* |
