# Supplementary material for: The swan genome and transcriptome, it is not all black and white
Source: Genome Biol. 2023 Jan 23;24:13. doi: 10.1186/s13059-022-02838-0 (PMC9867998; doi:10.1186/s13059-022-02838-0)
Supplement: Supplementary file 14 — Additional file 14: Supplementary Table S12. 113 GO terms were significantly enriched in infected black swan endothelial cells. [file 13059_2022_2838_MOESM14_ESM.docx]

**Supplementary Table S12: 113 GO terms were significantly enriched in infected black swan endothelial cells**

| **Term** | **Weight** |
| --- | --- |
| cleavage involved in rRNA processing | 0.000057 |
| defense response to virus | 0.000085 |
| positive regulation of transforming | 0.00012 |
| ribonucleoprotein complex assembly | 0.00019 |
| snRNA 3'-end processing | 0.00019 |
| maturation of 5.8S rRNA from | 0.00047 |
| Somitogenesis | 0.00077 |
| positive regulation of histone | 0.00088 |
| lens morphogenesis in camera-type eye | 0.00184 |
| centrosome localization | 0.00194 |
| negative regulation of MAP kinase | 0.00221 |
| ribosome assembly | 0.00245 |
| peptide hormone secretion | 0.00281 |
| maternal process involved in female | 0.00301 |
| positive regulation of cell cycle | 0.00346 |
| positive regulation of phosphorylation | 0.00353 |
| tRNA metabolic process | 0.0036 |
| positive regulation of cytoskeleton | 0.00373 |
| regulation of G2/M transition of | 0.0042 |
| social behavior | 0.00567 |
| nuclear-transcribed mRNA catabolic | 0.00605 |
| intraciliary transport | 0.00607 |
| negative regulation of intrinsic | 0.00607 |
| regulation of cytosolic calcium ion | 0.00618 |
| ribosomal large subunit biogenesis | 0.00747 |
| negative regulation of transcription by | 0.00748 |
| cytoplasmic microtubule organization | 0.00924 |
| RNA phosphodiester bond hydrolysis | 0.00978 |
| spleen development | 0.00978 |
| positive regulation of telomere | 0.00978 |
| regulation of cell population | 0.00991 |
| regulation of cell cycle | 0.01028 |
| ciliary basal body-plasma membrane | 0.01068 |
| snRNA transcription by RNA polymerase II | 0.01105 |
| negative regulation of JNK cascade | 0.01105 |
| positive regulation of cellular protein | 0.01125 |
| regulation of cellular response to heat | 0.0114 |
| positive regulation of cell population | 0.01183 |
| metaphase plate congression | 0.01233 |
| ganglioside metabolic process | 0.01239 |
| RNA 5'-end processing | 0.01239 |
| modulation by host of viral process | 0.01239 |
| positive regulation of protein | 0.01239 |
| protein localization to chromosome | 0.01239 |
| establishment of protein localization | 0.01239 |
| nuclear-transcribed mRNA catabolic | 0.01239 |
| ceramide catabolic process | 0.01239 |
| regulation of necroptotic process | 0.01239 |
| peptidyl-threonine dephosphorylation | 0.01239 |
| anion transmembrane transport | 0.01257 |
| membrane docking | 0.01382 |
| megakaryocyte differentiation | 0.01456 |
| regulation of JUN kinase activity | 0.01456 |
| positive regulation of establishment of | 0.01485 |
| negative regulation of microtubule | 0.01487 |
| regulation of cardiac muscle cell | 0.01487 |
| negative regulation of cellular | 0.01565 |
| protein folding | 0.01568 |
| nitrogen compound transport | 0.0157 |
| positive regulation of mitotic cell | 0.01571 |
| negative regulation of small molecule | 0.01756 |
| positive regulation of cell growth | 0.0197 |
| RNA localization | 0.01973 |
| cellular response to gamma radiation | 0.01979 |
| developmental induction | 0.01979 |
| extrinsic apoptotic signaling pathway | 0.01987 |
| focal adhesion assembly | 0.01998 |
| maintenance of location in cell | 0.0201 |
| regulation of translational initiation | 0.02123 |
| neuron maturation | 0.02147 |
| positive regulation of stress fiber | 0.02147 |
| positive regulation of interleukin-2 | 0.02152 |
| positive regulation of microtubule | 0.02152 |
| negative regulation of ERK1 and ERK2 | 0.02203 |
| RNA processing | 0.02409 |
| histone deacetylation | 0.02434 |
| regulation of viral genome replication | 0.02485 |
| positive regulation of cell cycle G2/M | 0.025 |
| positive regulation of lipase activity | 0.0251 |
| positive regulation of receptor | 0.02513 |
| DNA damage response signal | 0.02545 |
| modification-dependent macromolecule | 0.02637 |
| DNA damage checkpoint | 0.02928 |
| regulation of multicellular organismal | 0.02931 |
| regulation of transcription from RNA | 0.02935 |
| zymogen activation | 0.02954 |
| embryonic camera-type eye morphogenesis | 0.02959 |
| positive regulation of cell cycle | 0.02963 |
| actin filament-based movement | 0.02968 |
| cellular response to interferon-gamma | 0.02983 |
| maturation of SSU-rRNA from | 0.02993 |
| regulation of organ growth | 0.03064 |
| CD4-positive alpha-beta T cell | 0.03091 |
| rRNA processing | 0.03466 |
| locomotory behavior | 0.03578 |
| transcription, DNA-templated | 0.03752 |
| spermatid development | 0.03783 |
| negative regulation of catalytic | 0.03904 |
| multi-multicellular organism process | 0.03998 |
| protein exit from endoplasmic reticulum | 0.04023 |
| transcription-coupled nucleotide-excisio | 0.04023 |
| oocyte development | 0.04042 |
| response to interleukin-1 | 0.04042 |
| protein localization to microtubule | 0.04046 |
| regulation of intracellular steroid | 0.04049 |
| neurogenesis | 0.04146 |
| thymus development | 0.04196 |
| protein stabilization | 0.04276 |
| developmental growth | 0.0465 |
| adult behavior | 0.04788 |
| carbohydrate metabolic process | 0.04809 |
| cell population proliferation | 0.04815 |
| positive regulation of translation | 0.04881 |
