# Supplementary material for: The swan genome and transcriptome, it is not all black and white
Source: Genome Biol. 2023 Jan 23;24:13. doi: 10.1186/s13059-022-02838-0 (PMC9867998; doi:10.1186/s13059-022-02838-0)
Supplement: Supplementary file 15 — Additional file 15: Supplementary Table S13. 123 GO terms were significantly enriched in infected chicken endothelial cells. [file 13059_2022_2838_MOESM15_ESM.docx]

**Supplementary Table S13: 123 GO terms were significantly enriched in infected chicken endothelial cells**

| **Term** | **Weight** |
| --- | --- |
| mitochondrial RNA metabolic process | 0.0001 |
| tRNA aminoacylation for protein | 0.00026 |
| regulation of protein tyrosine kinase | 0.00033 |
| Translation | 0.00062 |
| ribosomal large subunit biogenesis | 0.00076 |
| negative regulation of intrinsic | 0.00114 |
| regulation of cyclin-dependent protein | 0.00118 |
| RNA methylation | 0.00308 |
| protein targeting | 0.00322 |
| cytosolic calcium ion transport | 0.00329 |
| negative regulation of apoptotic | 0.00347 |
| mitochondrial gene expression | 0.00386 |
| cytoplasmic translation | 0.00444 |
| early endosome to late endosome | 0.00454 |
| intracellular protein transport | 0.00515 |
| establishment of protein localization | 0.00525 |
| cytoplasmic pattern recognition | 0.00528 |
| intrinsic apoptotic signaling pathway | 0.00528 |
| Golgi to plasma membrane transport | 0.00528 |
| establishment of cell polarity | 0.00536 |
| cellular response to UV | 0.0058 |
| regulation of stem cell differentiation | 0.00581 |
| protein ubiquitination | 0.00614 |
| negative regulation of leukocyte | 0.00635 |
| regulation of epidermal growth factor | 0.00635 |
| positive regulation of viral process | 0.00644 |
| regulation of phosphatidylinositol 3-kin | 0.00645 |
| mRNA polyadenylation | 0.00698 |
| axonal transport | 0.00698 |
| RNA phosphodiester bond hydrolysis | 0.00737 |
| ER-nucleus signaling pathway | 0.00791 |
| translational elongation | 0.00791 |
| myeloid leukocyte migration | 0.00841 |
| alpha-amino acid biosynthetic process | 0.00852 |
| aerobic respiration | 0.00852 |
| rRNA processing | 0.00869 |
| lysosomal transport | 0.00881 |
| dicarboxylic acid metabolic process | 0.00901 |
| regulation of leukocyte chemotaxis | 0.01066 |
| regulation of osteoclast differentiation | 0.01241 |
| positive regulation of innate immune | 0.01255 |
| endoplasmic reticulum unfolded protein | 0.01255 |
| regulation of synaptic plasticity | 0.01257 |
| negative regulation of response to DNA | 0.01271 |
| cellular response to hypoxia | 0.01383 |
| positive regulation of protein | 0.01508 |
| sulfur compound metabolic process | 0.0155 |
| substrate adhesion-dependent cell | 0.01626 |
| mitochondrial membrane organization | 0.01677 |
| ncRNA processing | 0.01692 |
| mitochondrial transport | 0.01718 |
| localization within membrane | 0.01735 |
| mononuclear cell migration | 0.01737 |
| chaperone-mediated protein folding | 0.01737 |
| spliceosomal snRNP assembly | 0.01737 |
| positive regulation of cellular protein | 0.01742 |
| cellular response to virus | 0.01779 |
| interaction with symbiont | 0.01813 |
| mRNA catabolic process | 0.01815 |
| chromatin organization | 0.01826 |
| autophagosome assembly | 0.01826 |
| microtubule organizing center | 0.01829 |
| apoptotic process | 0.01959 |
| mRNA splicing | 0.02116 |
| regulation of protein dephosphorylation | 0.02172 |
| microtubule cytoskeleton organization | 0.02176 |
| positive regulation of translation | 0.02329 |
| telomere maintenance | 0.02394 |
| regulation of GTPase activity | 0.02509 |
| positive regulation of binding | 0.02523 |
| osteoclast differentiation | 0.02523 |
| regulation of mRNA stability | 0.0254 |
| natural killer cell activation | 0.0254 |
| regulation of extracellular matrix | 0.0254 |
| cholesterol biosynthetic process | 0.0254 |
| protein import | 0.02546 |
| spindle assembly | 0.02565 |
| positive regulation of protein | 0.02566 |
| long-chain fatty acid metabolic process | 0.02566 |
| positive regulation of proteasomal | 0.02724 |
| protein localization to cell periphery | 0.028 |
| ubiquitin-dependent ERAD pathway | 0.02896 |
| cell population proliferation | 0.02948 |
| peptidyl-amino acid modification | 0.02987 |
| protein N-linked glycosylation | 0.03069 |
| protein modification by small protein | 0.03085 |
| positive regulation of signal | 0.03171 |
| regulation of cell cycle | 0.03192 |
| cell migration involved in sprouting | 0.03224 |
| organelle transport along microtubule | 0.03239 |
| RNA 3'-end processing | 0.03348 |
| protein methylation | 0.03397 |
| DNA packaging | 0.03409 |
| camera-type eye morphogenesis | 0.0342 |
| cellular response to abiotic stimulus | 0.03452 |
| leukocyte mediated cytotoxicity | 0.03538 |
| endothelial cell development | 0.03538 |
| purine ribonucleoside metabolic process | 0.03538 |
| regulation of blood pressure | 0.03607 |
| positive regulation of lymphocyte | 0.0363 |
| regulation of cell adhesion | 0.03636 |
| regulation of lymphocyte migration | 0.0364 |
| L-alpha-amino acid transmembrane | 0.0364 |
| regulation of double-strand break | 0.0364 |
| cellular iron ion homeostasis | 0.0364 |
| regulation of DNA binding | 0.03646 |
| BMP signaling pathway | 0.03649 |
| cellular response to external stimulus | 0.0365 |
| regulation of organelle assembly | 0.03762 |
| cation transmembrane transport | 0.03809 |
| positive regulation of intracellular traunsduction | 0.04161 |
| carboxylic acid catabolic process | 0.04558 |
| regulation of oxidative stress-induced | 0.04589 |
| ribosome assembly | 0.04634 |
| cellular response to interleukin-1 | 0.04634 |
| mitochondrial translation | 0.04634 |
| regulation of vesicle-mediated transport | 0.04672 |
| peptide metabolic process | 0.04757 |
| regulation of G1/S transition of | 0.04857 |
| positive regulation of Notch signaling | 0.04888 |
| establishment of spindle localization | 0.04888 |
| cellular calcium ion homeostasis | 0.04906 |
| negative regulation of translation | 0.04975 |
