# Supplementary material for: The swan genome and transcriptome, it is not all black and white
Source: Genome Biol. 2023 Jan 23;24:13. doi: 10.1186/s13059-022-02838-0 (PMC9867998; doi:10.1186/s13059-022-02838-0)
Supplement: Supplementary file 16 — Additional file 16: Supplementary Table S14. 150 GO terms were significantly enriched in infected duck endothelial cells. [file 13059_2022_2838_MOESM16_ESM.docx]

**Supplementary Table S14: 150 GO terms were significantly enriched in infected duck endothelial cells**

| **Term** | **Weight** |
| --- | --- |
| endodermal cell differentiation | 0.00046 |
| Angiogenesis | 0.00048 |
| peptidyl-proline hydroxylation | 0.00087 |
| trabecula formation | 0.00087 |
| inactivation of MAPK activity | 0.00087 |
| negative regulation of cell population p... | 0.00093 |
| cell-substrate adhesion | 0.00179 |
| response to hypoxia | 0.00199 |
| IMP metabolic process | 0.00229 |
| serine family amino acid biosynthetic pr... | 0.00229 |
| sphingomyelin metabolic process | 0.00229 |
| dicarboxylic acid metabolic process | 0.0023 |
| tricarboxylic acid cycle | 0.00246 |
| lymphocyte proliferation | 0.00312 |
| negative regulation of transcription by ... | 0.00331 |
| in utero embryonic development | 0.00352 |
| nitrogen compound metabolic process | 0.00377 |
| negative regulation of canonical Wnt sig... | 0.00427 |
| thymus development | 0.00445 |
| protein homotetramerization | 0.00445 |
| negative regulation of apoptotic process | 0.00456 |
| regulation of osteoblast proliferation | 0.005 |
| positive regulation of phospholipid meta... | 0.00504 |
| tRNA aminoacylation for protein translat... | 0.00511 |
| purine ribonucleoside monophosphate bios... | 0.00548 |
| endoplasmic reticulum calcium ion homeos... | 0.00548 |
| regulation of ERK1 and ERK2 cascade | 0.00579 |
| cerebral cortex radial glia guided migra... | 0.00587 |
| regulation of stem cell proliferation | 0.00588 |
| mitochondrial transmembrane transport | 0.00679 |
| skeletal system morphogenesis | 0.00698 |
| regulation of blood pressure | 0.0071 |
| skin development | 0.00791 |
| proteolysis involved in cellular protein... | 0.00801 |
| retrograde protein transport | 0.00802 |
| response to toxic substance | 0.00818 |
| long-chain fatty acid metabolic process | 0.00914 |
| neuron apoptotic process | 0.00919 |
| myelination | 0.00944 |
| positive regulation of DNA-binding trans... | 0.00975 |
| cardiac chamber morphogenesis | 0.00982 |
| regulation of T cell proliferation | 0.00991 |
| meiotic cell cycle process | 0.00994 |
| outflow tract septum morphogenesis | 0.01051 |
| heart trabecula morphogenesis | 0.01051 |
| post-embryonic development | 0.01091 |
| glycolytic process | 0.01136 |
| cell fate commitment involved in formati... | 0.01186 |
| negative regulation of striated muscle c... | 0.01186 |
| rhythmic process | 0.01193 |
| cartilage development | 0.01246 |
| endosomal transport | 0.01285 |
| collagen metabolic process | 0.01313 |
| nuclear envelope organization | 0.01318 |
| regulation of cardiac muscle cell differ... | 0.01318 |
| regulation of keratinocyte differentiati... | 0.01341 |
| intracellular receptor signaling pathway | 0.01346 |
| response to unfolded protein | 0.01371 |
| dendritic spine organization | 0.0138 |
| mesenchyme development | 0.01403 |
| Golgi localization | 0.01427 |
| histone H3-K36 methylation | 0.01427 |
| methionine metabolic process | 0.01427 |
| positive regulation of carbohydrate meta... | 0.01432 |
| mRNA catabolic process | 0.01473 |
| negative regulation of gene expression | 0.01476 |
| aromatic compound catabolic process | 0.01517 |
| myoblast differentiation | 0.01565 |
| negative regulation of ERK1 and ERK2 cas... | 0.0158 |
| cellular response to lipid | 0.01587 |
| negative regulation of gene expression | 0.01706 |
| negative regulation of cell differentiat... | 0.01789 |
| positive regulation of transforming grow... | 0.01837 |
| positive regulation of developmental gro... | 0.01871 |
| carboxylic acid metabolic process | 0.01876 |
| oxidation-reduction process | 0.02009 |
| cellular response to hypoxia | 0.02025 |
| epithelial cell proliferation | 0.02088 |
| regulation of protein stability | 0.02136 |
| negative regulation of cyclin-dependent ... | 0.02142 |
| sphingolipid catabolic process | 0.02142 |
| negative regulation of small molecule me... | 0.02268 |
| aorta morphogenesis | 0.02486 |
| one-carbon metabolic process | 0.02486 |
| regulation of calcium ion import | 0.02486 |
| protein N-linked glycosylation via aspar... | 0.02486 |
| extracellular matrix organization | 0.02508 |
| negative regulation of neurogenesis | 0.0252 |
| negative regulation of cellular metaboli... | 0.02534 |
| fatty acid beta-oxidation | 0.0268 |
| regulation of growth | 0.02776 |
| somite development | 0.02839 |
| peptide catabolic process | 0.02862 |
| sulfur amino acid biosynthetic process | 0.02862 |
| regulation of phospholipid biosynthetic ... | 0.02862 |
| interleukin-1-mediated signaling pathway | 0.02862 |
| folic acid-containing compound metabolic... | 0.02862 |
| positive regulation of glial cell differ... | 0.02862 |
| apoptotic process involved in morphogene... | 0.02862 |
| carboxylic acid biosynthetic process | 0.02902 |
| carbohydrate derivative catabolic proces... | 0.02941 |
| branching involved in blood vessel morph... | 0.02982 |
| embryonic hindlimb morphogenesis | 0.02982 |
| regulation of SMAD protein signal transd... | 0.02982 |
| regulation of hematopoietic progenitor c... | 0.02982 |
| positive regulation of transcription by ... | 0.03033 |
| epithelium development | 0.03049 |
| positive regulation of cell population p... | 0.03067 |
| response to endoplasmic reticulum stress | 0.03099 |
| protein localization to organelle | 0.03101 |
| negative regulation of cell growth | 0.03135 |
| embryonic eye morphogenesis | 0.0314 |
| regulation of transcription by RNA polym... | 0.03143 |
| establishment of protein localization to... | 0.03146 |
| regulation of interleukin-1 production | 0.03146 |
| organic anion transport | 0.03149 |
| regulation of immune response | 0.03215 |
| cellular response to fibroblast growth f... | 0.03231 |
| ER-nucleus signaling pathway | 0.03262 |
| actin nucleation | 0.03277 |
| tissue homeostasis | 0.03492 |
| antibiotic metabolic process | 0.03534 |
| branching involved in ureteric bud morph... | 0.03534 |
| positive regulation of T cell differenti... | 0.03536 |
| chondrocyte development | 0.03546 |
| sulfur amino acid metabolic process | 0.03551 |
| membrane protein proteolysis | 0.03569 |
| cardiac septum development | 0.03573 |
| extrinsic apoptotic signaling pathway in... | 0.03579 |
| regulation of phosphatidylinositol 3-kin... | 0.03583 |
| regulation of protein deacetylation | 0.03588 |
| prostate gland development | 0.03592 |
| regulation of histone acetylation | 0.03602 |
| myeloid cell differentiation | 0.03665 |
| sensory perception of mechanical stimulu... | 0.03685 |
| apoptotic mitochondrial changes | 0.03691 |
| cellular response to unfolded protein | 0.03692 |
| negative regulation of secretion | 0.03699 |
| postsynapse organization | 0.03701 |
| female pregnancy | 0.03707 |
| positive regulation of vasculature devel... | 0.03707 |
| cellular response to steroid hormone sti... | 0.03708 |
| synaptic vesicle recycling | 0.03709 |
| ceramide biosynthetic process | 0.03711 |
| regulation of interleukin-10 production | 0.03713 |
| regulation of G2/M transition of mitotic... | 0.03716 |
| peptidyl-lysine modification | 0.03745 |
| face morphogenesis | 0.03797 |
| protein stabilization | 0.03818 |
| cellular response to insulin stimulus | 0.03867 |
