# Supplementary material for: The swan genome and transcriptome, it is not all black and white
Source: Genome Biol. 2023 Jan 23;24:13. doi: 10.1186/s13059-022-02838-0 (PMC9867998; doi:10.1186/s13059-022-02838-0)
Supplement: Supplementary file 17 — Additional file 17: Supplementary Table S15. Dual-specificity phosphatases in black swan, chicken and duck endothelial cells infected with VN04*. [file 13059_2022_2838_MOESM17_ESM.docx]

**Supplementary Table S15: Dual-specificity phosphatases in black swan, chicken and duck endothelial cells infected with VN04***

| **Gene** | **Black Swan** | **Chicken** | **Duck** |
| --- | --- | --- | --- |
| *DUSP1* |  |  |  |
| *DUSP4* |  | NE |  |
| DUSP5 |  |  |  |
| DUSP6 | NE |  | NE |
| DUSP7 |  |  |  |
| DUSP8 | NE | NE |  |
| DUSP10 |  |  |  |
| DUSP12 | NE |  |  |
| DUSP15 | NE |  | NE |
| DUSP16 | NE | NE |  |
| DUSP19 | NE |  | NE |
| DUSP23 | NE |  | NE |
| RGS3 |  | NE |  |
| SPRED1 | NE | NE |  |

* Green indicates significantly upregulated genes; red indicates significantly down regulated genes; NE = not differentially expression (adjusted p value >0.05) or not expressed
