# Supplementary material for: The swan genome and transcriptome, it is not all black and white
Source: Genome Biol. 2023 Jan 23;24:13. doi: 10.1186/s13059-022-02838-0 (PMC9867998; doi:10.1186/s13059-022-02838-0)
Supplement: Supplementary file 18 — Additional file 18: Supplementary Table S16. Parameters used for the FALCON run. Length cut-off was detected automatically for seed-read length. [file 13059_2022_2838_MOESM18_ESM.docx]

**Supplementary Table S16: Parameters used for the FALCON run.** Length cut-off was detected automatically for seed-read length

| **Parameter** | **Value** |
| --- | --- |
| Length cut-off | -1 |
| Length cut-off pre-reads | 5000 |
| Target | Assembly |
| Genome size | 1,4181 x 10^9 |
| Seed-coverage | 30 |
