# Supplementary material for: The swan genome and transcriptome, it is not all black and white
Source: Genome Biol. 2023 Jan 23;24:13. doi: 10.1186/s13059-022-02838-0 (PMC9867998; doi:10.1186/s13059-022-02838-0)
Supplement: Supplementary file 19 — Additional file 19: Review history. [file 13059_2022_2838_MOESM19_ESM.docx]

Review History

**First round of review**

**Reviewer 1**

**Were you able to assess all statistics in the manuscript, including the appropriateness of statistical tests used?**

Yes, and I have assessed the statistics in my report.

**Comments to author:**

The authors presented the genome assemblies and data analysis on the black and mute genomes. The assembly procedures followed the recommendations from VGP and ISO-seq data was used to help gene annotation. The authors also discussed immune repertoire and susceptibility to infectious disease, such as highly pathogenic avian influenza (HPAI), which is very interesting. Overall, the assemblies and the analysis work provide valuable resources for the genomic communities.

I have a few issues/suggestions, mainly on assembly QC and would like to share with the authors:

1. Base accuracy QV

Please run Merqury to get QV using 10X reads:

https://genomebiology.biomedcentral.com/articles/10.1186/s13059-020-02134-9

https://github.com/marbl/merqury

Please report the QV and also the completeness values

2. Difference of gene numbers between the black and mute swans

There seems to be a big difference of annotated genes between the Black (15478) and Mute (14791) swans as outlined in Table 1. I also looked at the gene number in the goose genome which is 16150, see Lu, L. et al. 2015 "The goose genome sequence leads to insights into the evolution of waterfowl and susceptibility to fatty liver", Genome Biology.

I noticed that the quality of the mute swan is not as good as the black swan, judging from scaffold N50. Ideally I would also like to see the contig N50. For assemblies from PacBio CLR reads, gene annotation could be affected if base polishing is not properly done.

The authors may need to explain the gene difference between the two swans. If it is due to assembly quality, it should be mentioned in the manuscript. Or this is due to difference in swan genetics.

3 Hi-C plots

Please provide plots of Hi-C maps for the curated black and mute assemblies.

4. Heterozygosity

Please run GenomeScope 2.0 to report heterozygosity using 10x reads:

https://github.com/tbenavi1/genomescope2.0.

**Reviewer 2**

**Were you able to assess all statistics in the manuscript, including the appropriateness of statistical tests used?**

Yes, and I have assessed the statistics in my report.

**Comments to author:**

Karawita and colleagues describe the newly assembled genome of the black (Cygnus atratus) and mute (Cygnus olor) swan and explore the genomic underpinnings of differences in plumage coloration and susceptibility to highly pathogenic avian influenza (HPAI). The study employs cutting-edge techniques to produce a high-quality near chromosome length genome and, in addition to using genomic information to infer functional changes, performs experimental validation for some of their findings. The study is overall well written and provides interesting new insights into the genetics of immune response to HPAI, being of interest to a large audience.

Some findings that I find particularly interesting are the hypothesis that the common ancestor of current swans could have been black (as opposed to the most common white form), and the absence of expression of TLR7 in infected cells of black swan (which is evidence that this gene could underlie the poor response against HPAI virus). In both of these cases (and at many segments of the manuscript), however, the study seems to take a candidate gene approach without giving a clear explanation for why certain genes are being particularly explored. For example, why choosing four candidate genes for plumage? Several other genes are known to be implicated in plumage evolution in birds, even when we consider only chicken (e.g., MC1R; ASIP; HERC2; TYR etc; Guo et al. 2010; Estalles C et al. 2022). It is not clear why these four were chosen to be investigated. Similarly, although TLR7 is clearly an important gene in the immune response against HPAI virus, many other immune genes (including other TLR genes) are as well. Chen at all 2013 presents an extensive list of genes involved in recognition of viruses in birds. Why has the focus been solely on TLR7? I don't doubt these genes could indeed be implicated in the phenotypes investigated (as the evidence exists), but some of these findings would be more convincing if enough context had been given for their choice or if the manuscript made a better job at rejecting other candidates.

Other minor comments:

L117-118. The acronym VGP is used multiple times throughout the manuscript but never defined. It could be defined here.

L123-124. Why the discrepancy between the number of assembled chromosomes and expected diploid number?

L148-149. "Structural genomic differences have been associated with the differential susceptibility of chickens and ducks to avian influenza". So why investigate only inversions? The rationale here should be clarified.

L152-153. Were the detected inverted regions only present in chromosome 1? It would be nice to see a figure or table with that information.

L155-158. "However, given the absence of substantive structural variants between the black and mute swan, it is likely that any immunological consequences of these structural variants would be present in multiple swan species." - if that's the case, why to look for inversions in duck in the first place?

L189. Although assessing "whether isolation of the black swan resulted in changes in immune gene repertoire" would be very interesting, this is not actually what is being investigated here, since no alternative hypothesis is being assessed. What is being actually tested is whether the black swan (which happens to be isolated in Australia) has a different immune gene repertoire when compared to its close relatives. Any statement about the reasons for these differences can only be hypothesized.

L284. I would argue this is not a striking finding. It is quite common that these higher-level GO terms, embracing a variety of other terms, end up as most significant. It is an inherent problem of these enrichment analysis.

L454. Scaffolds. "s" is missing.

Table 1. A different symbol for micro and macro-chromosomes (e.g., † §) was probably meant here, since "*" is already assigned to ISO-seq coverage.

Figure 1. I am not a huge fan of the design of this figure. Is the amino acid sequence for black swan important to be shown? Maybe having all four species consistently shown as blue bar would make it more intelligible.

Figure 2. Numbers at the tip of the tree are poorly aligned.

Figure 3. Extra "a" before "(" at the figure title on the left.

Figure 5. C "Biological process" on top is inconsistent with A and B.

Suppl. The order of supplemental figures and tables do not follow the manuscript order neither are sorted in a logic way.

**Authors Response**

**Point-by-point responses to the reviewers’ comments:**

Reviewer #1

1. Please run Merqury to get QV using 10X reads: Please report the QV and also the completeness values.

*Reply: 10X library preparations have been generated and sequenced for the mute swan (https://vgp.github.io/genomeark/Cygnus_olor/). However, following the reviewer’s question we were unable to completement these data by running 10X on the black swan genome reads as unfortunately 10X Genomics discontinued their Chromium Linked-Reads product in 2020. Instead, we ran standard Illumina short read DNA sequencing to address the reviewer’s comments and reported the QV and completeness values using Merqury. These are now included in Supplementary Table 1 of the revised manuscript and are shown below:*

*Species Black swan Mute swan*

*Heterozygosity 0.5% 0.4%*

*QV 40.9373 38.5247*

*Completeness value 95.0324% 94.4407%*

*The methods for this additional work is listed in the online methods Lines 2 – 22.*

2. There seems to be a big difference of annotated genes between the Black (15478) and Mute (14791) swans, as outlined in Table 1. I also looked at the gene number in the goose genome , which is 16150, see Lu, L. et al. 2015 “The goose genome sequence leads to insights into the evolution of waterfowl and susceptibility to the fatty liver”, Genome Biology. I noticed that the quality of the mute swan is not as good as the black swan, judging from scaffold N50. Ideally, I would also like to see the contig N50. For assemblies from PacBio CLR reads, gene annotation could be affected if base polishing is not properly done. The authors may need to explain the gene difference between the two swans. If it is due to assembly quality, it should be mentioned in the manuscript. Or this is due to difference in swan genetics.

*Reply: In response to the reviewer’s comments we have now added the contig N50 information to Table 1. It is difficult to explain the differences in the number of annotated genes between the two species. The reviewer is correct in noting that this could reflect either assembly or biological differences. However, arguing against biological differences is the observation that gene gain/loss was more or less equivalent in both species. Indeed, the higher QV value of the black swan genome (see question 1) may be suggestive of improved polishing of this genome. Nevertheless, the possibility of both these options is now discussed in Lines 169-171 of the revised manuscript.*

3 Please provide plots of Hi-C maps for the curated black and mute assemblies.

*Reply: These are now included in Supplementary Figure 3 in the revised manuscript.*

4. Please run GenomeScope 2.0 to report heterozygosity using 10x reads

*Reply: As described in question 1, these data have now been included in Supplementary Table 1 of the revised manuscript.*

Reviewer #2:

1. Why choosing four candidate genes for plumage? Several other genes are known to be implicated in plumage evolution in birds, even when we consider only chicken (e.g., MC1R; ASIP; HERC2; TYR etc; Guo et al. 2010; Estalles C et al. 2022). It is not clear why these four were chosen to be investigated.

*Reply: A broader list of genes for plumage was indeed investigated, these results were simply not shown due to not notable difference between the black and mute swan. In response to the reviewer’s question we have now included Supplementary Table 5 in the revised manuscript which documents all the genes investigated in regards to plumage.*

2. Similarly, although TLR7 is clearly an important gene in the immune response against HPAI virus, many other immune genes (including other TLR genes) are as well. Chen at all 2013 presents an extensive list of genes involved in recognition of viruses in birds. Why has the focus been solely on TLR7? I don’t doubt these genes could indeed be implicated in the phenotypes investigated (as the evidence exists),

*Reply: Again, a broader list of genes previously implicated in influenza virus infection was investigated using the ISOSeq and RNASeq data (specifically we interrogated the influenza A KEGG pathway https://www.kegg.jp/kegg-bin/show_pathway?ko05164). The only striking difference between mute and black swans was noted in TLR7 expression. This additional information described in Lines 264-266 of the text.*

3. L117-118. The acronym VGP is used multiple times throughout the manuscript but never defined. It could be defined here.

*Reply: This is now corrected in the manuscript.*

4. L123-124. Why the discrepancy between the number of assembled chromosomes and the expected diploid number?

*Reply: Although we could identify several distinct scaffolds from the Hi-C contact maps (see supplementary figure on the Hi-C maps), we could only assign 34 autosomal and two-sex chromosomes. Others were nominated as unplaced scaffolds due to their lack of resolution to be placed as chromosomes. However, the largest 36 chromosomes had over 99% of the annotated genes; suggesting that uplaced scaffolds did not significantly alter the findings of the present study.*

5. L148-149. “Structural genomic differences have been associated with the differential susceptibility of chickens and ducks to avian influenza”. So why investigate only inversions? The rationale here should be clarified.

*Reply: We apologise for our lack of clarity here, we should have written inversions instead of structural variants. This has now been corrected in the revised manuscript.*

6. L152-153. Were the detected inverted regions only present in chromosome 1? It would be nice to see a figure or table with that information.

*Reply: In response to the reviewer’s suggestion, we have now included Supplemental Figure 7 in the revised manuscript which displays in the inverted regions present in chromosome 1. Inversions were recorded in the comparison of some other autosomal chromosomes but this was not viewed as highly biologically significant as they did not contain a high density of immune genes.*

7. L155-158. “However, given the absence of substantive structural variants between the black and mute swan, it is likely that any immunological consequences of these structural variants would be present in multiple swan species.” - if that’s the case, why to look for inversions in duck in the first place?

*Reply: We apologize for the confusion here. In evolutionary terms, ducks and chickens split earlier than ducks and swans. We also know that ducks and swans display differential susceptibility to highly pathogenic avian influenza (with ducks being more resistant than both species of swans). Therefore, we were not only interested in the genetic differences which explain the differential susceptibility of black and mute swans to HPAIV, but also in uncovering mechanism of resistance in closely related species i.e., duck.*

8. L189. Although assessing “whether isolation of the black swan resulted in changes in immune gene repertoire” would be very interesting, this is not actually what is being investigated here, since no alternative hypothesis is being assessed. What is being actually tested is whether the black swan (which happens to be isolated in Australia) has a different immune gene repertoire when compared to its close relatives. Any statement about the reasons for these differences can only be hypothesized.

*Reply: We agree with the reviewer in this point – we have now reworded this sentence to ask whether the black swan, which was isolated in Australia has different immune gene repertoire compared to its closest relatives.*

9. L284. I would argue this is not a striking finding. It is quite common that these higher-level GO terms, embracing a variety of other terms, end up as most significant. It is an inherent problem of these enrichment analysis.

*Reply: In accordance with reviewer’s suggestion we have now removed the work ‘Strikingly’ from this sentence in the revised manuscript.*

10. L454. Scaffolds. “s” is missing.

*Reply: We now fixed this in the revised manuscript.*

11. Table 1. A different symbol for micro and macro-chromosomes (e.g., † §) was probably meant here, since “*” is already assigned to ISO-seq coverage.

*Reply: This is indeed correct. We now fixed this in the revised manuscript.*

12. Figure 1. I am not a huge fan of the design of this figure. Is the amino acid sequence for black swan important to be shown? Maybe having all four species consistently shown as blue bar would make it more intelligible.

*Reply: We believe that for completeness it is important to show the amino acid sequence for the black swan. However, in accordance with the reviewer’s suggestion we have now shown all four species consistently in blue bars.*

13. Figure 2. The numbers at the tip of the tree are poorly aligned.

*Reply: We have now fixed this in the revised manuscript.*

14. Figure 3. Extra “a” before “(“at the figure title on the left.

*Reply: We have now fixed this in the revised manuscript.*

15. Figure 5. C “Biological process” on top is inconsistent with A and B.

*Reply: We have now fixed this in the revised manuscript.*

Suppl. The order of supplemental figures and tables do not follow the manuscript order neither are sorted in a logic way.

*Reply: We apologise that this was not clear. We have now re-listed all the supplementary figures followed by all the supplementary tables in a separate file to the manuscript in an attempt to clarify the order.*

**Second round of review**

**Reviewer 1**

Many thanks to the authors for the efforts in improving the manuscript. I am happy with the revised version and therefore recommend for publication.

**Reviewer 2**

Karawita et al. present a revised version of their manuscript "The swan genome and transcriptome: its not all black and white", in which they address the suggestions pointed out by the two reviewers. My comments touched on two main issues: (1) their limited list of candidate genes for plumage and immune response; (2) minor suggestions for figure improvement. I will go over how they addressed these comments:

(1) I pointed out that in the first version of the manuscript, the study seemed to take a candidate gene approach without giving a clear explanation for why certain genes were being particularly explored. In this version, authors clarified that a broad gene list was investigated, and made that clearer in the text.

(2) I also made some minor suggestion to improve figures, which have been followed by the authors. I must highlight however that although the authors affirm they added a blue bar to Figure 1, it looks identical to the first version. So I wonder if they might have added the wrong figure here.

The suggestions made by the other reviewer were appropriately addressed in the revised version of the manuscript.
